# The Vascular Flora of Pisa (Tuscany, Central Italy)

**DOI:** 10.3390/plants14030307

**Published:** 2025-01-21

**Authors:** Lorenzo Peruzzi, Brunello Pierini, Iduna Arduini, Gianni Bedini, Jacopo Franzoni

**Affiliations:** 1 PLANTSEED Lab, Department of Biology, University of Pisa, 56127 Pisa, Italy; lorenzo.peruzzi@unipi.it (L.P.); gianni.bedini@unipi.it (G.B.); 2 Independent Researcher, Via Zamenhof 2, 56127 Pisa, Italy; calcesano4@gmail.com; 3 Department of Agriculture, Food and Environment, University of Pisa, 56124 Pisa, Italy; iduna.arduini@unipi.it

**Keywords:** alien species, biodiversity, community science, citizen science, field botany, flora, native species, urban ecology

## Abstract

We present the first vascular flora of the municipality of Pisa. The floristic inventory was built on previous literature and field observations deposited in the online database Wikiplantbase #Toscana, integrated by observations from iNaturalist. The established flora of Pisa includes a total of 1404 specific and subspecific taxa (594 genera, 123 families), out of which 112 are alien species. *Silene subconica* is excluded from the regional flora of Tuscany, while *Solanum nitidibaccatum* is reported as a new regional casual alien, and the regional alien status of *Salpichroa origanifolia* shifts from naturalized to invasive. Native taxa exceed species-area predictions by 33.3%, attesting for a high floristic richness, and there are taxa of high biogeographical and conservation interest. However, also alien taxa exceed predictions by 34.9%, and there are many invasive species, pointing out a high anthropogenic impact in the territory of Pisa, mostly due to urbanization. The biological and chorological spectra reflect the coexistence of typical Mediterranean and central European habitats in this territory, especially within the Migliarino–San Rossore–Massaciuccoli Regional Park. The vascular flora of the municipality is quite rich, although threatened by anthropic pressures, fostering the arrival and establishment of invasive alien species.

## 1. Introduction

**Figure 1:**
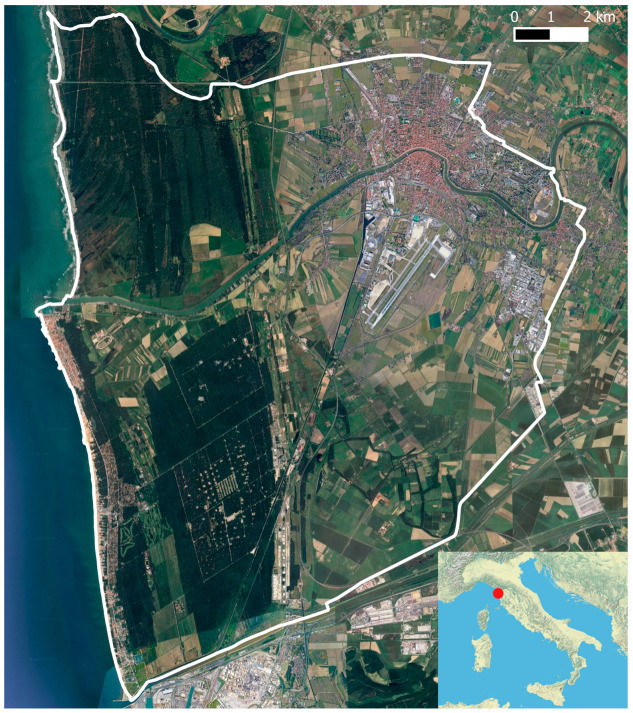
The location of Pisa in Italy. The white line indicates the study area, corresponding to the administrative border of the municipality of Pisa.

Urban floras, i.e., the inventories of native and/or alien plant species occurring in cities, are fundamental tools for science-based urbanistic planning, but also starting points for studies concerning the effect of human activities on ecological and evolutionary dynamics in urban environments [1,2,3]. Sharing this knowledge with citizens, together with identification tools, may help an active monitoring of urban biotas. Here, we provide the first checklist of vascular plant species occurring in the municipality of Pisa (Tuscany, Italy; Figure 1).

Pisa is home to the world’s first academic botanical garden, founded in 1543 [4], so that surrounding areas of this city have been explored and studied by botanists for almost 400 years. The first comprehensive work on the flora of the surroundings of Pisa was published by Savi in 1798 [5]. This work was among the main sources for the regional floristic treatment later published by Caruel [6], then supplemented by Baroni [7]. In recent times, a flora of the province of Pisa was assembled by Garbari and Borzatti von Loewenstern [8]. Despite this, a comprehensive and updated checklist of the vascular plant species growing in the municipality of Pisa is still missing. Many vegetation and floristic records were published in the last 70 years, especially concerning the natural and semi-natural areas surrounding Pisa, falling in the “Parco Naturale Regionale Migliarino–San Rossore–Massaciuccoli” (MSRM Regional Park hereinafter) [9,10,11,12,13,14,15,16,17,18,19,20,21,22,23,24,25,26,27,28,29,30,31,32,33,34,35,36,37,38,39,40,41,42,43,44,45,46,47,48,49,50,51,52].

Accordingly, the aim of this work was to provide an updated flora of the municipality of Pisa by summarizing floristic records from previous published literature and field observations, and by also including and carefully reviewing the records deposited in the online database Wikiplantbase #Toscana (https://bot.biologia.unipi.it/wpb/toscana/index, accessed on 26 December 2024; [53,54]). This inventory will also be used in the IDEM FLOS project, which proposes an innovative approach based on citizen science (or ‘community science’, as recently proposed by Christian et al. [55]) by providing interactive identification tools for a broad public, to discover and monitor the vascular plant species composition in urban areas. This updated inventory will contribute to urban biodiversity monitoring and support future ecological studies, especially within the MSRM Regional Park.

## 2. Results

The complete floristic inventory of the municipality of Pisa is reported in Supplementary Materials. The expected numbers of native and alien taxa according to the species-area relationship are 968 and 83, respectively.

The established flora (i.e., native and cryptogenic + naturalized and invasive alien taxa + hybrids) of the municipality of Pisa consists of 1404 specific and subspecific taxa. This figure includes 8 hybrids (2 native + 6 alien) and 112 aliens (67 naturalized, 45 invasive) (Table 1). Casual aliens are 73, while 62 taxa are recorded as only cultivated. A total of 314 (287 native and 27 alien) taxa historically reported in the area were not confirmed after 1965, 6 were assessed as locally extinct, 10 were doubtfully occurring, and one (*Bellevalia webbiana* Parl.) was excluded from the study area. *Silene subconica* Friv. is excluded from the regional flora of Tuscany (see Supplementary Materials for more details), while *Solanum nitidibaccatum* Bitter is reported as a new regional casual alien, and the regional alien status of *Salpichroa origanifolia* (Lam.) Baill. shifts from naturalized to invasive. A total of 72 new taxa (53 cultivated, 9 native, 6 casual, 2 naturalized, 1 invasive alien, 1 cryptogenic) and 5 native taxa confirmed were obtained by contrasting the data from Wikiplantbase #Toscana and the list of observations from iNaturalist.

Among the 594 genera recorded, *Trifolium* (38) and *Carex* (31) are the most species-rich in the study area, followed by *Euphorbia* (21) and *Juncus* (21). Out of the 123 families reported, the 3 most represented, accounting for 33% of the whole flora, are Poaceae (168), Asteraceae (159), and Fabaceae (138).

**Figure 2:**
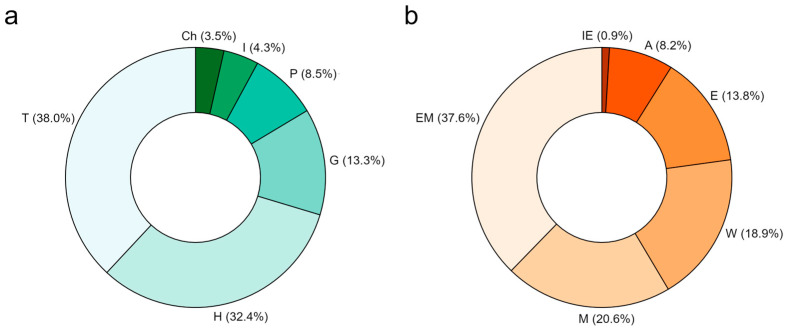
Biological (**a**) and chorological (**b**) spectra of the flora of the municipality of Pisa. (**a**) Biological spectrum: Ch, chamaephytes; H, hemicryptophytes; G, geophytes; I, hydrophytes; P, phanerophytes; T, therophytes. (**b**) Chorological spectrum: A, alien; E, Eurosiberian; EM, Eurosiberian-Mediterranean; IE, Italian endemics; M, Mediterranean; W, wide distribution.

According to the biological spectrum (Figure 2a), the most abundant life forms are therophytes (38.0%) and hemicryptophytes (32.4%), followed by geophytes (13.3%). The perennial woody component is represented by 8.5% of phanerophytes and 3.5% of chamaephytes. Given the presence in the study area of the Arno River and many wetlands, swamps, and canals, a not negligible proportion of hydrophytes (4.3%) is also observed.

Concerning the chorological spectrum of the flora of Pisa (Figure 2b), the transitional Eurosiberian–Mediterranean (37.6%) element is the most abundant, followed by Mediterranean (20.6%), taxa showing wider distribution (18.9%), and Eurosiberian elements (13.8%). Established alien taxa are 8.2% of the total flora, whereas Italian endemics are just 0.9%.

The Italian endemics recently reported for the municipality of Pisa are 12: *Cardamine apennina* Lihová & Marhold, *Centaurea aplolepa* Moretti subsp. *subciliata* (DC.) Arcang., *Colchicum neapolitanum* (Ten.) Ten. subsp. *neapolitanum*, *Crocus biflorus* Mill., *Daucus broteroi* Ten., *Digitalis micrantha* Roth ex Schweigg., *Ophrys sphegodes* Mill. subsp. *classica* (Devillers-Tersch. & Devillers) Kreutz, *O. sphegodes* subsp. *maritima* (Pacifico & Soca) Kreutz, *Ornithogalum exscapum* Ten., *Polygala nicaeensis* Risso ex W.D.J.Koch subsp. *italiana* (Chodat) Arrigoni, *Scabiosa uniseta* Savi, and *Solidago virgaurea* L. subsp. *litoralis* (Savi) Briq. & Cavill. *Polygala flavescens* DC. subsp. *flavescens* was also reported by previous authors but was not confirmed in this study.

## 3. Discussion

The native floristic richness in Pisa exceeds expectations by 33.3%, a higher value than the geographically close Monte Pisano (+19.8% [56,57]). This high floristic richness could be due to the habitat heterogeneity of the study area (see Study Area in Materials and Methods), well known to foster suitable conditions for the survival of species with different ecological preferences [58]. Considering only the native taxa documented for the study area after 1965, their number still exceeds expectations by 3.6%, but also possibly suggests a rarefaction of the native flora in the last 60 years. On the other hand, the number of established alien taxa also exceeds expectations by 34.9%, comparable to other highly impacted urban areas, as for instance Empoli in the Arno valley (+207%, [59]). Urbanization is indeed well known as a potential driver in increasing the invasive potential of alien plant species [60].

From a phytogeographical perspective, the municipality of Pisa represents the limit of the distribution range for a number of taxa, such as *Crepis bellidifolia* Loisel. and *Euphorbia pithyusa* L. subsp. *pithyusa* (eastern limit), *Centaurea sphaerocephala* L. subsp. *sphaerocephala*, *Euphorbia biumbellata* Poir. and *Paronychia echinulata* Chater (northeastern limit), *Verbascum phoeniceum* L. (southwestern limit). A small, isolated population of *Symphytum tanaicense* Steven is located in the study area, representing the extreme southwestern distribution limit of this species range. Interestingly, also *Crepis suffreniana* (DC.) Steud. would be at its disjunct western range limit; however, the actual distribution in Italy of this species should be further investigated. Other species occur in the study area at the margin of their distribution range, nationally and/or globally, such as *Polygala monspeliaca* L., *Scolymus maculatus* L., *Stipellula capensis* (Thunb.) Röser & H.R.Hamasha, *Trigonella segetalis* (Brot.) Coulot & Rabaute, and *Trigonella sicula* (Turra) Coulot & Rabaute (northern margin) or *Phagnalon sordidum* (L.) Rchb. (northeastern margin). *Polygonatum odoratum* (Mill.) Druce and *Veronica montana* L. usually grow at higher elevations but can be found close to the sea level in the municipality of Pisa. Moreover, six taxa reported in the floristic inventory have been described on material originating from the surroundings of Pisa: *Allium savii* Parl., *Festuca segetum* Savi (≡*Trisetaria segetum* (Savi) Soldano), *Lamium bifidum* Cirillo, *Lavatera arborea* L. (=*Malva arborea* (L.) Webb & Berthel.), *Trifolium michelianum* Savi, and *T. vesiculosum* Savi [61].

The taxa included in the Red Lists of the Italian Flora [62,63] are 43: 22 were assessed as Least Concern, 2 as Data Deficient, 5 as Near Threatened (*Allium savii* Parl., *Epipactis palustris* (L.) Crantz, *Osmunda regalis* L., *Ranunculus baudotii* Godr., *Zannichellia palustris* L.), 4 as Vulnerable (*Butomus umbellatus* L., *Leucojum aestivum* L. subsp. *aestivum*, *Ranunculus ophioglossifolius* Vill., *Thelypteris palustris* Schott), 9 as Endangered (*Anacamptis palustris* (Jacq.) R.M.Bateman, Pridgeon & M.W.Chase, *Baldellia ranunculoides* (L.) Parl., *Cardamine apennina* Lihová & Marhold, *Centaurea aplolepa* Moretti subsp. *subciliata* (DC.) Arcang., *Hottonia palustris* L., *Hydrocotyle vulgaris* L., *Sagittaria sagittifolia* L., *Solidago virgaurea* L. subsp. *litoralis* (Savi) Briq. & Cavill., *Triglochin barrelieri* Loisel.), and 1 as Critically Endangered (*Symphytum tanaicense* Steven). Most of these taxa grow in freshwater or brackish wetlands, which, fortunately, are preserved in the MSRM Regional Park.

On a more concerning note, among the alien taxa occurring in the study area, *Ailanthus altissima* (Mill.) Swingle, *Alternanthera philoxeroides* (Mart.) Griseb., *Baccharis halimifolia* L., and *Ludwigia peploides* (Kunth) P.H.Raven subsp. *montevidensis* (Spreng.) P.H.Raven are included in the list of invasive alien species of Union concern under the European regulation UE 1143/2014 and the application regulations UE 2016/1141, 2017/1263, 2019/1262, and 2022/1203.

The abundance of hemicryptophytes and therophytes (Figure 2a) and of Eurosiberian–Mediterranean element (Figure 2b) reflects the coexistence of typical Mediterranean and central European habitats in this territory, especially within the MSRM Regional Park. Biological and chorological spectra are in line with those obtained from the flora of the neighboring areas, such as Monte Pisano [59] and Cerbaie hills [64]; however, hydrophytes are more represented in the flora of Pisa. Indeed, urban floras often reflect the species composition of the more natural surrounding areas [65].

The vascular flora of Pisa is still quite rich, although threatened by anthropic pressures, especially linked to the increase in alien invasive species, and this checklist will serve as an important basic reference for continued monitoring and informed conservation actions [66]. In addition, the checklist provided here will serve as a starting point to produce citizen-oriented identification tools that would hopefully help a broader public to discover, be aware, monitor, and possibly protect the plant diversity of the city.

## 4. Materials and Methods

### 4.1. Study Area

The municipality of Pisa lies in the lowest Arno plain (maximum elevation is 4 m a.s.l.) and occupies a surface of 185.3 km^2^. On the west, the municipality directly faces the Ligurian Sea, also including the estuary of the Arno River. In the north, the border between Pisa and its neighbor municipality, San Giuliano Terme, mainly follows the Arno River and the canal Fiume Morto, running from the Monte Pisano, near Caprona, down to the sea. In the south and the east, the boundaries with other municipalities (Cascina in the east, Collesalvetti and Livorno in the south) are less clear (Figure 1).

Since the whole municipality of Pisa is developed in a fluvial plain, most of its territory is a recent (Holocene) alluvial deposit, mainly composed of fine-grained sand mixed with silt. In a zone closer to the sea, consolidated sand, deposited from the Arno and Serchio rivers, forms an alternation of ridges (the old dunes) and depressions (the old inter-dunes), the latter often flooded year-round. The coastal area directly facing the sea is characterized by sandy coasts [67]. The latter coastal areas form part of the MSRM Regional Park, that are in close proximity to urbanized areas, especially in the southwestern portion of the municipality.

The whole municipality of Pisa is fully collocated within the Mediterranean bioclimatic region, with different potential vegetational series according to the distance from the sea. The eastern part, farther from the seaside, is included in the hygrophilous peninsular *geosigmetum* of riparian vegetation (*Salicion albae*, *Populion albae*, *Alno*-*Ulmion*); however, most of these areas are nowadays urbanized or used as cultivated fields. The coastal area of the MSRM Regional Park is included in the Tyrrhenian coastal *geosigmetum* of hygrophilous and marsh vegetation of backdune systems and coastal plains (*Carici remotae-Fraxinetum oxycarpae*, *Populion albae*, *Juncion maritimi*, *Magnocaricion elatae*, *Phragmiton australis*). The seashore is included within the psammophilous and halophilous peninsular *geosigmetum* of the vegetation of dune systems (*Salsolo kali*-*Cakiletum maritimae*, *Echinophoro spinosae*-*Elytrigietum junceae*, *Crucianellion maritimae*, *Malcolmietalia*, *Asparago*-*Juniperetum macrocarpae*, *Quercetalia ilicis*) [68,69].

Since the foundation of the city by the Etruscans in the 5th century B.C. [70], humans have deeply modified the surrounding landscape, influencing the native flora and vegetation. Indeed, the eastern part of the municipality is extremely anthropized, hosting the city of Pisa (43.721445 N, 10.401156 E), an international airport (43.686646 N, 10.394262 E), and agricultural land, with the exception of some relict strips of floodplain forest occurring in the “Tenuta di Coltano” (43.637958 N, 10.383394 E), south of Pisa, still in the MSRM Regional Park [22]. The western part of the municipality includes the “Tenuta di San Rossore” (43.719412 N, 10.311690 E), north of the Arno River, and the “Tenuta di Tombolo” (43.646420 N, 10.328506 E), south of the Arno, two portions of the MSRM Regional Park. These two areas are mainly characterized by an alternation of Mediterranean sclerophyllous vegetation, dominated by *Quercus ilex* L., in the above-mentioned dune ridges, and mesophilous and hygrophilous vegetation, with *Quercus robur* L. subsp. *robur*, *Fraxinus angustifolia* Vahl subsp. *oxycarpa* (M.Bieb. ex Willd.) Franco & Rocha Afonso, *Alnus glutinosa* (L.) Gaertn., and *Populus alba* L., in the inundated or waterlogged depressions [12]. Nevertheless, the landscape has been deeply influenced by the extensive cultivation of pines (*Pinus pinea* L. and *Pinus pinaster* Aiton subsp. *pinaster*) started in the 17th century [12]. Within the “Tenuta di San Rossore” closer to the sea, there are swamps, called “Lame di San Rossore” (43.701203 N, 10.290331 E), characterized by brackish and fresh water, in which halophilous and hygrophilous communities grow [17]. The vegetation of the sandy seashore is extremely degraded, mostly due to strong erosive phenomena in the “Tenuta di San Rossore” and to touristic over-exploitation of the coast south of the Arno estuary [16,18,19].

### 4.2. Floristic Inventory

The expected number (S) of native and alien taxa occurring in the study area based on its extension (A = 185.3 km^2^) was calculated using the Arrhenius’ species–area relationship S = _C_A^Z^, by using the c (245.2 and 10.1 for native and alien taxa, respectively) and z (0.263 and 0.404 for native and alien taxa, respectively) coefficients empirically calculated for the floristic richness of Italy by D’Antraccoli et al. [56].

A total of 12,002 floristic records were extracted from the Wikiplantbase #Toscana portal [53], a regional free online floristic database whose records are curated and validated by the editors [54]. A total of 7764 bibliographic records came from previous literature [6,7,9,10,11,12,13,14,15,16,17,18,19,20,21,22,23,24,25,26,27,28,29,30,31,32,33,34,35,36,37,38,39,40,41,42,43,44,45,46,47,48,49,50,51,52,71,72,73,74,75,76,77,78,79,80,81,82,83,84,85,86,87,88,89,90,91,92,93,94,95,96,97,98,99,100,101,102,103,104,105], 1492 from unpublished herbarium records, and 2747 from field observations uploaded in the last 10 years [53,54]. The resulting list was contrasted with a list of taxa deriving from the records stored in iNaturalist, a useful tool in floristic research [106,107,108]. Records of putatively new taxa were then individually checked for reliability by means of photographic documentation before inclusion in the floristic inventory.

Nomenclature was aligned with the two recently published checklists of the native [109] and alien [110] vascular flora of Italy, and with their updates made available through the Portal to the Flora of Italy (https://dryades.units.it/floritaly/, accessed 26 December 2024 [111]). Families of flowering plants were organized following the latest phylogenetic classification of the APG IV [112]. Taxa were considered as not confirmed if no records were available after 1965. Aliens recorded only historically (i.e., not confirmed) were automatically considered as casual aliens at the local level, irrespective of the regional alien status in Tuscany [110]. Once we compiled the floristic inventory, we cross-checked it with the species observed in the iNaturalist database (https://www.inaturalist.org, accessed 26 December 2024) within the municipality of Pisa. Life forms and chorotypes were derived from Pignatti [113,114,115] for all taxa, excluding cryptogenic species, cultivated and casual taxa, and hybrids. The retrieved chorotypes were compared and adjusted with the distribution data available on the Euro+Med database [116] and POWO [117]. Chorotypes were then included in the floristic regions proposed by Arrigoni [118] for Europe. Given the extremely low number of Mediterranean–Macaronesian (13) and Mediterranean–Iranoturanian (31) elements, they were aggregated into the Mediterranean element. Information on the Italian endemics was retrieved from Bartolucci et al. [109] and Peruzzi et al. [119,120]. We also obtained the available regional IUCN Red List categories from the Red Lists for the Italian Flora [62,63,121].

## Figures and Tables

**Table 1:** Number of taxa listed in the floristic inventory of the municipality of Pisa. P (present, i.e., recorded after 1965), NC (not confirmed, i.e., not recorded after 1965), Ex (locally extinct), D (doubtfully occurring), NP (not present, i.e., erroneously reported for the area). Numbers in bold are those constituting the established flora.

	P	NC	Ex	D	NP	Total
Native	**1002**	**287**	5	9	(1)	1304
Cryptogenic	**3**	**0**	0	0	0	3
Invasive alien	**45**	**0**	0	0	0	45
Naturalized alien	**67**	**0**	0	0	0	67
Casual alien	49	24	1	1	0	75
Cultivated	59	3	0	0	0	62
Total	1225	314	6	10	(1)	1556

## Data Availability

The complete floristic inventory and information about floristic records are available in Supplementary Materials.

## Supplementary Materials

The following supporting information can be downloaded at: https://www.mdpi.com/article/10.3390/plants14030307/s1.

## References

[1] Tanner C.J., Adler F.R., Grimm N.B., Groffman P.M., Levin S.A., Munshi-South J., Pataki D.E., Pavao-Zuckerman M., Wilson W.G. (2014). Urban ecology: Advancing science and society. Front. Ecol. Environ..

[2] Johnson M.T.J., Thompson K.A., Saini H.S. (2015). Plant evolution in the urban jungle. Am. J. Bot..

[3] Carta A., Forbicioni L., Frangini G., Pierini B., Peruzzi L. (2018). An updated inventory of the vascular flora of Elba Island (Tuscan Archipelago, Italy). Ital. Bot..

[4] Chiarugi A. (1953). Le date di fondazione dei primi orti botanici del mondo: Pisa (estate 1543); Padova (7 Luglio 1545); Firenze (1° Dicembre 1545). Nuovo Giorn. Bot. Ital..

[5] Savi G. (1798). Flora Pisana.

[6] Caruel T. (1860). Prodromo Della Flora Toscana.

[7] Baroni E. (1897). Supplemento Generale al Prodromo Della Flora Toscana di T. Caruel.

[8] Garbari F., Borzatti von Loewenstern A. (2005). Flora pisana: Elenco annotato delle piante vascolari della provincia di Pisa. Atti Soc. Toscana Sci. Nat. Mem. Ser. B.

[9] Savelli M. (1915). Brevi notizie su alcune piante naturalizzatesi nei dintorni di Pisa. Bull. Soc. Bot. Ital..

[10] Corti R. (1956). Ricerche sulla vegetazione dell’Etruria. X. Aspetti geobotanici della selva costiera. La Selva Pisana a S. Rossore e l’importanza di questa formazione relitta per la storia della vegetazione mediterranea. Nuovo Giorn. Bot. Ital. n.s..

[11] Montelucci G. (1962). Avventizie nuove e antiche sul litorale pisano-versiliese. Nuovo Giorn. Bot. Ital. n.s..

[12] Gellini R., Pedrotti F., Venanzoni R. (1986). Le associazioni forestali ripariali e palustri della selva di San Rossore (Pisa). Doc. Phytosoc..

[13] Coaro E. (1987). Flora e vegetazione del Bosco dell’Ulivo. Quad. Mus. St. Nat. Livorno.

[14] Garbari F. (2001). La flora di S. Rossore (Pisa) aggiornata al 1999. Atti Soc. Toscana Sci. Nat. Mem. Ser. B.

[15] Tomei P.E., Bertacchi A., Sani A., Consiglio M. (2004). La Vegetazione Della Tenuta di S. Rossore. Note Esplicative Della Carta Della Vegetazione di San Rossore 1:10,000.

[16] Sani A., Tomei P.E. (2006). La vegetazione psammofila del litorale di San Rossore (Toscana settentrionale) e la sua importanza conservazionistica. Parlatorea.

[17] Bertacchi A., Lombardi T., Tomei P.E. (2007). Le aree umide salmastre della Tenuta di San Rossore (PI): Zonazione e successione delle specie vegetali in relazione alla salinità del suolo. Inter Nos.

[18] Bertacchi A., Lombardi T., Bocci G. (2009). Il paesaggio vegetale dell’ambiente dunale di Calambrone nel litorale pisano (Toscana settentrionale). Inform. Bot. Ital..

[19] Bertacchi A., Lombardi T., Vecci L. (2010). Gli ambienti dunali di Tirrenia (PI): Aspetti vegetazionali e floristici. Inter Nos.

[20] Bertacchi A., Lombardi T. (2014). Diachronic analysis (1954–2010) of transformations of the dune habitat in a stretch of the northern Tyrrhenian coast (Italy). Plant Biosyst..

[21] Bertacchi A., Lombardi T. (2014). *Spartina versicolor* Fabre in coastal areas of Tuscany (Italy). Contrib. Bot..

[22] Bertacchi A., Lombardi T. (2016). I boschi di Coltano: Aspetti storici, fisionomici e vegetazionali di un paesaggio forestale relitto nella pianura di Pisa (Toscana). Atti Soc. Toscana Sci. Nat. Mem. Ser. B.

[23] Lazzeri V., Buono V., Canzonieri A., Longo D., Nicolella G. (2022). Contributo alla Flora vascolare delle zone umide di Toscana I. La flora dell’area umida di Stagno (Pisa). Acta Plantarum Notes 8.

[24] Corti R. (1951). *Stipa trichotoma* Nees nella selva di San Rossore, nuovo inquilino della flora toscana. Nuovo Giorn. Bot. Ital. n.s..

[25] Anzalone B. (1979). La diffusione di *Artemisia annua* L. in Italia. Inform. Bot. Ital..

[26] Anzalone B., Brilli-Cattarini A.J.B. (1980). Segnalazioni floristiche italiane: 26. *Cyperus eragrostis* Lam. (Cyperaceae). Inform. Bot. Ital..

[27] Marchetti D. (2003). Notule pteridologiche italiche. II (32–63). Ann. Mus. Civ. Rovereto Sez. Arch. St. Sc. Nat..

[28] Sezione Toscana della Società Botanica Italiana (2005). Notule Floristiche Per la Toscana.

[29] Peruzzi L., Pierini B., Tison J.-M. (2007). Notulae alla checklist della flora vascolare italiana, 5: 1435–1438. Inform. Bot. Ital..

[30] Peruzzi L., Viciani D., Bedini G. (2009). Contributi per una flora vascolare di Toscana. I. (1–85). Atti Soc. Toscana Sci. Nat. Mem. Ser. B.

[31] Gestri G., Alessandrini A., Siriotti M., Carta A., Peruzzi L. (2010). Contributo alla conoscenza della flora vascolare endemica di Toscana ed aree contermini. 2. *Bellevalia webbiana* Parl. (Asparagaceae). Inform. Bot. Ital..

[32] Orlandi C., Arduini I. (2010). Note ad integrazione della flora di San Rossore (Pisa). Inform. Bot. Ital..

[33] Peruzzi L., Viciani D., Bedini G. (2011). Contributi per una flora vascolare di Toscana. III (143–180). Atti Soc. Toscana Sci. Nat. Mem. Ser. B.

[34] Pierini B. (2011). Notulae alla flora esotica d’Italia, 5: 99. Inform. Bot. Ital..

[35] Alessandrini A., Buono V., Lazzeri V., Magni C., Manni Q.G., Nicolella G. (2013). Acta Plantarum Notes 1.

[36] Iamonico D., Lastrucci L., Viciani D. (2013). Notulae alla checklist della flora vascolare italiana, 15: 1965. Inform. Bot. Ital..

[37] Lazzeri V., Mascia F., Sammartino F., Campus G., Caredda A., Carlesi V., Fois M., Gestri G., Mannocci M., Mazzoncini V., Alessandrini A., Buono V., Lazzeri V., Longo D., Magni C., Manni Q.C., Nicolella G. (2013). Novità floristiche per le regioni Sardegna e Toscana. Acta Plantarum Notes 2.

[38] Peruzzi L., Viciani D., Bedini G. (2013). Contributi per una flora vascolare di Toscana. V (247–319). Atti Soc. Toscana Sci. Nat. Mem. Ser. B.

[39] Pierini B. (2013). Notulae alla checklist della flora vascolare italiana, 16: 2010. Inform. Bot. Ital..

[40] Lazzeri V. (2014). Note floristiche tosco–sarde III: Novità per le regioni Toscana e Sardegna. Quad. Mus. St. Nat. Livorno.

[41] Peruzzi L., Viciani D., Bedini G. (2014). Contributi per una flora vascolare di Toscana. VI (320–356). Atti Soc. Toscana Sci. Nat. Mem. Ser. B.

[42] von Raab-Straube E., Raus T. (2015). Euro+ Med–checklist notulae, 4 [notulae ad floram Euro–Mediterraneam pertinentes 33]. Willdenowia.

[43] Galasso G., Domina G., Adorni M., Ardenghi N.M.G., Banfi E., Bedini G., Bertolli A., Brundu G., Calbi M., Cecchi L. (2016). Notulae to the Italian alien vascular flora: 1. Ital. Bot..

[44] Galasso G., Domina G., Ardenghi N.M.G., Arrigoni P., Banfi E., Bartolucci F., Bonari G., Buccomino G., Ciaschetti G., Conti F. (2016). Notulae to the Italian alien vascular flora: 2. Ital. Bot..

[45] Peruzzi L., Viciani D., Bedini G. (2016). Contributi per una flora vascolare di Toscana. VII (357–439). Atti Soc. Toscana Sci. Nat. Mem. Ser. B.

[46] Peruzzi L., Viciani D., Agostini N., Angiolini C., Ardenghi N.M.G., Astuti G., Bardaro M.R., Bertacchi A., Bonari G., Boni S. (2017). Contributi per una flora vascolare di Toscana. VIII (440–506). Atti Soc. Toscana Sci. Nat. Mem. Ser. B.

[47] Peruzzi L., Viciani D., Angiolini C., Astuti G., Banfi E., Benocci A., Bonari G., Bruni G., Caramante P., Caré M. (2017). Contributi per una flora vascolare di Toscana. IX (507–605). Atti Soc. Toscana Sci. Nat. Mem. Ser. B.

[48] Galasso G., Domina G., Alessandrini A., Ardenghi N.M.G., Bacchetta G., Ballelli S., Bartolucci F., Brundu G., Buono S., Busnardo G. (2018). Notulae to the Italian alien vascular flora: 6. Ital. Bot..

[49] Peruzzi L., Viciani D., Angiolini C., Astuti G., Banfi E., Bardaro M.R., Bianchetto E., Bonari G., Cannucci S., Cantini D. (2018). Contributi per una flora vascolare di Toscana. X (606–663). Atti Soc. Toscana Sci. Nat. Mem. Ser. B.

[50] Peruzzi L., Viciani D., Angiolini C., Apruzzese M., Banfi E., Bonini I., Bonari G., Calvia G., Carta A., Castagnini P. (2020). Contributi per una flora vascolare di Toscana. XII (739–812). Atti Soc. Toscana Sci. Nat. Mem. Ser. B.

[51] Galasso G., Domina G., Angiolini C., Bacchetta G., Banfi E., Barberis D., Bardi S., Bartolucci F., Bonari G., Bovio M. (2021). Notulae to the Italian alien vascular flora: 12. Ital. Bot..

[52] Peruzzi L., Viciani D., Adami M., Angiolini C., Astuti G., Bonari G., Bonaventuri G., Castagnini P., De Simone L., Domina G. (2021). Contributi per una flora vascolare di Toscana. XIII (813–873). Atti Soc. Toscana Sci. Nat. Mem. Ser. B.

[53] Wikiplantbase #Toscana. https://bot.biologia.unipi.it/wpb/toscana/index.

[54] Bedini G., Pierini B., Roma-Marzio F., Caparelli K.F., Bonari G., Dolci D., Gestri G., D’Antraccoli M., Peruzzi L. (2016). Wikiplantbase #Toscana, breaking the dormancy of floristic data. Plant Biosyst..

[55] Christian C., Gwilliam G.F., von Konrat M., Ahn J., Bailey C., Dodinval D., Ellwood E.R., Golembiewski K., Higgins L.M., Jones C. (2024). Embracing inclusivity: The case against the term ‘citizen science’. RIO.

[56] D’Antraccoli M., Peruzzi L., Conti F., Galasso G., Roma-Marzio F., Bartolucci F. (2023). Floristic richness in a mediterranean hotspot: A journey across Italy. Plants.

[57] Pierini B., Garbari F., Peruzzi L. (2009). Flora vascolare del Monte Pisano (Toscana nord-occidentale). Inform. Bot. Ital..

[58] Deshaye J., Morisset P. (1988). Floristic Richness, Area, and Habitat Diversity in a Hemiarctic Archipelago. J. Biogeogr..

[59] Peruzzi L. (2023). The vascular flora of Empoli (Tuscany, central Italy). Ital. Bot..

[60] Kühn I., Wolf J., Schneider A. (2017). Is there an urban effect in alien plant invasions?. Biol. Invasions.

[61] Peruzzi L., Galasso G., Domina G., Bartolucci F., Santangelo A., Alessandrini A., Astuti G., D’Antraccoli M., Roma-Marzio F., Ardenghi N.M.G. (2019). An inventory of the names of native, non-endemic vascular plants described from Italy, their loci classici and types. Phytotaxa.

[62] Rossi G., Montagnani C., Gargano D., Peruzzi L., Abeli T., Ravera S., Cogoni A., Fenu G., Magrini S., Gennai M. (2013). Lista Rossa Della Flora Italiana. 1. Policy Species e Altre Specie Minacciate.

[63] Rossi G., Orsenigo S., Gargano D., Montagnani C., Peruzzi L., Fenu G., Abeli T., Alessandrini A., Astuti G., Bacchetta G. (2020). Lista Rossa Della Flora Italiana. 2 Endemiti e Altre Specie Minacciate.

[64] Gestri G., Pierini B., D’Antraccoli M., Bernardini A., Peruzzi L. (2023). An updated inventory of the vascular flora of the Cerbaie hills (Tuscany, Italy). Ital. Bot..

[65] Grapow L., Blasi C. (1998). A comparison of the urban flora of different phytoclimatic regions in Italy: Italian urban floras. Glob. Ecol. Biogeogr..

[66] Peruzzi L. (2018). Floristic inventories and collaborative approaches: A new era for checklists and floras?. Plant Biosyst..

[67] Carosi R., Montomoli C., Pertusati P.C., Sarti G., Frassi C., Leoni L. (2003). Note Illustrative Della Carta Geologica d’Italia Alla Scala 1:50,000. Foglio 273 Pisa.

[68] De Dominicis V., Angiolini C., Gabellini A., Blasi C. (2010). Carta della serie di vegetazione della Toscana. La Vegetazione d’Italia Carta Della Ser. di Vegetazione, Scala 1:500,000.

[69] De Dominicis V., Angiolini C., Gabellini A., Blasi C. (2010). La serie di vegetazione della regione Toscana. La Vegetazione d’Italia.

[70] Sarti G., Bini M., Giacomelli S. (2010). The growth and decline of Pisa (Tuscany, Italy) up to the Middle Ages: Correlations with landscape and geology. Il Quat..

[71] Caruel T. (1870). Secondo Supplemento Prodromo Della Flora Toscana di T. Caruel.

[72] Levier E., Sommier S. (1891). Addenda ad floram Etruriae. Nuovo Giorn. Bot. Ital..

[73] Fiori A. (1943). Flora Italica Cryptogama, 5. Pteridophyta.

[74] Corti R. (1970). Visita alla tenuta di San Rossore. Inform. Bot. Ital..

[75] Mainardi R. (1982). Alcune considerazioni sulla nidificazione del cavaliere d’Italia *Himantopus himantopus* (L.) all’Ulivo (Pisa). Quad. Mus. St. Nat. Livorno.

[76] Ferrarini E., Ciampolini F., Pichi Sermolli R.E.G., Marchetti D. (1986). Iconographia palynologica pteridophytorum Italiae. Webbia.

[77] Baldini R.M. (1993). The genus *Phalaris* L. (Gramineae) in Italy. Webbia.

[78] Soldano A. (1993). Il genere *Oenothera* L., subsect. Oenothera, in Italia (Onagraceae). Nat. Brescia..

[79] Falciani L. (1997). Systematic revision of *Stachys* sect. *Eriostomum* (Hoffmans. & Link) Dumort. in Italy. Lagascalia.

[80] Corsi G., Garbari F., Maffei F. (1999). Il genere *Urtica* L. (Urticaceae) in Italia. Revisione biosistematica. Webbia.

[81] Pedullà M.L., Garbari F. (2002). Piante di interesse biogeografico ecologico nei canali di bonifica della pianura pisana. Atti Soc. Toscana Sci. Nat. Mem. Ser. B.

[82] Bottega S., Garbari F. (2003). II genere *Symphytum* L. (Boraginaceae) in Italia. Revisione biosistematica. Webbia.

[83] Pignotti L. (2003). *Scirpus* L. and related genera (Cyperaceae) in Italy. Webbia.

[84] Pedullà M.L., Garbari F. (2004). La Flora della rete di canalizzazione della pianura nord-occidentale pisana. Quad. Mus. St. Nat. Livorno.

[85] Carta A., Pierini B., Peruzzi L. (2008). Aggiornamenti e novità sulla distribuzione di *Isoëtes gymnocarpa* e *I. histrix* (Lycopodiophytina) in Toscana. Atti Soc. Toscana Sci. Nat. Mem. Ser. B.

[86] Carta A., Pierini B., Peruzzi L. (2008). Distribuzione di *Ophioglossum lusitanicum* L. (Psilotopsida) in Toscana. Inform. Bot. Ital..

[87] Bedini G., Carta A., Garbari F., Peruzzi L. (2011). Schede per una Lista Rossa della Flora vascolare e crittogamica italiana: *Hypericum elodes* L.. Inform. Bot. Ital..

[88] Welti E.M., Ansaldi M., Carta A., Bedini G. (2011). Distribuzione del genere *Epipactis* (Orchidaceae) in provincia di Pisa. Atti Soc. Toscana Sci. Nat. Mem. Ser. B.

[89] Lazzaro L., Ferretti G., Galasso G., Lastrucci L., Foggi B. (2013). Contributo alla conoscenza della flora esotica dell’Arcipelago Toscano, Italia. Nat. Hist. Sci..

[90] Petraglia A. (2013). Consulenza Specialistica per l’acquisizione di Dati Mediante Rilievi Fitosociologici e Floristici Delle Principali Zone Umide del Parco Migliarino, San Rossore, Massaciuccoli.

[91] Tomei P.E., Camangi F. (2014). Tradizioni Alimurgiche in Toscana.

[92] Lombardi T. (2015). Monitoraggio Vegetazionale e Floristico Delle Aree Interessate Dalle Azioni, Coordinamento Scientifico Interventi di Compensazione. Relazione Tecnica Per Progetto Allestimento Route Nazionale R–S 2014.

[93] Coulot P., Rabaute P. (2016). Monographie des Leguminosae de France. Tome 4. Tribù des Fabeae, des Cicereae et des Genisteae.

[94] Saggese A. (2016). Studi Floristici e Vegetazionali Delle Aree Umide Salmastre Della Toscana Settentrionale: Il Caso di Galanchio. Master’s Thesis.

[95] Arrigoni P.V. (2018). Flora Analitica Della Toscana.

[96] Arrigoni P.V. (2019). Flora Analitica Della Toscana.

[97] Bonari G., Knollová I., Vlčková P., Xystrakis F., Çoban S., Sağlam C., Didukh Y.P., Hennekens S.M., Acosta A.T.R., Angiolini C. (2019). CircumMed Pine Forest Database: An electronic archive for mediterranean and submediterranean pine forest vegetation data. Phytocoenologia.

[98] Arrigoni P.V. (2020). Flora Analitica Della Toscana.

[99] Lazzeri V. (2021). Ecology of *Baccharis halimifolia* L. in Tuscany (Italy) and its impacts on native vegetation: Where are we and where are we going to?. Quad. Mus. St. Nat. Livorno.

[100] Arduini I., Alessandrini V. (2024). The novel invader *Salpichroa origanifolia* modifies the soil seed bank of a mediterranean mesophile forest. Plants.

[101] Lastrucci L., Saiani D., Mugnai A., Ferretti G., Viciani D. (2024). Updating the distribution of the genus *Callitriche* (Plantaginaceae) in Italy from the study of the Herbarium Centrale Italicum collections. Medit. Bot..

[102] Särkinen T., Poczai P., Barboza G.E., Van Der Weerden G.M., Baden M., Knapp S. (2018). A revision of the Old World black nightshades (morelloid clade of *Solanum* L., Solanaceae). PhytoKeys.

[103] Arduini I., Ercoli L. (2012). Recovery of understory vegetation in clear-cut stone pine (*Pinus pinea* L.) plantations. Plant Biosyst..

[104] Montelucci G. (1934). L’*Artemisia verlotorum* Lamotte a Roma e in altre località italiane. Nuovo Giorn. Bot. Ital. n.s..

[105] Montelucci G. (1964). Ricerche sulla vegetazione dell’Etruria XIII. Materiali per la flora e la vegetazione di Viareggio. Webbia.

[106] Khapugin A., Silaeva T., Fedasheva E., Tyapukhina M., Guryanova A., Shlyapkina V., Esina I., Kochetkova A., Konusova D., Mukletsova N. (2021). Additions to the vascular plant flora of the Republic of Mordovia (Russia): Contribution of the iNaturalist platform. Contrib. Bot..

[107] Hicks A. (2022). Standards for Conducting an iFlora: Using iNaturalist to Conduct a Vascular Flora of the Charles B Henson Cave Preserve in Dade County, Georgia. Honors Thesis.

[108] White E., Soltis P.S., Soltis D.E., Guralnick R. (2023). Quantifying error in occurrence data: Comparing the data quality of iNaturalist and digitized herbarium specimen data in flowering plant families of the southeastern United States. PLoS ONE.

[109] Bartolucci F., Peruzzi L., Galasso G., Alessandrini A., Ardenghi N.M.G., Bacchetta G., Banfi E., Barberis G., Bernardo L., Bouvet D. (2024). A second update to the checklist of the vascular flora native to Italy. Plant Biosyst..

[110] Galasso G., Conti F., Peruzzi L., Alessandrini A., Ardenghi N.M.G., Bacchetta G., Banfi E., Barberis G., Bernardo L., Bouvet D. (2024). A second update to the checklist of the vascular flora alien to Italy. Plant Biosyst..

[111] Martellos S., Bartolucci F., Conti F., Galasso G., Moro A., Pennesi R., Peruzzi L., Pittao E., Nimis P.L. (2020). FlorItaly—The Portal to the Flora of Italy. PhytoKeys.

[112] The Angiosperm Phylogeny Group (2016). An update of the Angiosperm Phylogeny Group classification for the orders and families of flowering plants: APG IV. Bot. J. Linn. Soc..

[113] Pignatti S., Guarino R., La Rosa M. (2017). Flora d’Italia.

[114] Pignatti S., Guarino R., La Rosa M. (2017). Flora d’Italia.

[115] Pignatti S., Guarino R., La Rosa M. (2018). Flora d’Italia.

[116] Euro+Med PlantBase—The Information Resource for Euro-Mediterranean Plant Diversity. http://www.europlusmed.org.

[117] Plants of the World Online. https://powo.science.kew.org.

[118] Arrigoni P.V. (1983). Aspetti corologici della flora sarda. Biogeographia.

[119] Peruzzi L., Conti F., Bartolucci F. (2014). An inventory of vascular plants endemic to Italy. Phytotaxa.

[120] Peruzzi L., Domina G., Bartolucci F., Galasso G., Peccenini S., Raimondo F.M., Albano A., Alessandrini A., Banfi E., Barberis G. (2015). An inventory of the names of vascular plants endemic to Italy, their loci classici and types. Phytotaxa.

[121] Orsenigo S., Fenu G., Gargano D., Montagnani C., Abeli T., Alessandrini A., Bacchetta G., Bartolucci F., Carta A., Castello M. (2021). Red List of threatened vascular plants in Italy. Plant Biosyst..

