# Supplementary material for: The Vascular Flora of Pisa (Tuscany, Central Italy)
Source: Plants (Basel). 2025 Jan 21;14(3):307. doi: 10.3390/plants14030307 (PMC11819712; doi:10.3390/plants14030307)
Supplement: Supplementary file 1 [file plants-14-00307-s001.zip › plants-3424047-supplementary.pdf]

Supplementary Materials: Floristic inventory and records of vascular plant taxa of the municipality of Pisa.
D = Doubtfully occurring
Ex = Locally extinct
NC = No longer recorded (presence of a reliable historical record before 1965)
NP = Recorded by mistake
Native (or putatively native) plants are in bold in the list; naturalized alien plants are not in bold, while casual aliens and/or cultivated taxa, but also NP taxa, are in italics.

Lycopodiidae

Isoetaceae

NC **Isoëtes durieui** Bory
G bulb - Stenomedit.-Occid.
Literature data: Caruel 1870; Baroni 1897-1908

NC **Isoëtes gymnocarpa** (Gennari) A.Braun
G bulb - S-Medit.
Literature data: Carta et al. 2008a [*Arcangeli*, 1863, FI; *Beccari*, 1863, FI; *s. coll.*, 1864, PI]

NC **Isoëtes histrix** Bory
G bulb - Stenomedit.
Literature data: Caruel 1870; Baroni 1897-1908; Carta et al. 2008a [*s. coll.*, 1862, FI; *O. Beccari*, 1863, FI; *O. Beccari*, 1863, FI; *Ricci*, 1863, FI; *G. Arcangeli*, 1864, FI, PI; *P. Savi*, 1864, FI, PI; *s. coll.*, 1866, PI; *Marcucci*, 1869; *Fiori*, 1885, FI; *s. coll.*, 1886, PI; *Rossetti*, 1887, FI; *P. Fantozzi*, 1891, FI; *E. Barsali*, 1904, PI]

Ophioglossidae

Ophioglossaceae

NC **Ophioglossum lusitanicum** L.
G rhiz - Stenomedit.-Subatl.
Literature data: Caruel 1870; Baroni 1897-1908; Carta et al. 2008b [*Mazzanti*, 1875, PI]

NC **Ophioglossum vulgatum** L.
G rhiz - Circumbor.
Literature data: Caruel 1870; Baroni 1897-1908

Equisetidae

Equisetaceae

Equisetum arvense L.
G rhiz - Circumbor.
Literature data: Caruel 1870; Baroni 1897-1908; Corti 1956, under the name *E. arvense* L. var. *agreste* Klinge; Garbari 2001; Pedullà & Garbari, 2004; Petraglia 2013
Field observations: I. Arduini, 2023

Equisetum palustre L.
G rhiz - Circumbor.
Literature data: Caruel 1870; Baroni 1897-1908; Gellini et al. 1986
Field observations: I. Arduini, 2023

Equisetum ramosissimum Desf.
G rhiz - Circumbor.
Literature data: Caruel 1870; Baroni 1897-1908; Fiori 1943, under the name *E. ramosissimum* Desf. var. *procerum* Asch. f. *polystachyum* Luerss.; Sani & Tomei 2006; Lazzeri in Buono et al. 2022
Herbarium data: L. Pinzani, 2021, Herb. Pinzani
Field observations: L. Peruzzi, 2019; L. Pinzani, 2020, 2021

Equisetum telmateia Ehrh.
G rhiz - Circumbor.
Literature data: Caruel 1870, Baroni 1897-1908, Fiori 1943, Corti 1956, under the name *E. maximum* Lam.; Garbari 2001; Tomei et al. 2004; Lazzeri 2021, 2022
Herbarium data: L. Pinzani, 2020, Herb. Pinzani
Field observations: H. Öhm, 2024; D. Marchetti, s.d.

Polypodiide

Osmundaceae

Osmunda regalis L.
G rhiz - Subcosmop.
Literature data: Tomei et al. 2004; Landi & Angiolini 2007 [*E. Barsali*, 1900, 1901, PI]
Assessed as NT in the Red List of the Italian Vascular Flora (Rossi et al. 2020)

Marsileaceae

Ex **Marsilea quadrifolia** L.
I rad - Circumbor.
Literature data: Baroni 1897-1908; Fiori 1943; Gentili et al. 2010; Arrigoni 2016 [*P.V. Arrigoni*, 1998, FI]
Unfortunately, this species was not found again in the study area after 2010.

Salviniaceae

NC *Azolla filiculoides* Lam.
Literature data: Baroni 1897-1908, under the name *A. caroliniana* Willd.; Fiori 1943, also under the name *A. caroliniana* Willd.; Lastrucci et al. 2019 [*P. Pellegrini*, 1888, 1920, PI-PELL; *E. Barsali*, 1911, PI; *s. coll.*, 1920, PI]
This species has been recently found in the neighbouring of the study area.

NC **Salvinia natans** (L.) All.
I nat - Eurasiat.
Literature data: Caruel 1870; Baroni 1897-1908

Dennstaedtiaceae

Pteridium aquilinum (L.) Kuhn subsp. **aquilinum**
G rhiz - Cosmop.
Literature data: Caruel 1870; Baroni 1897-1908, Fiori 1943, under the name *Pteris aquilina* L.; Corti 1956, under the names *P. aquilinum* (L.) Kuhn f. *pubescens* Zumagl. subf. *pinnatifidum* Warnst. and f. *glabrum* (Hook) Junge; Corti, 1970; Gellini et al. 1986; Coaro 1987; Garbari 2001; Tomei et al. 2004; Bertacchi et al. 2010; Lombardi 2015
Field observations: L. Pinzani, 2021, 2022; I. Arduini, 2022

Pteridaceae

Adiantum capillus-veneris L.
G rhiz - Pantrop.
Literature data: Fiori 1943
Herbarium data: F. Roma-Marzio, 2014, PI
Field observations: B. Pierini, 2014; L. Pinzani, 2021

Anogramma leptophylla (L.) Link
T caesp - Cosmop.-Subtropic.
Herbarium data: L. Pinzani, 2021, Herb. Pinzani
Field observations: partecipanti all'escursione Wikiplantbase #Parco di Migliarino - San Rossore - Massaciuccoli, 2018; L. Pinzani, 2021

D **Oeosporangium guanchicum** (Bolle) Fraser-Jenk. & Pariyar
Literature data: Ferrarini et al. 1986

Pteris multifida Poir.
Literature data: Galasso et al. 2021 [*L. Pinzani*, 2021, FI]

Aspleniaceae

Asplenium ceterach L. subsp. **ceterach**
H ros - Eurasiat.-Temp.
Literature data: Caruel 1860, under the name *Ceterach officinarum* Willd.
Field observations: L. Pinzani, 2021, 2022; F. Roma-Marzio, 2021

Asplenium onopteris L.
H ros - Subtrop.
Literature data: Fiori 1943, Corti 1956, under the name *A. adiantum-nigrum* L. var. *onopteris* Heufl.; Coaro 1987; Garbari 2001; Lombardi 2015
Field observations: partecipanti all'escursione Wikiplantbase #Parco di Migliarino - San Rossore - Massaciuccoli, 2018

NC **Asplenium ruta-muraria** L. subsp. **ruta-muraria**
H ros - Circumbor.
Literature data: Caruel 1860

NC **Asplenium scolopendrium** L. subsp. **scolopendrium**
H ros - Circumbor.-Temp.
Literature data: Fiori 1943, under the name *Scolopendrium vulgare* Sm.

Asplenium trichomanes L. subsp. **quadrivalens** D.E.Mey.
H ros - Cosmop.-Temp.
Literature data: Caruel 1860, Garbari 2001, under the name *Asplenium trichomanes* L.

Athyriaceae

Athyrium filix-femina (L.) Roth
H ros - Subcosmop.
Literature data: Corti 1956, under the name *A. filix foemina* (L.) Roth. var. *fissidens* (Döll) Milde; Garbari 2001

Thelypteridaceae

Thelypteris palustris Schott
G rhiz - Subcosmop.
Literature data: Caruel 1870, Baroni 1897-1908, under the name *Aspidium thelypteris* Swartz; Fiori 1943, Fiori 1943, under the name *Dryopteris thelypteris* A.Gray; Corti 1956, under the name *Lastrea thelypteris* (L.) Bory; Gellini et al. 1986; Garbari 2001; Tomei et al. 2004
Assessed as VU in the Red List of the Italian Vascular Flora (Rossi et al. 2020)

Dryopteridaceae

NC **Dryopteris dilatata** (Hoffm.) A.Gray
G rhiz - Circumbor.
Literature data: Caruel 1870, under the name *Aspidium spinulosum* Döll; Baroni 1897-1908, under the name *Nephrodium spinulosum* Stremp.; Fiori 1943, Corti 1956, under the name *D. austriaca* Woynar var. *dilatata* Underw.

Dryopteris filix-mas (L.) Schott
G rhiz - Subcosmop.
Literature data: Corti 1956, under the name *D. filix-mas* (L.) Schott. var. *crenata* (Milde) Hayek; Gellini et al. 1986

NC **Polystichum setiferum** (Forssk.) T.Moore ex Woyn.
G rhiz/H ros - Circumbor.
Literature data: Fiori 1943, under the name *Dryopteris aculeata* O.Kuntze var. *setifera* Guadagno; Corti 1956, under the name *P. aculeatum* (L.) Roth subsp. *angulare* (Kit.) Vollm.

Nephrolepidaceae

Nephrolepis cordifolia (L.) C.Presl
G rhiz - Orig. E-Asia-Oceania
Literature data: Bernardello et al. in Marchetti 2003

Polypodiaceae

Polypodium cambricum L.
H ros - Medit.
Literature data: Fiori 1943, Corti 1956, under the name *P. vulgare* L. var. *serratum* W.; Garbari 2001, Lombardi 2015, Bonari et al. 2019, under the name *P. vulgare* L.
Field observation: L. Peruzzi, 2024; I. Arduini, 2024

Pinidae

Ginkgoaceae

Ginkgo biloba L.
Field observations: observed on iNaturalist in 2024
Only cultivated

Cupressaceae

Cupressus sempervirens L.
Field observations: observed on iNaturalist in 2018, 2024
Only cultivated

Juniperus communis L.
P caesp - Circumbor.
Literature data: Corti 1956, under the name *J. communis* L. var. *communis*; Coaro 1987 [*G. Pistolesi, E. Coaro*, 1984, PI]; Garbari 2001
Herbarium data: Corinaldi, 1843, FI; A. Chiarugi, R. Corti, 1951, FI

Juniperus macrocarpa Sm.
P caesp/P scap - Eurimedit.
Literature data: Baroni 1897-1908; Corti 1956, 1970, Bertacchi et al. 2009, Bertacchi et al. 2010, Bertacchi & Lombardi 2014a, under the name *J. oxycedrus* L. subsp. *macrocarpa* (Sibth. et Sm.) Neilreich; Tomei et al. 2004
Herbarium data: Savi, 1826, FI; P. Savi, 1840, FI; s. coll., 1846, PAD; Parlatore, 1892, 1863, FI; s. coll., 1862, FI; S. Sommier, 1876, 1882, 1899, FI; s. coll., 1886, 1892, FI; A. Fiori, 1903; M. Savelli, 1912; A. Chiarugi, R. Corti, 1951, FI
Field observations: L. Pinzani, 2022

NC **Juniperus turbinata** Guss.
P caesp - W-Medit.
Herbarium data: F. Parlatore, 1863, FI; s. coll., 1863, FI

Platycladus orientalis (L.) Franco
Field observations: observed on iNaturalist in 2013, 2023
Only cultivated

Taxodium distichum (L.) Rich.
Herbarium data: F. Roma-Marzio, L. Peruzzi, 2019, PI
Field observations: I. Arduini, 2022
Only cultivated

Pinaceae

Cedrus atlantica (Endl.) G.Manetti ex Carrière
Field observations: observed on iNaturalist in 2022, 2023
Only cultivated
Cedrus deodara (Roxb. ex D.Don) G.Don
Field observations: observed on iNaturalist in 2022, 2024
Only cultivated

NC **Pinus halepensis** Mill. subsp. **halepensis**
P scap - Stenomedit.
Literature data: Baroni 1897-1908

Pinus pinaster Aiton subsp. **pinaster**
P scap - Stenomedit.-Occid.
Literature data: Caruel 1860; Corti 1956; Coaro 1987 [*G. Pistolesi*, *E. Coaro*, 1976, PI]; Tomei et al. 2004; Bertacchi et al. 2009; Dell'Orso & Franchini 2009; Saggese 2016; Bertacchi et al. 2010; Bertacchi & Lombardi 2014a
Field observations: partecipanti all'escursione Wikiplantbase #Parco di Migliarino - San Rossore - Massaciuccoli, 2018

Pinus pinea L.
P scap - Orig. E-Medit.
Literature data: Caruel 1860; Corti 1956; Coaro 1987; Garbari 2001; Pedullà & Garbari, 2004; Tomei et al. 2004; Bertacchi et al. 2010; Arduini & Ercoli 2012; Bertacchi & Lombardi 2014a; Lombardi 2015; Bertacchi & Lombardi 2016; Bonari et al. 2019
Field observations: L. Peruzzi, 2013; 2017; Roberto Dell'Orso, 2015; F. Roma-Marzio, 2019; L. Pinzani, 2020, 2022, 2023; I. Arduini, 2022

Magnoliidae

Nymphaeaceae

NC **Nuphar lutea** (L.) Sm.
I rad - Eurasiat.
Literature data: Caruel 1860

Nymphaea alba L.
Field observations: observed on iNaturalist in 2024
Only cultivated

Aristolochiaceae

Aristolochia clematitis L.
G rad - Submedit.
Literature data: Caruel 1860; Corti 1956; Nardi 1984 [s. *coll*., 1863, FI; *Danielli*, 1876, PI; *Della Nave*, 1881, FI; *Biondi*, s.d., 1886, FI; *N. Passerini*, 1905, PI; *A. Chiarugi*, 1933, FI; *A. Chiarugi*

et *al.*, 1951, FI; *A. Chiarugi, R. Corti*, 1951, FI; *C. Ricceri, Benini*, 1958, FI; *G. Cela*, 1967, FI; *F. Garbari*, 1968, PI]; Garbari 2001; Lazzeri 2021, 2022
Herbarium data: T. Fiaschi, 2018, SIENA; Partecipanti all'escursione Wikiplantbase #Parco di Migliarino - San Rossore - Massaciuccoli, 2018, PI
Field observations: B. Pierini, 2014; partecipanti all'escursione Wikiplantbase #Parco di Migliarino - San Rossore - Massaciuccoli, 2018; L. Peruzzi, 2019; F. Roma-Marzio, 2019; L. Pinzani, 2020, 2021, 2022

Aristolochia rotunda L. subsp. **rotunda**
G bulb - Eurimedit.
Literature data: Caruel 1860; Corti 1956; Nardi 1984 [*P. Savi*, 1860, PI; *P. Savi*, 1861, FI; *T. Caruel*, s.d., FI; *E. Barsali*, 1900, PI; *P. Pellegrini*, 1901, PI; *M. Savelli*, 1913, FI; *A. Chiarugi*, 1933, FI; *A. Chiarugi* et *al.*, 1951, FI; *A. Chiarugi, R. Corti*, 1951, FI; *A. Chiarugi* et al., 1952, FI; *F. Garbari*, 1968, PI; *G. Cela*, 1968, FI; *R.M. Baldini*, 1983, FI; *s. coll.*, s.d., PI]; Gellini et al. 1986; Coaro 1987; Tomei et al. 2004; Lombardi 2015
Herbarium data: F. Picco, 1995, Herb. Picco; M.L. Pedullà, 2000, PI; Partecipanti all'escursione Wikiplantbase #Parco di Migliarino - San Rossore - Massaciuccoli, 2018, PI
Field observations: B. Pierini, 2017; partecipanti all'escursione Wikiplantbase #Parco di Migliarino - San Rossore - Massaciuccoli, 2018; M. D'Antraccoli, 2019; L. Pinzani, 2021; A. Mo, 2022; I. Arduini, 2023

Magnoliaceae

Magnolia grandiflora L.
Field observations: observed on iNaturalist in 2022, 2024
Only cultivated

Lauraceae

Camphora glandulifera (Wall.) Nees
Field observations: observed on iNaturalist in 2022, under the name *Camphora officinarum* auct., non Nees
Only cultivated

Laurus nobilis L.
P caesp - Stenomedit.
Literature data: Caruel 1860; Corti 1956; Gellini et al. 1986; Tomei et al. 2004; Bertacchi et al. 2010; Bertacchi & Lombardi 2014a; Lombardi 2015; Bertacchi & Lombardi 2016
Herbarium data: C. Del Prete, 1979, PI; B. Ciacchi, G. Lorè, 1990, PI
Field observations: L. Peruzzi, 2017; M. D'Antraccoli, 2018; partecipanti all'escursione Wikiplantbase #Parco di Migliarino - San Rossore - Massaciuccoli, 2018; L. Pinzani, 2020, 2022; I. Arduini, 2022

Araceae

Arisarum vulgare O.Targ.Tozz. subsp. **vulgare**
G rhiz - Stenomedit.
Literature data: Pedullà & Garbari, 2004
Herbarium data: L. Pinzani, 2020, Herb. Pinzani

Arum italicum Mill. subsp. **italicum**
G rhiz - Stenomedit.
Literature data: Caruel 1860, under the name *Arisarum italicum* Mill.; Corti 1956; Gellini et al. 1986; Coaro 1987; Garbari 2001; Pedullà & Garbari, 2004; Bertacchi & Lombardi 2016
Herbarium data: F. Picco, 1995, Herb. Picco
Field observations: B. Pierini, 2014; partecipanti all'escursione Wikiplantbase #Parco di Migliarino - San Rossore - Massaciuccoli, 2018; L. Pinzani, 2020, 2021, 2022; I. Arduini, 2023

NC *Dracunculus vulgaris* Schott
Literature data: Caruel 1860

Lemna gibba L.
I nat - Subcosmop.
Literature data: Caruel 1860; Corti 1956; Garbari 2001; Pedullà & Garbari, 2004
Field observations: partecipanti all'escursione Wikiplantbase #Parco di Migliarino - San Rossore - Massaciuccoli, 2018

Lemna minor L.
I nat - Subcosmop.
Literature data: Caruel 1860; Corti 1956; Coaro 1987
Field observations: partecipanti all'escursione Wikiplantbase #Parco di Migliarino - San Rossore - Massaciuccoli, 2018

Lemna minuta Kunth
I nat - Orig. America
Herbarium data: L. Pinzani, A. Giacò, J. Franzoni, 2021, Herb. Pinzani

Spirodela polyrhiza (L.) Schleid.
I nat - Subcosmop.
Literature data: Pedullà & Garbari, 2004
Herbarium data: M. D'Antraccoli, 2017, PI

NC **Wolffia arrhiza** (L.) Horkel ex Wimm.
I nat - Paleosubtrop.
Literature data: Caruel 1860, under the name *Lemna arrhiza* L.

Alismataceae

Alisma lanceolatum With.
I rad - Subcosmop.
Literature data: Garbari 2001

Alisma plantago-aquatica L.
I rad - Subcosmop.
Literature data: Caruel 1860; Corti 1956, under the name *A. plantago-aquatica* L. subsp. *michaletii* Asch. u. Gr. var. *stenophyllum* Asch. u. Gr.; Gellini et al. 1986; Coaro 1987; Pedullà & Garbari, 2004
Herbarium data: L. Pinzani, 2020, Herb. Pinzani

Field observations: partecipanti all'escursione Wikiplantbase #Parco di Migliarino - San Rossore - Massaciuccoli, 2018; I. Arduini, 2024

Baldellia ranunculoides (L.) Parl.
I rad - Medit.-Atl.
Literature data: Caruel 1860, Baroni 1897-1908, under the name *Alisma ranunculoides* L.; Corti 1956 [*Picciuoli*, FI]; Corti 1956, under the name *Echinodorus ranunculoides* (L.) Engelm. in Aschers.; Gellini et al. 1986; Coaro 1987; Garbari 2001; Tomei et al. 2004
Assessed as EN in the Red List of the Italian Vascular Flora (Rossi et al. 2020)

NC **Damasonium alisma** Mill.
I rad - Eurimedit.
Literature data: Caruel 1860, under the name *D. stellatum* Pers.

Sagittaria sagittifolia L.
I rad - Eurasiat.
Literature data: Caruel 1860; Pedullà & Garbari, 2002 [*M.L. Pedullà*, 2000, PI]; Pedullà & Garbari, 2004
Assessed as EN in the Red List of the Italian Vascular Flora (Rossi et al. 2013)

Butomaceae

Butomus umbellatus L.
I rad - Eurasiat.
Literature data: Caruel 1860; Garbari 2001; Pedullà & Garbari, 2004
Assessed as VU in the Red List of the Italian Vascular Flora (Rossi et al. 2020)

Hydrocharitaceae

NC *Elodea canadensis* Michx.
Literature data: Savelli 1915, under the name *Anacharis canadensis* (Michx.) Planch.; Montelucci 1962

NC **Najas major** All.
I rad - Cosmop.
Literature data: Caruel 1860

NC **Najas minor** All.
I rad - Paleotemp.-Subtrop.
Literature data: Caruel 1860

Juncaginaceae

Triglochin barrelieri Loisel.
G bulb - Stenomedit.
Literature data: Coaro 1987, Petraglia 2013, under the name *T. bulbosum* L. subsp. *barrelieri* (Loisel.) Rouy
Herbarium data: L. Pinzani, A. Giacò, J. Franzoni, 2021, Herb. Pinzani
Assessed as EN in the Red List of the Italian Vascular Flora (Rossi et al. 2020)

NC **Triglochin maritima** L.
H scap - Subcosmop.
Literature data: Caruel 1860

Potamogetonaceae

NC **Potamogeton coloratus** Hornem.
I rad - Subtrop.
Literature data: Baroni 1987-1908

NC **Potamogeton crispus** L.
I rad - Subcosmop.
Literature data: Caruel 1860

NC **Potamogeton lucens** L.
I rad - Circumbor.
Literature data: Caruel 1860

Potamogeton natans L.
I rad - Subcosmop.
Literature data: Caruel 1860; Corti 1956, under the name *P. natans* L. var. *prolixus* Koch; Garbari 2001

Potamogeton nodosus Poir.
I rad - Subcosmop.
Literature data: Lastrucci et al. 2010 [*M. Savelli*, 1916, FI; *M. Tani*, 1926, FI; *A. Chiarugi, R. Corti*, 1951, FI]
Field observations: L. Peruzzi, 2023

NC **Potamogeton perfoliatus** L.
I rad - Subcosmop.
Literature data: Caruel 1860

Stuckenia pectinata (L.) Börner
I rad - Subcosmop.
Literature data: Sani & Tomei 2006, Bertacchi et al. 2007, under the name *Potamogetum pectinatus* L.

Zannichellia palustris L.
I rad - Cosmop.
Literature data: Caruel 1860; Coaro 1987
Field observations: L. Pinzani, 2021
Assessed as NT in the Red List of the Italian Vascular Flora (Rossi et al. 2020)

Ruppiaceae

Ruppia maritima L.
I rad/I nat - Cosmop.

Literature data: Garbari 2001; Bertacchi et al. 2007
Field observations: L. Pinzani, 2021

Dioscoreaceae

Dioscorea communis (L.) Caddick & Wilkin
G rad - Eurimedit.
Literature data: Bertacchi et al. 2009; Bertacchi & Lombardi 2016; Lazzeri 2021

Colchicaceae

Colchicum lusitanum Brot.
G bulb - W-Medit.-Mont.
Literature data: D’Amato 1955 [*F. D'Amato*, 1952, PI; *F. D'Amato*, 1954, PI]; Corti 1956
Field observations: observed on iNaturalist in 2021, under the name *Colchicum longifolium* Castagne

Colchicum neapolitanum (Ten.) Ten. subsp. **neapolitanum**
G bulb - Endem. Ital.
Literature data: Caruel 1860, under the name *Colchicum autumnale* L.; D’Amato 1955 [*F. D'Amato*, 1949, PI]; Corti 1956; D’Amato 1957 [*F. D'Amato*, 1956, PI]
Field observations: observed on iNaturalist in 2024, under the name *Colchicum autumnale* L.

Smilacaceae

Smilax aspera L.
G rhiz - Eurimedit.
Literature data: Corti 1956 [*S. Sommier*, s.d., FI]; Coaro 1987 [*G. Pistolesi*, *E. Coaro*, 1977, 1984, PI; *E. Coaro*, 1984, PI]; Tomei et al. 2004; Bertacchi et al. 2009; Bertacchi et al. 2010; Arduini & Ercoli 2012; Bertacchi & Lombardi 2014a; Lombardi 2015; Bertacchi & Lombardi 2016; Bonari et al. 2019
Herbarium data: A. Preta, 1979, PI; R. Poli, 1989, PI; T. Fiaschi, 2018, SIENA
Field observations: R. Dell’Orso, 2014; partecipanti all'escursione Wikiplantbase #Parco di Migliarino - San Rossore - Massaciuccoli, 2018; L. Pinzani, 2020, 2021, 2022; A. Mo, 2022

Liliaceae

NC **Lilium bulbiferum** L. subsp. **croceum** (Chaix) Jan
G bulb - Orof. Centroeurop.
Literature data: Caruel 1860, under the name *L. bulbiferum* L.; Corti 1956, under the name *L. bulbiferum* L. var. *croceum* (Chaix) Ducomm

Lilium candidum L.
Field observations: observed on iNaturalist in 2022
Only cultivated

NC *Tulipa clusiana* Redouté
Literature data: Caruel 1860

NC **Tulipa sylvestris** L.
G bulb - Eurimedit.
Literature data: Baroni 1897-1908

Orchidaceae

NC **Anacamptis coriophora** (L.) R.M.Bateman, Pridgeon & M.W.Chase
G bulb - Eurimedit.
Literature data: Caruel 1860, under the name *Orchis coriophora* L.; Corti, 1951, 1956, under the name *Orchis coriophora* L. var. *fragrans* Boiss.; Corti 1956 [*P. Savi*, s.d., FI]

Anacamptis laxiflora (Lam.) R.M.Bateman, Pridgeon & M.W.Chase
G bulb - Eurimedit.
Literature data: Caruel 1860, Garbari 2001, under the name *Orchis laxiflora* Lam.; Corti 1956, under the name *Orchis laxiflora* Lam. var. *laxiflora*; Peruzzi et al. 2017a [*F. Roma-Marzio, M. D'Antraccoli*, 2016, PI]

Anacamptis morio (L.) R.M.Bateman, Pridgeon & M.W.Chase
G bulb - Europ.-Caucas.
Literature data: Caruel 1860, Corti 1956, Coaro 1987, Garbari 2001, under the name *Orchis morio* L.
Field observations: L. Peruzzi, 2010; R. Dell'Orso, 2020; L. Pinzani, 2021

Anacamptis palustris (Jacq.) R.M.Bateman, Pridgeon & M.W.Chase
G bulb - Eurimedit.
Literature data: Baroni 1897-1908, under the name *Orchis laxiflora* Lam. var. *palustris* Jacq.; Del Prete & Tomei 1981 [*T. Caruel*, 1856, PI; *P. Sav*i, 1858, FI; s. coll., 1861, PI; *Savelli*, 1876, FI; *G. Arcangeli*, 1882, PI; *Fantozzi*, 1891, FI; *Barsali*, 1920, PI]; Dell'Orso & Franchini 2009
Assessed as EN in the Red List of the Italian Vascular Flora (Rossi et al. 2013)

Anacamptis papilionacea (L.) R.M.Bateman, Pridgeon & M.W.Chase
G bulb - Eurimedit.
Literature data: Corti 1956, under the name *Orchis papilionacea* L. var. *parviflora* Willk. in Willk. et Lange; Garbari 2001, under the name *Orchis papilionacea* L.

Barlia robertiana (Loisel.) Greuter
G bulb - Stenomedit.
Field observations: R. Dell'Orso, 2020
Assessed as LC in the Red List of the Italian Vascular Flora (Rossi et al. 2020)

NC **Cephalanthera longifolia** (L.) Fritsch
G rhiz - Eurasiat.
Literature data: Caruel 1860, under the name *C. ensifolia* Rich.; Corti 1956 [*P. Fantozzi*, FI]; Corti 1956; Del Prete 1978 [*Danielli*, 1877, PI; *E. Barsali*, 1921, PI; *A. Chiarugi, R. Corti*, 1951, FI]

Cephalanthera rubra (L.) Rich.
G rhiz - Eurasiat.
Literature data: Garbari 2001

Epipactis microphylla (Ehrh.) Sw. subsp. **microphylla**
G rhiz - Europ.-Caucas.
Literature data: Corti 1956; Welti et al. 2011

Epipactis palustris (L.) Crantz
G rhiz - Circumbor.
Literature data: Baroni 1897-1908; Corti 1956 [*Poggi, Rossetti*, s.d., FI]; Del Prete & Tomei 1981 [*s. coll.*, 1862, PI; *Poggi, Rossetti*, 1887, FI; *E. Barsali*, 1923, PI]; Dell'Orso & Franchini 2009; Welti et al. 2011; Saggese 2016
Field observations: R. Righini, 2021
Assessed as NT in the Red List of the Italian Vascular Flora (Rossi et al. 2013)

Limodorum abortivum (L.) Sw.
G rhiz - Eurimedit.
Literature data: Corti 1956; Garbari 2001
Field observations: L. Pinzani, 2021

NC **Neotinea maculata** (Desf.) Stearn
G bulb - Stenomedit.
Literature data: Caruel 1860, under the name *Himantoglossum secundiflorum* Reich.; Corti 1956, under the name *N. intacta* (Lk) Rchb.f.

Ophrys apifera Huds.
G bulb - Eurimedit.
Literature data: Coaro 1987
Field observations: L. Peruzzi, 2010; A. Mirabella, R. Dell'Orso, 2022.

NC **Ophrys funerea** Viv.
G bulb - Stenomedit.
Literature data: Caruel 1860, under the name *O. fusca* Link

Ophrys sphegodes Mill. subsp**. maritima** (Pacifico & Soca) Kreutz
G bulb - Endem. Ital.
Field observations: A. Mirabella, R. Dell'Orso, 2022
Assessed as LC in the Red List of the Italian Vascular Flora (Rossi et al. 2020)

Ophrys sphegodes Mill. subsp. **classica** (Devillers-Tersch. & Devillers) Kreutz
G bulb - Endem. Ital.
Literature data: Caruel 1860, under the name *O. aranifera* Huds.; Corti, 1954, 1956, *O. aranifera* Huds. subsp. *euaranifera* Hayek var. *genuina* Rchb.; Corti 1956 [*M. Savelli*, s.d., FI]; Coaro 1987, under the name *O. sphecodes* Mill. subsp. *sphecodes*
Field observations: R. Dell'Orso, 2020, 2024; L. Pinzani, 2020
Assessed as LC in the Red List of the Italian Vascular Flora (Rossi et al. 2020)

NC **Orchis purpurea** Huds.
G bulb - Eurasiat.
Literature data: Caruel 1860

Platanthera chlorantha (Custer) Rchb.

G bulb - Eurosiber.
Literature data: Corti, 1951, 1954, 1956; Coaro 1987; Garbari 2001

NC **Serapias cordigera** L. subsp. **cordigera**
G bulb - Stenomedit.
Literature data: Caruel 1860

Serapias lingua L.
G bulb - Stenomedit.-Occid.
Field observations: M. Martinelli, 2022; A. Mirabella, R. Dell’Orso, 2022
Assessed as LC in the Red List of the Italian Vascular Flora (Rossi et al. 2020)

Serapias neglecta De Not.
G bulb – N-Medit.
Literature data: Caruel 1860; Corti 1956, under the name *S. cordigera* L. var. *neglecta* (De Not.) Fiori in Fiori e Paol.
Field observations: M. Martinelli, 2022; L. Pinzani, 2021, 2022; R. Dell'Orso, 2023, 2024
Assessed as LC in the Red List of the Italian Vascular Flora (Rossi et al. 2020)

Serapias parviflora Parl.
G bulb - Stenomedit.
Field observations: R. Dell'Orso, 2020; A. Mirabella, R. Dell’Orso, 2022

Serapias vomeracea (Burm.f.) Briq.
G bulb - Stenomedit.
Field observations: observed on iNaturalist in 2020, 2022, 2023, 2024

Ex **Spiranthes aestivalis** (Poir.) Rich.
G rhiz - Medit.-Atl.
Literature data: Caruel 1860; Del Prete & Tomei 1981 [*T. Caruel*, 1858, PI; *N. Passerini*, 1927, PI]

Spiranthes spiralis (L.) Chevall.
G rhiz - Europ.-Caucas.
Literature data: Caruel 1860, under the name *S. autumnalis* Rich.; Baroni, 1897-1980.
Field observations: J. Franzoni, 2018; L. Pinzani, 2021; R. Dell’Orso, 2021, 2022, 2024; A. Mirabella, 2022; L. Peruzzi, 2023

Iridaceae

Chamaeiris foetidissima (L.) Medik.
G rhiz - Eurimedit.
Literature data: Caruel 1860, Corti, 1954, 1956, 1970, Corti 1956 [*M. Savelli*, FI; *A. Fiori*, FI], Gellini et al. 1986, Coaro 1987, Garbari 2001, Pedullà & Garbari, 2004, Tomei et al. 2004, Lombardi 2015, Bonari et al. 2019, under the name *Iris foetidissima* L.
Field observations: partecipanti all'escursione Wikiplantbase #Parco di Migliarino - San Rossore - Massaciuccoli, 2018; I. Arduini, 2024

Chamaeiris orientalis (Mill.) M.B.Crespo

Field observations: observed on iNaturalist in 2024, under the name *Iris orientalis* Mill.

Crocus biflorus Mill.
G bulb – Endem. Ital.
Literature data: Caruel 1860
Field observations: L. Pinzani, 2022
Assessed as LC in the Red List of the Italian Vascular Flora (Rossi et al. 2020)

Freesia leichtlinii Klatt subsp. *alba* (G.L.Mey.) J.C.Manning & Goldblatt
Herbarium data: L. Pinzani, 2022, Herb. Pinzani

Gladiolus italicus Mill.
G bulb - Eurimedit.
Literature data: Caruel 1860, under the name *G. segetum* Gawl.; Pedullà & Garbari, 2004, under the name *G. communis* L.
Field observations: B. Pierini, 2016; A. Mo, 2023

Iris florentina L.
Literature data: Peruzzi et al. 2017b [*F. Roma-Marzio, M. D'Antraccoli*, 2017, PI], under the name *Iris albicans* Lange

Limniris pseudacorus (L.) Fuss
G rhiz - Eurasiat.
Literature data: Caruel 1860, Corti 1956 [*S. Sommier*, s.d., FI]; Corti 1956, Tomei et al. 2004, under the name *Iris pseudacorus* L.; Gellini et al. 1986; Bertacchi & Lombardi 2016
Field observations: F. Roma-Marzio, 2014; B. Pierini, 2015, 2020; partecipanti all'escursione Wikiplantbase #Parco di Migliarino - San Rossore - Massaciuccoli, 2018; L. Pinzani, 2021, 2022; I. Arduini, 2024

Romulea bulbocodium (L.) Sebast. & Mauri
G bulb - Stenomedit.
Literature data: Caruel 1860; Corti 1956 [*P. Fantozzi*, FI]
Herbarium data: L. Pinzani, 2020, Herb. Pinzani

Romulea columnae Sebast. & Mauri
G bulb - Stenomedit.
Literature data: Caruel 1860; Garbari 2001
Field observations: J. Franzoni, 2022 (growing spontaneously inside the Botanic Garden of Pisa)

Romulea ramiflora Ten. subsp. **ramiflora**
G bulb - Stenomedit.-Macarones.
Literature data: Caruel 1860
Field observations: L. Peruzzi, 2016
Assessed as LC in the Red List of the Italian Vascular Flora (Rossi et al. 2020)

NC **Romulea rollii** Parl.
G bulb - Stenomedit.-Occid.
Literature data: Baroni 1897-1908

D *Romulea rosea* (L.) Eckl.
Literature data: Garbari 2001, under the name *Romulea purpurascens* Ten.

Sisyrinchium micranthum Cav.
Field observations: observed on iNaturalist in 2023, 2024

Asphodelaceae

Asphodelus fistulosus L.
H bienn/H scap - Subtrop/Paleosubtrop.
Literature data: Caruel 1860
Field observations: V. Lazzeri, 2017; B. Pierini, 2017; J. Franzoni, 2019; F. Roma-Marzio, 2019, 2021; L. Pinzani, 2022

NC **Asphodelus ramosus** L. subsp. **ramosus**
G rhiz - Stenomedit.
Literature data: Caruel 1860, under the name *A. microcarpus* Viv.

Amaryllidaceae

NC **Allium chamaemoly** L. subsp. **chamaemoly**
G bulb - Stenomedit.-Occid.
Literature data: Caruel 1860

Allium coloratum Spreng.
G bulb - Medit.-Mont.
Literature data: Caruel 1860, under the name *A. pulchellum* Don; Coaro 1987, under the name *A. cirrhosum* Vandelli
Field observations: B. Pierini, 2014; I. Arduini, 2024

Allium longispathum Redouté
G bulb - Eurimedit.
Literature data: Coaro 1987, under the name *A. paniculatum* L.

Allium neapolitanum Cirillo
G bulb - Stenomedit.
Literature data: Caruel 1860
Herbarium data: L. Pinzani, 2019, FI
Field observations: B. Pierini, 2014; F. Roma-Marzio, 2014; L. Peruzzi, 2016, 2017; L. Pinzani, 2019, 2021, 2022; F. Roma-Marzio, 2021
Assessed as LC in the Red List of the Italian Vascular Flora (Rossi et al. 2020)

NC **Allium nigrum** L.
G bulb - Stenomedit.
Literature data: Caruel 1860

NC **Allium pallens** L.
G bulb - Stenomedit.
Literature data: Caruel 1860

Allium polyanthum Schult. & Schult.f.
G bulb - Eurimedit.
Literature data: Caruel 1860, under the name *A. ampeloprasum* Linn.
Field observation: observed on iNaturalist in 2022a, 2022b, 2022c, 2023, 2024

Allium porrum L.
Literature data: Tomei & Camangi 2014

Allium roseum L. subsp. **roseum**
G bulb - Stenomedit.
Literature data: Garbari 2001
Field observations: L. Peruzzi, 2018; R. Dell'Orso, 2015; L. Pinzani, 2021
Assessed as LC in the Red List of the Italian Vascular Flora (Rossi et al. 2020)

Allium savii Parl.
G bulb - S-Medit.
Literature data: Caruel 1860, under the name *A. fuscum* Waldst. & Kit.; Coaro 1987, under the name *A. fuscum* W. et K.; Garbari 2001
Herbarium data: L. Pinzani, 2020, Herb. Pinzani
This species was described by Parlatore (1857) on material collected by Pietro Savi from Tombolo (Peruzzi et al. 2019).
Assessed as NT in the Red List of the Italian Vascular Flora (Rossi et al. 2020)

Allium sphaerocephalon L. subsp. **sphaerocephalon**
G bulb - Paleotemp.
Field observations: partecipanti all'escursione Wikiplantbase #Parco di Migliarino - San Rossore - Massaciuccoli, 2018

Allium triquetrum L.
G bulb - Stenomedit.-Occid.
Literature data: Coaro 1987
Herbarium data: L. Pinzani, 2020, Herb. Pinzani
Field observations: B. Pierini, 2014; L. Pinzani, 2021, 2022; F. Roma-Marzio, 2021
Assessed as LC in the Red List of the Italian Vascular Flora (Rossi et al. 2020)

Allium vineale L.
G bulb - Eurimedit.
Literature data: Caruel 1860; Corti 1956, under the name *A. vineale* L. var. *compactum* (Thuill.) Asch. forma *fuscencens* Asch. u. Gr.; Garbari 2001; Lombardi 2015; Saggese 2016
Field observations: partecipanti all'escursione Wikiplantbase #Parco di Migliarino - San Rossore - Massaciuccoli, 2018

Ipheion uniflorum (Lindl.) Raf.
G bulb - Orig. S-America
Field observations: J. Franzoni, 2020

Leucojum aestivum L. subsp. **aestivum**
G bulb - Europ.-Caucas.

Literature data: Caruel 1860; Corti 1956; Garbari 2001
Assessed as VU in the Red List of the Italian Vascular Flora (Rossi et al. 2020)

Narcissus poëticus L.
G bulb - Orof. S-Europ.
Literature data: Caruel 1860; Corti 1956, under the name *N. pöeticus* L. subsp. *angustifolius* (Curtis) Schinz u. Keller; Corti, 1970; Garbari 2001, under the name *N. radiiflorus* Salisb.

Narcissus tazetta L. subsp. italicus (Ker Gawl.) Baker
G bulb - Steno-Medit.
Field observation: observed on iNaturalist in 2020

Narcissus tazetta L. subsp. **tazetta**
G bulb - Stenomedit.
Literature data: Caruel 1860; Garbari 2001
Field observations: B. Pierini, 2021

Nothoscordum gracile (Aiton) Stearn
G bulb - Orig. CS-America
Literature data: Peruzzi et al. 2019 [*M. D'Antraccoli*, 2018, PI]
Herbarium data: L. Pinzani, 2021, Herb. Pinzani
Field observations: H. Öhm, 2024

Pancratium maritimum L.
G bulb - Stenomedit.
Literature data: Caruel 1860; Baroni 1897-1908; Corti 1956; Garbari 2001; Sani & Tomei 2006; Bertacchi et al. 2009; Bertacchi et al. 2010; Bertacchi & Lombardi 2014a; Bertacchi & Lombardi 2014b
Herbarium data: D. Ciccarelli & M. Sammartino, 2009, PI
Field observations: B. Pierini, 2014; partecipanti all'escursione Wikiplantbase #Parco di Migliarino - San Rossore - Massaciuccoli, 2018; L. Pinzani, 2022

Tulbaghia violacea Harv.
Field observations: observed on iNaturalist in 2022
Only cultivated

Asparagaceae

Agave americana L. subsp. *americana*
Field observations: observed on iNaturalist in 2023
Only cultivated

Asparagus acutifolius L.
G rhiz - Stenomedit.
Literature data: Corti, 1951, 1954, 1956; Corti 1956 [*M. Savelli*, FI]; Coaro 1987 [*G. Pistolesi*, *E. Coaro*, 1976, PI; E. *Coaro*, 1985, PI]; Garbari 2001; Tomei et al. 2004; Bertacchi et al. 2010; Arduini & Ercoli 2012; Bertacchi & Lombardi 2014a; Lombardi 2015; Bonari et al. 2019
Herbarium data: R. Poli, 1989, PI; L. Pinzani, 2020, Herb. Pinzani

Field observations: partecipanti all'escursione Wikiplantbase #Parco di Migliarino - San Rossore - Massaciuccoli, 2018; L. Pinzani, 2020, 2021, 2022; A. Mo, 2022

Asparagus aethiopicus L.
Field observations: observed on iNaturalist in 2024
Only cultivated

Asparagus officinalis L. subsp. **officinalis**
G rhiz - Eurimedit.
Literature data: Corti 1956
Herbarium data: L. Pinzani, 2020, Herb. Pinzani

Asparagus tenuifolius Lam.
G rhiz - SE-Europ.
Literature data: Caruel 1860; Corti 1956; Garbari 2001

Bellevalia romana (L.) Sweet
G bulb - Centromedit.
Literature data: Caruel 1860; Corti 1956 [*A. Vaccari*, s.d., FI]; Corti 1956; Garbari 2001; Pedullà & Garbari, 2004
Field observations: B. Pierini, 2017; J. Franzoni, 2020; L. Pinzani, 2021, 2022; A. Mo, 2022; J. Franzoni, 2023
Assessed as LC in the Red List of the Italian Vascular Flora (Rossi et al. 2020)

NP *Bellevalia webbiana* Parl.
Literature data: Caruel 1860
According to Gestri et al. (2010), this species was wrongly reported for the study area.

Loncomelos narbonense (L.) Raf.
G bulb - Eurimedit.
Literature data: Saggese 2016

Loncomelos pyrenaicum (L.) L.D.Hrouda subsp. **pyrenaicum**
G bulb - Eurimedit.
Literature data: Caruel 1860, Garbari 2001, under the name *Ornithogalum pyrenaicum* L.; Corti 1956, under the name *Ornithogalum pyrenaicum* L. var. *flavescens* (Lam.) Baker

NC **Muscari botryoides** (L.) Mill. subsp. **botryoides**
G bulb - Submedit.
Literature data: Caruel 1860, under the name *Botryanthus vulgaris* Kunth

Muscari comosum (L.) Mill.
G bulb - Eurimedit.
Literature data: Caruel 1860, under the name *Bellevalia comosa* Kunth; Corti 1956, under the name *M. comosum* (L.) Mill. var. *typicum* Fiori in Fiori e Paol.; Coaro 1987, Garbari 2001, under the name *Leopoldia comosa* (L.) Parl.
Field observations: B. Pierini, 2017; L. Pinzani, 2022.

Muscari neglectum Guss. ex Ten. & Sangiovanni

G bulb - Eurimedit.
Literature data: Coaro 1987
Herbarium data: L. Pinzani, 2020, Herb. Pinzani
Field observations: L. Pinzani, 2021, 2022

Ornithogalum divergens Boreau
G bulb - S-Europ.
Literature data: Caruel 1860, under the name *O. umbellatum* L.; Pedullà & Garbari, 2004
Herbarium data: F. Roma-Marzio, 2013, PI
Field observations: B. Pierini, 2014; L. Peruzzi, 2016

Ornithogalum exscapum Ten.
G bulb - Endem. Ital.
Literature data: Garbari 2001
Herbarium data: L. Pinzani, 2020, Herb. Pinzani
Field observations: B. Pierini, 2016, 2018, 2021
Assessed as LC in the Red List of the Italian Vascular Flora (Rossi et al. 2020)

Polygonatum odoratum (Mill.) Druce
G rhiz - Circumbor.
Literature data: Caruel 1860, under the name *P. officinale* All.; Corti 1956 [*P. Savi*, s.d., FI]; Gellini et al. 1986; Tomei et al. 2004

Prospero autumnale (L.) Speta
G bulb - Eurimedit.
Literature data: Caruel 1860, Corti 1956 [*M. Savelli*, s.d., FI], Garbari 2001, under the name *Scilla autumnalis* L.; Lombardi 2015
Herbarium data: F. Picco, 1995, Herb. Picco; J. Franzoni, 2019, Herb. Franzoni
Field observations: J. Franzoni, 2018

Ruscus aculeatus L.
G rhiz - Eurimedit.
Literature data: Caruel 1860; Corti, 1951, 1954, 1956, 1970; Gellini et al. 1986; Coaro 1987; Tomei et al. 2004; Lombardi 2015; Bertacchi & Lombardi 2016; Bonari et al. 2019
Field observations: R. Dell'Orso, 2015; L. Peruzzi, 2017; partecipanti all'escursione Wikiplantbase #Parco di Migliarino - San Rossore - Massaciuccoli, 2018; L. Pinzani, 2020, 2022; I. Arduini, 2023
Assessed as LC in the Red List of the Italian Vascular Flora (Rossi et al. 2013)

NC **Squilla maritima** (L.) Steinh.
G bulb - Stenomedit.
Literature data: Caruel 1860, under the name *Urginea scilla* Steinh.

Yucca gloriosa L.
P caesp - Orig. N-America
Literature data: Garbari 2001; Tomei et al. 2004; Sani & Tomei 2006; Bertacchi et al. 2009; Bertacchi et al. 2010; Bertacchi & Lombardi 2014a
Field observations: I. Arduini, 2024

Arecaceae

Chamaerops humilis L. subsp. *humilis*
Field observations: F. Roma-Marzio, 2021

Commelinaceae

Tradescantia fluminensis Vell.
G rhiz - Orig. S-America
Field observations: L. Pinzani, 2020

Strelitziaceae

Strelitzia reginae Banks
Field observations: observed on iNaturalist in 2022
Only cultivated

Typhaceae

NC **Sparganium emersum** Rehmann
I rad - Eurasiat.
Literature data: Caruel 1860, under the name *S. simplex* Huds. var. *fluitans* Gr. et Godr.

Sparganium erectum L.
I rad - Eurasiat.
Literature data: Caruel 1860, under the name *S. ramosum* Huds.; Coaro 1987; Pedullà & Garbari, 2004; Bertacchi et al. 2007

Typha angustifolia L.
G rhiz - Circumbor.
Literature data: Caruel 1860; Coaro 1987; Petraglia 2013

Typha latifolia L.
G rhiz - Cosmop.
Literature data: Caruel 1860; Corti 1956, 1970; Coaro 1987; Garbari 2001; Lazzeri in Buono et al. 2022

NC **Typha minima** Funk ex Hoppe
G rhiz - Eurasiat.
Literature data: Caruel 1860

Juncaceae

Juncus acutiflorus Ehrh. ex Hoffm.
G rhiz - Europ.
Literature data: Caruel 1860, under the name *J. sylvaticus* Reich.; Coaro 1987

Juncus acutus L. subsp. **acutus**
H caesp - Eurimedit.

Literature data: Mainardi 1982; Coaro 1987; Garbari 2001; Tomei et al. 2004; Sani & Tomei 2006 Bertacchi et al. 2007; Bertacchi et al. 2009; Dell'Orso & Franchini 2009; Arduini & Ercoli 2012; Petraglia 2013; Bertacchi & Lombardi 2014b; Saggese 2016; Lazzeri 2021, 2022
Herbarium data: Partecipanti all'escursione Wikiplantbase #Parco di Migliarino - San Rossore - Massaciuccoli, 2018, PI
Field observations: partecipanti all'escursione Wikiplantbase #Parco di Migliarino - San Rossore - Massaciuccoli, 2018

NC **Juncus anceps** Laharpe
G rhiz - W-Medit.-Atl.
Literature data: Caruel 1860; Corti 1956 [*Roberti*, FI]

Juncus articulatus L. subsp. **articulatus**
G rhiz - Circumbor.
Literature data: Caruel 1860, under the name *J. lamprocarpus* Ehrh.; Corti 1956, under the name *J. articulatus* L. varieties excluded; Gellini et al. 1986; Coaro 1987; Garbari 2001; Pedullà & Garbari, 2004; Arduini & Ercoli 2012; Petraglia 2013; Lombardi 2015; Saggese 2016; Lazzeri in Buono et al. 2022
Herbarium data: F. Roma-Marzio, M. D'Antraccoli, 2015, PI
Field observations: partecipanti all'escursione Wikiplantbase #Parco di Migliarino - San Rossore - Massaciuccoli, 2018

Juncus bufonius L.
T caesp - Cosmop.
Literature data: Caruel 1860; Corti 1956, under the name *J. bufonius* var. *genuinus* Coutinho; Garbari 2001; Arduini & Ercoli 2012; Lombardi 2015; Lazzeri in Buono et al. 2022
Herbarium data: A. Sani, 2005, PI; Partecipanti all'escursione Wikiplantbase #Parco di Migliarino - San Rossore - Massaciuccoli, 2018, PI
Field observations: partecipanti all'escursione Wikiplantbase #Parco di Migliarino - San Rossore - Massaciuccoli, 2018; L. Pinzani, 2021; I. Arduini, 2024

Juncus bulbosus L.
I rad - Europ.
Literature data: Garbari 2001 [*B. Ciacchi, G. Lorè*, 1997, PI]; Lastrucci et al. 2008 [A. *Tassi*, 1843, SIENA; *A. Chiarugi, R. Corti*, 1951, FI; *A. Chiarugi, R. Corti, G. Negri*, 1951, FI; *R. Corti, E. Francini, A. Messeri*, 1951, FI; *A. Chiarugi, R. Corti, R. Corradi*, 1952, FI; *B. Ciacchi, G. Lorè*, 1997, PI]

NC **Juncus capitatus** Weigel
T scap - Eurimedit.-Atl.
Literature data: Caruel 1860

Juncus compressus Jacq.
G rhiz - Eurasiat.
Literature data: Caruel 1860; Coaro 1987; Lazzeri in Buono et al. 2022
Herbarium data: Partecipanti all'escursione Wikiplantbase #Parco di Migliarino - San Rossore - Massaciuccoli, 2018, PI

Juncus conglomeratus L.

H caesp - Eurosiber.
Literature data: Caruel 1860; Corti 1956, under the name *J. conglomeratus* L. var. *typicus* Asch. u. Gr.; Gellini et al. 1986; Tomei et al. 2004; Bertacchi et al. 2007; Bertacchi et al. 2009; Arduini & Ercoli 2012; Lombardi 2015

Juncus effusus L. subsp. **effusus**
H caesp - Cosmop.
Literature data: Caruel 1860; Corti 1956, under the name *J. effusus* L. var. *elatus* Asch. u. Gr.; Gellini et al. 1986; Coaro 1987; Tomei et al. 2004; Sani & Tomei 2006; Arduini & Ercoli 2012; Petraglia 2013; Lombardi 2015; Lazzeri in Buono et al. 2022
Field observations: B. Pierini, 2017; partecipanti all'escursione Wikiplantbase #Parco di Migliarino - San Rossore - Massaciuccoli, 2018; I. Arduini, 2024

Juncus gerardi Loisel. subsp. **gerardi**
G rhiz - Circumbor.
Literature data: Sani & Tomei 2005 [*A. Sani, P.E. Tomei*, 2005, FI]; Sani & Tomei 2006 [*A. Sani*, 2005, PI]; Bertacchi et al. 2007; Petraglia 2013; Saggese 2016; Lazzeri 2021, 2022

NC **Juncus heterophyllus** Dufour
I rad - W-Medit.-Atl.
Literature data: Caruel 1860

Juncus hybridus Brot.
T caesp - Medit.-Atl.
Literature data: Coaro 1987; Bertacchi et al. 2009; Lazzeri in Buono et al. 2022
Herbarium data: Partecipanti all'escursione Wikiplantbase #Parco di Migliarino - San Rossore - Massaciuccoli, 2018, PI

Juncus inflexus L. subsp. **inflexus**
H caesp - Paleotemp.
Literature data: Corti 1956, under the name *J. inflexus* L. var. *longicornis* (Bastard) Fiori; Gellini et al. 1986; Garbari 2001; Pedullà & Garbari, 2004; Tomei et al. 2004; Arduini & Ercoli 2012; Petraglia 2013; Lazzeri in Buono et al. 2022
Herbarium data: A. Sani, 2005, PI
Field observations: B. Pierini, 2017; I. Arduini, 2023

Juncus littoralis C.A.Mey.
H caesp - Medit.-Tura.
Literature data: Caruel 1860, under the name *J. tommasinii* Parl.; Corti 1956 [*P. Savi*, s.d., FI; *O. Beccari*, s.d., FI]; Corti 1956, under the name *J. acutus* L. var. *tommasinii* (Parl.) Arcangeli; Bertacchi et al. 2009
Herbarium data: Partecipanti all'escursione Wikiplantbase #Parco di Migliarino - San Rossore - Massaciuccoli, 2018, PI
Field observations: partecipanti all'escursione Wikiplantbase #Parco di Migliarino - San Rossore - Massaciuccoli, 2018

Juncus maritimus Lam.
G rhiz - Subcosmop.

Literature data: Caruel 1860; Corti 1956, under the name *J. acutus* L. var. *tommasinii* (Parl.) Arcangeli; Coaro 1987; Garbari 2001; Tomei et al. 2004; Sani & Tomei 2006; Bertacchi et al. 2007; Bertacchi et al. 2009; Petraglia 2013; Bertacchi & Lombardi 2014b; Saggese 2016; Lazzeri 2021, 2022

D **Juncus pygmaeus** Rich. ex Thuill.
T caesp - Eurimedit.
Literature data: Caruel 1860
Garbari (2001) reports only *Juncus* cf. *pygmaeus*.

NC **Juncus subnodulosus** Schrank
G rhiz - Europ.-Caucas.
Literature data: Baroni 1897-1908, under the name *J. obtusiflorus* Ehrh.

Juncus subulatus Forssk.
G rhiz - S-Medit.
Literature data: Petraglia 2013

Juncus tenageia L.f. subsp. **tenageia**
T caesp - Paleotemp.
Literature data: Caruel 1860; Corti 1956; Bonari et al. 2019

Juncus tenuis Willd.
H caesp - Orig. N-America
Literature data: Orlandi & Arduini 2010; Arduini & Ercoli 2012, also under the name *J.* cf. *squarrosus* L.

Luzula campestris (L.) DC. subsp. **campestris**
H caesp - Europ.-Caucas.
Literature data: Corti 1956, under the name *L. campestris* DC. var. *vulgaris* Gaud. forma *genuina* Asch.; Coaro 1987; Garbari 2001; Tomei et al. 2004; Sani & Tomei 2006; Arduini & Ercoli 2012; Bonari et al. 2019
Herbarium data: A. Sani, 2005, PI
Field observations: L. Pinzani, 2021; I. Arduini, 2024

Luzula forsteri (Sm.) DC.
H caesp - Eurimedit.
Literature data: Corti, 1951, 1956; Gellini et al. 1986; Coaro 1987; Garbari 2001; Tomei et al. 2004; Arduini & Ercoli 2012; Lombardi 2015; Bonari et al. 2019

Cyperaceae

Bolboschoenus glaucus (Lam.) S.G.Sm.
G rhiz - Subcosmop.
Literature data: Corti 1956, under the name *Scirpus maritimus* L. var. *macrostachys* (Willd.) Viv.; Lazzeri in Buono et al. 2022
Herbarium data: L. Pinzani, 2020, Herb. Pinzani

Bolboschoenus maritimus (L.) Palla

G rhiz - Cosmop.
Literature data: Caruel 1860, under the name *Scirpus maritimus* L.; Coaro 1987; Garbari 2001; Pignotti 2003 [*A. Caldesi*, 1862, TO; *C. Costa Reghini*, 1886, RO; *C. Costa Reghini*, 1886, TO; *P. Pellegrini*, 1912, PI]; Pedullà & Garbari, 2004; Tomei et al. 2004; Bertacchi et al. 2007; Bertacchi et al. 2009; Petraglia 2013; Bertacchi & Lombardi 2014b; Saggese 2016
Herbarium data: G. Gestri, 2018, PI; Partecipanti all'escursione Wikiplantbase #Parco di Migliarino - San Rossore - Massaciuccoli, 2018, PI

Carex acuta L.
G rhiz - Eurasiat.
Literature data: Garbari 2001, under the name *C. gracilis* Curtis; Petraglia 2013

Carex acutiformis Ehrh.
He/G rhiz - Eurasiat.
Literature data: Pedullà & Garbari, 2004
Field observations: I. Arduini, 2024

Carex caryophyllea Latourr.
H scap - Eurasiat.
Literature data: Corti 1956 [*P. Savi*, s.d., FI; *T. Caruel*, s.d., FI]; Coaro 1987; Tomei et al. 2004; Bonari et al. 2019
Herbarium data: L. Pinzani, 2021, Herb. Pinzani

Carex demissa Hornem. subsp. **demissa**
H caesp - Anfiatl.
Literature data: Coaro 1987, under the name *C. tumidicarpa* Anderss.

Carex depauperata Curtis ex With.
H caesp - Medit.-Subatl.
Literature data: Lombardi 2015

Carex distachya Desf.
H caesp - Stenomedit.
Literature data: Corti 1956; Garbari 2001; Tomei et al. 2004; Arduini & Ercoli 2012; Lombardi 2015; Bonari et al. 2019
Field observations: L. Pinzani, 2022

Carex distans L.
H caesp - Eurimedit.
Literature data: Corti 1956; Coaro 1987; Garbari 2001; Tomei et al. 2004; Lombardi 2015; Petraglia 2013; Lazzeri in Buono et al. 2022
Herbarium data: G. Gestri, 2018, PI
Field observations: M. D’Antraccoli, 2019

Carex divisa Huds.
G rhiz - Eurimedit.-Atl.
Literature data: Caruel 1860; Corti, 1951, 1956; Corti 1956 [*P. Fantozzi*, FI]; Garbari 2001; Bertacchi & Lombardi 2014b; Saggese 2016
Herbarium data: A. Sani, 2005, PI; L. Pinzani, 2020, Herb. Pinzani

Field observations: B. Pierini 2017; M. D’Antraccoli, 2020; L. Pinzani, 2022; I. Arduini, 2024

Carex divulsa Stokes
H caesp - Eurimedit.
Literature data: Corti 1956; Gellini et al. 1986; Garbari 2001; Tomei et al. 2004; Arduini & Ercoli 2012; Lazzeri 2021
Herbarium data: Partecipanti all'escursione Wikiplantbase #Parco di Migliarino - San Rossore - Massaciuccoli, 2018, PI
Field observations: F. Roma-Marzio, 2017; M. D'Antraccoli et F. Roma-Marzio, 2017, PI; M. D’Antraccoli, 2020; J. Franzoni, 2020; L. Pinzani, 2022; I. Arduini, 2024

Carex echinata Murray subsp. **echinata**
H caesp - Euroamer.
Literature data: Tomei et al. 2004

Carex elata All. subsp. **elata**
H caesp - Europ.-Caucas.
Literature data: Caruel 1860, under the name *C. stricta* Good.; Corti 1956; Gellini et al. 1986; Garbari 2001; Tomei et al. 2004; Bertacchi et al. 2007; Petraglia 2013
Field observations: I. Arduini, 2024

Carex extensa Gooden.
H caesp - Medit.-Atl.
Literature data: Caruel 1860; Corti 1956 [*P. Fantozzi*, FI]; Garbari 2001; Tomei et al. 2004; Petraglia 2013; Lazzeri 2021, 2022
Herbarium data: L. Pinzani, A. Mo, I. Pucci, S. Pastacaldi, 2022, Herb. Pinzani

Carex flacca Schreb. subsp. **erythrostachys** (Hoppe) Holub
G rhiz - Europ.
Literature data: Corti 1956, under the name *C. flacca* Schreb var. *arrecta* (Drej.) Briq.; Coaro 1987, under the name *C. flacca* Schreb. subsp. *serrulata* (Biv.) Greuter
Herbarium data: Partecipanti all'escursione Wikiplantbase #Parco di Migliarino - San Rossore - Massaciuccoli, 2018, PI

Carex flacca Schreb. subsp. **flacca**
G rhiz - Europ.
Literature data: Caruel 1860, under the name *C. glauca* Scop.; Gellini et al. 1986; Garbari 2001; Tomei et al. 2004; Arduini & Ercoli 2012; Lombardi 2015; Bonari et al. 2019; Lazzeri in Buono et al. 2022
Field observations: L. Pinzani, 2022

Carex flava L.
H caesp - Euroamer.
Literature data: Caruel 1860; Baroni 1897-1908; Garbari 2001

Carex hirta L.
G rhiz - Europ.-Caucas.
Literature data: Caruel 1860; Corti 1956; Garbari 2001; Tomei et al. 2004; Lombardi 2015; Lazzeri in Buono et al. 2022

Field observations: L. Pinzani, 2022

Carex leporina L.
H caesp - Eurosiber.
Literature data: Baroni 1897-1908; Corti 1956; Garbari 2001

NC **Carex liparocarpos** Gaudin subsp. **liparocarpos**
G rhiz - SE-Europ.
Literature data: Corti 1956 [*A. Targioni Tozzetti*, s.d., FI; *P. Savi*, s.d., FI; *O. Beccari*, s.d., FI]

Carex oederi Retz.
H caesp - Eurasiat.
Literature data: Garbari 2001; Tomei et al. 2004, under the name *C. serotina* Merat; Lombardi 2015, under the name *C. viridula* Michx.

NC **Carex olbiensis** Jord.
H caesp - Stenomedit.-Occid.
Literature data: Caruel 1860, under the name *C. strigosa* Huds.

Carex otrubae Podp.
H caesp - Eurimedit.-Atl.
Literature data: Caruel 1860, Corti 1956, 1970, Tomei et al. 2004under *C. vulpina* L.; Coaro 1987; Garbari 2001; Pedullà & Garbari, 2004; Sani & Tomei 2006; Bertacchi et al. 2007; Petraglia 2013; Bertacchi & Lombardi 2014b; Bonari et al. 2019; Lazzeri 2021, 2022
Herbarium data: A. Sani, 2005, PI; Partecipanti all'escursione Wikiplantbase #Parco di Migliarino - San Rossore - Massaciuccoli, 2018, PI
Field observations: partecipanti all'escursione Wikiplantbase #Parco di Migliarino - San Rossore - Massaciuccoli, 2018; M. D'Antraccoli, 2019; L. Pinzani, 2022

Carex pallescens L.
H caesp - Circumbor.
Literature data: Corti 1956, under the name *C. pallescens* L. forma *typica* Asch. u. Gr.; Gellini et al. 1986; Garbari 2001
Field observations: I. Arduini, 2024

Carex panicea L.
G rhiz - Eurosiber.
Literature data: Garbari 2001

Carex pendula Huds.
He - Eurasiat.
Literature data: Baroni 1897-1908; Corti 1956; Gellini et al. 1986; Tomei et al. 2004; Lombardi 2015; Bertacchi & Lombardi 2016
Field observations: partecipanti all'escursione Wikiplantbase #Parco di Migliarino - San Rossore - Massaciuccoli, 2018; I. Arduini, 2023

Carex praecox Schreb.
G rhiz - SE-Europ.
Literature data: Pedullà & Garbari, 2004; Lombardi 2015

Carex punctata Gaudin
H caesp - Eurimedit.-Subatl.
Literature data: Caruel 1860; Corti 1956 [*A. Fiori*, s.d., FI]; Corti 1956; Gellini et al. 1986; Garbari 2001; Arduini & Ercoli 2012
Herbarium data: Partecipanti all'escursione Wikiplantbase #Parco di Migliarino - San Rossore - Massaciuccoli, 2018, PI

Carex remota L.
H caesp - Europ.-Caucas.
Literature data: Corti, 1954, 1956, 1970; Gellini et al. 1986; Coaro 1987; Garbari 2001; Tomei et al. 2004; Lombardi 2015; Petraglia 2013; Bertacchi & Lombardi 2016
Herbarium data: Partecipanti all'escursione Wikiplantbase #Parco di Migliarino - San Rossore - Massaciuccoli, 2018, PI
Field observations: I. Arduini, 2024

Carex riparia Curtis
G rhiz/He - Eurasiat.
Literature data: Caruel 1860; Corti 1956, 1970; Gellini et al. 1986; Garbari 2001; Lombardi 2015
Field observations: B. Pierini, 2017, 2020; L. Pinzani, 2021, 2022; I. Arduini, 2023

Carex spicata Huds. subsp. **spicata**
H caesp - Eurasiat.
Literature data: Corti 1956, Garbari 2001, under the name *C. contigua* Hoppe; Gellini et al. 1986; Bonari et al. 2019

Carex strigosa Huds.
H caesp - Eurasiat.
Literature data: Garbari 2001; Tomei et al. 2004

Carex sylvatica Huds.
H caesp - Europ.-W-Asiat.
Literature data: Caruel 1860; Corti, 1954, 1956; Corti 1956 [*G. Savi*, s.d., FI]; Gellini et al. 1986; Coaro 1987; Garbari 2001; Tomei et al. 2004; Lombardi 2015
Field observations: I. Arduini, 2024

Cladium mariscus (L.) Pohl
G rhiz - Subcosmop.
Literature data: Caruel 1860; Baroni 1897-1908; Corti 1956; Garbari 2001; Tomei et al. 2004; Bertacchi et al. 2007; Petraglia 2013

Cyperus alternifolius L. subsp. *flabelliformis* Kük.
Field observations: observed on iNaturalist in 2022
Only cultivated

Cyperus capitatus Vand.
G rhiz - Stenomedit.
Literature data: Caruel 1860, under the name *Galilea mucronata* Parl.; Corti 1956 [*G. Savi*, s.d., FI]; Garbari 2001, under the name *C. kalli* (Forsskl) Murb.

Cyperus eragrostis Lam.
G rhiz - Orig. Subtrop.
Literature data: Anzalone, Brilli-Cattarini 1980 [*B. Anzalone*, *A. Brilli Cattarini*, 1978, FI, PESA RO]

Cyperus esculentus L.
Literature data: Garbari 2001

NC **Cyperus flavescens** L.
T caesp - Subcosmop.
Literature data: Caruel 1860; Corti 1956 [*P. Savi*, s.d., FI]

NC **Cyperus fuscus** L.
T caesp - Paleotemp.
Literature data: Caruel 1860

Cyperus glomeratus L.
He/T scap - Paleosubtrop.
Literature data: Peruzzi et al. 2007 [*B. Pierini*, 2007, PI; *J.-M. Tison*, 2007, Herb. Tison]
Herbarium data: F. Roma-Marzio, M. D'Antraccoli, 2015, PI
Field observations: J. Molina, 2007

Cyperus longus L.
G rhiz - Paleotemp.
Literature data: Caruel 1860; Corti 1956; Pedullà & Garbari, 2004; Lombardi 2015; Bertacchi et al. 2007; Lazzeri in Buono et al. 2022
Field observations: B. Pierini, 2014

Cyperus microiria Steud.
Literature data: Galasso et al. 2018 [*R. Guarino,* 2017, FI]

Cyperus odoratus L.
T caesp - Orig. Pantrop.
Literature data: La Rosa et al. 2008 [*J.-M. Tison*, 2007, Herb. Tison], under the name *C. strigosus* L.; Galasso et al. 2016 [*J.-M. Tison*, 2007, Herb. Tison]

Cyperus rotundus L.
G rhiz - Subcosmop.-Trop.-Subtrop.
Literature data: Caruel 1860; Savelli 1915; Lazzeri in Buono et al. 2022
Herbarium data: F. Roma-Marzio, 2014, PI; G. Bedini, 2016, PI; F. Roma-Marzio, M. D'Antraccoli, 2017, PI; L. Pinzani, 2020, Herb. Pinzani

NC *Cyperus serotinus* Rottb.
Literature data: Caruel 1860, under the name *C. monti* L.f.

Eleocharis palustris (L.) Roem. & Schult. subsp. **palustris**
G rhiz - Subcosmop.
Literature data: Garbari 2001; Tomei et al. 2004; Bertacchi et al. 2007; Petraglia 2013

Herbarium data: A. Sani, 2005, PI
Field observations: B. Pierini, 2017

Eleocharis uniglumis (Link) Schult.
G rhiz - Subcosmop.
Literature data: Baroni 1897-1908; Coaro 1987

NC **Fimbristylis bisumbellata** (Forssk.) Bubani
T scap - Pantropic.-Subtrop.
Literature data: Caruel 1860, under the name *F. dichotomus* Vahl

Isolepis cernua (Vahl) Roem. & Schult.
T scap/H caesp - Subcosmop.
Literature data: Caruel 1860, under the name *Scirpus savii* Seb. et Maur.; Pignotti 2003 [*M. Savelli*, 1915, FI]; Orlandi & Arduini 2010

Isolepis setacea (L.) R.Br.
T scap - Paleotemp.-Subtrop.
Literature data: Garbari 2001; Pignotti 2003 [*C. Costa Reghini*, 1886, PI]; Arduini & Ercoli 2012; Lombardi 2015
Herbarium data: A. Sani, 2005, PI

NC **Schoenoplectiella mucronata** (L.) J.Jung & H.K.Choi
He/T scap - Subcosmop.
Literature data: Caruel 1860, under the name *Scirpus mucronatus* L.; Pignotti 2003 [*Savi*, 1814, PI; *s. coll.*, s.d., FI]

Schoenoplectus lacustris (L.) Palla
G rhiz/He - Subcosmop.
Literature data: Caruel 1860, under the name *Scirpus lacustris* L.; Coaro 1987; Bertacchi et al. 2007

Schoenoplectus litoralis (Schrad.) Palla
G rhiz/He - Paleosubtrop.
Literature data: Caruel 1860, under the name *Scirpus litoralis* Schrad.; Petraglia 2013

Schoenoplectus pungens (Vahl) Palla
G rhiz - Subcosmop.
Literature data: Caruel 1860, under the name *Scirpus pungens* Vahl; Coaro 1987, under the name *S. americanus* (Pers.) Volkart; Pignotti 2003 [*P. Pellegrini*, 1897, PI]
Field observations: partecipanti all'escursione Wikiplantbase #Parco di Migliarino - San Rossore - Massaciuccoli, 2018

Schoenoplectus tabernaemontani (C.C.Gmel.) Palla
G rhiz/He - Eurosiber.
Literature data: Pignotti 2003 [s. *coll.*, 1909, PI; *Savelli*, 1916, FI; *G. Fiorini, L. Pignotti, S. Turrini*, 1995, FI]

NC **Schoenoplectus triqueter** (L.) Palla

G rhiz/He - Circumbor.
Literature data: Caruel 1860, under the name *Scirpus triqueter* L.; Pignotti 2003 [*Savi*, 1817, PI]

Schoenus nigricans L.
H caesp - Subcosmop.
Literature data: Caruel 1860; Baroni 1897-1908; Corti 1956 [*P. Savi*, s.d., FI]; Corti 1956; Coaro 1987; Garbari 2001; Tomei et al. 2004; Sani & Tomei 2006; Bertacchi et al. 2007; Bertacchi et al. 2009; Arduini & Ercoli 2012; Petraglia 2013; Bertacchi & Lombardi 2014b; Lombardi 2015; Bonari et al. 2019; Lazzeri in Buono et al. 2022
Herbarium data: F. Roma-Marzio, M. D'Antraccoli, 2017, PI
Field observations: L. Pinzani, 2022

Scirpoides holoschoenus (L.) Soják
G rhiz - Stenomedit.
Literature data: Caruel 1860, Baroni 1897-1908, Sani & Tomei 2006, Bonari et al. 2019, under the name *Scirpus holoschoenus* L.; Corti 1956 [*P. Fantozzi*, FI; *M. Savelli*, FI]; Corti 1956, under the name *Scirpus holoschoenus* L. var. *australis* Koch, var. *romanus* Koch; Coaro 1987, under the name *Holoschoenus australis* (L.) Rchb.; Garbari 2001, Tomei et al. 2004, Bertacchi et al. 2007, Bertacchi et al. 2009, under the name *Holoschoenus romanus* (L.) Fritsch, *Holoschoenus australis* (L.) Rchb.; Pignotti 2003 [*Savelli*, 1916, FI; *Chiarugi, Corti*, 1951, FI; *Bavazzano, Ricceri*, 1963, FI]; Pedullà & Garbari, 2004, under the name *Holoschoenus australis* (L.) Rchb.; Arduini & Ercoli 2012; Petraglia 2013; Lombardi 2015; Saggese 2016; Lazzeri in Buono et al. 2022
Herbarium data: A. Sani, 2005, PI; Partecipanti all'escursione Wikiplantbase #Parco di Migliarino - San Rossore - Massaciuccoli, 2018, PI; L. Pinzani, 2020, Herb. Pinzani

Poaceae

Achnatherum bromoides (L.) P.Beauv.
H caesp - Stenomedit.
Literature data: Caruel 1860, under the name *Stipa aristella* L.; Garbari 2001, under the name *Stipa bromoides* (L.) Dorfl.; Arduini & Ercoli 2012; Lombardi 2015

Aeluropus littoralis (Gouan) Parl. subsp. **littoralis**
G rhiz - Medit.-Turan.
Literature data: Caruel 1860, under the name *Dactylis litoralis* Willd.; Coaro 1987; Sani & Tomei 2005 (*A. Sani, P.E. Tomei*, 2005, FI); Sani & Tomei 2006 [*A. Sani*, 2005, PI]; Bertacchi et al. 2007; Petraglia 2013; Bertacchi & Lombardi 2014b; Saggese 2016
Herbarium data: T. Fiaschi, 2018, Siena; Partecipanti all'escursione Wikiplantbase #Parco di Migliarino - San Rossore - Massaciuccoli, 2018, PI; L. Pinzani, 2020, Herb. Pinzani

Agrostis canina L.
H caesp - Eurosiber.
Literature data: Caruel 1860; Tomei et al. 2004

NC **Agrostis capillaris** L.
H caesp - Circumbor.
Literature data: Caruel 1860, under the name *A. vulgaris* With.

Agrostis castellana Boiss. & Reut.

H caesp - Eurimedit.-Occid.
Literature data: Coaro 1987; Garbari 2001; Arduini & Ercoli 2012; Lombardi 2015

NC **Agrostis gigantea** Roth subsp. **gigantea**
H caesp - Circumbor.
Literature data: Corti 1956 [*P. Savi*, s.d., FI]; Corti 1956, under the name *A. alba* L. var. *gigantea* (Gaud.) Meyer

D **Agrostis stolonifera** L. subsp. **maritima** (Lam.) Vasc.
H rept - Circumbor.
Literature data: Caruel 1860, Corti 1956, under the name *A. alba* L. var. *maritima* Parl.; Bertacchi et al. 2007; Bertacchi & Lombardi 2014b

Agrostis stolonifera L. subsp. **stolonifera**
H rept - Circumbor.
Literature data: Caruel 1860, under the name *A. alba* L.; Gellini et al. 1986; Garbari 2001; Tomei et al. 2004; Lombardi 2015; Saggese 2016; Lazzeri in Buono et al. 2022
Field observations: I. Arduini, 2024

Aira caryophyllea L.
T scap - Paleo-Subtrop.
Literature data: Caruel 1860; Corti, 1951, 1956, under the name *A. caryophyllea* L. subsp. *eucaryophyllea* Becherer var. *genuina* Mutel; Garbari 2001; Tomei et al. 2004; Sani & Tomei 2006; Arduini & Ercoli 2012; Lombardi 2015; Bonari et al. 2019
Field observations: L. Pinzani, 2021

Aira cupaniana Guss.
T scap - Stenomedit.-Occid.
Literature data: Tomei et al. 2004

Aira elegans Willd. subsp. **elegans**
T scap - Eurimedit.
Literature data: Caruel 1860, under the name *A. capillaris* Host; Coaro 1987; Tomei et al. 2004

Aira multiculmis Dumort.
T scap - Subtrop.
Literature data: Peruzzi et al. 2018 [*G. Bonari*, 2015, SIENA]

Alopecurus bulbosus Gouan subsp. **bulbosus**
H caesp - Eurimedit.
Literature data: Caruel 1860; Coaro 1987; Petraglia 2013

Alopecurus myosuroides Huds.
T scap - Paleotemp.-Subcosmop.
Literature data: Caruel 1860, under the name *A. agrestis* L.; Garbari 2001
Herbarium data: J. Franzoni, 2020, Herb. Franzoni
Field observations: B. Pierini, 2017; L. Pinzani, 2022; H. Öhm, 2024

NC **Alopecurus rendlei** Eig

T scap - Eurimedit.
Literature data: Caruel 1860, under the name *A. utriculatus* Pers.

Anisantha diandra (Roth) Tutin ex Tzvelev
T scap - Eurimedit.
Literature data: Corti 1956, under the name *Bromus villosus* Forsk. var. *maximus* (Desf.) Asch. u. Gr. forma *gussonei* (Parl.) Asch. u. Gr.; Coaro 1987, under the name *B. gussonei* Parl.; Saggese 2016; Bonari et al. 2019, under the name *Bromus diandrus*
Herbarium data: A. Sani, 2007, PI; G. Bonari, 2015, SIENA; F. Roma-Marzio, 2016, PI; T. Fiaschi, 2018, SIENA
Field observations: partecipanti all'escursione Wikiplantbase #Parco di Migliarino - San Rossore - Massaciuccoli, 2018; L. Pinzani, 2022

Anisantha madritensis (L.) Nevski subsp. **madritensis**
T scap - Eurimedit.
Literature data: Caruel 1860, Corti, 1951, 1956, Garbari 2001, Sani & Tomei 2006 [*A. Sani*, 2005, PI], Bertacchi et al. 2009, Bertacchi et al. 2010, Bertacchi & Lombardi 2014a, Bertacchi & Lombardi 2014b, Bonari et al. 2019, under the name *Bromus madritensis* L.; Saggese 2016; Lazzeri in Buono et al. 2022
Herbarium data: T. Fiaschi, 2018, SIENA; F. Roma-Marzio, L. Peruzzi, 2019, PI

Anisantha rigida (Roth) Hyl.
T scap - Paleo-Subtrop.
Literature data: Caruel 1860, under the name *Bromus maximus* Desf.; Garbari 2001, under the name *Bromus rigidus* Roth subsp. *rigidus*

Anisantha rubens (L.) Nevski
T scap - Medit.-Turan.
Literature data: Garbari 2001, under the name *Bromus rubens* L.

Anisantha sterilis (L.) Nevski
T scap - Eurimedit.-Turan.
Literature data: Caruel 1860, Corti 1956, Garbari 2001, Sani & Tomei 2006, Arduini & Ercoli 2012, under the name *Bromus sterilis* L.; Lazzeri 2021
Herbarium data: D. Ciccarelli, 2012, PI; F. Roma-Marzio, L. Peruzzi, 2019, PI
Field observations: I. Arduini, 2024

Anthoxanthum odoratum L.
H caesp - Eurasiat.
Literature data: Caruel 1860; Corti 1956, under the name *A. odoratum* L. var. *glabrescens* Celak subvar. *vulgatum* Asch. u. Gr., var. *villosum* Lois. ex DC. subvar. *corsicum* Briq.; Coaro 1987; Garbari 2001; Tomei et al. 2004; Sani & Tomei 2006; Arduini & Ercoli 2012; Lombardi 2015; Saggese 2016; Bonari et al. 2019
Herbarium data: A. Sani, 2007, PI; T. Fiaschi, 2018, SIENA
Field observations: L. Pinzani, 2021; I. Arduini, 2024

Arrhenatherum elatius (L.) P.Beauv. ex J.Presl & C.Presl subsp. **elatius**
H caesp - Paleotemp.
Literature data: Corti, 1954, under the name *Avena alba* Vahl; Bonari et al. 2019

Field observations: L. Pinzani, 2021

Arundo donax L.
G rhiz - Orig. C-Asia
Literature data: Tomei et al. 2004; Lazzeri 2021
Field observations: partecipanti all'escursione Wikiplantbase #Parco di Migliarino - San Rossore - Massaciuccoli, 2018; L. Pinzani, 2020, 2021, 2022

Arundo plinii Turra
G rhiz - Stenomedit.
Literature data: Hardion 2012 [*Hardion, Vila*, s.d., MARS]
Field observations: G. Bedini, 2014
Assessed as DD in the Red List of the Italian Vascular Flora (Rossi et al., 2020)

Avellinia festucoides (Link) Valdés & H.Scholz
T scap - Stenomedit.
Literature data: Corti 1956, Garbari 2001, Lombardi 2015, under the name *A. michelii* (Savi) Parl.

Avena barbata Pott ex Link
T scap - Eurimedit.-Turan.
Literature data: Caruel 1860; Corti 1956, under the name *A. alba* Vahl; Coaro 1987; Garbari 2001; Sani & Tomei 2006; Bertacchi et al. 2010; Bertacchi & Lombardi 2014a; Lombardi 2015; Saggese 2016; Bonari et al. 2019; Lazzeri 2021
Field observations: L. Peruzzi, 2018; M. D’Antraccoli, 2019

NC *Avena fatua* L.
Literature data: Caruel 1860

Avena sterilis L.
T scap - Orig. Eurimedit.-Turan.
Literature data: Caruel 1860; Bertacchi et al. 2009; Saggese 2016; Lazzeri in Buono et al. 2022

Avena strigosa Schreb.
Literature data: Pedullà & Garbari, 2004
Only cultivated

NC **Beckmannia eruciformis** (L.) Host subsp. **eruciformis**
G rhiz - Eurosiber.
Literature data: Caruel 1860

Bellardiochloa variegata (Lam.) Kerguélen
H caesp - Orof. S-Europ.
Literature data: Coaro 1987, under the name *Poa violacea* Bellardi

NC **Bothriochloa ischaemum** (L.) Keng
H caesp - Eurosiber.
Literature data: Caruel 1860, under the name *Andropogon ischaemum* L.

NC **Brachypodium distachyon** (L.) P.Beauv.

T scap - Stenomedit.-Turan.
Literature data: Caruel 1860

Brachypodium retusum (Pers.) P.Beauv.
H caesp - Stenomedit.-Occid.
Literature data: Corti 1956, under the name *B. ramosum* (L.) Roem. et Sch; Garbari 2001; Arduini & Ercoli 2012; Bonari et al. 2019
Herbarium data: G. Bonari, 2015, SIENA

Brachypodium rupestre (Host) Roem. & Schult.
H caesp - Eurasiat.
Literature data: Tomei et al. 2004; Bonari et al. 2019; Lazzeri 2021

Brachypodium sylvaticum (Huds.) P.Beauv. subsp. **sylvaticum**
H caesp - Paleotemp.
Literature data: Caruel 1860; Corti, 1951, 1954, 1956; Gellini et al. 1986; Coaro 1987; Garbari 2001; Tomei et al. 2004; Arduini & Ercoli 2012; Lombardi 2015; Bertacchi & Lombardi 2016; Bonari et al. 2019
Herbarium data: Partecipanti all'escursione Wikiplantbase #Parco di Migliarino - San Rossore - Massaciuccoli, 2018, PI
Field observations: L. Pinzani, 2022; I. Arduini, 2024

Briza media L.
H caesp - Eurosiber.
Literature data: Coaro 1987
Field observations: L. Pinzani, 2022

Briza minor L.
T scap - Subcosmop.
Literature data: Caruel 1860; Corti 1956; Coaro 1987; Garbari 2001; Saggese 2016; Lazzeri in Buono et al. 2022
Herbarium data: T. Fiaschi, 2018, SIENA; Partecipanti all'escursione Wikiplantbase #Parco di Migliarino - San Rossore - Massaciuccoli, 2018, PI
Field observations: partecipanti all'escursione Wikiplantbase #Parco di Migliarino - San Rossore - Massaciuccoli, 2018; L. Pinzani, 2021

NC **Bromus arvensis** L.
T scap - Eurosiber.
Literature data: Caruel 1860, under the name *Serrafalcus arvensis* Godr.

Bromus commutatus Schrad. subsp. **commutatus**
T scap - Europ.
Literature data: Baroni 1897-1908, under the name *Serrafalcus commutatus* Bab.; Corti 1956; Lazzeri 2021
Herbarium data: T. Fiaschi, 2018, SIENA

Bromus hordeaceus L. subsp. **hordeaceus**
T scap - Subcosmop.

Literature data: Caruel 1860, under the name *Serrafalcus mollis* Parl.; Corti 1956, under the name *B. mollis* L.; Coaro 1987; Garbari 2001; Lombardi 2015; Bertacchi & Lombardi 2016
Herbarium data: T. Fiaschi, 2018, SIENA; Partecipanti all'escursione Wikiplantbase #Parco di Migliarino - San Rossore - Massaciuccoli, 2018, PI; L. Pinzani, 2020, Herb. Pinzani
Field observations: M. D'Antraccoli, 2019

Bromus hordeaceus L. subsp. **thominei** (Hardouin) Braun-Blanq.
T scap - Subcosmop.
Literature data: Bartolucci et al. 2019 [*G. Bonari*, 2015, FI]; Bonari et al. 2019
Herbarium data: G. Bonari, 2015, SIENA

NC **Bromus racemosus** L. subsp. **racemosus**
T scap - Europ.-Caucas.
Literature data: Caruel 1860, under the name *Serrafalcus racemosus* Parl.; Corti 1956

Bromus scoparius L.
T scap - Stenomedit.
Herbarium data: A. Sani, 2005, PI

Bromus squarrosus L.
T scap - Paleotemp.
Literature data: Caruel 1860, under the name *Serrafalcus squarrosus* Bab.; Saggese 2016

Calamagrostis arenaria (L.) Roth subsp. **arundinacea** (Husn.) Banfi, Galasso & Bartolucci
G rhiz - Eurimedit.
Literature data: Caruel 1860; Corti 1956, 1970, Garbari 2001, Sani & Tomei 2006, Bertacchi et al. 2009, Bertacchi et al. 2010, Bertacchi & Lombardi 2014a, Bertacchi & Lombardi 2014b, under the name *Ammophila arenaria* (Roth) Link

Calamagrostis epigejos (L.) Roth subsp. **epigejos**
H caesp - Eurosiber.
Literature data: Caruel 1860; Garbari 2001; Lombardi 2015; Saggese 2016
Field observations: F. Roma-Marzio, 2019

NC **Catabrosa aquatica** (L.) P.Beauv.
G rhiz - Circumbor.
Literature data: Caruel 1860

Catapodium balearicum (Willk.) H.Scholz
T scap - Medit.-Atl.
Literature data: Garbari 2001, Tomei et al. 2004, under the name *C. marinum* (L.) Hubbard; Sani & Tomei 2006; Brullo et al. 2003 [*M. Savelli*, 1914, FI]
Herbarium data: A. Sani, 2005, PI; T. Fiaschi, 2018, SIENA; Partecipanti all'escursione Wikiplantbase #Parco di Migliarino - San Rossore - Massaciuccoli, 2018, PI; L. Pinzani, 2020, Herb. Pinzani
Field observations: H. Öhm, 2024

NC **Catapodium hemipoa** (Delile ex Spreng.) M.Laínz
T scap - Stenomedit.-Occid.

Literature data: Caruel 1860, under the name *Scleropoa hemipoa* Parl.

Catapodium rigidum (L.) C.E.Hubb. subsp. **majus** (C.Presl) F.H.Perring & P.D.Sell
T scap - Eurimedit.
Literature data: Pierini 2013 [*B. Pierini*, 2013, PI]
Herbarium data: T. Fiaschi, 2018, SIENA; G. Gestri, 2018, PI

Catapodium rigidum (L.) C.E.Hubb. subsp. **rigidum**
T scap - Eurimedit.
Literature data: Caruel 1860, Corti 1956, under the name *Scleropoa rigida* Gris.; Bertacchi et al. 2009
Herbarium data: L. Pinzani, 2021, Herb. Pinzani
Field observations: L. Pinzani, 2021, 2022; I. Arduini, 2024

Cenchrus spinifex Cav.
T scap - Orig. N-America
Literature data: Bertacchi et al. 2009, Lombardi 2015, under the name *C. incertus* L.
Herbarium data: D. Ciccarelli, 2011, PI; J. Franzoni, 2019, Herb. Franzoni; L. Pinzani, 2020, Herb. Pinzani

Ceratochloa cathartica (Vahl) Herter
Literature data: Peruzzi et al. 2020 [*L. Pinzani*, 2020, PI]
Field observations: L. Pinzani, 2022

Cortaderia selloana (Schult. & Schult.f.) Asch. & Graebn.
H caesp - Orig. S-America
Literature data: Lazzeri in Buono et al. 2022
Field observations: L. Pinzani, 2020, 2022

Corynephorus articulatus (Desf.) P.Beauv.
T scap - Stenomedit.
Literature data: Caruel 1860; Corti, 1954, 1956; Arduini & Ercoli 2012

Corynephorus divaricatus (Pourr.) Breistr.
T scap -
Literature data: Garbari 2001; Lombardi 2015
Assessed as LC in the Red List of the Italian Vascular Flora (Rossi et al. 2020)

NC **Cutandia maritima** (L.) Benth. ex Barbey
T scap -
Literature data: Caruel 1860, under the name *Scleropoa maritima* Parl.

Cynodon dactylon (L.) Pers.
G rhiz - Termocosmop.
Literature data: Caruel 1860; Corti 1956 [*M. Savelli*, s.d., FI]; Corti 1956; Coaro 1987; Garbari 2001; Tomei et al. 2004; Sani & Tomei 2006; Bertacchi et al. 2009; Petraglia 2013; Bertacchi & Lombardi 2014b; Lombardi 2015; Lazzeri in Buono et al. 2022
Herbarium data: A. Sani, 2007, PI; D. Ciccarelli, 2012, PI

Field observations: partecipanti all'escursione Wikiplantbase #Parco di Migliarino - San Rossore - Massaciuccoli, 2018; L. Peruzzi, 2019; L. Pinzani, 2020, 2021, 2022; A. Mo, 2023

NC **Cynosurus cristatus** L.
H caesp -
Literature data: Caruel 1860

Cynosurus echinatus L.
T scap - Eurimedit.
Literature data: Caruel 1860; Coaro 1987
Herbarium data: T. Fiaschi, 2018, SIENA
Field observations: partecipanti all'escursione Wikiplantbase #Parco di Migliarino - San Rossore - Massaciuccoli, 2018; L. Pinzani, 2022

Dactylis glomerata L. subsp. **glomerata**
H caesp - Paleotemp.
Literature data: Caruel 1860; Corti, 1951, 1956; Coaro 1987; Garbari 2001; Pedullà & Garbari, 2004; Tomei et al. 2004; Arduini & Ercoli 2012; Saggese 2016; Lazzeri 2021, 2022
Herbarium data: L. Pinzani, 2021, Herb. Pinzani
Field observations: partecipanti all'escursione Wikiplantbase #Parco di Migliarino - San Rossore - Massaciuccoli, 2018; M. D’Antraccoli, 2019; L. Pinzani, 2020, 2021, 2022

Dactylis glomerata L. subsp. **hispanica** (Roth) Nyman
H caesp - Stenomedit.
Literature data: Garbari 2001; Sani & Tomei 2006; Bonari et al. 2019
Herbarium data:
Field observations: partecipanti all'escursione Wikiplantbase #Parco di Migliarino - San Rossore - Massaciuccoli, 2018; F. Roma-Marzio, 2021

Danthonia decumbens (L.) DC. subsp. **decumbens**
H caesp - Europ.
Literature data: Caruel 1860; Orlandi & Arduini 2010; Arduini & Ercoli 2012; Lombardi 2015

Dasypyrum villosum (L.) P.Candargy
T scap - Medit.-Turan.
Literature data: Caruel 1860, under the name *Triticum villosum* Bieb.
Field observations: partecipanti all'escursione Wikiplantbase #Parco di Migliarino - San Rossore - Massaciuccoli, 2018; L. Pinzani, 2020, 2021, 2022

Deschampsia cespitosa (L.) P.Beauv. subsp. **cespitosa**
H caesp - Subcosmop.-Temp.
Literature data: Caruel 1860; Corti 1956 [*O. Beccari*, s.d., FI]; Corti 1956, under the name *D. caespitosa* (L.) Pal. Beauv. var. *altissima* (Lam.) Gremli; Garbari 2001; Lombardi 2015
Field observations: I. Arduini, 2024

Digitaria ciliaris (Retz.) Koeler
Herbarium data: L. Pinzani, 2020, Herb. Pinzani

Digitaria debilis (Desf.) Willd.

T scap - Stenomedit.-Sudafr.
Literature data: Baroni 1897-1908; Tomei et al. 2004

Digitaria ischaemum (Schreb.) Muhl. subsp. **ischaemum**
T scap - Subcosmop.
Literature data: Orlandi & Arduini 2010

Digitaria sanguinalis (L.) Scop.
T scap - Cosmop.
Literature data: Caruel 1860; Lombardi 2015
Field observations: B. Pierini, 2014

Echinochloa colonum (L.) Link subsp. *colonum*
Herbarium data: J. Franzoni, 2019, Herb. Franzoni

Echinochloa crus-galli (L.) P.Beauv. subsp. **crus-galli**
T scap - Subcosmop.
Literature data: Caruel 1860, under the name *Panicum crus-galli* L.; Baroni 1897-1908, under the name *Panicum phyllopogon* Stapf in Hooker; Lazzeri in Buono et al. 2022
Herbarium data: T. Fiaschi, 2018, SIENA; Partecipanti all'escursione Wikiplantbase #Parco di Migliarino - San Rossore - Massaciuccoli, 2018, PI
Field observations: B. Pierini, 2014; L. Peruzzi, 2019

Eleusine indica (L.) Gaertn.
T scap - Orig. Subtrop.
Literature data: Peruzzi et al. 2017a [*G. Bedini*, 2015, PI]
Herbarium data: L. Pinzani, 2020, Herb. Pinzani
Field observations: B. Pierini, 2014; G. Bedini, 2018

Elymus caninus (L.) L.
H caesp - Circumbor.
Literature data: Caruel 1860, under the name *Triticum caninum* L.
Herbarium data: T. Fiaschi, 2018, SIENA

Elymus repens (L.) Gould subsp. **repens**
G rhiz - Circumbor.
Literature data: Caruel 1860, under the name *Triticum repens* L.; Corti 1956, under the name *Agropyron repens* (L.) Pal. Beauv. forma *aristatum* (Neilr.) Hayek subf. *dumetorum* (Hoffm.) Rchb.; Garbari 2001, Pedullà & Garbari, 2004, Tomei et al. 2004, Sani & Tomei 2006, Petraglia 2013, under the name *Agropyron repens* (L.) Beauv.; Saggese 2016
Herbarium data: A. Sani, 2005, PI; J. Franzoni, 2019, Herb. Franzoni
Field observations: partecipanti all'escursione Wikiplantbase #Parco di Migliarino - San Rossore - Massaciuccoli, 2018; L. Pinzani, 2021, 2022

NC **Eragrostis cilianensis** (All.) Vignolo ex Janch.
T scap - Termocosmop.
Literature data: Caruel 1860, under the name *E. megastachya* Link

Eragrostis minor Host

T scap - Subcosmop.
Literature data: Orlandi & Arduini 2010
Herbarium data: B. Pierini, 2024, PI

Eragrostis pectinacea (Michx.) Nees
T scap - N-Americ.
Field observations: observed on iNaturalist in 2024

NC **Eragrostis pilosa** (L.) P.Beauv. subsp. **pilosa**
T scap - Cosmop.
Literature data: Caruel 1860

Festuca ambigua Le Gall
T caesp - Subcosmop.
Literature data: Caruel 1860, under the name *F. ciliata* Danth.; Corti 1956, Garbari 2001, Lombardi 2015, under the name *Vulpia ciliata* (Danth.) Link

Festuca bromoides L.
T caesp - Paleotemp.
Literature data: Corti 1956, Garbari 2001, Arduini & Ercoli 2012, Lombardi 2015, under the name *Vulpia bromoides* (L.) Gray

Festuca fasciculata Forssk.
T caesp - Medit.-Atl.
Literature data: Caruel 1860; Corti 1956 [*Poggi, Rossetti*, s.d., FI]; Garbari 2001, under the name *Vulpia membranacea* auct., non (L.) Dumort.; Tomei et al. 2004, Sani & Tomei 2006 [*A. Sani*, 2005, PI], Bertacchi et al. 2009; Bertacchi et al. 2010, Lombardi 2015, under the name *Vulpia fasciculata* (Forssk.) Fritsch; Tomei et al. 2004, Bertacchi & Lombardi 2014a, under the name *Vulpia membranacea* (L.) Link
Herbarium data: F. Roma-Marzio, 2012, PI; T. Fiaschi, 2018, SIENA

NC **Festuca geniculata** (L.) Lag. & Rodr. subsp. **geniculata**
T caesp - Stenomedit.-Occid.
Literature data: Caruel 1860

Festuca incurva (Gouan) Gutermann
T scap - Eurimedit.
Literature data: Caruel 1860, under the name *Psilurus nardoides* Trin.; Corti, 1954, 1956, Garbari 2001, Lombardi 2015, under the name *Psilurus incurvus* (Gouan) Schinz & Thell.

Festuca ligustica (All.) Bertol.
T caesp - Stenomedit.-Occid.
Literature data: Caruel 1860; Corti 1956 [*P. Savi*, s.d., FI]; Corti 1956, under the name *Vulpia ligustica* (All.) Lk; Saggese 2016, under the name *Loretia ligustica* (All.) Duval-Jouve
Field observations: H. Öhm, 2024

Festuca muralis Kunth
T caesp - Stenomedit.-Occid.
Herbarium data: T. Fiaschi, 2018, SIENA

Festuca myuros L. subsp. **myuros**
T caesp - Subcosmop.
Literature data: Caruel 1860; Corti, 1951, 1956, Coaro 1987, Orlandi & Arduini 2010, Arduini & Ercoli 2012, Bonari et al. 2019, under the name *Vulpia myuros* Gmel.
Herbarium data: T. Fiaschi, 2018, SIENA

Gaudinia fragilis (L.) P.Beauv.
T scap - Eurimedit.
Literature data: Caruel 1860, under the name *G. avenacea* Pal.; Corti, 1951, 1956; Petraglia 2013; Lombardi 2015; Saggese 2016; Lazzeri 2021, 2022
Herbarium data: T. Fiaschi, 2018, SIENA; Partecipanti all'escursione Wikiplantbase #Parco di Migliarino - San Rossore - Massaciuccoli, 2018, PI
Field observations: partecipanti all'escursione Wikiplantbase #Parco di Migliarino - San Rossore - Massaciuccoli, 2018; L. Pinzani, 2021, 2022

Glyceria fluitans (L.) R.Br.
I rad/G rhiz - Subcosmop.
Literature data: Caruel 1860; Mainardi 1982

Glyceria maxima (Hartm.) Holmb. subsp. **maxima**
G rhiz/I rad - Circumbor.
Literature data: Caruel 1860; Pedullà & Garbari, 2004
Field observations: partecipanti all'escursione Wikiplantbase #Parco di Migliarino - San Rossore - Massaciuccoli, 2018

NC **Glyceria spicata** Guss. subsp. **spicata**
G rhiz - Subcosmop.
Literature data: Caruel 1860

Holcus lanatus L. subsp. **lanatus**
H caesp - Circumbor.
Literature data: Caruel 1860; Corti, 1951, 1956, under the name *H. lanatus* L. var. *lanatus*; Gellini et al. 1986; Coaro 1987; Garbari 2001; Tomei et al. 2004; Sani & Tomei 2006; Arduini & Ercoli 2012; Lombardi 2015; Saggese 2016; Bonari et al. 2019; Lazzeri in Buono et al. 2022
Herbarium data: Partecipanti all'escursione Wikiplantbase #Parco di Migliarino - San Rossore - Massaciuccoli, 2018, PI; L. Pinzani, 2021, Herb. Pinzani
Field observations: I. Arduini, 2024

Holcus mollis L. subsp. **mollis**
H caesp - Circumbor.
Literature data: Lombardi 2015
Herbarium data: T. Fiaschi, 2018, SIENA

NC **Hordeum bulbosum** L.
H caesp - Paleotrop.
Literature data: Corti 1956 [*Levier*, s.d., FI; *P. Fantozzi*, s.d., FI]; Corti 1956, under the name *H. nodosum* L.

Hordeum marinum Huds.
T scap - Eurimedit.-Occid.
Literature data: Caruel 1860; Corti 1956 [*P. Fantozzi*, s.d., FI]; Corti 1956, Garbari 2001, Tomei et al. 2004, Sani & Tomei 2006, Bertacchi et al. 2007, under the name *H. maritimum* With.; Petraglia 2013; Saggese 2016

Hordeum murinum L. subsp. **leporinum** (Link) Arcang.
T scap - Circumbor.
Literature data: Caruel 1860, Bertacchi et al. 2009, under the name *H. murinum* L.; Corti 1956; Coaro 1987; Garbari 2001; Lombardi, 2015, under the name *H. murinum* L. subsp. *murinum*; Bonari et al. 2019
Herbarium data: T. Fiaschi, 2018, SIENA; Partecipanti all'escursione Wikiplantbase #Parco di Migliarino - San Rossore - Massaciuccoli, 2018, PI
Field observations: partecipanti all'escursione Wikiplantbase #Parco di Migliarino - San Rossore - Massaciuccoli, 2018; L. Peruzzi, 2018; L. Pinzani, 2022; I. Arduini, 2024

Hordeum secalinum Schreb.
H caesp - Eurimedit.-Occid.-Subatl.
Literature data: Caruel 1860; Garbari 2001; Tomei et al. 2004

Imperata cylindrica (L.) Raeusch.
G rhiz - Cosmop.
Literature data: Caruel 1860, under the name *I. arundinacea* Cyr.; Baroni 1897-1908; Montelucci 1964; Garbari 2001; Tomei et al. 2004; Sani & Tomei 2006 [*A. Sani*, 2005, PI]
Herbarium data: L. Pinzani, 2020, Herb. Pinzani

Lagurus ovatus L.
T scap - Eurimedit.
Literature data: Corti, 1951, 1954, under the name *L. ovatus* L. subsp. *ovatus* var. *glabrilemmis* Maire; Corti 1956 [*P. Savi*, s.d., FI; *Levier*, s.d., FI; *Rossetti*, s.d., FI]; Coaro 1987; Garbari 2001; Tomei et al. 2004; Sani & Tomei 2006; Bertacchi et al. 2009; Bertacchi et al. 2010; Arduini & Ercoli 2012; Bertacchi & Lombardi 2014a; Lombardi 2015; Saggese 2016; Bonari et al. 2019
Herbarium data: F. Roma-Marzio, 2012, PI; T. Fiaschi, 2018, SIENA; L. Pinzani, 2019, Herb. Pinzani
Field observations: L. Pinzani, 2021, 2022; A. Mo, 2023

Lolium arundinaceum (Schreb.) Darbysh. subsp. **arundinaceum**
H caesp - Paleotemp.
Literature data: Caruel 1860, Tomei et al. 2004, under the name *Festuca arundinacea* Schreb.; Lombardi 2015, under the name *Schedonorus arundinaceus* (Schreb.) Dumort. subsp. arundinaceus; Lazzeri in Buono et al. 2022
Herbarium data: J. Franzoni, 2020, Herb. Franzoni

Lolium giganteum (L.) Darbysh.
H caesp - Eurasiat.
Literature data: Corti 1956, under the name *Festuca gigantea* Vill. var. *nemoralis* Asch. u. Gr.

Lolium multiflorum Lam.
T scap/H scap - Eurimedit.

Literature data: Caruel 1860, under the name *L. boucheanum* Kunth; Saggese 2016; Lazzeri in Buono et al. 2022
Herbarium data: T. Fiaschi, 2018, SIENA; Partecipanti all'escursione Wikiplantbase #Parco di Migliarino - San Rossore - Massaciuccoli, 2018, PI; L. Pinzani, 2020, Herb. Pinzani
Field observations: partecipanti all'escursione Wikiplantbase #Parco di Migliarino - San Rossore - Massaciuccoli, 2018; L. Pinzani, 2021, 2022

Lolium perenne L.
H caesp - Eurasiat.
Literature data: Caruel 1860; Corti, 1954, 1956, under the name *L. perenne* L. var. *typicum* Fiori ; Garbari 2001; Lazzeri in Buono et al. 2022
Herbarium data: Partecipanti all'escursione Wikiplantbase #Parco di Migliarino - San Rossore - Massaciuccoli, 2018, PI

Lolium rigidum Gaudin subsp. **rigidum**
T scap - Paleo-Subtrop.
Literature data: Garbari 2001

Lolium temulentum L.
T scap - Subcosmop.
Literature data: Caruel 1860; Coaro 1987

Macrobriza maxima (L.) Tzvelev
T scap - Paleo-Subtrop.
Literature data: Caruel 1860, Coaro 1987, Garbari 2001, Bertacchi et al. 2009, Lombardi 2015, under the name *Briza maxima* L.
Herbarium data: T. Fiaschi, 2018, SIENA

Melica ciliata L.
H caesp - Eurimedit.
Literature data: Caruel 1860; Tomei et al. 2004

Melica transsilvanica Schur
H caesp - Eurimedit.-Turan.
Herbarium data: J. Franzoni, 2019, Herb. Franzoni; F. Roma-Marzio, L. Peruzzi, 2019, PI; M. Tiburtini, 2019, Herb. Tiburtini

Melica uniflora Retz.
H caesp - Paleotemp.
Literature data: Coaro 1987

Milium effusum L. subsp. **effusum**
G rhiz - Circumbor.
Literature data: Coaro 1987

Molinia caerulea (L.) Moench
H caesp - Europ.-Caucas.
Literature data: Caruel 1860; Corti 1956 [*Sommier*, s.d., FI]; Corti 1956; Orlandi & Arduini 2010; Arduini & Ercoli 2012

Nassella neesiana (Trin. & Rupr.) Barkworth
H caesp - Orig. S-America
Literature data: Peruzzi et al. 2007 [*B. Ciacchi*, *G. Lorè*, 1996, FI; *B. Ciacchi*, *G. Lorè*, 1997, PI]
Herbarium data: L. Pinzani, 2022, Herb. Pinzani
Field observations: M. D'Antraccoli, 2018; I. Arduini, 2024

Nassella trichotoma (Nees) Hack. ex Arechav.
H caesp - Orig. S-America
Literature data: Corti, 1951, 1956, Garbari 2001, Tomei et al. 2004, under the name *Stipa trichotoma* Nees; Arduini & Ercoli 2012; Lombardi 2015
Field observations: I. Arduini, 2024

Oloptum miliaceum (L.) Röser & H.R.Hamasha
H caesp - Stenomedit.-Turan.
Literature data: Caruel 1860, under the name *Milium multiflorum* Cav.
Herbarium data: J. Franzoni, 2019, Herb. Franzoni; L. Pinzani, 2019, Herb. Pinzani
Field observations: B. Pierini, 2020

Oloptum thomasii (Duby) Banfi & Galasso
H caesp - Stenomedit.
Field observations: L. Peruzzi, 2018; L. Pinzani, 2020; I. Arduini, 2024

Parapholis cylindrica (Willd.) Romero Zarco
T scap - Eurimedit.
Literature data: Caruel 1860, under the name *Lepturus cylindricus* Trin.
Herbarium data: T. Fiaschi, 2018, SIENA; G. Gestri, 2018, PI
Field observations: L. Pinzani, 2020, 2022

Parapholis filiformis (Roth) C.E.Hubb.
T scap - Medit.-Atl.
Literature data: Caruel 1860, under the name *Lepturus filiformis* Trin.; Corti 1956, under the name *Lepturus incurvus* Druce subsp. *filiformis* (Roth) A.Camus; Saggese 2016

Parapholis incurva (L.) C.E.Hubb.
T scap - Medit.-Atl.
Literature data: Caruel 1860, under the name *Lepturus incurvatus* Trin.; Corti 1956, under the name *Lepturus incurvus* (L.) Druce subsp. *incurvatus* (L.) Maire in Jah. et Maire; Sani & Tomei 2006; Petraglia 2013
Herbarium data: Partecipanti all'escursione Wikiplantbase #Parco di Migliarino - San Rossore - Massaciuccoli, 2018, PI
Field observations: partecipanti all'escursione Wikiplantbase #Parco di Migliarino - San Rossore - Massaciuccoli, 2018

Parapholis strigosa (Dumort.) C.E.Hubb.
T scap - Medit.-Atl.
Literature data: Bertacchi et al. 2009

Paspalum dilatatum Poir.

H caesp - Orig. S-America
Literature data: Coaro 1987; Lazzeri in Buono et al. 2022
Herbarium data: G. Bedini, 2016, PI; T. Fiaschi, 2018, SIENA; Partecipanti all'escursione Wikiplantbase #Parco di Migliarino - San Rossore - Massaciuccoli, 2018, PI;
Field observations: partecipanti all'escursione Wikiplantbase #Parco di Migliarino - San Rossore - Massaciuccoli, 2018; L. Peruzzi, 2019; L. Pinzani, 2020, 2022

Paspalum distichum L.
H caesp - Orig. Subcosmop.
Literature data: Savelli 1915; Montelucci 1962; Pedullà & Garbari, 2004, Bertacchi et al. 2009, under the name *P. paspaloides* (Michx.) Scribner; Lazzeri in Buono et al. 2022
Herbarium data: F. Picco, 1995, Herb. Picco; Partecipanti all'escursione Wikiplantbase #Parco di Migliarino - San Rossore - Massaciuccoli, 2018, PI
Field observations: L. Pinzani, 2021

Paspalum quadrifarium Lam.
Field observations: observed on iNaturalist in 2021
Only cultivated

Paspalum vaginatum Sw.
G rhiz - Orig. America
Literature data: Soldano 2014 [*A. Soldano*, 1983, PI]

NC **Phalaris aquatica** L.
H rhiz - Macarones.
Literature data: Baldini 1993 [*A. Fiori*, 1916, FI]

Phalaris arundinacea L. subsp. **arundinacea**
He - Circumbor.
Literature data: Caruel 1860; Corti 1956 [*M. Savelli*, s.d., FI]; Baldini 1993 [s. *coll*., 1847, PI; *P. Sav*i, 1857, PI; *Rossetti*, 1887, PI; *Pellegrini*, 1913, PI; *M. Savelli*, 1914, FI]; Garbari 2001; Pedullà & Garbari, 2004, under the name *Typhoides arundinacea* (L.) Moench; Arduini & Ercoli 2012, under the name *Phalaris rotgesii* (Husn.) Holub
Field observations: L. Pinzani, 2022.

Phalaris brachystachys Link
T scap - Stenomedit.
Literature data: Caruel 1860; Baldini 1993 [*P. Savi*, 1856, BM, JE, PI; *Mori*, 1879, MOD; *Rossetti*, 1879, PI; *Poggi*, 1880, PI; *P. Pellegrini*, 1888, PI; *M. Savelli*, 1915, FI; *s. coll.*, s.d., PI]; Saggese 2016
Field observations: J. Franzoni, 2020

NC *Phalaris canariensis* L.
T scap - Orig. Macaronesia
Literature data: Baldini 1993 [*M. Savelli*, 1916, FI]

Phalaris coerulescens Desf.
H caesp - Stenomedit.

Literature data: Caruel 1860; Corti 1956; Baldini 1993 [*F. Parlatore*, 1845, FI; *P. Savi*, 1861, BM; *O. Beccari*, 1863, FI; *Poggi*, 1886, PI; *Rossetti*, 1886, PI; *P. Pellegrini*, 1889, PI; *M. Savelli*, 1914, 1917; *A. Chiarugi, R. Corti*, 1951, FI; *O. Beccari*, 1956, FI]; Garbari 2001; Pedullà & Garbari, 2004; Saggese 2016; Lazzeri 2021
Herbarium data: T. Fiaschi, 2018, SIENA; Partecipanti all'escursione Wikiplantbase #Parco di Migliarino - San Rossore - Massaciuccoli, 2018, PI; J. Franzoni, 2020, Herb. Franzoni
Field observations: B. Pierini, 2014, 2017; partecipanti all'escursione Wikiplantbase #Parco di Migliarino - San Rossore - Massaciuccoli, 2018; L. Pinzani, 2022; H. Öhm, 2024

Phalaris paradoxa L.
T scap - Stenomedit.
Literature data: Caruel 1860; Baldini 1993 [*P. Savi*, 1840, FI; *P. Savi*, 1861, BM, K, JE, PI; *P. Fantozzi*, 1887, FI; *Poggi*, 1887, PI; *M. Savelli*, 1916, FI; *P. Pellegrini*, 1923, PI]
Herbarium data: T. Fiaschi, 2018, SIENA

Phalaris truncata Guss. ex Bertol.
H caesp - S-Medit.
Field observations: partecipanti all'escursione Wikiplantbase #Parco di Migliarino - San Rossore - Massaciuccoli, 2018

Phleum arenarium L.
T scap - Medit.-Atl.
Literature data: Caruel 1860; Corti 1956, under the name *P. arenarium* L. var. *arenarium*; Sani & Tomei 2006; Garbari 2001; Tomei et al. 2004; Bertacchi et al. 2009
Herbarium data: A. Sani, 2005, PI; G. Gestri, 2018, PI; L. Pinzani, 2020, Herb. Pinzani

NC **Phleum paniculatum** Huds.
T scap - Eurimedit.- Medit.-Turan.
Literature data: Caruel 1860, under the name *P. asperum* Jacq.

NC **Phleum pratense** L.
H caesp - C-Europ.
Literature data: Caruel 1860

Phragmites australis (Cav.) Trin. ex Steud.
He/G rhiz - Subcosmop.
Literature data: Caruel 1860, Corti 1956 [*M. Savelli*, s.d., FI]; Corti 1956, 1970, under the name *P. communis* Trin.; Coaro 1987; Garbari 2001; Tomei et al. 2004; Sani & Tomei 2006; Bertacchi et al. 2007; Bertacchi et al. 2009; Dell'Orso & Franchini 2009; Saggese 2016; Petraglia 2013; Bertacchi & Lombardi 2014b; Saggese 2016; Lazzeri 2021
Field observations: partecipanti all'escursione Wikiplantbase #Parco di Migliarino - San Rossore - Massaciuccoli, 2018; L. Peruzzi, 2019; L. Pinzani, 2021, 2022

NC **Poa angustifolia** L.
H caesp - Cosmop.
Literature data: Corti 1956, under the name *P. pratensis* L. subsp. *angustifolia* (L.) Hayek

Poa annua L.
T caesp - Cosmop.

Literature data: Caruel 1860; Corti, 1951; Gellini et al. 1986; Tomei et al. 2004; Arduini & Ercoli 2012; Saggese 2016; Bonari et al. 2019
Herbarium data: Partecipanti all'escursione Wikiplantbase #Parco di Migliarino - San Rossore - Massaciuccoli, 2018, PI
Field observations: L. Peruzzi, 2013; partecipanti all'escursione Wikiplantbase #Parco di Migliarino - San Rossore - Massaciuccoli, 2018; F. Roma-Marzio, 2019; L. Pinzani, 2021, 2022; I. Arduini, 2023

Poa bulbosa L.
H caesp - Paleotemp.
Literature data: Corti 1956, under the name *P. bulbosa* L. mut. *vivipara* Koehler; Coaro 1987; Lombardi 2015
Field observations: L. Pinzani, 2021, 2022

Poa compressa L.
H caesp - Circumbor.
Literature data: Caruel 1860; Orlandi & Arduini 2010; Arduini & Ercoli 2012

Poa nemoralis L.
H caesp - Circumbor.
Literature data: Garbari 2001; Tomei et al. 2004

Poa palustris L.
H caesp - Circumbor.
Literature data: Coaro 1987; Garbari 2001; Tomei et al. 2004

Poa pratensis L. subsp. **pratensis**
H caesp - Circumbor.
Literature data: Caruel 1860; Coaro 1987; Tomei et al. 2004; Petraglia 2013

Poa trivialis L.
H caesp - Eurasiat.
Literature data: Caruel 1860; Gellini et al. 1986; Coaro 1987; Garbari 2001; Tomei et al. 2004; Saggese 2016; Lazzeri 2021, 2022
Field observations: I. Arduini, 2024

Polypogon maritimus Willd. subsp. **maritimus**
T scap - Stenomedit.-Macarones.
Literature data: Caruel, 186; Corti 1956; Garbari 2001; Saggese 2016; Lazzeri in Buono et al. 2022

Polypogon monspeliensis (L.) Desf.
T scap - Paleosubtrop.
Literature data: Caruel 1860; Corti 1956; Coaro 1987; Garbari 2001; Pedullà & Garbari, 2004; Tomei et al. 2004; Bertacchi et al. 2007; Petraglia 2013; Bertacchi & Lombardi 2014b; Saggese 2016; Lazzeri in Buono et al. 2022
Herbarium data: A. Sani, 2005, PI; T. Fiaschi, 2018, SIENA; G. Gestri, 2018, PI; Partecipanti all'escursione Wikiplantbase #Parco di Migliarino - San Rossore - Massaciuccoli, 2018, PI
Field observations: B. Pierini, 2016; L. Pinzani, 2021

Polypogon viridis (Gouan) Breistr. subsp. **viridis**
H caesp - Paleo-Subtrop.
Literature data: Pedullà & Garbari, 2004; Lazzeri in Buono et al. 2022
Field observations: L. Pinzani, 2021, 2022

Puccinellia distans (Jacq.) Parl. subsp. **distans**
H caesp - Paleotemp.
Literature data: Caruel 1860, under the name *Glyceria distans* (L.) Wahlenb.; Bertacchi et al. 2007

Puccinellia festuciformis (Host) Parl. subsp. **festuciformis**
H caesp - Stenomedit.
Literature data: Caruel 1860, under the name *Glyceria festuciformis* Heynh.; Coaro 1987, under the name *P. palustris* (Seen.) Hayek; Petraglia 2013; Saggese 2016; Lazzeri 2021
Herbarium data: T. Fiaschi, 2018, SIENA; Partecipanti all'escursione Wikiplantbase #Parco di Migliarino - San Rossore - Massaciuccoli, 2018, PI

Rostraria cristata (L.) Tzvelev
T caesp - Paleotemp.-Subcosmop.
Literature data: Caruel 1860, under the name *Koeleria phleoides* Pers.; Lazzeri in Buono et al. 2022
Herbarium data: J. Franzoni, 2019, Herb. Franzoni
Field observations: L. Pinzani, 2021; H. Öhm, 2024

Rostraria hispida (Savi) Doğan
T scap - Stenomedit.-S-Occid.
Literature data: Coaro 1987
Assessed as LC in the Red List of the Italian Vascular Flora (Rossi et al. 2020)

Rostraria litorea (All.) Holub
T scap - Stenomedit.-Occid.
Literature data: Caruel 1860, under the name *Koeleria villosa* Pers.; Corti 1956 [*E. Levier*, s.d., FI; *C. Rossetti*, s.d., FI]
Herbarium data: T. Fiaschi, 2018, SIENA; Partecipanti all'escursione Wikiplantbase #Parco di Migliarino - San Rossore - Massaciuccoli, 2018, PI

NC **Sclerochloa dura** (L.) P.Beauv.
T scap - Eurimedit.
Literature data: Caruel 1860

Setaria italica (L.) P.Beauv. subsp. **viridis** (L.) Thell.
T scap - Subcosmop.
Literature data: Caruel 1860, Garbari 2001, under the name *S. viridis* P.Beauv.
Herbarium data: A. Sani, 2005, PI

Setaria parviflora (Poir.) Kerguélen
H caesp - Orig. S-America
Literature data: Lombardi 2015; Peruzzi et al. 2017b [*A. Sani, M. D'Antraccoli*, 2014, PI]; Lazzeri in Buono et al. 2022

Herbarium data: F. Roma-Marzio, M. D’Antraccoli, 2017, PI; J. Franzoni, 2019, Herb. Franzoni; L. Pinzani, 2022, Herb. Pinzani
Field observations: F. Roma-Marzio, 2019

Setaria pumila (Poir.) Roem. & Schult.
T scap - Subcosmop.
Literature data: Caruel 1860, under the name *S. glauca* P.Beauv.; Corti 1956 [*P. Savi*, s.d., FI]; Orlandi & Arduini 2010; Arduini & Ercoli 2012; Lazzeri in Buono et al. 2022
Herbarium data: F. Roma-Marzio, G. Bedini, 2016, PI; L. Pinzani, 2020, Herb. Pinzani

Setaria verticillata (L.) P.Beauv.
T scap - Termocosmop.
Literature data: Caruel 1860; Corti 1956, under the name *S. verticillata* (R. Br.) Pal. Beauv. subsp. *ambigua* (Guss.) Briquet
Field observations: J. Molina, 2007

Sorghum halepense (L.) Pers.
G rhiz - Orig. Subtrop.
Literature data: Caruel 1860; Saggese 2016
Field observations: L. Pinzani, 2020, 2021

NC **Sphenopus divaricatus** (Gouan) Rchb. subsp. **divaricatus**
T scap - Medit.-Turan.
Literature data: Caruel 1860, under the name *S. gouani* Trin.

Sporobolus aculeatus (L.) P.M.Peterson
T scap - Paleosubtrop.
Literature data: Caruel 1860, Coaro 1987, Garbari 2001, Tomei et al. 2004, Bertacchi et al. 2007, Petraglia 2013, Bertacchi & Lombardi 2014b, under the name *Crypsis aculeata* (L.) Aiton
Herbarium data: L. Pinzani, 2020, Herb. Pinzani

NC **Sporobolus alopecuroides** (Piller & Mitterp.) P.M.Peterson
T scap - Medit.-Turan.
Literature data: Caruel 1860, under the name *Crypsis alopecuroides* Schrad.

Sporobolus cryptandrus (Torr.) A.Gray
H caesp - Orig. N-America
Literature data: Lombardi 2015; Sani et al. in von Raab-Straube & Raus 2015 [*A. Sani, M. D’Antraccoli*, 2014, PI]

Sporobolus indicus (L.) R.Br.
H caesp - Orig. N-America
Literature data: Corti 1954 [*M. Savelli*, s.d., FI; *A. Chiarugi*, s.d., FI], Corti 1956, Garbari 2001, Tomei et al. 2004, Bertacchi et al. 2007, under the name *S. poiretii* (R. et S.) Hitchc.; Arduini & Ercoli 2012; Lombardi 2015
Herbarium data: T. Fiaschi, 2018, SIENA; J. Franzoni, 2019, Herb. Franzoni
Field observations: L. Peruzzi, 2020, 2021; L. Pinzani, 2021, 2022; I. Arduini, 2024

Sporobolus pumilus (Roth) P.M.Peterson & Saarela

G rhiz - Anfiatl.
Literature data: Garbari 2001, Sani & Tomei 2006, Bertacchi et al. 2007, Petraglia 2013, under the name *Spartina juncea* auct., non (Michx.) Willd.; Soldano 1985; Bertacchi et al. 2009, Bertacchi et al. 2010, Bertacchi & Lombardi 1993 [*A. Bertacchi, T. Lombardi*, 1992, FI, PI]; Bertacchi & Lombardi 2014a, Bertacchi & Lombardi 2014b, Saggese 2016, under the name *Spartina versicolor* Fabre

Sporobolus pungens (Schreb.) Kunth
G rhiz - Subtrop.
Literature data: Caruel 1860, Bertacchi et al. 2007, Bertacchi et al. 2009, under the name *S. virginicus* (L.) Kunth
Herbarium data: D. Ciccarelli, 2011, PI

NC **Sporobolus schoenoides** (L.) P.M.Peterson
T scap - Paleosubtrop.
Literature data: Caruel 1860, under the name *Crypsis schoenoides* Lam.; Tomei et al. 1986 [*P. Savi*, s.d., 1840, PI; *B. Puccinelli*, s.d., PI; *F. Parlatore*, 1855, FI; *Poggi*, 1888, PI; *M. Savelli*, 1917, 1918, FI]

NC **Stipa juncea** L.
H caesp - Senomedit.- N-Occid.
Literature data: Arrigoni 2018 [s. *coll.*, s.d., TO]

Stipellula capensis (Thunb.) Röser & H.R.Hamasha
T scap - Stenomedit.
Literature data: Caruel 1860, under the name *Stipa tortilis* Desf.; Peruzzi et al. 2020 [*F. Roma-Marzio, L. Peruzzi*, 2019, PI]

Thinopyrum acutum (DC.) Banfi
H caesp - Medit.
Literature data: Garbari 2001; Pedullà & Garbari, 2004; Sani & Tomei 2006, Petraglia 2013, under the name *Agropyron pungens* (Pers.) R. et S.; Saggese 2016, Lazzeri 2021, 2022, under the name *Elytrigia atherica* (Link) Kerguélen
Herbarium data: T. Fiaschi, 2018, SIENA; Partecipanti all'escursione Wikiplantbase #Parco di Migliarino - San Rossore - Massaciuccoli, 2018, PI

Thinopyrum junceum (L.) Á.Löve
G rhiz - Eurimedit.
Literature data: Caruel 1860, under the name *Triticum junceum* L.; Corti 1956, Sani & Tomei 2006, under the name *Agropyron junceum* (L.) Pal. Beauv. subsp. *mediterraneum*; Bertacchi et al. 2009, Bertacchi & Lombardi 2014a, Bertacchi & Lombardi 2014b, under the name *Elymus farctus* (Viv.) Runemark ex Melderis
Herbarium data: Ciccarelli & M. Sammartino, 2009, PI; Partecipanti all'escursione Wikiplantbase #Parco di Migliarino - San Rossore - Massaciuccoli, 2018, PI

Thinopyrum obtusiflorum (DC.) Banfi
G rhiz - Avv.
Literature data: Saggese 2016, under the name *Elytrigia elongata* (Host) Nevski
Herbarium data: S. Gerace, 2018, Herb. Gerace

Tragus racemosus (L.) All.
T scap - Cosmop.
Herbarium data: J. Franzoni, 2019, Herb. Franzoni
Field observations: B. Pierini, 2024

Tripidium ravennae (L.) H.Scholz subsp. **ravennae**
H caesp - Medit.-Turan.
Literature data: Caruel 1860, Baroni 1897-1908, under the name *Saccharum ravennae* Murr.; Corti 1956, Garbari 2001, Tomei et al. 2004, Sani & Tomei 2006, Bertacchi et al. 2007, Bertacchi et al. 2009, Dell'Orso & Franchini 2009, Bertacchi & Lombardi 2014b, under the name *Erianthus ravennae* (L.) Pal. Beauv.
Field observations: A. Mo, 2024

Triticum aestivum L. subsp. *Aestivum*
Field observations: observed on iNaturalist in 2022
Only cultivated

NC **Trisetaria aurea** (Ten.) Pignatti ex Kerguélen
T scap - Stenomedit.-Orient.
Literature data: Baroni 1897-1908, under the name *Trisetum aureum* Ten..

Trisetaria panicea (Lam.) Paunero
T scap - Stenomedit.-Occid.-Macarones.
Literature data: Caruel 1860, under the name *Trisetum neglectum* Roem. et Schult.; Corti, 1954, under the name *Trisetum paniceum* Pers.; Corti 1956 [*P. Savi*, s.d., FI]; Garbari 2001; Arduini & Ercoli 2012; Lombardi 2015; Lazzeri in Buono et al. 2022
Herbarium data: T. Fiaschi, 2018, SIENA; Partecipanti all'escursione Wikiplantbase #Parco di Migliarino - San Rossore - Massaciuccoli, 2018, PI
Field observations: L. Peruzzi, 2021; L. Pinzani, 2022

NC **Trisetaria segetum** (Savi) Soldano
T scap - SW-Medit.
Literature data: Caruel 1860, under the name *Trisetum parviflorum* Pers.
The basyonim of this species, *Festuca segetum* Savi, was described by Gaetano Savi (1798) for the wheat fields surrounding Pisa (Peruzzi et al., 2009).

NC **Triticum neglectum** (Req. ex Bertol.) Greuter
T scap - Medit.-Turan.
Literature data: Caruel 1860, under the name *Aegilops ovata* L., *Aegilops triaristata* Willd.

Ceratophyllaceae

Ceratophyllum demersum L.
I rad - Subcosmop.
Literature data: Caruel 1860; Corti 1956; Garbari 2001

Papaveraceae

Chelidonium majus L.
H scap - Circumbor.
Field observations: B. Pierini, 2014, 2016, 2017; F. Roma-Marzio, 2017; L. Pinzani, 2021, 2022

Fumaria bastardii Boreau
T scap - Subatl.
Herbarium data: G. Gestri, 2018, PI; L. Pinzani, 2020, Herb. Pinzani

Fumaria capreolata L. subsp. **capreolata**
T scap - Eurimedit.
Literature data: Caruel 1860; Lazzeri 2021
Herbarium data: F. Roma-Marzio, L. Peruzzi, 2019, PI
Field observations: B. Pierini, 2014; L. Pinzani, 2021

D **Fumaria muralis** Sond. ex W.D.J.Koch subsp. **muralis**
Literature data: Corti 1956

Fumaria officinalis L. subsp. **officinalis**
T scap - Subcosmop.
Literature data: Coaro 1987; Bertacchi et al. 2009
Herbarium data: L. Pinzani, 2020, Herb. Pinzani
Field observations: B. Pierini, 2014

Fumaria vaillantii Loisel.
T scap - Medit.-Turan.
Field observations: observed on iNaturalist in 2024

Glaucium flavum Crantz
H scap - Eurimedit.-W-Europ.
Literature data: Caruel 1860; Corti 1956 [*P. Savi*, s.d., FI]; Corti 1956; Garbari 2001; Sani & Tomei 2006; Bertacchi et al. 2009; Bertacchi et al. 2010; Bertacchi & Lombardi 2014a
Herbarium data: L. Pinzani, 2021, Herb. Pinzani

Papaver dubium L.
T scap - E-Medit.-Turan.
Field observations: partecipanti all'escursione Wikiplantbase #Parco di Migliarino - San Rossore - Massaciuccoli, 2018

Papaver rhoeas L. subsp. **rhoeas**
T scap - Eurimedit.
Literature data: Caruel 1860; Corti 1956, under the name *P. rhoeas* L. var. *strigosum* Bonning; Coaro 1987; Pedullà & Garbari, 2004; ; Saggese 2016; Bonari et al. 2019
Herbarium data: M.L. Pedullà, 2000, PI; Partecipanti all'escursione Wikiplantbase #Parco di Migliarino - San Rossore - Massaciuccoli, 2018, PI
Field observations: partecipanti all'escursione Wikiplantbase #Parco di Migliarino - San Rossore - Massaciuccoli, 2018; M. D'Antraccoli, 2019; L. Pinzani, 2022

Papaver setigerum DC.
T scap - W-Medit.

Literature data: Caruel 1860; Pedullà & Garbari, 2004; Peruzzi et al. 2019 [*M. D'Antraccoli*, 2019, PI]
Herbarium data: L. Pinzani, 2021, Herb. Pinzani

Roemeria sicula (Guss.) Galasso, Banfi, L.Sáez & Bartolucci
T scap - Medit.-Turan.
Literature data: Caruel 1860, under the name *Papaver hybridum* L.
Field observations: partecipanti all'escursione Wikiplantbase #Parco di Migliarino - San Rossore - Massaciuccoli, 2018

Berberidaceae

Nandina domestica Thunb.
Field observations: observed on iNaturalist in 2024
Only cultivated

Ranunculaceae

Adonis annua L.
T scap - Stenomedit.-Sett.
Literature data: Caruel 1860, under the name *A. autumnalis* L.; Steinberg 1971, under the name *A. annua* L. subsp. *cupaniana* (Guss.) C.H.Steinb. [*A. Tassi*, 1842, FI; *s. coll.*, 1862, P, PI; *s. coll.*, 1863, FI, TO; *G. Arcangeli*, 1865, PI; *Danielli*, 1876, PI; *Van Heurek*, 1868, P; *Poggi*, 1885, PI; *P. Fantozzi*, 1887, FI; *P. Pellegrini*, 1887, PI; *Poggi*, *Rossetti*, 1887, TO; *Rossetti*, *P. Pellegrini*, 1887, PAL; *P. Pellegrini*, 1888, PI; *E. Barsali*, 1900, PI]
Field observations: L. Pinzani, 2022; A. Mo, 2023; J. Franzoni, A. Giacò, 2023; A. Mo, A. Giacò, 2023

Anemone hortensis L. subsp. **hortensis**
G bulb - N-Medit.
Literature data: Coaro 1987; Garbari 2001
Field observations: B. Pierini, 2014

Clematis flammula L.
P lian - Eurimedit.
Literature data: Caruel 1860; Corti 1956 [*O. Beccari*, s.d., FI]; Corti 1956, under the name *C. flammula* L. var. *maritima* (L.) DC.; Coaro 1987 [*E. Coaro*, 1984, PI; *G. Pistolesi*, *E. Coaro*, 1984, PI]; Garbari 2001; Tomei et al. 2004; Arduini & Ercoli 2012; Lombardi 2015; Saggese 2016; Bonari et al. 2019
Herbarium data: O. Beccari, 1862, FI; S. Sommier, 1899, FI; M. Savelli, 1915, 1916, FI; R. Poli, 1989, PI
Field observations: partecipanti all'escursione Wikiplantbase #Parco di Migliarino - San Rossore - Massaciuccoli, 2018; L. Sandroni, 2021; L. Pinzani, 2022

Clematis vitalba L.
P lian - Europ.-Caucas.
Literature data: Caruel 1860; Corti 1956; Gellini et al. 1986; Coaro 1987 [*G. Pistolesi*, *E. Coaro*, 1977, PI; *E. Coaro*, 1984, PI]; Garbari 2001 [*B. Ciacchi*, *G. Lorè*, 1996, PI]; Tomei et al. 2004; Bertacchi & Lombardi 2016; Lazzeri in Buono et al. 2022

Herbarium data: M.L. Pedullà, 1999, PI
Field observations: L. Peruzzi, 2017; partecipanti all'escursione Wikiplantbase #Parco di Migliarino - San Rossore - Massaciuccoli, 2018; L. Pinzani, 2020, 2021, 2022; I. Arduini, 2023

Delphinium consolida L. subsp. **consolida**
T scap - Eurimedit.
Field observations: L. Peruzzi, 2015

Delphinium hispanicum Willk. ex Costa
T scap - Orig. Eurasiat.
Literature data: Pedullà & Garbari, 2004, under the name *Consolida orientalis* (Gay) Schrödinger, [*M.L. Pedullà*, 2000, PI]

Ficaria verna Huds. subsp. **ficariiformis** (F.W.Schultz) B.Walln.
G bulb/H scap - Eurimedit.
Field observations: B. Pierini, 2014

Ficaria verna Huds. subsp. **fertilis** (Lawalrée ex Laegaard) Stace
G bulb - W-Europ.
Field observations: observed on iNaturalist in 2021

Ficaria verna Huds. subsp. **verna**
G bulb/H scap - Eurasiat.
Literature data: Coaro 1987, Garbari 2001, under the name *Ranunculus ficaria* L. subsp. *ficaria*
Field observations: L. Pinzani, 2022; I. Arduini, 2024

NC **Nigella damascena** L.
T scap - Eurimedit.
Literature data: Caruel 1860

NC **Ranunculus acris** L. subsp. **acris**
H scap - Subcosmop.
Literature data: Caruel 1860

Ranunculus arvensis L.
T scap - Paleotemp.
Literature data: Pedullà & Garbari, 2004

Ranunculus baudotii Godr.
I rad - Medit.-Atl.
Literature data: Baroni 1897-1908, under the names *R. aquatilis* L. var. *peltatus* Schrank and *R. aquatilis* var. *succulentus* Koch; Coaro 1987, under the name *R. peltatus* Schrank; Petraglia 2013, under the name *R. peltatus* subsp. *baudotii*
Herbarium data: L. Pinzani, A. Giacò, J. Franzoni, 2021, Herb. Pinzani
Assessed as NT in the Red List of the Italian Vascular Flora (Rossi et al. 2020)

Ranunculus bulbosus L.
H scap - Eurasiat.

Literature data: Caruel 1860; Corti 1956, under the name *R. bulbosus* L. forma *bulbifer* (Jord.) Rouy et Fouc.; Garbari 2001
Field observations: B. Pierini, 2014; L. Pinzani, 2021, 2022; I. Arduini, 2024; H. Öhm, 2024

Ranunculus flammula L.
H scap - Eurasiat.
Literature data: Caruel 1860; Corti 1956 [*A. Fiori*, s.d., FI]; Gellini et al. 1986; Tomei et al. 2004; Lastrucci et al. 2008 [*s. coll.*, 1862, PI; *A. Chiarugi, R. Corti, R. Negri*, 1951, FI]; Lombardi 2015
Field observations: I. Arduini, 2024

Ranunculus lanuginosus L.
H scap - Europ.-Caucas.
Literature data: Coaro 1987
Field observations: B. Pierini, 2014; partecipanti all'escursione Wikiplantbase #Parco di Migliarino - San Rossore - Massaciuccoli, 2018

Ranunculus lingua L.
H scap/He - Eurasiat.
Literature data: Caruel 1860; Baroni 1897-1908; Garbari 2001; Tomei et al. 2004

Ranunculus muricatus L.
T scap - Eurimedit.
Literature data: Saggese 2016
Herbarium data: T. Fiaschi, 2018, SIENA; L. Pinzani, 2020, 2021, Herb. Pinzani
Field observations: B. Pierini, 2015, 2020; L. Pinzani, 2021, 2022

Ranunculus ophioglossifolius Vill.
T scap - Eurimedit.
Literature data: Caruel 1860; Baroni 1897-1908; Corti 1956 [*P. Fantozzi*, FI; *A. Fiori*, FI]; Gellini et al. 1986; Coaro 1987; Tomei et al. 2004
Assessed as VU in the Red List of the Italian Vascular Flora (Rossi et al. 2020)

Ranunculus parviflorus L.
T scap - Medit.-Atl.
Literature data: Caruel 1860; Corti 1956 [*P. Fantozzi*, FI]; Corti 1956; Coaro 1987; Garbari 2001; Sani & Tomei 2006
Herbarium data: A. Sani, 2005, PI; F. Roma-Marzio, L. Peruzzi, 2019, PI
Field observations: B. Pierini, 2015; L. Pinzani, 2021, 2022; J. Franzoni, 2023; A. Mo, 2023; I. Arduini, 2023

Ranunculus repens L.
H rept - Paleotemp.-Subcosmop.
Literature data: Corti 1956, under the name *R. repens* L. var. *glabratus* DC.; Gellini et al. 1986; Coaro 1987; Tomei et al. 2004; Bertacchi & Lombardi 2016
Field observations: L. Pinzani, 2022; I. Arduini, 2024

D **Ranunculus saniculifolius** Viv.
I rad - Stenomedit.
Literature data: Baroni 1897-1908, under the name *R. aquatilis* L. var. *triphyllos* Vallr.

Ranunculus sardous Crantz
T scap - Eurimedit.
Literature data: Caruel 1860, under the name *R. philonotis* Retz.; Corti 1956, under the name *R. sardous* Crantz subsp. *philonotis* Briquet; Coaro 1987; Garbari 2001; Tomei et al. 2004; Petraglia 2013; Lombardi 2015; Saggese 2016; Bonari et al. 2019; Lazzeri 2021, 2022
Herbarium data: A. Sani, 2005, PI; Partecipanti all'escursione Wikiplantbase #Parco di Migliarino - San Rossore - Massaciuccoli, 2018, PI
Field observations: partecipanti all'escursione Wikiplantbase #Parco di Migliarino - San Rossore - Massaciuccoli, 2018

Ranunculus sceleratus L.
T scap - Paleotemp.
Herbarium data: J. Franzoni, 2019, Herb. Franzoni; L. Pinzani, 2021, Herb. Pinzani
Field observations: B. Pierini, 2020, 2023; L. Pinzani, 2021, 2022; I. Arduini, 2023

Ranunculus trichophyllus Chaix
I rad - Europ.
Literature data: Caruel 1860; Corti 1956 [*P. Fantozzi*, s.d., FI]; Corti 1956; Coaro 1987; Garbari 2001

Ranunculus velutinus Ten.
H scap - N-Medit.
Literature data: Caruel 1860; Corti 1956; Coaro 1987; Garbari 2001
Field observations: L. Pinzani, 2020, 2022; I. Arduini, 2024

Thalictrum flavum L.
H scap - Eurasiat.
Literature data: Caruel 1860; Corti 1956, under the name *T. flavum* L. subsp. *heterophyllum* (Lej.) Rouy et Fouc.; Coaro 1987; Garbari 2001; Bertacchi & Lombardi 2016

Thalictrum lucidum L.
H scap - SE-Europ.
Field observations: observed on iNaturalist in 2024

Nelumbonaceae

Nelumbo nucifera Gaertn.
I rad - Orig. Paleotrop.
Literature data: Peruzzi in Selmi 2021
Field observations: L. Peruzzi, 2023

Platanaceae

Platanus hispanica Mill. ex Münchh.
P scap - Orig. Eurimedit.
Literature data: Corti 1956, under the name *P. orientalis* L.; Garbari 2001, Pedullà & Garbari, 2004, under the name *P. hybrida* Brot.

Buxaceae

Buxus sempervirens L.
Field observations: observed on iNaturalist in 2024
Only cultivated

Altingiaceae

Liquidambar styraciflua L.
Field observations: observed on iNaturalist in 2020, 2024
Only cultivated

Grossulariaceae

NC *Ribes rubrum* L.
Literature data: Corti 1956 [*A. Chiarugi, R. Corti*, 1951, FI]
Only formerly cultivated in the study area.

Saxifragaceae

Saxifraga tridactylites L.
T scap - Eurimedit.
Literature data: Caruel 1860; Corti 1956, under the name *S. tridactylites* L. subsp. *eutridactylites* Engl. et Irmsch.; Garbari 2001
Herbarium data: L. Pinzani, 2020, Herb. Pinzani
Field observations: B. Pierini, 2015; F. Roma-Marzio, 2019; L. Pinzani, 2021, 2022

Crassulaceae

Crassula tillaea Lest.-Garl.
T scap - Subatl.-Submedit.
Literature data: Caruel 1860; Baroni 1897-1908, Corti 1956 [*P. Fantozzi*, FI], Corti 1956, under the name *Tillaea muscosa* L.
Herbarium data: L. Pinzani, 2020, Herb. Pinzani

Kalanchoë ×houghtonii D.B.Ward
Field observations: F. Roma-Marzio, 2015

NC **Petrosedum rupestre** (L.) P.V.Heath
Ch succ - W-Centroeurop.
Literature data: Caruel 1860, under the name *Sedum rupestre* L.

NC **Sedum album** L. subsp. **album**
Ch succ - Eurimedit.
Literature data: Caruel 1860

NC **Sedum cepaea** L.
T scap - Submedit.
Literature data: Caruel 1860; Corti 1956

Sedum dasyphyllum L. subsp. **dasyphyllum**
Ch succ - Eurimedit.
Literature data: Caruel 1860
Field observations: L. Pinzani, 2021

Sedum hispanicum L.
T scap - SE-Europ.-Pont.
Field observations: B. Pierini, 2014

Sedum palmeri S.Watson
NP - Orig. C-America
Field observations: L. Pinzani, 2022

NC **Sedum rubens** L.
T scap - Eurimedit.-Subatl.
Literature data: Baroni 1897-1908

Sedum sexangulare L.
Ch succ - Centroeurop.
Literature data: Caruel 1860, Corti 1956 [*Picciuoli*, FI], Corti 1956, under the name *S. boloniense* Lois.; Corti, 1951, under the name *S. mite* Gilib.; Garbari 2001; Tomei et al. 2004

Umbilicus rupestris (Salisb.) Dandy
G bulb - Medit.-Atl.
Literature data: Caruel 1860; Pedullà & Garbari, 2004
Field observations: L. Pinzani, 2020, 2021; F. Roma-Marzio, 2021

Haloragaceae

Myriophyllum spicatum L.
I rad - Subcosmop.
Literature data: Caruel 1860
Field observations: L. Peruzzi, 2018

NC **Myriophyllum verticillatum** L.
I rad - Circumbor.
Literature data: Caruel 1860

Vitaceae

Parthenocissus inserta (A.Kern.) Fritsch
P lian - Orig. N-America
Literature data: Corti 1956, under the name *Parthenocissus quinquefolia* (L.) Planch.; Galasso et al. 2022 [*Partecipanti all'escursione Wikiplantbase #Migliarino-San Rossore-Massaciuccoli*, 2018, PI; *F. Roma-Marzio*, 2022, PI]
Herbarium data: T. Fiaschi, 2018, SIENA; F. Roma-Marzio, 2022, PI

Vitis vinifera L.

P lian - N-Medit.
Literature data: Corti 1956, under the name *V. vinifera* L. var. *silvestris* (C. C. Gmel) Beck; Gellini et al. 1986; Coaro 1987; Garbari 2001; Tomei et al. 2004; Bertacchi et al. 2010; Lazzeri in Buono et al. 2022
Herbarium data: F. Roma-Marzio, M. D'Antraccoli, 2017, PI
Often only cultivated in the study area.

Vitis ×instabilis Ardenghi, Galasso, Banfi & Lastrucci
(*Vitis riparia* × *V. rupestris*)
Herbarium data: L. Pinzani, 2020, Herb. Pinzani

Vitis ×koberi Ardenghi, Galasso, Banfi & Lastrucci
(*Vitis berlandieri* × *V. riparia*)
Field observations: B. Pierini, 2015

Zygophyllaceae

Tribulus terrestris L.
T rept - Cosmop.
Literature data: Caruel 1860; Corti 1956 [*P. Savi*, s.d., FI; *A. Archbald*, s.d., FI; *Levier*, s.d., FI; *Amidei*, s.d., FI]; Pedullà & Garbari, 2004; Bertacchi et al. 2009
Herbarium data: T. Fiaschi, 2018, SIENA; Partecipanti all'escursione Wikiplantbase #Parco di Migliarino - San Rossore - Massaciuccoli, 2018, PI; L. Pinzani, 2020, Herb. Pinzani
Field observations: B. Pierini, 2014; F. Roma-Marzio, 2014; L. Pinzani, 2021

Fabaceae

Albizia julibrissin Durazz.
Field observations: observed on iNaturalist in 2024
Only cultivated

Amorpha fruticosa L.
P caesp - Orig. N-America
Literature data: Baroni 1897-1908; Montelucci 1962; Sani & Tomei 2006
Herbarium data: M. Savelli, 1914, 1916, 1917
Field observations: L. Peruzzi, 2013; 2019; M. D'Antraccoli, 2019

NC **Astragalus glycyphyllos** L.
H rept - Europ.-Sudsib.
Literature data: Caruel 1860

NC **Astragalus hamosus** L.
T scap - Medit.-Turan.
Literature data: Caruel 1860

Astragalus sesameus L.
T scap - Stenomedit.
Literature data: Coaro 1987

Bituminaria bituminosa (L.) C.H.Stirt.
H scap - Euri-Medit.
Field observations: observed on iNaturalist in 2024

Ceratonia siliqua L.
Field observations: I. Arduini, 2023
Only cultivated.

Cercis siliquastrum L. subsp. *siliquastrum*
Field observations: observed on iNaturalist in 2023
Only cultivated

NC **Coronilla scorpioides** (L.) W.D.J.Koch
T scap - Eurimedit.
Literature data: Caruel 1860

NC **Cytisus scoparius** (L.) Link subsp. **scoparius**
P caesp - Centromedit.
Literature data: Caruel 1860, under the name *Sarothamnus vulgaris* Wimm.

Cytisus villosus Pourr.
P caesp - W-Medit.
Literature data: Caruel 1860, under the name *C. triflorus* L'Her.; Garbari 2001

Emerus major Mill. subsp. **major**
NP - Centroeurop.-S-Europ.
Literature data: Baroni 1897-1908, under the name *Coronilla emerus* L. var. *emeroides* Boiss. et Sprun.; Coaro 1987 [*G. Pistolesi, E. Coaro*, 1977, PI; *G. Pistolesi, E. Coaro*, 1984, PI], under the name *Coronilla emerus* L. subsp. *emerus*
Herbarium data: L. Pinzani, 2020, Herb. Pinzani

Ervilia hirsuta (L.) Opiz
T scap - Paleotemp.-Subcosmop.
Literature data: Caruel 1860, under the name *Ervum hirsutum* L.; Corti, 1954, 1956, under the name *Vicia hirsuta* (L.) S. F. Gray var. *eriocarpa* (Gr. et Godr.) Rouy; Corti 1956 [*G. Savi*, s.d., FI]; Garbari 2001, under the name *Vicia hirsuta* (L.) Gray; Lazzeri in Buono et al. 2022
Herbarium data: L. Pinzani, A. Giacò, J. Franzoni, 2021, Herb. Pinzani

Ervilia sativa Link
T scap - Orig. E-Medit.
Literature data: Caruel 1860, under the name *Ervum ervilia* L.; Garbari 2001, under the name *Vicia ervilia* (L.) Willd.

Ervilia sylvatica (L.) Schur
H scap - Eurosiber.
Literature data: Caruel 1860, under the name *Vicia sylvatica* L.; Corti, 1954, under the name *Vicia hirsuta* (L.) S. F. Gray var. *eriocarpa* (Gr. et Godr.) Rouy

Ervum gracile DC.

T scap - Eurimedit.
Literature data: Caruel 1860; Baroni 1897-1908; Corti, 1954, 1956, Garbari 2001, under the name *Vicia tenuissima* (M. Bieb.) Schinz & Thell; Lombardi 2015; Saggese 2016, under the name *Vicia parviflora* Cav.; Lazzeri 2021, 2022
Herbarium data: G. Gestri, 2018, PI; L. Pinzani, 2021, 2022, Herb. Pinzani
Field observations: B. Pierini, 2017; L. Pinzani, 2022

Ervum tetraspermum L.
T scap - Paleotemp.
Literature data: Caruel 1860; Corti 1956 [*P. Savi*, s.d., FI; *Grilli*, s.d., FI]; Coaro 1987, under the name *Vicia tetrasperma* (L.) Schreb.
Field observations: partecipanti all'escursione Wikiplantbase #Parco di Migliarino - San Rossore - Massaciuccoli, 2018

Galega officinalis L.
H scap - E-Europ.-Pont.
Literature data: Caruel 1860; Garbari 2001
Field observations: B. Pierini, 2017; L. Pinzani, 2021

Genista germanica L.
Ch suffr (NP) - Centroeurop.
Literature data: Coaro 1987 [*G. Pistolesi, E. Coaro*, 1977, 1984, PI]

NC **Genista monspessulana** (L.) L.A.S.Johnson
P caesp - Stenomedit.
Literature data: Caruel 1860, under the name *G. candicans* L.

Genista tinctoria L.
Ch suffr - Eurasiat.
Literature data: Coaro 1987

Gleditsia triacanthos L.
P caesp/P scap - Orig. N-America
Herbarium data: M. Savelli, 1915
Field observations: I. Arduini, 2023

Glycyrrhiza glabra L.
G rhiz - Stenomedit.-W-Asiat.
Literature data: Garbari 2001

Lathyrus annuus L.
T scap - Eurimedit.
Literature data: Caruel 1860; Coulot & Rabaute 2016 [*Schoenefeld*, 1844, MPU]
Field observations: B. Pierini, 2015; L. Pinzani, 2022; H. Öhm, 2024

Lathyrus aphaca L. subsp. **aphaca**
T scap - Eurimedit.
Literature data: Caruel 1860; Coaro 1987; Lazzeri 2021

Herbarium data: Partecipanti all'escursione Wikiplantbase #Parco di Migliarino - San Rossore - Massaciuccoli, 2018, PI
Field observations: partecipanti all'escursione Wikiplantbase #Parco di Migliarino - San Rossore - Massaciuccoli, 2018

Lathyrus cicera L.
T scap - Eurimedit.
Literature data: Garbari 2001; Tomei et al. 2004; Lombardi 2015

Lathyrus clymenum L.
T scap - Stenomedit.
Literature data: Caruel 1860; Saggese 2016

Lathyrus hirsutus L.
T scap - Eurimedit.
Literature data: Caruel 1860; Baroni 1897-1908; Corti 1956 [*Beccari*, s.d., FI; *Poggi*, *Rossetti*, s.d., FI]; Corti 1956; Coaro 1987; Pedullà & Garbari, 2004; Lazzeri 2021
Herbarium data: G. Gestri, 2018, PI
Field observations: B. Pierini, 2017, 2020; partecipanti all'escursione Wikiplantbase #Parco di Migliarino - San Rossore - Massaciuccoli, 2018; L. Pinzani, 2020, 2022

Lathyrus latifolius L.
H scand - S-Europ.
Literature data: Coaro 1987, under the name *L. sylvestris* L.

Lathyrus nissolia L.
T scap - Eurimedit.
Literature data: Sani & Tomei in Peruzzi et al. 2009 [*A. Sani*, *P.E. Tomei*, 2009, PI]

Lathyrus ochrus (L.) DC.
T scap - Stenomedit.
Literature data: Caruel 1860; Garbari 2001; Pedullà & Garbari, 2004; Coulot & Rabaute 2016 [*P. Savi*, 1861, MPU]
Herbarium data: J. Franzoni, 2020, Herb. Franzoni
Field observations: B. Pierini, 2017; L. Pinzani, 2019, 2022

Lathyrus oleraceus Lam. subsp. **biflorus** (Raf.) H.Schaef.
T scap - Medit.-Turan.
Field observations: observed on iNaturalist in 2022, under the name *Pisum sativum* L.

NC **Lathyrus palustris** L.
H scap - Circumbor.
Literature data: Caruel 1860

Lathyrus pratensis L. subsp. **pratensis**
H scap - Paleotemp.
Literature data: Caruel 1860; Coaro 1987

NC *Lathyrus sativus* L.

Literature data: Caruel 1860

Lathyrus sphaericus Retz.
T scap - Eurimedit.
Literature data: Caruel 1860; Corti 1956 [*P. Savi*, s.d., FI; *A. Tassi*, s.d., FI]; Corti 1956; Coaro 1987; Garbari 2001; Bonari et al. 2019
Field observations: L. Pinzani, 2020

D **Lathyrus sylvestris** L. subsp. **sylvestris**
Literature data: Caruel 1860, under the name *L. sylvestris* L.
The record of this species is doubtful, and could refer to *Lathyrus latifolius* L.

NC **Lathyrus tuberosus** L.
H scap - Paleotemp.
Literature data: Caruel 1860

Lotus angustissimus L.
T scap - Eurimedit.
Literature data: Caruel 1860; Corti 1956 [*P. Savi*, s.d., FI; *Roberti*, s.d., FI]; Corti 1956; Coaro 1987; Arduini & Ercoli 2012; Lombardi 2015; Lazzeri in Buono et al. 2022
Herbarium data: G. Gestri, 2018, PI; Partecipanti all'escursione Wikiplantbase #Parco di Migliarino - San Rossore - Massaciuccoli, 2018, PI; L. Pinzani, 2021, Herb. Pinzani

Lotus conimbricensis Brot.
T scap - W-Medit.
Literature data: Garbari 2001

Lotus corniculatus L. subsp. **corniculatus**
H scap - Paleotemp.
Literature data: Caruel 1860; Coaro 1987; Garbari 2001; Petraglia 2013; Saggese 2016; Lazzeri 2021, 2022
Herbarium data: Partecipanti all'escursione Wikiplantbase #Parco di Migliarino - San Rossore - Massaciuccoli, 2018, PI

D **Lotus corniculatus** L. subsp. **preslii** (Ten.) P.Fourn.
H scap - Eurimedit.
Literature data: Coaro 1987, under the name *L. preslii* Ten.

Lotus dorycnium L. subsp. **dorycnium**
Ch suffr - SE-Europ.
Literature data: Coaro 1987, Garbari 2001, under the name *Dorycnium pentaphyllum* Scop. subsp. *pentaphyllum*

Lotus hirsutus L.
Ch suffr - Eurimedit.
Literature data: Caruel 1860, Coaro 1987, under the name *Dorycnium hirsutum* (L.) Ser.; Corti 1956, under the name *Dorycnium hirsutum* (L.) Ser. in DC. var. *hirtum* Rikli; Tomei et al. 2004, Sani & Tomei 2006 [*A. Sani*, 2005, PI], Bertacchi et al. 2009, Bertacchi et al. 2010, Bertacchi & Lombardi 2014a, under the name *Dorycnium hirsutum* (L.) Ser.

Herbarium data: D. Ciccarelli, 2011, PI; Partecipanti all'escursione Wikiplantbase #Parco di Migliarino - San Rossore - Massaciuccoli, 2018, PI
Field observations: L. Pinzani, 2019

Lotus hispidus DC.
T scap - W-Medit.
Literature data: Caruel 1860; Corti 1956 [*P. Savi*, s.d., FI]; Arduini & Ercoli 2012; Lazzeri in Buono et al. 2022

Lotus maritimus L.
H scap - Medit.-Pont.
Literature data: Caruel 1860, under the name *Tetragonolobus siliquosus* Roth; Corti 1956 [*F. Parlatore*, s.d., FI; *Picciuoli*, s.d., FI; *A. Tassi*, s.d., FI]; Corti 1956, under the name *L. siliquosus* L. f. *genuinus* Gren. et Godr.; Coaro 1987, Garbari 2001, under the name *Tetragonolobus maritimus* (L.) Roth; Petraglia 2013; Saggese 2016
Herbarium data: G. Gestri, 2018, PI; Partecipanti all'escursione Wikiplantbase #Parco di Migliarino - San Rossore - Massaciuccoli, 2018, PI

Lotus ornithopodioides L.
T scap - Stenomedit.
Literature data: Garbari 2001; Tomei et al. 2004

Lotus pedunculatus Cav.
H scap - Paleotemp.
Literature data: Tomei et al. 2004, under the name *L. uliginosus* Schk.

Lotus rectus L.
Ch suffr/H scap - Stenomedit.
Literature data: Lazzeri in Buono et al. 2022

Lotus tenuis Waldst. & Kit. ex Willd.
H scap - Paleotemp.
Literature data: Corti 1956, under the name *L. corniculatus* L. var. *tenuifolius* L.; Coaro 1987; Sani & Tomei 2006 [*A. Sani*, 2005, PI]; Arduini & Ercoli 2012; Petraglia 2013; Saggese 2016; Lazzeri in Buono et al. 2022
Herbarium data: L. Pinzani, 2020, Herb. Pinzani

Lupinus angustifolius L.
T scap - Stenomedit.
Literature data: Caruel 1860; Baroni 1897-1908; Corti 1956 [*P. Savi*, s.d., FI; *O. Beccari*, s.d., FI; *P. Fantozzi*, s.d., FI]; Corti 1956; Coaro 1987, under the name *L. angustifolius* L. subsp. *reticulatus* (Desv.) Coutinho; Garbari 2001; Coulot & Rabaute 2016 [s. *coll.*, 1836, MPU; *P. Savi*, s.d., PO]
Herbarium data: L. Pinzani, A. Giacò, J. Franzoni, 2021, Herb. Pinzani
Field observations: partecipanti all'escursione Wikiplantbase #Parco di Migliarino - San Rossore - Massaciuccoli, 2018

Medicago arabica (L.) Huds.
T scap - Eurimedit.
Literature data: Caruel 1860, under the name *M. maculata* Willd.; Garbari 2001

Field observations: B. Pierini, 2016, 2017; L. Pinzani, 2021, 2022; F. Roma-Marzio, 2021; I. Arduini, 2023; H. Öhm, 2024

Medicago arborea L.
Field observations: observed on iNaturalist in 2022
Only cultivated

NC **Medicago ciliaris** (L.) All.
T scap - S-Medit.-Macarones.
Literature data: Caruel 1860

Medicago intertexta (L.) Mill.
T scap - W-Medit.-Macarones.
Literature data: Caruel 1860, under the name *M. echinus* Dec.; Coulot & Rabaute 2013 [*Roberti*, 1877, MPU]
Herbarium data: L. Pinzani, 2022, Herb. Pinzani

Medicago littoralis Rohde ex Loisel.
T scap - Eurimedit.
Literature data: Caruel 1860; Corti 1956 [*O. Beccari*, s.d., FI; *Levier*, s.d., FI; *C. Rossetti*, s.d., FI; *A. Tassi*, s.d., FI]; Corti 1956, under the name *M. litoralis* Rhode subsp. *longiseta* (DC.) Fiori var. *arenaria* (Ten.) Fiori; Garbari 2001; Bertacchi et al. 2009; Bertacchi et al. 2010; Bertacchi & Lombardi 2014a; Bertacchi & Lombardi 2014b
Herbarium data: F. Roma-Marzio, 2012, PI; L. Pinzani, 2020, Herb. Pinzani
Field observations: partecipanti all'escursione Wikiplantbase #Parco di Migliarino - San Rossore - Massaciuccoli, 2018

Medicago lupulina L.
T scap/H scap - Paleotemp.
Literature data: Caruel 1860; Coaro 1987; Garbari 2001; Saggese 2016; Lazzeri in Buono et al. 2022
Herbarium data: Partecipanti all'escursione Wikiplantbase #Parco di Migliarino - San Rossore - Massaciuccoli, 2018, PI
Field observations: B. Pierini, 2016; partecipanti all'escursione Wikiplantbase #Parco di Migliarino - San Rossore - Massaciuccoli, 2018; L. Pinzani, 2021, 2022

Medicago marina L.
Ch rept - Eurimedit.
Literature data: Caruel 1860; Corti 1956 [*P. Savi*, s.d., FI; *E. Levier*, s.d., FI; *C. Rossetti*, s.d., FI; *s. coll.*, s.d., FI]; Garbari 2001; Tomei et al. 2004; Sani & Tomei 2006; Bertacchi et al. 2009; Bertacchi et al. 2010; Coulot & Rabaute 2013 [*P. Savi*, s.d., MPU]; Bertacchi & Lombardi 2014a
Herbarium data: L. Pinzani, 2020, Herb. Pinzani
Field observations: L. Pinzani, 2022

Medicago minima (L.) L.
T scap - Eurimedit.-Centroasiat.
Literature data: Caruel 1860; Corti 1956, Coaro 1987, under the name *M. minima* (L.) Grufb. var. *recta* (Desf.) Burnat; Garbari 2001; Sani & Tomei 2006 [*A. Sani*, 2005, PI]; Arduini & Ercoli 2012; Lombardi 2015; Lazzeri in Buono et al. 2022

Herbarium data: F. Roma-Marzio, L. Peruzzi, 2019, PI
Field observations: partecipanti all'escursione Wikiplantbase #Parco di Migliarino - San Rossore - Massaciuccoli, 2018

NC **Medicago monspeliaca** (L.) Trautv.
T scap - Eurimedit.
Literature data: Baroni 1897-1908, under the name *Trigonella monspeliaca* L.

NC **Medicago murex** Willd.
T scap - Stenomedit.
Literature data: Caruel 1860, under the name *M. sphaerocarpos* Bertol.

NC **Medicago orbicularis** (L.) Bartal.
T scap - Eurimedit.
Literature data: Caruel 1860

Medicago polymorpha L.
T scap - Eurimedit.-Subcosmop.
Literature data: Caruel 1860, under the name *M. denticulata* Mor.; Coaro 1987, under the name *M. hispida* Gaertner; Lazzeri in Buono et al. 2022
Herbarium data: L. Pinzani, 2020, Herb. Pinzani
Field observations: B. Pierini, 2016; partecipanti all'escursione Wikiplantbase #Parco di Migliarino - San Rossore - Massaciuccoli, 2018; L. Peruzzi, 2019; I. Arduini, 2024; H. Öhm, 2024

Medicago rigidula (L.) All.
T scap - Eurimedit.
Field observations: partecipanti all'escursione Wikiplantbase #Parco di Migliarino - San Rossore - Massaciuccoli, 2018

Medicago sativa L.
H scap - Eurasiat.
Literature data: Coaro 1987; Pedullà & Garbari, 2004; Saggese 2016; Lazzeri in Buono et al. 2022
Herbarium data: Partecipanti all'escursione Wikiplantbase #Parco di Migliarino - San Rossore - Massaciuccoli, 2018, PI
Field observations: partecipanti all'escursione Wikiplantbase #Parco di Migliarino - San Rossore - Massaciuccoli, 2018; L. Peruzzi, 2019; L. Pinzani, 2022

NC **Medicago scutellata** (L.) Mill.
T scap - Eurimedit.
Literature data: Caruel 1860; Coulot & Rabaute 2013 [*P. Savi*, s.d., TOU]

Medicago turbinata (L.) All.
T scap - Eurimedit.
Literature data: Caruel 1860, Baroni 1897-1908, under the name *M. tuberculata* (Retz.) Willd; Coulot & Rabaute 2013 [*P. Savi*, 1846, 1861, MPU]

NC **Onobrychis caput-galli** (L.) Lam.
T scap - Stenomedit.
Literature data: Caruel 1860; Baroni 1897-1908; Corti 1956 [*C. Rossetti*, s.d., FI]

Ononis reclinata L.
T scap - S-Medit.-Turan.
Literature data: Caruel 1860; Baroni 1897-1908; Corti 1956 [*O. Beccari*, s.d., FI; *C. Rossetti*, s.d., FI; *A. Tassi*, s.d., FI]; Corti 1956, under the name O. *reclinata* L. var. *minor* Moris; Coulot & Rabaute 2013 [*P. Savi*, 1862, PO]; Garbari 2001

Ononis spinosa L. subsp. **spinosa**
Ch suffr - Eurimedit.
Literature data: Caruel 1860; Coaro 1987; Garbari 2001; Bertacchi et al. 2009
Field observations: B. Pierini, 2014; L. Pinzani, 2020

Ornithopus compressus L.
T scap - Eurimedit.
Literature data: Caruel 1860; Corti 1956 [*P. Savi*, s.d., FI; *Narducci*, s.d., FI]; Corti 1956; Garbari 2001; Lombardi 2015; Bonari et al. 2019
Field observations: A. Giacò, J. Franzoni, L. Pinzani, 2021; L. Pinzani, 2021, 2022

Ornithopus pinnatus (Mill.) Druce
T scap - Medit.-Atl.
Literature data: Caruel 1860, under the name *O. ebracteatus* Brot.; Lombardi 2015

Robinia pseudoacacia L.
P scap - Orig. N-America
Literature data: Baroni 1897-1908; Corti 1956 [*M. Savelli*, s.d., FI]; Corti 1956, 1970; Coaro 1987 [*G. Pistolesi*, *E. Coaro*, 1977, 1985, PI]; Bertacchi et al. 2009; Bertacchi et al. 2010; Bertacchi & Lombardi 2014a; Bertacchi & Lombardi 2016
Field observations: L. Peruzzi, 2017; M. D’Antraccoli, 2018; partecipanti all'escursione Wikiplantbase #Parco di Migliarino - San Rossore - Massaciuccoli, 2018; F. Roma-Marzio, 2019; L. Pinzani, 2020, 2021, 2022; F. Roma-Marzio, 2021; I. Arduini, 2023

Scorpiurus subvillosus L.
T scap - Eurimedit.
Literature data: Caruel 1860
Herbarium data: Partecipanti all'escursione Wikiplantbase #Parco di Migliarino - San Rossore - Massaciuccoli, 2018, PI

Securigera varia (L.) Lassen
H scap - Circumbor.
Literature data: Caruel 1860, under the name *Coronilla varia* L.
Field observations: B. Pierini, 2020; L. Pinzani, 2021

Spartium junceum L.
P caesp - Eurimedit.
Literature data: Caruel 1860; Corti 1956 [*Chabert*, s.d., FI]; Corti 1956; Coaro 1987 [G. *Pistolesi*, *E. Coaro*, 1977, 1984, PI]; Garbari 2001; Bertacchi et al. 2009; Bertacchi et al. 2010; Bertacchi & Lombardi 2014a; Coulot & Rabaute 2016 [*P. Savi*, 1860, MPU, P]

Sulla coronaria (L.) B.H.Choi & H.Ohashi

H scap - W-Medit.
Literature data: Baroni 1897-1908, Corti 1956, Coaro 1987, Garbari 2001, under the name *Hedysarum coronarium* L.; Saggese 2016
Field observations: partecipanti all'escursione Wikiplantbase #Parco di Migliarino - San Rossore - Massaciuccoli, 2018; L. Pinzani, 2020, 2021, 2022; J. Franzoni, A. Giacò, 2023

Trifolium alexandrinum L.
T scap - E-Medit.
Literature data: Saggese 2016
Herbarium data: F. Roma-Marzio, M. D'Antraccoli, 2018, PI; L. Pinzani, 2020, Herb. Pinzani
Field observations: B. Pierini, 2017; partecipanti all'escursione Wikiplantbase #Parco di Migliarino - San Rossore - Massaciuccoli, 2018

Trifolium angustifolium L. subsp. **angustifolium**
T scap - Eurimedit.
Literature data: Caruel 1860; Corti 1956 [*G. Savi*, s.d., FI]; Corti 1956; Coaro 1987; Garbari 2001; Saggese 2016
Field observations: L. Pinzani, 2022

Trifolium arvense L. subsp. **arvense**
T scap - Paleotemp.
Literature data: Caruel 1860; Corti 1956 [*G. Savi*, s.d, FI; *A. Tassi*, s.d., FI]; Corti 1956, under the name *T. arvense* L. var. *agrestinum* (Jord.) Fiori; Coaro 1987; Garbari 2001; Bertacchi et al. 2009; Arduini & Ercoli 2012; Lombardi 2015; Bonari et al. 2019; Lazzeri in Buono et al. 2022
Field observations: partecipanti all'escursione Wikiplantbase #Parco di Migliarino - San Rossore - Massaciuccoli, 2018; L. Pinzani, 2021; I. Arduini, 2024

NC **Trifolium bocconei** Savi
T scap - Stenomedit.
Literature data: Caruel 1860

Trifolium campestre Schreb.
T scap - Paleotemp.
Literature data: Caruel 1860, under the name *T. procumbens* L.; Corti, 1951, under the name *T. campestre* Schreb. v. *pseudoprocumbens* (Gmel.) A. u. G. f. *nanum* (Ser. ap. DC.) A. u. G.; Coaro 1987; Garbari 2001; Arduini & Ercoli 2012; Lombardi 2015; Lazzeri in Buono et al. 2022
Field observations: B. Pierini, 2014; partecipanti all'escursione Wikiplantbase #Parco di Migliarino - San Rossore - Massaciuccoli, 2018; F. Roma-Marzio, 2021; L. Pinzani, 2021, 2022; I. Arduini, 2024; H. Öhm, 2024

Trifolium cherleri L.
T scap - Eurimedit.
Literature data: Caruel 1860; Corti, 1954, 1956; Corti 1956 [*P. Savi*, s.d., FI]; Lombardi 2015; Peruzzi et al. 2017b [*A. Sani*, 2015, PI]

NC **Trifolium diffusum** Ehrh.
T scap - Europ.-Caucas.
Literature data: Baroni 1897-1908, under the name *T. pallidum* Waldst. et Kit. var. *diffusum* Ehrh.

Trifolium dubium Sibth.
T scap - Europ.-Caucas.
Literature data: Coulot & Rabaute 2013 [*P. Savi*, 1860, MPU]; Lazzeri in Buono et al. 2022
Herbarium data: L. Pinzani, A. Giacò, J. Franzoni, 2021, Herb. Pinzani
Field observations: J. Franzoni, 2020

Trifolium echinatum M.Bieb.
T scap - Medit.-Turan.
Literature data: Caruel 1860, under the name *T. supinum* Savi; Coaro 1987; Coulot & Rabaute 2013 [*P. Savi*, 1856, MPU]; Saggese 2016

Trifolium fragiferum L. subsp. **fragiferum**
H rept - Paleotemp.
Literature data: Caruel 1860; Coaro 1987; Lazzeri in Buono et al. 2022
Herbarium data: Partecipanti all'escursione Wikiplantbase #Parco di Migliarino - San Rossore - Massaciuccoli, 2018, PI
Field observations: B. Pierini, 2017; partecipanti all'escursione Wikiplantbase #Parco di Migliarino - San Rossore - Massaciuccoli, 2018

Trifolium glomeratum L.
T scap - Eurimedit.
Literature data: Caruel 1860; Lombardi 2015

NC **Trifolium hybridum** L. subsp. **elegans** (Savi) Asch. & Graebn.
T scap - S-Europ.
Literature data: Baroni 1897-1908, under the name *T. elegans* Savi

Trifolium hybridum L. subsp. **hybridum**
H caesp - Medit.-Atl.
Field observations: partecipanti all'escursione Wikiplantbase #Parco di Migliarino - San Rossore - Massaciuccoli, 2018

Trifolium incarnatum L. subsp. **incarnatum**
T scap/H bienn - Eurimedit.
Literature data: Caruel 1860; Coaro 1987; Garbari 2001
Field observations: partecipanti all'escursione Wikiplantbase #Parco di Migliarino - San Rossore - Massaciuccoli, 2018; A. Giacò, J. Franzoni, L. Pinzani, 2021 A. Mo, 2022

Trifolium incarnatum L. subsp. **molinerii** (Hornem.) Ces.
T scap/H bienn - Eurimedit.
Literature data: Corti 1956 [*G. Savi*, s.d., FI; *P. Savi*, s.d., FI]; Coaro 1987

Trifolium infamia-ponertii Greuter
T scap - N-Medit.
Literature data: Coaro 1987, under the name *T. angustifolium* L. subsp. *gibellianum* Pign.

Trifolium lappaceum L.
T scap - Eurimedit.

Literature data: Caruel 1860; Baroni 1897-1908; Corti 1956 [*C. Rossetti*, s.d., FI; *F. Poggi*, s.d., FI; s. coll., s.d., FI]; Coaro 1987 [*G. Pistolesi, E. Coaro*, 1984, PI], under the name *T. hirtum* All.; Orlandi & Arduini 2010; Arduini & Ercoli 2012; Coulot & Rabaute 2013 [*P. Savi*, 1861, MPU]

Trifolium leucanthum M.Bieb.
T scap - E-Medit.
Herbarium data: G. Gestri, 2018, PI; Partecipanti all'escursione Wikiplantbase #Parco di Migliarino - San Rossore - Massaciuccoli, 2018, PI

NC **Trifolium ligusticum** Balb. ex Loisel.
T scap - Stenomedit.
Literature data: Caruel 1860

Trifolium medium L. subsp. **medium**
G rhiz - Eurasiat.-Occid.
Literature data: Coaro 1987
Herbarium data: S. Gerace, 2018, Herb. Gerace

NC **Trifolium michelianum** Savi
T scap - W-Medit.
Literature data: Caruel 1860; Baroni 1897-1908; Corti 1956 [*O. Beccari*, s.d., FI]; Coulot & Rabaute 2013 [*P. Savi*, 1861, MPU]
This species was described by Gaetano Savi (1798) on material collected from the Tenuta di San Rossore (Peruzzi et al. 2019).

Trifolium micranthum Viv.
T scap - Paleotemp.
Literature data: Caruel 1860, under the name *T. filiforme* L.; Corti 1956 [*G. Savi*, s.d., FI]; Corti 1956 [*P. Savi*, s.d., FI]
Herbarium data: G. Gestri, 2018, PI; Partecipanti all'escursione Wikiplantbase #Parco di Migliarino - San Rossore - Massaciuccoli, 2018, PI

Trifolium nigrescens Viv. subsp. **nigrescens**
T scap - Eurimedit.
Literature data: Caruel 1860; Corti, 1951, 1956; Garbari 2001; Lombardi 2015; Lazzeri in Buono et al. 2022
Herbarium data: F. Roma-Marzio, 2017, PI; Partecipanti all'escursione Wikiplantbase #Parco di Migliarino - San Rossore - Massaciuccoli, 2018, PI; L. Pinzani, 2021, Herb. Pinzani
Field observations: B. Pierini, 2016; F. Roma-Marzio, 2017; partecipanti all'escursione Wikiplantbase #Parco di Migliarino - San Rossore - Massaciuccoli, 2018; F. Roma-Marzio, 2019; A. Giacò, J. Franzoni, L. Pinzani, 2021; F. Roma-Marzio, 2021; L. Pinzani, 2022; H. Öhm, 2024

NC **Trifolium pallidum** Waldst. & Kit.
T scap/H bienn - Eurimedit.
Literature data: Caruel 1860; Corti 1956 [*A. Fiori*, s.d., FI]; Corti 1956, under the name *T. pratense* L. subsp. *pallidum* (W. et K.) Gib. et Bell.

NC **Trifolium patens** Schreb.
T rept - S-Europ.

Literature data: Caruel 1860; Corti 1956 [*P. Savi*, s.d., FI; *A. Tassi*, s.d., FI]; Corti 1956; Coulot & Rabaute 2013 [*P. Savi*, 1857, MPU]

Trifolium pratense L. subsp. **pratense**
H scap - Eurosiber.
Literature data: Caruel 1860; Baroni 1897-1908; Corti 1956 [*S. Sommier*, s.d., FI]; Coaro 1987; Garbari 2001; Pedullà & Garbari, 2004; Saggese 2016; Lazzeri in Buono et al. 2022
Herbarium data: Partecipanti all'escursione Wikiplantbase #Parco di Migliarino - San Rossore - Massaciuccoli, 2018, PI
Field observations: B. Pierini, 2016; partecipanti all'escursione Wikiplantbase #Parco di Migliarino - San Rossore - Massaciuccoli, 2018; F. Roma-Marzio, 2021; A. Mo, 2022; L. Pinzani, 2021, 2022; I. Arduini, 2023

Trifolium repens L.
H rept - Subcosmop.
Literature data: Caruel 1860; Coaro 1987; Orlandi & Arduini 2010; Arduini & Ercoli 2012; Saggese 2016; Lazzeri in Buono et al. 2022
Herbarium data: Partecipanti all'escursione Wikiplantbase #Parco di Migliarino - San Rossore - Massaciuccoli, 2018, PI
Field observations: B. Pierini, 2016; L. Peruzzi, 2018; L. Pinzani, 2022; I. Arduini, 2024

Trifolium resupinatum L.
T rept/H rept - Paleotemp.
Literature data: Caruel 1860; Corti 1956, under the name *T. resupinatum* L. var. *typicum* Asch. u. Gr.; Coaro 1987; Bonari et al. 2019; Lazzeri in Buono et al. 2022
Herbarium data: Partecipanti all'escursione Wikiplantbase #Parco di Migliarino - San Rossore - Massaciuccoli, 2018, PI
Field observations: B. Pierini, 2016, 2017; M. D'Antraccoli, 2019; L. Pinzani, 2021, 2022

Trifolium rubens L.
H scap - Centroeurop.
Herbarium data: G. Gestri, 2018, PI

Trifolium scabrum L.
T rept/T scap - Eurimedit.
Literature data: Caruel 1860; Lombardi 2015; Peruzzi et al. 2017b [*A. Sani*, 2015, PI]
Herbarium data: L. Pinzani, 2021, Herb. Pinzani
Field observations: B. Pierini, 2020

Trifolium squamosum L.
T scap - Eurimedit.
Literature data: Caruel 1860, Baroni 1897-1908, Corti 1956 [*F. Parlatore*, s.d., FI], Corti 1956, Pedullà & Garbari, 2004, under the name *T. maritimum* Hudson; Petraglia 2013; Saggese 2016; Peruzzi et al. 2017b [*F. Roma-Marzio, A. Carta, M. D'Antraccoli*, 2017, PI]; Lazzeri 2021
Herbarium data: F. Roma-Marzio, 2021, PI
Field observations: B. Pierini, 2017; A. Mo, I. Pucci, S. Pascacaldi, L. Pinzani, 2022; L. Pinzani, 2022

Trifolium squarrosum L.

T scap - Eurimedit.
Literature data: Caruel 1860; Pedullà & Garbari, 2004
Herbarium data: G. Gestri, 2018, PI; Partecipanti all'escursione Wikiplantbase #Parco di Migliarino - San Rossore - Massaciuccoli, 2018, PI
Field observations: B. Pierini, 2016; L. Pinzani, 2022

Trifolium stellatum L.
T scap - Eurimedit.
Literature data: Sani & Tomei 2006

Trifolium striatum L. subsp. **striatum**
T scap - Paleotemp.
Literature data: Caruel 1860; Coaro 1987

NC **Trifolium strictum** L.
T scap - Eurimedit.
Literature data: Caruel 1860

Trifolium subterraneum L. subsp. **subterraneum**
T rept - Eurimedit.
Literature data: Caruel 1860; Corti 1956 [*P. Savi*, s.d., FI; *O. Beccari*, s.d, FI; *P. Fantozzi*, s.d., FI; *Narducci*, FI]; Corti 1956, under the name *T. subterraneum* L. α *genuinum* Rouy in Rouy et Fouc.; Coaro 1987
Field observations: L. Pinzani, 2021, 2022

Trifolium tomentosum L.
T rept - Paleotemp.
Literature data: Baroni 1897-1908; Coaro 1987

Trifolium vesiculosum Savi
T scap - N-Medit.
Literature data: Corti 1956 [*P. Savi*, s.d., FI; *A. Fiori*, s.d., FI]; Coaro 1987; Coulot & Rabaute 2013 [*Lesourd*, 1860, MPU; *P. Savi*, 1870, MPU]
This species was described by Gaetano Savi (1798) for the maritime areas of Pisa (Peruzzi et al. 2019).

Trigonella alba (Medik.) Coulot & Rabaute
T scap - Subcosmop.
Literature data: Caruel 1860, Baroni 1897-1908, Coaro 1987, under the name *Melilotus alba* Desr.
Field observations: B. Pierini, 2016; partecipanti all'escursione Wikiplantbase #Parco di Migliarino - San Rossore - Massaciuccoli, 2018; L. Pinzani, 2020, 2022

Trigonella altissima (Thuill.) Coulot & Rabaute
G rhiz - Eurosiber.
Literature data: Garbari 2001

Trigonella dentata (Waldst. & Kit.) Coulot & Rabaute
Literature data: Coaro 1987, under the name *M. dentata* (W. et K.) Pers.

NC **Trigonella italica** (L.) Coulot & Rabaute
T scap - N-Medit.
Literature data: Caruel 1860, under the name *Melilotus italica* Pers.

Trigonella officinalis (L.) Coulot & Rabaute
H bienn - Eurasiat.-Subcosmop.
Literature data: Caruel 1860, Coaro 1987, under the name *Melilotus officinalis* (L.) Desr.
Herbarium data: Partecipanti all'escursione Wikiplantbase #Parco di Migliarino - San Rossore - Massaciuccoli, 2018, PI

Trigonella segetalis (Brot.) Coulot & Rabaute
T scap - S-Medit.
Literature data: Coaro 1987, under the name *Melilotus segetalis* (Brot.) Ser.

Trigonella sicula (Turra) Coulot & Rabaute
T scap - S-Medit.
Literature data: Saggese 2016, under the name *Melilotus messanensis* (L.) All.

Trigonella smallii Coulot & Rabaute
T scap - Medit.-Turan.
Literature data: Baroni 1897-1908, Saggese 2016, under the name *Melilotus indicus* (L.) All.
Herbarium data: G. Gestri, 2018, PI

Trigonella sulcata (Desf.) Coulot & Rabaute
T scap - S-Medit.
Literature data: Caruel 1860, Corti 1956 [*P. Fantozzi*, FI]; Saggese 2016, under the name *Melilotus sulcata* Desf.
Herbarium data: L. Pinzani, 2021, Herb. Pinzani

Trigonella wojciechowskii Coulot & Rabaute
T scap - Stenomedit.
Literature data: Caruel 1860, under the name *Melilotus neapolitana* Ten.; Lazzeri et al. 2018 [*V. Lazzeri*, 2018, FI]
Field observations: L. Pinzani, 2021, 2022

Ulex europaeus L. subsp. **europaeus**
P caesp - Subatl.
Literature data: Garbari 2001
Field observations: A. Mo, 2022.

Vicia angustifolia L.
T scap - Stenomedit.
Literature data: Caruel 1860; Lazzeri 2021, 2022
Field observations: L. Pinzani, 2022

NC **Vicia benghalensis** L.
T scap - Stenomedit.
Literature data: Caruel 1860, under the name *V. atropurpurea* Desf.

Vicia bithynica (L.) L.
T scap - Eurimedit.
Literature data: Caruel 1860; Coaro 1987; Garbari 2001
Field observations: B. Pierini, 2016; L. Pinzani, 2021

Vicia cassubica L.
H scap - Centroeurop.
Literature data: Coaro 1987

NC **Vicia cordata** Wulfen ex Hoppe
T scap - Eurimedit.
Literature data: Corti 1956, under the name *V. sativa* L. subsp. *cordata* (Wulf) Asch. u. Gr.

Vicia dasycarpa Ten.
T scap (H bienn) - Eurimedit.
Literature data: Caruel 1860, under the name *V. villosa* Roth var. *glabrescens* Koch; Corti 1956 [*O. Beccari*, s.d., FI]; Sani & Tomei 2006 [*A. Sani*, 2005, PI], under the name *V. villosa* Roth subsp. *varia* (Host) Corb.
Field observations: B. Pierini, 2017

NC **Vicia disperma** DC.
T scap - W-Medit.
Literature data: Caruel 1860, under the name *Ervum parviflorum* Bert.

Ex **Vicia elegantissima** Shuttlew. ex Rouy
Literature data: Coulot & Rabaute 2016 [*Billot*, 1857, MPU]

Vicia faba L.
Field observations: B. Pierini, 2024

Vicia hybrida L.
T scap - Eurimedit.
Literature data: Caruel 1860; Peruzzi et al. 2017b [*F. Roma-Marzio, A. Carta, M. D'Antraccoli*, 2017, PI]
Field observations: B. Pierini, 2020; L. Pinzani, 2021, 2022

Vicia incana Gouan
H scap - Eurimedit.-W-Asiat.
Field observations: partecipanti all'escursione Wikiplantbase #Parco di Migliarino - San Rossore - Massaciuccoli, 2018

NC **Vicia lathyroides** L.
T scap - Eurimedit.
Literature data: Corti 1956 [*O. Beccari*, s.d., FI]

Vicia lutea L.
T scap - Eurimedit.
Literature data: Caruel 1860; Coulot & Rabaute 2016 [*P. Savi*, 1861, MPU]
Herbarium data: G. Gestri, 2018, PI

Field observations: L. Pinzani, 2022

Vicia macrocarpa (Moris) Bertol.
T scap - Medit.-Turan.-Subcosmop.
Herbarium data: Partecipanti all'escursione Wikiplantbase #Parco di Migliarino - San Rossore - Massaciuccoli, 2018, PI

NC **Vicia narbonensis** L.
T scap - Eurimedit.
Literature data: Baroni 1897-1908

NC **Vicia pannonica** Crantz subsp. **striata** (M.Bieb.) Nyman
T scap - Medit.
Literature data: Baroni 1897-1908, under the name *Trisetum aureum* Ten..

Vicia peregrina L.
T scap - Medit.-Turan.
Literature data: Baroni 1897-1908; Tomei et al. 2004; Coulot & Rabaute 2016 [*P. Savi*, 1860, MPU]

Vicia pseudocracca Bertol.
T scap - Stenomedit.
Literature data: Caruel 1860; Corti, 1951, 1956; Corti 1956 [*P. Savi*, s.d., FI; *O. Beccari*, s.d., FI; *Narducci*, s.d., FI; *A. Fiori*, s.d., FI]; Garbari 2001; Tomei et al. 2004; Arduini & Ercoli 2012; Lombardi 2015 Coulot & Rabaute 2016 [*P. Savi*, s.d., MPU]
Herbarium data: L. Pinzani, 2020, Herb. Pinzani
Field observations: L. Pinzani, 2021, 2022

Vicia sativa L.
T scap - Medit.-Subcosmop.
Literature data: Caruel 1860; Coaro 1987; Garbari 2001; Saggese 2016; Lazzeri in Buono et al. 2022
Herbarium data: F. Roma-Marzio, L. Peruzzi, 2019, PI; L. Pinzani, 2020, Herb. Pinzani
Field observations: B. Pierini, 2017; M. D'Antraccoli, 2019; L. Pinzani, 2021

Vicia segetalis Thuill.
T scap - Paleotrop.
Literature data: Garbari 2001, under the name *V. sativa* L. subsp. *segetalis* (Thuill.) Gaudin

Wisteria sinensis (Sims) DC.
Field observations: observed on iNaturalist in 2020, 2022
Only cultivated

Polygalaceae

NC **Polygala flavescens** DC. subsp. **flavescens**
H scap - Endem. Ital.
Literature data: Baroni 1897-1908; Peruzzi et al. 2019 [*A. Chiarugi*, 1933, FI]

Polygala monspeliaca L.
T scap - Stenomedit.
Literature data: Garbari 2001

Polygala nicaeensis Risso ex W.D.J.Koch subsp. **italiana** (Chodat) Arrigoni
H scap - Endem. Ital.
Literature data: Corti 1956 [*A. Biondi*, FI]; Garbari 2001
Herbarium data: L. Pinzani, 2020, Herb. Pinzani
Assessed as DD in the Red List of the Italian Vascular Flora (Rossi et al. 2020)

Polygala vulgaris L. subsp. **vulgaris**
H scap - Eurasiat.
Literature data: Coaro 1987

Rosaceae

Agrimonia eupatoria L. subsp. **eupatoria**
H scap - Subcosmop.
Literature data: Caruel 1860; Corti 1956 [*M. Savelli*, s.d., FI], Corti 1956; Coaro 1987; Garbari 2001; Pedullà & Garbari, 2004; Saggese 2016; Lazzeri 2021
Herbarium data: Partecipanti all'escursione Wikiplantbase #Parco di Migliarino - San Rossore - Massaciuccoli, 2018, PI
Field observations: partecipanti all'escursione Wikiplantbase #Parco di Migliarino - San Rossore - Massaciuccoli, 2018

Aphanes arvensis L.
T scap - Subcosmop.
Herbarium data: L. Pinzani, 2020, Herb. Pinzani
Field observations: L. Pinzani, 2021, 2022

Aphanes australis Rydb.
T scap - Subatl.
Literature data: Corti 1956, under the name *A. microcarpa* (Boiss. & Reut.) Rothm.; Orlandi & Arduini 2010; Arduini & Ercoli 2012, Lombardi 2015, Bonari et al. 2019, under the name *A. inexspectata* Lippert; Peruzzi et al. 2017 [*A. Sani*, 2015, PI], under the name *Aphanes microcarpa* (Boiss. & Reut.) Rothm.
Field observations: I. Arduini, 2024

Crataegus monogyna Jacq.
P caesp/P scap - Paleotemp.
Literature data: Caruel 1860, Corti, 1951, 1956, under the name *C. oxyacantha* L. var. *monogyna* Jacq.; Corti, 1970; Gellini et al. 1986; Coaro 1987 [*G. Pistolesi, E. Coaro*, 1977, PI; *E. Coaro*, 1984, PI]; Garbari 2001 [*B. Ciacchi, G. Lorè*, 1996, 1997, PI]; Tomei et al. 2004; Arduini & Ercoli 2012; Petraglia 2013; Lombardi 2015; Bertacchi & Lombardi 2016; Bonari et al. 2019; Lazzeri 2021
Herbarium data: C. Del Prete, 1979, PI; R. Poli, 1989, PI;; M.L. Pedullà, 2000, PI
Field observations: L. Peruzzi, 2017; partecipanti all'escursione Wikiplantbase #Parco di Migliarino - San Rossore - Massaciuccoli, 2018; L. Pinzani, 2020, 2021, 2022; I. Arduini, 2023

Cydonia oblonga Mill.
Field observations: observed on iNaturalist in 2023
Only cultivated

NC **Filipendula vulgaris** Moench
H scap - C-Europ.-S-Siber.
Literature data: Caruel 1860, under the name *Spiraea filipendula* L.

Fragaria vesca L. subsp. **vesca**
H rept - Eurosiber.
Literature data: Corti 1956, under the name *F. vesca* L. var. *sylvestris* L.; Coaro 1987; Garbari 2001

Geum urbanum L.
H scap - Circumbor.
Literature data: Baroni 1897-1908; Corti 1956 [*P. Fantozzi*, s.d., FI]; Corti 1956; Garbari 2001; Lombardi 2015
Field observations: partecipanti all'escursione Wikiplantbase #Parco di Migliarino - San Rossore - Massaciuccoli, 2018; I. Arduini, 2024

Malus sylvestris (L.) Mill.
P scap - C-Europ.-Caucas.
Literature data: Coaro 1987; Orlandi & Arduini 2010; Arduini & Ercoli 2012
Herbarium data: R. Poli, 1989, PI
Field observations: I. Arduini, 2024

Mespilus germanica L.
Field observations: observed on iNaturalist in 2022, under the name *Crataegus germanica* (L.) Kuntze
Only cultivated

Photinia ×fraseri Dress
Field observations: observed on iNaturalist in 2022
Only cultivated

D **Potentilla anglica** Laichard.
H scap - C-Europ.-N-Europ.
Literature data: Caruel 1860, under the name *P. procumbens* Sibth.; Baroni 1897-1908

Potentilla argentea L.
H scap - Circumbor.
Literature data: Caruel 1860; Baroni 1897-1908; Corti 1956 [*P. Savi*, s.d., FI]; Gellini et al. 1986

Potentilla erecta (L.) Raeusch.
H scap - Eurasiat.
Literature data: Gellini et al. 1986

NC **Potentilla inclinata** Vill.
H scap - Eurasiat.-Temp.

Literature data: Caruel 1860

Potentilla pedata Willd. ex Hornem.
H scap - Centromedit.
Literature data: Caruel 1860, under the name *P. hirta* L.; Corti 1956 [*O. Beccari*, s.d., FI]

Potentilla reptans L.
H ros - Paleotemp.
Literature data: Caruel 1860, Baroni 1897-1908, under the name *P. procumbens* Sibth.; Corti 1956 [*P. Savi*, s.d., FI; *Picciuoli*, s.d., FI]; Corti 1956, under the name *P. reptans* L. var. *typica* Th. Wolf; Gellini et al. 1986; Coaro 1987; Garbari 2001; Tomei et al. 2004; Sani & Tomei 2006 [*A. Sani*, 2005, PI]; Petraglia 2013; Lombardi 2015; Bertacchi & Lombardi 2016; Lazzeri 2021, 2022
Herbarium data: Partecipanti all'escursione Wikiplantbase #Parco di Migliarino - San Rossore - Massaciuccoli, 2018, PI
Field observations: B. Pierini, 2014; partecipanti all'escursione Wikiplantbase #Parco di Migliarino - San Rossore - Massaciuccoli, 2018; M. D'Antraccoli, 2019; L. Pinzani, 2020, 2021, 2022; I. Arduini, 2023

Poterium sanguisorba L. subsp. **sanguisorba**
H scap - Paleotemp.
Literature data: Corti 1956, Garbari 2001, under the name *Sanguisorba minor* Scop.; Coaro 1987, under the name *Sanguisorba minor* Scop. subsp. *minor*
Field observations: L. Pinzani, 2022

Prunus avium L.
P scap - Eurasiat.-Pont.
Literature data: Levier & Sommier 1891
Herbarium data: G. Pistolesi, E. Coaro, 1977, PI

Prunus spinosa L. subsp. **spinosa**
P caesp - Europ.-Caucas.
Literature data: Caruel 1860; Corti 1956, 1970; Gellini et al. 1986; Coaro 1987 [*E. Coaro*, 1984, PI]; Garbari 2001; Tomei et al. 2004; Lombardi 2015; Bertacchi & Lombardi 2016; Lazzeri 2021
Herbarium data: M.L. Pedullà, 1999, PI
Field observations: L. Peruzzi, 2017; M. D'Antraccoli, 2018; partecipanti all'escursione Wikiplantbase #Parco di Migliarino - San Rossore - Massaciuccoli, 2018; L. Pinzani, 2020; I. Arduini, 2023

Pyracantha coccinea M.Roem.
P caesp - Stenomedit.
Literature data: Caruel 1860, under the name *Cotoneaster pyracantha* Spach; Corti 1956; Garbari 2001
Herbarium data: D. Marchetti, 2006, PI

Pyrus communis L.
P scap - Eurasiat.
Literature data: Corti 1956; Coaro 1987, Garbari 2001, under the name *P. pyraster* Burgsd.; Coaro 1987, Lazzeri 2021

Herbarium data: P.E. Tomei, 1977, PI; Partecipanti all'escursione Wikiplantbase #Parco di Migliarino - San Rossore - Massaciuccoli, 2018, PI
All the records refer to the wild pear (*Pyrus communis* L. subsp. *pyraster* (L.) Ehrh.).

Rhaphiolepis bibas (Lour.) Galasso & Banfi
Field observations: L. Pinzani, 2022

Rosa agrestis Savi
NP - Eurimedit.
Literature data: Caruel 1860, under the name *R. rubiginosa* Linn. var. *sepium* Savi; Orlandi & Arduini 2010; Arduini & Ercoli 2012
Herbarium data: F. Roma-Marzio, M. D'Antraccoli, 2017, PI

Rosa canina L.
NP - Paleotemp.
Literature data: Caruel 1860, under the name *R. canina* var. *vulgaris* Koch; Corti 1956 [*s. coll.*, s.d., FI]; Garbari 2001
Field observations: partecipanti all'escursione Wikiplantbase #Parco di Migliarino - San Rossore - Massaciuccoli, 2018.

Rosa corymbifera Borkh.
NP - Paleotemp.
Literature data: Caruel 1860, under the name *R. canina* var. *dumetorum* Koch
Herbarium data: L. Pinzani, 2020, Herb. Pinzani

Rosa nitidula Besser
NP - Eurimedit.
Herbarium data: L. Pinzani, 2020, Herb. Pinzani

Rosa sempervirens L.
NP - Stenomedit.
Literature data: Caruel 1860; Corti 1956 [*T. Caruel*, s.d., FI]; Corti 1956; Coaro 1987 [*E. Coaro*, 1977, 1984, PI]; Garbari 2001; Tomei et al. 2004; Lazzeri 2021
Herbarium data: C. Del Prete, 1979, PI; R. Poli, 1989, PI
Field observations: L. Peruzzi, 2017; partecipanti all'escursione Wikiplantbase #Parco di Migliarino - San Rossore - Massaciuccoli, 2018; L. Pinzani, 2020

Rubus caesius L.
NP - Eurasiat.
Literature data: Caruel 1860; Corti 1956 [*M. Savelli*, s.d., FI]; Corti 1956, under the name *R. caesius* L. var. *arvalis* Rchb.; Garbari 2001; Tomei et al. 2004

NC **Rubus praecox** Bertol.
NP - C-Europ.
Literature data: Corti 1956

NC **Rubus sulcatus** Vest
NP - Subatl.
Herbarium data: O. Beccari, 1863, FI

Rubus ulmifolius Schott
NP - Eurimedit.
Literature data: Caruel 1860, under the name *R. discolor* Weih.et Nees; Corti 1956, under the name *R. fruticosus* L. *sensu latissimo*; Gellini et al. 1986; Coaro 1987: [*G. Pistolesi, E. Coaro*, 1977, PI; *E. Coaro*, 1984, PI]; Garbari 2001; Tomei et al. 2004; Sani & Tomei 2006; Bertacchi et al. 2009; Bertacchi et al. 2010; Arduini & Ercoli 2012; Bertacchi & Lombardi 2014b; Bertacchi & Lombardi 2014a; Lombardi 2015; Bertacchi & Lombardi 2016; Saggese 2016; Bonari et al. 2019; Lazzeri 2021, 2022
Herbarium data: C. Del Prete, 1979, PI
Field observations: L. Peruzzi, 2017, 2019; partecipanti all'escursione Wikiplantbase #Parco di Migliarino - San Rossore - Massaciuccoli, 2018; L. Pinzani, 2021, 2022; A. Mo, 2023

Rhamnaceae

Frangula alnus Mill. subsp. **alnus**
P caesp - Europ.-Caucas.
Literature data: Gellini et al. 1986; Coaro 1987 [*E. Coaro*, 1984, PI]; Garbari 2001; Tomei et al. 2004
Herbarium data: B. Ciacchi, G. Lorè, 1996, 1997, PI

Rhamnus alaternus L. subsp. **alaternus**
P caesp - Stenomedit.
Literature data: Baroni 1897-1908; Bertacchi et al. 2010; Lazzeri 2021
Field observations: partecipanti all'escursione Wikiplantbase #Parco di Migliarino - San Rossore - Massaciuccoli, 2018; L. Pinzani, 2020, 2021; A. Mo, 2023; L. Peruzzi, 2024

Rhamnus cathartica L.
P caesp/P scap - S-Europ.-Pont.
Literature data: Caruel 1860; Gellini et al. 1986; Tomei et al. 2004

Elaeagnaceae

Elaeagnus angustifolia L.
P scap - Orig. Asia
Literature data: Garbari 2001; Bertacchi et al. 2009; Bertacchi et al. 2010
Herbarium data: K.F. Caparelli, L. Peruzzi, 2010, PI; Partecipanti all'escursione Wikiplantbase #Parco di Migliarino - San Rossore - Massaciuccoli, 2018, PI

Hippophaë fluviatilis (Soest) Rivas Mart.
P caesp - Eurasiat.
Literature data: Garbari 2001, Tomei et al. 2004, under the name *H. rhamnoides* L.

Ulmaceae

NC **Ulmus glabra** Huds.
P scap – Europ.-Caucas.
Literature data: Corti 1956, under the name *U. scabra* Mill.

Ulmus minor Mill. subsp. **minor**
P caesp/P scap - Europ.-Caucas.
Literature data: Caruel 1860, Corti, 1951, 1956, under the name *U. campestris* L.; Corti, 1970, under the name *U. carpinifolia*; Gellini et al. 1986; Coaro 1987; Garbari 2001; Uncini Manganelli et al. 2001; Tomei et al. 2004; Bertacchi et al. 2010; Arduini & Ercoli 2012; Lombardi 2015; Bertacchi & Lombardi 2016; Lazzeri 2021
Herbarium data: A. Preta, 1979, PI; R. Poli, 1989, PI
Field observations: L. Peruzzi, 2017, 2019; partecipanti all'escursione Wikiplantbase #Parco di Migliarino - San Rossore - Massaciuccoli, 2018; L. Pinzani, 2020, 2021, 2022; I. Arduini, 2022; A. Mo, 2023

Cannabaceae

Celtis australis L. subsp. **australis**
P scap - Eurimedit.
Literature data: Baroni 1897-1908; Pedullà & Garbari, 2004
Herbarium data: M.L. Pedullà, 2000, PI
Field observations: I. Arduini, 2024 (cultivated)

Humulus lupulus L.
P lian - Europ.-Caucas.
Literature data: Caruel 1860; Corti 1956; Peruzzi et al. 2017b [*F. Roma-Marzio, M. D'Antraccoli*, 2017, PI]; Garbari 2001; Lazzeri 2021
Field observations: L. Pinzani, 2021, 2022

Moraceae

Broussonetia papyrifera (L.) Vent.
P caesp/P scap. - Orig. E-Asia
Literature data: Corti 1956; Garbari 2001

Ficus carica L.
P scap - Medit.-Turan.
Literature data: Caruel 1860; Corti 1956; Gellini et al. 1986; Garbari 2001; Tomei et al. 2004; Arduini & Ercoli 2012
Field observations: L. Pinzani, 2021, 2022; I. Arduini, 2024

Juglans nigra L.
Field observations: I. Arduini, 2024
Only cultivated

Maclura pomifera (Raf.) C.K.Schneid.
Literature data: Galasso et al. 2016 [*M. D'Antraccoli*, *F. Roma-Marzio*, 2015, FI]

Morus indica L.
Field observations: observed on iNaturalist in 2024
Only cultivated

Urticaceae

Parietaria judaica L.
H scap - Europ.
Literature data: Corti 1956, under the name *P. vulgaris* Hill; Garbari 2001, Pedullà & Garbari, 2004, under the name *P. diffusa* M. et K.
Field observations: L. Peruzzi, 2015, 2019; partecipanti all'escursione Wikiplantbase #Parco di Migliarino - San Rossore - Massaciuccoli, 2018; F. Roma-Marzio, 2019; L. Pinzani, 2022; H. Öhm, 2024

Parietaria officinalis L.
H scap - Centroeurop.-W-Asiat.
Literature data: Caruel 1860; Bertacchi et al. 2009

Soleirolia soleirolii (Req.) Dandy
H scap - Orig. W-Medit.
Field observations: H. Öhm, 2024

Urtica dioica L.
H scap - Subcosmop.
Literature data: Caruel 1860; Corti 1956, under the name *U. dioica* L. var. *vulgaris* Wedd. forma *typica* Pospich; Gellini et al. 1986; Corsi et al. 1999 [*Chiarugi, Corti, Negri*, 1951, FI]; Garbari 2001; Pedullà & Garbari, 2004; Tomei et al. 2004; Arduini & Ercoli 2012; Lombardi 2015; Bertacchi & Lombardi 2016
Field observations: L. Peruzzi, 2015; partecipanti all'escursione Wikiplantbase #Parco di Migliarino - San Rossore - Massaciuccoli, 2018; L. Pinzani, 2021, 2022; I. Arduini, 2023

Urtica membranacea Poir. ex Savigny
T scap - S-Medit.
Literature data: Caruel 1860; Corti 1956; Corsi et al. 1999 [*P. Savi*, 1842, BR, FI, RO]; Garbari 2001
Herbarium data: L. Pinzani, 2020, Herb. Pinzani
Field observations: partecipanti all'escursione Wikiplantbase #Parco di Migliarino - San Rossore - Massaciuccoli, 2018; L. Pinzani, 2021, 2022; I. Arduini, 2023

NC **Urtica urens** L.
T scap - Subcosmop.
Literature data: Corsi et al. 1999 [*P. Savi*, 1842, BR, FI]

Fagaceae

Quercus cerris L.
P scap - Eurimedit.
Literature data: Caruel 1860; Tomei et al. 2004; Bertacchi & Lombardi 2016; Bonari et al. 2019
Field observations: partecipanti all'escursione Wikiplantbase #Parco di Migliarino - San Rossore - Massaciuccoli, 2018; I. Arduini, 2022, 2024

Quercus ilex L.
P scap - Stenomedit.

Literature data: Caruel 1860; Corti 1956, 1970; Gellini et al. 1986; Coaro 1987 [*G. Pistolesi*, *E. Coaro*, 1985, PI]; Arrigoni & Menicagli 1999; Garbari 2001; Tomei et al. 2004; Bertacchi et al. 2009; Bertacchi et al. 2010; Arduini & Ercoli 2012; Lombardi 2015; Bertacchi & Lombardi 2016; Bonari et al. 2019; Lazzeri 2021
Herbarium data: M. Savelli, 1906, 1916, FI; A. Preta, 1979, PI
Field observations: R. Dell'Orso, 2015; L. Peruzzi, 2017; M. D’Antraccoli, 2018; partecipanti all'escursione Wikiplantbase #Parco di Migliarino - San Rossore - Massaciuccoli, 2018; F. Roma-Marzio, 2019; L. Pinzani, 2020, 2021, 2022; A. Giacò, J. Franzoni, L. Pinzani, 2021; F. Roma-Marzio, 2021; I. Arduini, 2023

Quercus petraea (Matt.) Liebl. subsp. **petraea**
P scap/P caesp - Europ.-Subatl.
Literature data: Corti 1956, 1970, under the name *Q. sessilis* Ehrh.
Field observations: L. Peruzzi, 2024

Quercus pubescens Willd. subsp. **pubescens**
P caesp - SE-Europ.
Literature data: Corti 1956, 1970; Garbari 2001
Field observations: I. Arduini, 2015; L. Peruzzi, 2017; M. D’Antraccoli, 2018; partecipanti all'escursione Wikiplantbase #Parco di Migliarino - San Rossore - Massaciuccoli, 2018; F. Roma-Marzio, 2019; I. Arduini, 2024

Quercus robur L. subsp. **robur**
P scap - Europ.-Caucas.
Literature data: Caruel 1860, Corti 1956 [*P. Savi*, s.d., FI; *T. Caruel*, s.d., FI]; Corti 1956, 1970, under the name *Q. pedunculata* Ehrh.; Gellini et al. 1986; Coaro 1987 [*G. Pistolesi*, *E. Coaro*, 1984, PI]; Tomei et al. 2004; Lombardi 2015; Bertacchi & Lombardi 2016; Lazzeri 2021
Herbarium data: Parlatore, 1863, FI; A. Preta, 1979, PI; R. Poli, 1989, PI; F. Roma-Marzio, M. D’Antraccoli, 2017, PI
Field observations: I. Arduini, 2025; L. Peruzzi, 2017; partecipanti all'escursione Wikiplantbase #Parco di Migliarino - San Rossore - Massaciuccoli, 2018; B. Pierini, 2020; L. Pinzani, 2020; I. Arduini, 2022

Quercus rubra L.
Field observations: L. Peruzzi, 2024

Quercus suber L.
P scap - W-Medit.
Literature data: Caruel 1860; Corti 1956 [*F. Parlatore*, s.d., FI; *Roberti*, s.d., FI; *Corinaldi*, s.d., FI]; Bertacchi et al. 2010; Bertacchi & Lombardi 2016
Field observations: L. Peruzzi, 2017; M. D’Antraccoli, 2018; L. Pinzani, 2020, 2021

Betulaceae

Alnus cordata (Loisel.) Duby
Field observations: observed on iNaturalist in 2019
Only cultivated

Alnus glutinosa (L.) Gaertn.

P scap - Paleotemp.
Literature data: Caruel 1860; Baroni 1897-1908; Corti 1956, under the name *A. glutinosa* (L.) Gaertn. var. *vulgaris* Spach; Corti, 1970; Gellini et al. 1986; Coaro 1987; Garbari 2001 [*B. Ciacchi, G. Lorè*, 1997, PI]; Tomei et al. 2004; Bertacchi et al. 2010; Petraglia 2013
Herbarium data: S. Sommier, 1871, 1899, FI; F. Roma-Marzio, M. D'Antraccoli, 2017, PI
Field observations: J. Franzoni, 2018; I. Arduini, 2022

Carpinus betulus L.
P scap/P caesp - Centroeurop.-Caucas.
Literature data: Corti 1956,1970, under the name *C. betulus* L. var. *carpinizza* Neilr.; Gellini et al. 1986; Coaro 1987 [*G. Pistolesi*, *E. Coaro*, 1984, PI]; Tomei et al. 2004; Bertacchi & Lombardi 2016
Herbarium data: A. Chiarugi, R. Corti, 1951, FI; A. Chiarugi, R. Corti, G. Negri, 1951, FI; R. Poli, 1989, PI
Field observations: I. Arduini, 2022

Cucurbitaceae

Bryonia dioica Jacq.
G rhiz/H scand - Eurimedit.
Literature data:
Herbarium data: Partecipanti all'escursione Wikiplantbase #Parco di Migliarino - San Rossore - Massaciuccoli, 2018, PI
Field observations: B. Pierini 2017

Ecballium elaterium (L.) A.Rich.
G bulb - Eurimedit.
Literature data: Caruel 1860; Corti 1956; Garbari 2001; Pedullà & Garbari, 2004; Sani & Tomei 2006

Celastraceae

Euonymus europaeus L.
P caesp - Eurasiat.
Literature data: Caruel 1860; Corti, 1951, 1956, 1970; Gellini et al. 1986; Coaro 1987 [*G. Pistolesi*, *E. Coaro*, 1977, PI]; Tomei et al. 2004; Bertacchi et al. 2010; Lombardi 2015; Bertacchi & Lombardi 2016; Lazzeri 2021
Herbarium data: R. Poli, 1989, PI; B. Ciacchi, G. Lorè, 1996, 1997, PI; L. Pinzani, 2020, Herb. Pinzani
Field observations: L. Peruzzi, 2017; B. Pierini, 2017; partecipanti all'escursione Wikiplantbase #Parco di Migliarino - San Rossore - Massaciuccoli, 2018; L. Pinzani, 2020, 2022; I. Arduini, 2023

Euonymus japonicus Thunb.
Herbarium data: Partecipanti all'escursione Wikiplantbase #Parco di Migliarino - San Rossore - Massaciuccoli, 2018, PI
Field observations: partecipanti all'escursione Wikiplantbase #Parco di Migliarino - San Rossore - Massaciuccoli, 2018; L. Pinzani, 2020

Oxalidaceae

Oxalis articulata Savigny
G rhiz - Orig. S-America
Literature data: Peruzzi et al. 2017b [*F. Roma-Marzio, M. D’Antraccoli*, 2017, PI]
Field observations: B. Pierini, 2014; L. Peruzzi, 2015; partecipanti all'escursione Wikiplantbase #Parco di Migliarino - San Rossore - Massaciuccoli, 2018; M. D’Antraccoli, 2020; L. Pinzani, 2021, 2022; I. Arduini, 2024

Oxalis corniculata L.
H rept - Eurimedit.
Literature data: Caruel 1860; Corti 1956, under the name *O. corniculata* L var. *villosa* (M. Bieb.) Hohenacker; Gellini et al. 1986; Garbari 2001; Arduini & Ercoli 2012; Bonari et al. 2019
Herbarium data: F. Roma-Marzio, M. D’Antraccoli, 2017, PI; F. Roma-Marzio et G. Astuti, 2017, PI
Field observations: L. Peruzzi, 2015, 2019; I. Arduini, 2023

Oxalis debilis Kunth
G bulb - Orig. S-America
Literature data: Pierini in Peruzzi et al. 2011 [B. Pierini, 2011, PI], under the name *Oxalis corymbosa* DC.
Field observations: F. Roma-Marzio, 2019

Oxalis dillenii Jacq.
H scap - Orig. N-America
Literature data: Coaro 1987; Lombardi 2015; Peruzzi et al. 2017b [*A. Sani, M. D’Antraccoli*, 2014, PI]

Oxalis latifolia Kunth
G bulb - Orig. America
Literature data: Pierini 2011 [*B. Pierini*, 2011, FI]
Field observations: M. D’Antraccoli, 2017; L. Pinzani, 2022

Oxalis pes-caprae L.
G bulb - Orig. S-Africa
Herbarium data: L. Pinzani, 2020, Herb. Pinzani

Oxalis stricta L.
Literature data: Caruel 1860; Corti 1956
Field observations: I. Arduini, 2024

Euphorbiaceae

Euphorbia amygdaloides L.
Ch suffr - Centroeurop.-Caucas.
Literature data: Coaro 1987

Euphorbia biumbellata Poir.
Ch suffr - W-Medit.
Literature data: Garbari 2001

NC **Euphorbia chamaesyce** L.
T rept - Eurimedit.
Literature data: Caruel 1860

NC **Euphorbia characias** L.
NP - Stenomedit.
Herbarium data*: s.coll.*, 1863, FI

Euphorbia cyparissias L.
H scap - Centroeurop.
Literature data: Caruel 1860; Corti 1956; Gellini et al. 1986; Coaro 1987; Garbari 2001; Tomei et al. 2004; Arduini & Ercoli 2012; Lombardi 2015
Field observations: L. Pinzani, 2022; I. Arduini, 2023

Euphorbia esula L. subsp. **esula**
H scap - Eurosiber.
Literature data: Caruel 1860; Corti 1956
Herbarium data: L. Pinzani, 2020, Herb. Pinzani

NC **Euphorbia exigua** L. subsp. **exigua**
H scap - Eurimedit.
Literature data: Caruel 1860

NC **Euphorbia falcata** L.
T scap - Eurimedit.-Turan.
Literature data: Caruel 1860

Euphorbia helioscopia L. subsp. **helioscopia**
T scap - Cosmop.
Literature data: Caruel 1860; Coaro 1987; Garbari 2001
Field observations: B. Pierini, 2014; L. Pinzani, 2020, 2021, 2022; F. Roma-Marzio, 2021; I. Arduini, 2023

Euphorbia hirsuta L.
G rhiz - Medit.-Macarones.
Literature data: Caruel 1860, Corti 1956 [*P. Savi*, s.d., FI; *Picciuoli*, s.d., FI], Coaro 1987, Garbari 2001, Tomei et al. 2004, Petraglia 2013, under the name *E. pubescens* Vahl

NC *Euphorbia humifusa* Willd.
T rept - Orig. Asia
Literature data: Baroni 1897-1908

Euphorbia lathyris L.
H bienn - Orig. Medit.-Turan.
Literature data: Caruel 1860
Field observations: L. Pinzani, 2020

Euphorbia maculata L.

T rept - Orig. N-America
Literature data: Baroni 1897-1908; Orlandi & Arduini 2010, Arduini & Ercoli 2012, under the name *Chamaesyce maculata* (L.) Small; Lombardi 2015; Lazzeri in Buono et al. 2022
Herbarium data: F. Roma-Marzio, 2016, PI, Herb. Roma-Marzio; F. Roma-Marzio, M. D'Antraccoli, 2017, PI
Field observations: I. Arduini, 2024; H. Öhm, 2024

Euphorbia nutans Lag.
T scap - Orig. N-America
Literature data: Savelli 1915
Field observations: partecipanti all'escursione Wikiplantbase #Parco di Migliarino - San Rossore - Massaciuccoli, 2018

NC **Euphorbia palustris** L.
G rhiz - Eurosiber.
Literature data: Caruel 1860; Corti 1956 [*P. Savi*, s.d., FI; *s. coll.*, s.d., FI]

Euphorbia paralias L.
Ch frut - Eurimedit.-Atl.
Literature data: Corti 1956 [*Levier*, s.d., FI]; Corti 1956, 1970; Garbari 2001; Tomei et al. 2004; Sani & Tomei 2006 [*A. Sani*, 2005, PI]; Bertacchi et al. 2009; Bertacchi et al. 2010; Arduini & Ercoli 2012; Bertacchi & Lombardi 2014a; Bertacchi & Lombardi 2014b
Field observations: R. Righini, 2018; L. Pinzani, 2022

Euphorbia peplis L.
T rept - Eurimedit.
Literature data: Caruel 1860; Corti 1956 [*Levier*, s.d., FI; *A. Tassi*, s.d., FI; *M. Savelli*, s.d., FI]; Gellini et al. 1986; Garbari 2001; Tomei et al. 2004; Sani & Tomei 2006; Bertacchi et al. 2009; Bertacchi & Lombardi 2014b
Herbarium data: L. Pinzani, 2020, Herb. Pinzani

Euphorbia peplus L.
T scap - Eurosiber.-Cosmop.
Literature data: Caruel 1860; Corti 1956; Garbari 2001; Tomei et al. 2004; Arduini & Ercoli 2012; Lombardi 2015; Bonari et al. 2019
Field observations: B. Pierini, 2014; L. Pinzani, 2020, 2021, 2022; I. Arduini, 2023

Euphorbia pithyusa L. subsp. **pithyusa**
Ch suffr - W-Medit.
Literature data: Sani & Tomei 2005 [*A. Sani, P.E. Tomei*, 2005, FI]

Euphorbia platyphyllos L.
T scap - Eurimedit.
Literature data: Caruel 1860; Coaro 1987; Garbari 2001; Lazzeri 2021
Field observations: L. Pinzani, 2021

Euphorbia prostrata Aiton
T rept - Orig. N-America
Literature data: Baroni 1897-1908

Field observations: L. Peruzzi, 2019; L. Pinzani, 2019

Euphorbia serpens Kunth subsp. serpens
T rept - Orig. N-America
Literature data: Garbari 2001
Herbarium data: L. Pinzani, 2019, Herb. Pinzani

Mercurialis annua L.
T scap - Paleotemp.
Literature data: Caruel 1860
Field observations: B. Pierini, 2014; L. Pinzani, 2021, 2022; I. Arduini, 2024

Elatinaceae

NC **Elatine alsinastrum** L.
I rad - Eurasiat.
Literature data: Baroni 1897-1908

Passifloraceae

Passiflora caerulea L.
Field observations: observed on iNaturalist in 2020, 2024
Only cultivated

Salicaceae

Populus alba L.
P scap - Paleotemp.
Literature data: Caruel 1860; Corti 1956, under the name *P. alba* L. subvar. *comesiana* (Dode) Asch. u. Gr.; Gellini et al. 1986; Coaro 1987; Tomei et al. 2004; Arduini & Ercoli 2012; Lombardi 2015; Lazzeri 2021
Herbarium data: A. Chiarugi, 1933, FI; F. Roma-Marzio, M. D'Antraccoli, 2017, PI
Field observations: M. D'Antraccoli, 2018; partecipanti all'escursione Wikiplantbase #Parco di Migliarino - San Rossore - Massaciuccoli, 2018; L. Peruzzi, 2019; L. Pinzani, 2020, 2021, 2022; I. Arduini, 2023, L. Peruzzi, 2024

Populus canescens (Aiton) Sm.
P scap - S-Europ.
Literature data: Coaro 1987; Garbari 2001; Tomei et al. 2004
Field observations: M. D'Antraccoli, 2018; I. Arduini, 2023

Populus deltoides W.Bartram ex Marshall subsp. *deltoides*
Field observations: observed on iNaturalist in 2018
Only cultivated

Populus nigra L. subsp. **nigra**
P scap - Paleotemp.

Literature data: Caruel 1860; Corti 1956, under the name *P. nigra* L. var. *italica* Duroi; Gellini et al. 1986; Coaro 1987; Garbari 2001; Bertacchi et al. 2009; Tomei et al. 2004; Lombardi 2015; Bonari et al. 2019; Lazzeri in Buono et al. 2022
Field observations: L. Pinzani, 2021

Populus tremula L.
P scap - Eurosiber.
Literature data: Baroni 1897-1908; Coaro 1987; Garbari 2001
Field observations: L. Peruzzi, 2024

Populus ×canadensis Moench nothosubsp. *canadensis*
Literature data: Lazzeri in Buono et al. 2022

Salix alba L.
P scap - Paleotemp.
Literature data: Caruel 1860; Coaro 1987; Bertacchi et al. 2010; Lazzeri 2021, 2022
Field observations: M. D'Antraccoli, 2018, L. Peruzzi, 2024

Salix babylonica L.
Field observations: observed on iNaturalist in 2022
Only cultivated

Salix caprea L.
P caesp - Eurasiat.
Literature data: Caruel 1860; Lazzeri in Buono et al. 2022
Herbarium data: s.c., 1863, FI; Parlatore, 1868, FI

Salix cinerea L.
P caesp - Paleotemp.
Literature data: Caruel 1860; Baroni 1897-1908; Coaro 1987; Lazzeri in Buono et al. 2022
Herbarium data: Parlatore, 1871, FI; S. Sommier, 1899, FI; M. Mannocci, 2009, Herb. Mus. Livorno

Salix purpurea L. subsp. **purpurea**
P scap - Eurasiat.
Literature data: Caruel 1860; Lazzeri in Buono et al. 2022

NC **Salix triandra** L. subsp. **triandra**
P caesp - Eurosiber.
Literature data: Caruel 1860, under the name *S. amygdalina* L.

Violaceae

Viola alba Besser subsp. **alba**
H ros - Eurimedit.
Literature data: Coaro 1987
Herbarium data: J. Franzoni, M. Tiburtini, 2019, Herb. Franzoni
Field observations: J. Franzoni, 2019, growing spontaneously inside the Botanic Garden of Pisa.

Viola alba Besser subsp. **dehnhardtii** (Ten.) W.Becker
H ros - Eurimedit.
Literature data: Bonari et al. 2019

Viola arvensis Murray
T scap - Eurimedit.
Literature data: Corti 1956, under the name *V. tricolor* L. subsp. *arvensis* (Murray) Gaudin; Garbari 2001
Herbarium data: L. Pinzani, 2021, Herb. Pinzani

NC **Viola canina** L.
H scap - Eurasiat.
Literature data: Caruel 1860; Baroni 1897-1908, under the name *V. canina* L. subsp. *ruppii* (All.) Schübl. & G.Martens

Viola odorata L.
H ros - Eurimedit.
Literature data: Caruel 1860
Herbarium data: J. Franzoni, 2019, Herb. Franzoni
Field observations: J. Franzoni, 2019; L. Pinzani, 2022

Viola reichenbachiana Jord. ex Boreau
H scap - Eurosiber.
Literature data: Corti 1956, under the name *V. silvatica* Fries; Gellini et al. 1986; Coaro 1987; Garbari 2001; Tomei et al. 2004; Arduini & Ercoli 2012; Lombardi 2015; Bonari et al. 2019
Field observations: I. Arduini, 2024

Viola tricolor L.
T scap/H bienn (H scap) - Eurasiat.
Literature data: Caruel 1860
Field observations: L. Pinzani, 2021

Linaceae

Linum maritimum L. subsp. **maritimum**
H scap - W-Medit.
Literature data: Caruel 1860; Baroni 1897-1908; Corti 1956 [*S. Sommier*, s.d., FI; *A. Tassi*, s.d., FI; *M. Savelli*, s.d., FI]; Coaro 1987; Garbari 2001; Tomei et al. 2004

NC **Linum radiola** L.
T scap - Paleotemp.
Literature data: Caruel 1860, under the name *Radiola linoides* Roth

Linum strictum L.
T scap - Stenomedit.
Literature data: Caruel 1860; Garbari 2001
Herbarium data: L. Pinzani, 2020, Herb. Pinzani

Linum tenuifolium L.
Ch suffr - Submedit.-Pont.
Literature data: Garbari 2001
Herbarium data: Partecipanti all'escursione Wikiplantbase #Parco di Migliarino - San Rossore - Massaciuccoli, 2018, PI

Linum trigynum L.
T scap - Eurimedit.
Literature data: Caruel 1860, under the name *L. gallicum* L.; Garbari 2001

Linum usitatissimum L. subsp. **angustifolium** (Huds.) Thell.
H bienn - Eurimedit.
Literature data: Caruel 1860, under the name *L. angustifolium* L.; Corti 1956 [*A. Fiori*, s.d., FI]; Corti 1956, under the name *L. angustifolium* Huds. var. *imperforatum* Strobl.; Coaro 1987, Garbari 2001, Saggese 2016, under the name *L. bienne* Miller
Herbarium data: Partecipanti all'escursione Wikiplantbase #Parco di Migliarino - San Rossore - Massaciuccoli, 2018, PI
Field observations: partecipanti all'escursione Wikiplantbase #Parco di Migliarino - San Rossore - Massaciuccoli, 2018; A. Mo, 2022; L. Pinzani, 2022

Linum usitatissimum L. subsp. *usitatissimum*
Herbarium data: M.L. Pedullà, 1999, PI

Hypericaceae

Hypericum androsaemum L.
NP - Eurimedit.
Literature data: Corti 1956; Gellini et al. 1986; Garbari 2001; Tomei et al. 2004; Arduini & Ercoli 2012
Field observations: I. Arduini, 2022

NC **Hypericum australe** Ten.
H scap - Stenomedit.-Occid.
Literature data: Caruel 1860

NC **Hypericum elodes** L.
H scap/I rad - Atl.-Europ.
Literature data: Corti 1956; Garbari 2001
The only still surviving population of *Hypericum elodes* in Italy is found just outside the study area (Bedini et al. 2011), within the San Giuliano Terme municipality (province of Pisa).

Hypericum humifusum L.
H scap - Subcosmop.
Literature data: Caruel 1860; Corti 1956; Arduini & Ercoli 2012; Lombardi 2015
Herbarium data: G. Gestri, 2018, PI; Partecipanti all'escursione Wikiplantbase #Parco di Migliarino - San Rossore - Massaciuccoli, 2018, PI

Hypericum montanum L.
H caesp - Europ.-Caucas.

Literature data: Corti 1956, under the name *H. montanum* L. var. *scabrum* Koch; Gellini et al. 1986; Coaro 1987; Garbari 2001

Hypericum mutilum L. subsp. mutilum
T scap - Orig. N-Amer.
Literature data: Lombardi 2015

Hypericum perfoliatum L.
H scap - Stenomedit.
Literature data: Garbari 2001; Lombardi 2015

Hypericum perforatum L. subsp. **perforatum**
H scap - Paleotemp.
Literature data: Caruel 1860; Corti 1956 [*s. coll.*, s.d., FI]; Corti, 1951, 1956, under the name *H. perforatum* L. var. *vulgare* Neilr.; Coaro 1987; Garbari 2001; Tomei et al. 2004; Arduini & Ercoli 2012; Lombardi 2015; Saggese 2016; Bonari et al. 2019; Lazzeri 2021
Field observations: L. Peruzzi, 2019; L. Pinzani, 2020, 2022

Hypericum perforatum L. subsp. **veronense** (Schrank) Ces.
H scap - Paleotemp.
Herbarium data: F. Roma-Marzio, M. D'Antraccoli, 2017, PI; Partecipanti all'escursione Wikiplantbase #Parco di Migliarino - San Rossore - Massaciuccoli, 2018, PI
Field observations: partecipanti all'escursione Wikiplantbase #Parco di Migliarino - San Rossore - Massaciuccoli, 2018

Hypericum tetrapterum Fr.
H scap - Paleotemp.
Field observations: I. Arduini, 2024

Geraniaceae

NC **Erodium alnifolium** Guss.
T scap - W-Medit.
Literature data: Baroni 1897-1908

Erodium botrys (Cav.) Bertol.
T scap - Stenomedit.
Literature data: Caruel 1860; Corti 1956 [*G. Savi*, s.d., FI; *P. Savi*, s.d., FI; *O. Beccari*, s.d., FI; *P. Fantozzi*, s.d., FI]; Corti 1956; Garbari 2001
Herbarium data: L. Pinzani, 2022, Herb. Pinzani

Erodium cicutarium (L.) L'Hér.
T scap - Subcosmop.
Literature data: Caruel 1860; Corti 1956 [*G. Savi*, s.d., FI]; Corti 1956, under the name *E. cicutarium* (L.) L'Hér var. *immaculatum* Koch; Coaro 1987; Garbari 2001; Tomei et al. 2004; Sani & Tomei 2006; Lombardi 2015
Herbarium data: A. Sani, 2005, PI; L. Pinzani, 2020, Herb. Pinzani
Field observations: L. Pinzani, 2022

Erodium malacoides (L.) L'Hér. subsp. **malacoides**
T scap/H bienn - Medit.-Macarones.
Literature data: Caruel 1860; Saggese 2016
Field observations: L. Pinzani, 2021, 2022

NC **Erodium maritimum** L'Hér.
H bienn/T scap - W-Europ.
Literature data: Caruel 1860

Erodium moschatum (L.) L'Hér.
T scap/H bienn - Eurimedit.
Herbarium data: L. Pinzani, 2021, Herb. Pinzani
Field observations: L. Pinzani, 2022

Geranium columbinum L.
T scap - Eurosiber.
Literature data: Caruel 1860; Corti 1956 [*Picciuoli*, FI]; Coaro 1987; Bonari et al. 2019
Herbarium data: Partecipanti all'escursione Wikiplantbase #Parco di Migliarino - San Rossore - Massaciuccoli, 2018, PI

Geranium dissectum L.
T scap - Eurasiat.
Literature data: Caruel 1860; Corti 1956, under the name *G. dissectum* L. forma *furcatum* Schur.; Coaro 1987; Garbari 2001; Bertacchi et al. 2009; Saggese 2016; Bonari et al. 2019
Field observations: B. Pierini, 2014; L. Peruzzi, 2019; L. Pinzani, 2021, 2022; A. Mo, 2022; I. Arduini, 2024; H. Öhm, 2024

Geranium molle L.
T scap - Eurasiat.
Literature data: Corti 1956, under the name *G. molle* L. var. *typicum* Maly; Coaro 1987; Tomei et al. 2004; Lombardi 2015
Herbarium data: A. Sani, 2005, PI; L. Pinzani, 2020, Herb. Pinzani
Field observations: B. Pierini, 2014; partecipanti all'escursione Wikiplantbase #Parco di Migliarino - San Rossore - Massaciuccoli, 2018; L. Pinzani, 2021, 2022; I. Arduini, 2024

Geranium purpureum Vill.
T scap - Eurimedit.
Literature data: Corti 1956 [*P. Savi*, s.d., FI]; Corti 1956, under the name *G. robertianum* L. var. *purpureum* (Vill.) DC.; Gellini et al. 1986; Garbari 2001; Bonari et al. 2019
Herbarium data: A. Sani, 2005, PI
Field observations: B. Pierini, 2014, 2015; partecipanti all'escursione Wikiplantbase #Parco di Migliarino - San Rossore - Massaciuccoli, 2018; L. Pinzani, 2021, 2022; I. Arduini, 2023

Geranium pusillum L.
T scap - Europ.-W-Asiat.
Literature data: Garbari 2001

Geranium robertianum L.
T scap - Subcosmop.

Literature data: Caruel 1860; Corti, 1951; Coaro 1987; Tomei et al. 2004; Bertacchi & Lombardi 2016; Bonari et al. 2019
Field observations: I. Arduini, 2024

Geranium rotundifolium L.
T scap - Paleotemp.
Literature data: Caruel 1860; Corti 1956
Herbarium data: F. Roma-Marzio, L. Peruzzi, 2019, PI
Field observations: partecipanti all'escursione Wikiplantbase #Parco di Migliarino - San Rossore - Massaciuccoli, 2018; J. Franzoni, 2019; L. Pinzani, 2021, 2022

Geranium sylvaticum L.
H scap - Eurasiat.
Literature data: Garbari 2001; Tomei et al. 2004

Pelargonium ribifolium Jacq.
Field observations: observed on iNaturalist in 2022
Only cultivated

Lythraceae

Lagerstroemia indica L.
Field observations: observed on iNaturalist in 2022, 2024
Only cultivated

Lythrum hyssopifolia L.
T scap - Subcosmop.
Literature data: Caruel 1860; Corti 1956; Lazzeri in Buono et al. 2022
Herbarium data: Partecipanti all'escursione Wikiplantbase #Parco di Migliarino - San Rossore - Massaciuccoli, 2018, PI
Field observations: L. Pinzani, 2021

Lythrum salicaria L.
H scap - Subcosmop.
Literature data: Caruel 1860; Corti 1956; Gellini et al. 1986; Coaro 1987; Garbari 2001; Pedullà & Garbari, 2004; Tomei et al. 2004; Petraglia 2013; Saggese 2016; Lazzeri in Buono et al. 2022
Field observations: L. Peruzzi, 2013; B. Pierini, 2014; partecipanti all'escursione Wikiplantbase #Parco di Migliarino - San Rossore - Massaciuccoli, 2018; L. Pinzani, 2021, 2022

Lythrum tribracteatum Salzm. ex Spreng.
T scap - Eurimedit.
Literature data: Caruel 1860, under the name *L. bibracteatum* Salzm.; Baroni 1897-1908; Coaro 1987; Petraglia 2013
Herbarium data: L. Pinzani, 2020, Herb. Pinzani

NC **Middendorfia borysthenica** (Schrank) Trautv.
T scap - Submedit.
Literature data: Baroni 1897-1908, under the name *Peplis erecta* Reg. ex Benth.

Punica granatum L.
Field observations: observed on iNaturalist in 2022, 2024
Only cultivated

Onagraceae

Circaea lutetiana L. subsp. **lutetiana**
H scap - Circumbor.
Literature data: Baroni 1897-1908; Corti 1956; Gellini et al. 1986; Coaro 1987

Epilobium hirsutum L.
H scap - Subcosmop.
Literature data: Caruel 1860; Lazzeri in Buono et al. 2022
Herbarium data: J. Franzoni, 2019, Herb. Franzoni
Field observations: B. Pierini, 2014

NC **Epilobium parviflorum** Schreb.
H scap - Paleotemp.
Literature data: Caruel 1860

Epilobium tetragonum L. subsp. **lamyi** (F.W.Schultz) Nyman
H scap - Paleotemp.
Literature data: Peruzzi et al. 2021 [*L. Pinzani*, 2020, PI]

Epilobium tetragonum L. subsp. **tetragonum**
H scap - Paleotemp.
Literature data: Caruel 1860; Lazzeri in Buono et al. 2022
Herbarium data: L. Pinzani, 2020, Herb. Pinzani
Field observations: B. Pierini, 2014

NC **Ludwigia palustris** (L.) Elliott
H caesp/T rept - Subcosmop.
Literature data: Caruel 1860; Corti 1956 [*A. Biondi*, FI]

Ludwigia peploides (Kunth) P.H.Raven subsp. montevidensis (Spreng.) P.H.Raven
H caesp - Orig. America
Literature data: Peruzzi 2021
Field observations: L. Peruzzi, 2023

Ex *Oenothera biennis* L.
Literature data: Corti 1956; Garbari 2001

Oenothera chicaginensis de Vries ex Renner & Cleland
H bienn - Orig. America
Literature data: Saggese 2016; Lazzeri 2014, 2022
Field observations: B. Pierini, 2014; V. Lazzeri, 2017

Oenothera indecora Cambess.
H bienn/T scap - Orig. S-America

Literature data: Lombardi 2015; Peruzzi et al. 2017b [*A. Sani*, 2015, PI]

Oenothera italica Rostanski & Soldano
H bienn - Orig. Europ.
Literature data: Soldano 1993

NC *Oenothera laciniata* Hill
Literature data: Corti, 1954, 1956, under the name *O. sinuata* L.

Oenothera latipetala (Soldano) Soldano
Herbarium data: F. Roma-Marzio et G. Astuti, 2017, PI

Oenothera lindheimeri (Engelm. & A.Gray) W.L.Wagner & Hoch
Field observations: L. Peruzzi, 2018
Only cultivated.

Oenothera stucchii Soldano
H bienn - Orig. Europ.
Field observations: G. Cataldi, 2019

Myrtaceae

Eucalyptus camaldulensis Dehnh. subsp. camaldulensis
P scap - Orig. Australia
Field observations: partecipanti all'escursione Wikiplantbase #Parco di Migliarino - San Rossore - Massaciuccoli, 2018

Feijoa sellowiana (O.Berg) O.Berg
Field observations: observed on iNaturalist in 2022
Only cultivated

Melaleuca citrina (Curtis) Dum.Cours.
Field observations: observed on iNaturalist in 2018, 2024
Only cultivated

Myrtus communis L.
P caesp - Stenomedit.
Literature data: Caruel 1860; Corti 1956, under the name *M. communis* L. var. *italica* L.; Coaro 1987 [*E. Coaro*, 1984, PI; *G. Pistolesi, E. Coaro*, 1984, PI]; Garbari 2001 [*B. Ciacchi, G. Lorè*, 1996, 1997, PI]; Tomei et al. 2004; Bertacchi et al. 2010; Arduini & Ercoli 2012; Bertacchi & Lombardi 2014a
Herbarium data: R. Poli, 1989, PI
Field observations: partecipanti all'escursione Wikiplantbase #Parco di Migliarino - San Rossore - Massaciuccoli, 2018; A. Mo, 2022

Anacardiaceae

Pistacia lentiscus L.
P caesp - Stenomedit.

Literature data: Corti 1956; Coaro 1987; Garbari 2001; Tomei et al. 2004; Lazzeri 2021
Herbarium data: Parlatore, 1871, FI
Field observations: partecipanti all'escursione Wikiplantbase #Parco di Migliarino - San Rossore - Massaciuccoli, 2018; A. Mo, 2022

NC **Pistacia terebinthus** L. subsp. **terebinthus**
P caesp/P scap - Eurimedit.
Herbarium data: Parlatore, 1871, FI

Sapindaceae

Acer campestre L.
P scap - Europ.-Caucas.
Literature data: Corti 1956, under the name *A. campestre* L. subsp. *hebecarpum* DC. var. l*obatum* Pax forma *affine* Opiz; Gellini et al. 1986; Coaro 1987; Tomei et al. 2004; Lombardi 2015; Bertacchi & Lombardi 2016
Herbarium data: C. Del Prete, 1980, PI; R. Poli, 1989, PI
Field observations: L. Peruzzi, 2017; partecipanti all'escursione Wikiplantbase #Parco di Migliarino - San Rossore - Massaciuccoli, 2018; F. Roma-Marzio, 2019; L. Pinzani, 2020; L. Pinzani, 2020; I. Arduini, 2022; L. Pinzani, 2022

Acer cappadocicum Gled. subsp. *lobelii* (Ten.) A.E.Murray
Field observations: observed on iNaturalist in 2022
Only cultivated

Acer negundo L.
P scap - Orig. N-America
Literature data: Bertacchi et al. 2010
Field observations: L. Pinzani, 2020; L. Pinzani, 2021

Aesculus hippocastanum L.
Literature data: Corti 1956; Garbari 2001
Field observations: M. D'Antraccoli, 2019
Only cultivated

Rutaceae

Citrus trifoliata L.
Field observations: observed on iNaturalist in 2023
Only cultivated

Ruta chalepensis L.
Ch suffr - S-Medit.
Literature data: Garbari 2001
Herbarium data: F. Roma-Marzio, M. D'Antraccoli, 2017, PI; F. Roma-Marzio, L. Peruzzi, 2019, PI
Field observations: L. Peruzzi, 2019

Simaroubaceae

Ailanthus altissima (Mill.) Swingle
P scap - Orig. E-Asia
Literature data: Corti 1956, under the name *A. glandulosa* Desf.; Garbari 2001; Tomei et al. 2004; Arduini & Ercoli 2012
Herbarium data: F. Ruggiero, 2007, PI
Field observations: L. Peruzzi, 2015; 2017, 2018; L. Pinzani, 2020, 2021, 2022; F. Roma-Marzio, 2021; I. Arduini, 2023, 2024

Meliaceae

Melia azedarach L.
Field observations: I. Arduini, 2024

Cytinaceae

NC **Cytinus hypocistis** (L.) L. subsp. **hypocistis**
G rad - Medit.-Macarones.
Literature data: Corti 1956 [*Rossetti, Pelegrini*, s.d., FI]

Malvaceae

Abutilon theophrasti Medik.
T scap - Orig. S-Siber.
Literature data: Tomei et al. 1986 [*N. Passerini*, 1909, PI]
Field observations: B. Pierini, 2014

Althaea cannabina L.
H scap - S-Europ.-W-Asiat.
Literature data: Caruel 1860
Herbarium data: Partecipanti all'escursione Wikiplantbase #Parco di Migliarino - San Rossore - Massaciuccoli, 2018, PI

Althaea officinalis L.
H scap - Subcosmop.
Literature data: Caruel 1860; Corti 1956, under the name *A. officinalis* L. var. *officinalis*; Coaro 1987; Garbari 2001; Pedullà & Garbari, 2004; Tomei et al. 2004; Sani & Tomei 2006
Field observations: B. Pierini, 2006

NC *Hibiscus trionum* L.
Literature data: Baroni 1987-1908

Hibiscus rosa-sinensis L.
Field observations: observed on iNaturalist in 2022
Only cultivated

Hibiscus syriacus L.
Field observations: observed on iNaturalist in 2021, 2023
Only cultivated

Malope malacoides L. subsp. **malacoides**
T scap/H scap - Stenomedit.-W-Asiat.
Herbarium data: M. Tiburtini, 2019, Herb. Tiburtini

Malva arborea (L.) Webb & Berthel.
H bienn - Stenomedit.
Literature data: Caruel 1860, under the name *Lavatera arborea* L.
Herbarium data: L. Pinzani, 2019, Herb. Pinzani
Field observations: B. Pierini, 2013; L. Pinzani, 2020, 2021, 2022
This species was described by Linnaeus (1753) for an area comprises between Pisa and Livorno (Peruzzi et al. 2019).

Malva multiflora (Cav.) Soldano, Banfi & Galasso
T scap - Stenomedit.
Literature data: Baroni 1897-1908, under the name *Lavatera cretica* L.
Herbarium data: L. Pinzani, 2020, 2021, Herb. Pinzani
Field observations: B. Pierini, 2020

Malva nicaeensis All.
T scap/H bienn - Steno-Medit.
Field observations: observed on iNaturalist in 2024

Malva parviflora L.
T scap - Eurimedit.
Herbarium data: L. Pinzani, 2022, Herb. Pinzani

Malva punctata (All.) Alef.
T scap - Stenomedit.
Literature data: Caruel 1860, Pedullà & Garbari, 2004, under the name *Lavatera punctata* All.; Saggese 2016
Field observations: L. Pinzani, 2021

Malva sylvestris L.
H scap/T scap - Eurosiber.-Subcosmop.
Literature data: Caruel 1860; Corti 1956; Garbari 2001; Pedullà & Garbari, 2004; Saggese 2016; Lazzeri in Buono et al. 2022
Field observations: B. Pierini, 2014; F. Roma-Marzio, 2014; partecipanti all'escursione Wikiplantbase #Parco di Migliarino - San Rossore - Massaciuccoli, 2018; L. Peruzzi, 2019; L. Pinzani, 2021, 2022

Tilia americana L.
Field observations: observed on iNaturalist in 2023, 2024
Only cultivated

Thymelaeaceae

Daphne gnidium L.
P caesp - Stenomedit.

Literature data: Caruel 1860; Corti, 1951, 1956; Corti 1956 [*P. Savi*, s.d., FI]; Garbari 2001 [*B. Ciacchi*, *G. Lorè*, 1996, PI]; Tomei et al. 2004; Sani & Tomei 2006; Bertacchi et al. 2010; Arduini & Ercoli 2012; Bertacchi & Lombardi 2014a; Lombardi 2015; Bonari et al. 2019
Herbarium data: G. Savi, 1839, FI; s. coll., 1868, FI; M. Savelli, 1913; A. Chiarugi, R. Corti, 1951, FI; F. Roma-Marzio, M. D'Antraccoli, 2017, PI
Field observations: A. Mo, 2022; L. Pinzani, 2022

Daphne laureola L.
P caesp - Submedit.-Subatl.
Herbarium data: G. Cela Renzoni, 1968, FI

NC **Thymelaea passerina** (L.) Coss. & Germ.
T scap - Eurimedit.-Centroasiat.
Literature data: Caruel 1860, under the name *Passerina annua* Wikstr.

Cistaceae

Cistus creticus L. subsp. **eriocephalus** (Viv.) Greuter & Burdet
NP - Medit.-Pont.
Literature data: Coaro 1987 [*G. Pistolesi*, *E. Coaro*, 1977, PI; *E. Coaro*, 1984, PI], under the name *C. incanus* L.; Bertacchi et al. 2010; Bertacchi & Lombardi 2014a
Herbarium data: A. Chiarugi, 1933; G. Moggi, 1958, FI; C. Ricceri, A. Benini, 1985, FI; R. Bavazzano, C. Ricceri, 1963, FI
Field observations: L. Pinzani, 2020

Cistus monspeliensis L.
NP - Stenomedit.
Literature data: Corti 1956; Garbari 2001
Field observations: U. Macchia, 2016

Cistus salviifolius L.
NP - Stenomedit.
Literature data: Corti, 1951, 1956; Coaro 1987 [*G. Pistolesi*, *E. Coaro*, 1977, PI; *E. Coaro*, 1984, PI]; Garbari 2001 [*B. Ciacchi*, *G. Lorè*, 1998, PI]; Tomei et al. 2004; Bertacchi et al. 2010; Arduini & Ercoli 2012; Bertacchi & Lombardi 2014a; Lombardi 2015; Bonari et al. 2019
Herbarium data: A. Chiarugi, R. Corti, 1951, FI; G. Moggi, 1958, FI
Field observations: L. Peruzzi, 2017; L. Pinzani, 2021, 2022

Fumana procumbens (Dunal) Gren. & Godr.
Ch suffr - Eurimedit.-Pont.
Literature data: Garbari 2001

Helianthemum nummularium (L.) Mill. subsp. **obscurum** (Čelak.) Holub
Ch suffr - Europ.-Caucas.
Literature data: Coaro 1987; Garbari 2001, also under the name *Helianthemum nummularium* (L.) Mill.

Helianthemum salicifolium (L.) Mill.
T scap - Eurimedit.

Literature data: Garbari 2001; Bonari et al. 2019

Tuberaria guttata (L.) Fourr.
T scap - Eurimedit.
Literature data: Caruel 1860, Corti, 1951, 1956, under the name *Helianthemum guttatum* Mill.; Corti 1956 [*A. Biondi*, FI; *Levier*, FI]; Coaro 1987; Garbari 2001; Tomei et al. 2004; Sani & Tomei 2006; Arduini & Ercoli 2012; Lombardi 2015
Field observations: A. Mo, 2023

Resedaceae

Reseda alba L. subsp. **alba**
H scap/T scap - Stenomedit.
Literature data: Peruzzi et al. 2017b [*F. Roma-Marzio, M. D'Antraccoli*, 2017, PI]
Herbarium data: Partecipanti all'escursione Wikiplantbase #Parco di Migliarino - San Rossore - Massaciuccoli, 2018, PI

NC **Reseda luteola** L.
H scap/T scap - Circumbor.
Literature data: Caruel 1860

NC **Reseda phyteuma** L. subsp. **phyteuma**
H scap/T scap - Eurimedit.-Stenomedit.
Literature data: Caruel 1860

Capparaceae

Capparis spinosa L. subsp. **rupestris** (Sm.) Nyman
NP - Stenomedit.
Literature data: Caruel 1860
Herbarium data: J. Campani, 1857, SIENA
Field observations: F. Roma-Marzio, 2019, 2021; Pinzani 2022

Brassicaceae

Alliaria petiolata (M.Bieb.) Cavara & Grande
H bienn - Paleotemp.
Literature data: Garbari 2001; Pedullà & Garbari, 2004; Lombardi 2015
Herbarium data: J. Franzoni, M. Franzoni et D. Scalzo, 2019, Herb. Franzoni
Field observations: L. Pinzani, 2022; I. Arduini, 2023

NC **Alyssum simplex** Rudolphi
T scap - Medit.-Turan.
Literature data: Baroni 1897-1908, under the name *A. campestre* (L.) L. subsp. *campestre*

Arabidopsis thaliana (L.) Heynh.
T scap - Cosmop.
Literature data: Caruel 1860, under the name *Sisymbrium thalianum* Gay
Field observations: L. Pinzani, 2021, 2022

NC **Barbarea verna** (Mill.) Asch.
H scap - W-Europ.
Literature data: Caruel 1860, under the name *B. praecox* R.Brown

NC **Barbarea vulgaris** W.T.Aiton
H scap - Eurosiber.-Cosmop.
Literature data: Caruel 1860

Brassica oleracea L.
Literature data: Coaro 1987

Brassica nigra (L.) W.D.J.Koch
T scap - Orig. Medit.
Literature data: Caruel 1860, under the name *B. sinapioides* Roth; Baroni 1897-1908
Field observations: B. Pierini 2017; L. Peruzzi, 2019

Brassica rapa L. subsp. **campestris** (L.) A.R.Clapham
H scap/T scap - Europ.
Field observations: L. Pinzani, 2022

Bunias erucago L.
T scap - N-Medit.
Literature data: Caruel 1860; Coaro 1987; Saggese 2016
Field observations: B. Pierini 2016; L. Pinzani, 2021

Cakile maritima Scop. subsp. **maritima**
T scap - Medit.-Atl.
Literature data: Caruel 1860; Corti 1956; Coaro 1987; Garbari 2001; Tomei et al. 2004; Sani & Tomei 2006; Bertacchi et al. 2009; Bertacchi et al. 2010; Bertacchi & Lombardi 2014a; Bertacchi & Lombardi 2014b
Herbarium data: L. Pinzani, 2019, Herb. Pinzani
Field observations: partecipanti all'escursione Wikiplantbase #Parco di Migliarino - San Rossore - Massaciuccoli, 2018

Calepina irregularis (Asso) Thell.
T scap - Medit.-Turan.
Field observations: L. Pinzani, 2022

Capsella bursa-pastoris (L.) Medik. subsp. **bursa-pastoris**
H bienn - Cosmop.
Literature data: Caruel 1860; Coaro 1987; Garbari 2001
Field observations: B. Pierini 2014; partecipanti all'escursione Wikiplantbase #Parco di Migliarino - San Rossore - Massaciuccoli, 2018; L. Pinzani, 2021, 2022; J. Franzoni, 2023; I. Arduini, 2024

Capsella rubella Reut.
T scap - Eurimedit.
Field observations: L. Pinzani, 2022

NC **Cardamine amporitana** Sennen & Pau
T scap - W-Medit.
Literature data: Caruel 1860, Corti 1956, under the name *C. amara* L.

Cardamine apennina Lihová & Marhold
H scap - Endem. Ital.
Literature data: Caruel 1860, Corti 1956 [*P. Savi*, s.d., FI; *F. Parlatore*, s.d., FI; *P. Fantozzi*, s.d., FI; *A. Tassi*, s.d., FI; *s. coll.*, s.d., FI], Gellini et al. 1986, Coaro 1987, Garbari 2001, Tomei et al. 2004, Lombardi 2015, under the name *C. pratensis* L.; Corti 1956, under the name *C. pratensis* L. subsp. *granulosa* (All.) O.E.Schulz
Herbarium data: C. Angiolini, G. Bonari, M. Chytrý, 2016, SIENA
Field observations: I. Arduini, 2023
Assessed as EN in the Red List of the Italian Vascular Flora (Rossi et al. 2013)

Cardamine hirsuta L.
T scap - Cosmop.
Literature data: Corti 1956; Coaro 1987; Garbari 2001; Tomei et al. 2004; Arduini & Ercoli 2012
Herbarium data: F. Roma-Marzio, M. D'Antraccoli, 2017, PI; F. Roma-Marzio, L. Peruzzi, 2019, PI
Field observations: B. Pierini 2014; J. Franzoni, 2019; L. Pinzani, 2021, 2022; I. Arduini, 2023

Cardamine occulta Hornem
H bienn - SE-Asiat.
Field observations: observed on iNaturalist in 2016, 2020, 2024

Cardamine parviflora L.
T scap - Eurosiber.
Literature data: Caruel 1860; Baroni 1897-1908; Corti 1956 [*O. Beccari*, s.d., FI]
Field observations: B. Pierini 2014

NC *Conringia orientalis* (L.) Andrz. ex DC.
T scap - Orig. E-Asia
Literature data: Baroni 1897-1908

Descurainia sophia (L.) Webb ex Prantl
H bienn/T scap - Eurasiat.-Paleotemp.
Literature data: Garbari 2001

Diplotaxis erucoides (L.) DC. subsp. **erucoides**
T scap - Steno-Medit.-Occid.
Field observations: observed on iNaturalist in 2023

Diplotaxis muralis (L.) DC.
T scap (H scap) - N-Medit.-Atl.
Literature data: Coaro 1987

Diplotaxis tenuifolia (L.) DC.
T scap - Submedit.
Literature data: Saggese 2016; Lazzeri in Buono et al. 2022

Field observations: B. Pierini, 2015; L. Peruzzi, 2019

Draba verna L. subsp. **praecox** (Steven) Rouy & Foucaud
T scap - Circumbor.
Literature data: Garbari 2001, under the name *Erophila verna* (L.) DC. subsp. *praecox* (Steven) Walp.
Field observations: L. Pinzani, 2021

Draba verna L. subsp. **verna**
T scap - Circumbor.
Literature data: Caruel 1860
Field observations: L. Pinzani, 2022

NC **Drabella muralis** (L.) Fourr.
T scap - Circumbor.
Literature data: Caruel 1860, under the name *Draba muralis* L.

Eruca vesicaria (L.) Cav.
T scap - Medit.-Turan.
Literature data: Baroni 1897-1908, under the name *E. vesicaria* (L.) Cav. subsp. *sativa* (Mill.) Thell.

NC *Erysimum cheiri* (L.) Crantz
Literature data: Caruel 1860, under the name *Cheiranthus cheiri* L.

NC **Hornungia petraea** (L.) Rchb. subsp. **petraea**
T scap - Eurimedit.
Literature data: Caruel 1860, under the name *Hutchinsia petraea* R.Brown.; Corti 1956 [*A. Tassi*, FI]

NC **Iberis pinnata** L.
T scap - Eurimedit.-Sett.
Literature data: Baroni 1897-1908

Isatis tinctoria L. subsp. **tinctoria**
H bienn - S-Europ.-S-Siber.
Herbarium data: L. Pinzani, 2022, Herb. Pinzani
Field observations: B. Pierini, 2016

NC **Lepidium campestre** (L.) W.T.Aiton
T scap - Europ.-Caucas.
Literature data: Caruel 1860

NC **Lepidium coronopus** (L.) Al-Shehbaz
T scap - Eurimedit.-Subcosmop.
Literature data: Caruel 1860, under the name *Senebiera coronopus* Poir.

Lepidium didymum L.
T rept - Orig. N-America

Literature data: Caruel 1860, under the name *Senebiera pinnatifida* Dec.
Herbarium data: F. Roma-Marzio, 2015, PI; F. Roma-Marzio, L. Peruzzi, 2019, PI; L. Pinzani, 2020, Herb. Pinzani
Field observations: B. Pierini, 2020; J. Franzoni, 2020; L. Pinzani, 2020, 2022

Lepidium draba L. subsp. **draba**
G rhiz/H scap - Medit.-Turan.
Literature data: Caruel 1860; Coaro 1987, under the name *Cardaria draba* (L.) Desv.
Field observations: B. Pierini, 2014; J. Franzoni, 2020; L. Pinzani, 2020, 2021

NC **Lepidium graminifolium** L. subsp. **graminifolium**
H scap - Eurimedit.
Literature data: Caruel 1860

Lobularia maritima (L.) Desv.
Ch suffr/H scap - Stenomedit.
Literature data: Caruel 1860
Herbarium data: L. Pinzani, 2019, Herb. Pinzani; F. Roma-Marzio, L. Peruzzi, 2019, PI
Field observations: L. Pinzani, 2021; F. Roma-Marzio, 2021

Lunaria annua L.
H scap - SE-Europ.
Literature data: Coaro 1987
Field observations: L. Pinzani, 2020, 2022

Marcus-kochia ramosissima (Desf.) Al-Shehbaz
T scap - W-Medit.
Literature data: Caruel 1860, Baroni 1897-1908, Corti 1956 [*A. Targioni Tozzetti*, s.d., FI; *P. Savi*, s.d., FI; *O. Beccari*, s.d., FI; *Levier*, s.d., FI; *C. Rossetti*, s.d., FI; *P. Fantozzi*, s.d., FI; *A. Tassi*, s.d., FI; *G. Giacomini*, s.d., FI; *s. coll.*, FI], Corti, 1970, under the name *Malcomia parviflora* Dec.; Corti 1956, Garbari 2001, Tomei et al. 2004, Sani & Tomei 2006, Sani & Dell'Orso 2014 [*A. Sani*, *R. dell'Orso*, 2012, PI], Lombardi 2015, under the name *Malcolmia ramosissima* (Desf.) Thell.
Herbarium data: A. Sani, 2005, PI; L. Pinzani, A. Giacò, J. Franzoni, 2021, Herb. Pinzani
Field observations: F. Roma-Marzio, 2012; L. Pinzani, 2021

Matthiola incana (L.) W.T.Aiton subsp. **incana**
Ch suffr - Stenomedit.
Herbarium data: L. Pinzani, 2019, Herb. Pinzani

NC **Microthlaspi perfoliatum** (L.) F.K.Mey.
T scap - Paleotemp.
Literature data: Baroni 1897-1908, under the name *Thlaspi perfoliatum* L.

NC **Moricandia arvensis** (L.) DC.
T scap - S-Medit.
Literature data: Baroni 1897-1908; Corti 1956 [*s. coll.*, s.d., FI]

NC **Mummenhoffia alliacea** (L.) Esmailbegi & Al-Shehbaz
T scap - S-Europ.

Literature data: Caruel 1860, under the name *Thlaspi alliaceum* L.; Corti 1956 [*G. Savi*, s.d., FI]

Myagrum perfoliatum L.
T scap - SW-Asiat.
Literature data: Caruel 1860; Corti 1956; Garbari 2001
Field observations: M. D'Antraccoli, 2019

Nasturtium officinale W.T.Aiton
H scap - Cosmop.
Literature data: Caruel 1860
Field observations: L. Pinzani, 2022

NC **Neslia paniculata** (L.) Desv. subsp. **paniculata**
T scap - Medit.-Turan.
Literature data: Baroni 1897-1908

Raphanus raphanistrum L. subsp. **landra** (Moretti ex DC.) Bonnier & Layens
T scap - Circumbor.
Literature data: Sani & Tomei 2006; Saggese 2016
Herbarium data: Partecipanti all'escursione Wikiplantbase #Parco di Migliarino - San Rossore - Massaciuccoli, 2018, PI
Field observations: L. Pinzani, 2022

Raphanus raphanistrum L. subsp. **raphanistrum**
T scap - Circumbor.
Literature data: Caruel 1860; Coaro 1987; Garbari 2001; Bertacchi et al. 2010; Bertacchi & Lombardi 2014a
Field observations: partecipanti all'escursione Wikiplantbase #Parco di Migliarino - San Rossore - Massaciuccoli, 2018; L. Pinzani, 2021

Rapistrum rugosum (L.) All.
T scap - Eurimedit.
Literature data: Caruel 1860; Coaro 1987
Field observations: A. Mo, I. Pucci, S. Pascacaldi, L. Pinzani, 2021, 2022

Rorippa amphibia (L.) Besser
H scap - Eurosiber.
Literature data: Caruel 1860, under the name *Nasturtium amphibium* R.Brown
Herbarium data: F. Roma-Marzio, M. D'Antraccoli, 2015, PI

NC **Rorippa palustris** (L.) Besser
T scap - Subcosmop.
Literature data: Baroni 1897-1908, under the name *Nasturtium palustre* DC.

NC **Rorippa prostrata** (J.P.Bergeret) Schinz & Thell.
H scap - Centroeurop.-Subatl.
Literature data: Baroni 1897-1908, under the name *Nasturtium anceps* Reich.

Rorippa sylvestris (L.) Besser subsp. **sylvestris**

H scap - Eurasiat.
Literature data: Baroni 1897-1908, under the name *Nasturtium silvestre* R.Br.
Field observations: B. Pierini, 2015; G. Bedini, 2020; J. Franzoni, 2020

Sinapis alba L. subsp. **alba**
T scap - Eurimedit.
Field observations: B. Pierini, 2015, 2017; partecipanti all'escursione Wikiplantbase #Parco di Migliarino - San Rossore - Massaciuccoli, 2018; L. Pinzani, 2022

Sinapis arvensis L. subsp. **arvensis**
T scap - Stenomedit.
Literature data: Corti 1956; Garbari 2001; Saggese 2016
Field observations: L. Pinzani, 2021, 2022

Sinapis pubescens L. subsp. **pubescens**
Ch suffr - SW-Medit.
Literature data: Garbari 2001

Sisymbrium irio L.
T scap - Paleotemp.
Literature data: Pierini in Peruzzi et al. 2013 [*B. Pierini*, 2013, PI]

Sisymbrium officinale (L.) Scop.
T scap - Eurosiber.
Literature data: Caruel 1860; Coaro 1987
Field observations: B. Pierini, 2020; L. Pinzani, 2022

Sisymbrium polyceratium L.
T scap - Eurimedit.
Literature data: Caruel 18602; Garbari 2001; Tomei et al. 2004; Sani & Tomei 2006

NC **Teesdalia coronopifolia** (J.P.Bergeret) Thell.
T scap - Eurimedit.
Literature data: Caruel 1860, under the name *T. regularis* Smith; Corti 1956 [*P. Savi*, s.d., FI]

Thlaspi arvense L.
T scap - W-Asiat.
Literature data: Peruzzi al. 2021 [*L. Pinzani*, 2021, PI]

NC **Turritis glabra** L.
H bienn - Circum-Artico-Alp.
Literature data: Caruel 1860

Santalaceae

Osyris alba L.
NP - Eurimedit.
Literature data: Caruel 1860; Garbari 2001
Field observations: I. Arduini, 2024

Thesium humifusum DC.
Ch suffr/H scap - Eurimedit.-Stenomedit.
Literature data: Garbari 2001, under the name *T. divaricatum* Jan ex Mert. & W.D.J. Koch

Polygonaceae

Fagopyrum esculentum Moench
T scap - Orig. Asia
Field observations: J. Franzoni, 2020

Fallopia convolvulus (L.) Á.Löve
T scap - Circumbor.
Literature data: Corti 1956, under the name *Polygonum convolvulus* L.; Garbari 2001

NC **Persicaria amphibia** (L.) Delarbre
G rhiz - Subcosmop.
Literature data: Caruel 1860, under the name *Polygonum amphibium* L.

Persicaria decipiens (R.Br.) K.L.Wilson
H scap - Subcosmop.
Herbarium data: M.L. Pedullà, 1999, PI

Persicaria hydropiper (L.) Delarbre
T scap - Circumbor.
Literature data: Caruel 1860, Tomei et al. 2004, under the name *Polygonum hydropiper* L.; Arduini & Ercoli 2012; Lombardi 2015
Field observations: I. Arduini, 2023

Persicaria lapathifolia (L.) Delarbre subsp. **lapathifolia**
T scap - Paleotemp.-Cosmop.
Literature data: Caruel 1860, Gellini et al. 1986, Garbari 2001, under the name *Polygonum lapathifolium* L.; Corti 1956, under the name *Polygonum lapathifolium* L. var. *nodosum* (Pers.) Weissn. forma *erectum* Rouy
Herbarium data: Partecipanti all'escursione Wikiplantbase #Parco di Migliarino - San Rossore - Massaciuccoli, 2018, PI

NC **Persicaria lapathifolia** (L.) Delarbre subsp. **pallida** (With.) Á.Löve
T scap - Paleotemp.
Literature data: Caruel 1860, under the name *Polygonum lapathifolium* L. var. *incanum* Koch

Persicaria maculosa Gray
T scap - Subcosmop.
Literature data: Caruel 1860, Baroni 1897-1908, Corti 1956 [*M. Savelli*, s.d., FI], Garbari 2001, under the name *Polygonum persicaria* L.; Lazzeri in Buono et al. 2022

Persicaria mitis (Schrank) Assenov
T scap - Europ.-Caucas.
Literature data: Garbari 2001, Tomei et al. 2004, under the name *Polygonum mite* Schrank

Field observations: I. Arduini, 2024

Polygonum arenastrum Boreau
T rept - Subcosmop.
Literature data: Pedullà & Garbari, 2004; Arrigoni 2019
Herbarium data: L. Pinzani, 2020, Herb. Pinzani

Polygonum aviculare L. subsp. **aviculare**
T rept - Cosmop.
Literature data: Caruel 1860; Coaro 1987; Garbari 2001

Polygonum maritimum L.
H rept - Subcosmop.
Literature data: Caruel 1860; Corti 1956; Garbari 2001; Tomei et al. 2004; Sani & Tomei 2006; Bertacchi et al. 2009; Bertacchi & Lombardi 2014b
Herbarium data: A. Sani, 2005, PI; L. Pinzani, 2019, Herb. Pinzani
Field observations: partecipanti all'escursione Wikiplantbase #Parco di Migliarino - San Rossore - Massaciuccoli, 2018

Polygonum rurivagum Jord. ex Boreau
T scap - Subcosmop.
Literature data: Lazzeri in Buono et al. 2022

Rumex acetosella L. subsp. **acetosella**
H scap - Subcosmop.
Literature data: Caruel 1860; Corti, 1951, 1956; Corti 1956 [*P. Savi*, s.d., FI]; Garbari 2001; Tomei et al. 2004; Sani & Tomei 2006; Arduini & Ercoli 2012; Bonari et al. 2019
Herbarium data: A. Sani, 2005, PI; I. Arduini, 2024

Rumex acetosella L. subsp. **pyrenaicus** (Pourr. ex Lapeyr.) Akeroyd
H scap - Subcosmop.
Literature data: Lombardi 2015, under the name *R. acetosella* L. subsp. *angiocarpus* (Murb.) Murb.; Lazzeri in Buono et al. 2022
Herbarium data: Partecipanti all'escursione Wikiplantbase #Parco di Migliarino - San Rossore - Massaciuccoli, 2018, PI

Rumex conglomeratus Murray
H scap - Eurasiat.-C-Occid.
Literature data: Caruel 1860; Corti 1956 [*Levier*, s.d., FI]; Corti 1956; Garbari 2001; Pedullà & Garbari, 2004; Tomei et al. 2004; Lazzeri 2021, 2022
Herbarium data: Partecipanti all'escursione Wikiplantbase #Parco di Migliarino - San Rossore - Massaciuccoli, 2018, PI

Rumex crispus L.
H scap - Subcosmop.
Literature data: Caruel 1860; Corti 1956; Garbari 2001; Saggese 2016; Bonari et al. 2019; Lazzeri 2021, 2022
Herbarium data: Partecipanti all'escursione Wikiplantbase #Parco di Migliarino - San Rossore - Massaciuccoli, 2018, PI

Field observations: partecipanti all'escursione Wikiplantbase #Parco di Migliarino - San Rossore - Massaciuccoli, 2018; L. Peruzzi, 2019; L. Pinzani, 2020, 2022

Rumex hydrolapathum Huds.
H scap/He - Europ.
Literature data: Coaro 1987

Rumex obtusifolius L. subsp. **obtusifolius**
H scap - Subcosmop.
Literature data: Caruel 1860; Pedullà & Garbari, 2004
Herbarium data: M.L. Pedullà, 1999, PI

Rumex patientia L. subsp. *patientia*
Literature data: Pedullà & Garbari, 2004

Rumex pulcher L. subsp. **pulcher**
H scap (T scap) - Eurimedit.
Literature data: Caruel 1860
Herbarium data: L. Pinzani, 2021, Herb. Pinzani

Rumex sanguineus L.
H scap - Europ.-Caucas.
Literature data: Corti 1956, under the name *R. sanguineus* L. f. *trigranis* K.H.Rechinger fil.; Gellini et al. 1986; Coaro 1987; Garbari 2001; Tomei et al. 2004; Lombardi 2015
Field observations: I. Arduini, 2024

Rumex ×pratensis Mert. & W.D.J.Koch
Herbarium data: M.L. Pedullà, 2000, PI

Tamaricaceae

Tamarix africana Poir.
P scap - W-Medit.
Literature data: Caruel 1860; Coaro 1987; Garbari 2001; Bertacchi et al. 2009

Tamarix gallica L.
P caesp /P scap. - W-Medit.
Literature data: Caruel 1860; Garbari 2001; Tomei et al. 2004; Bertacchi et al. 2010; Petraglia 2013; Bertacchi & Lombardi 2014a
Herbarium data: M. Savelli, 1913; Partecipanti all'escursione Wikiplantbase #Parco di Migliarino - San Rossore - Massaciuccoli, 2018, PI
Field observations: partecipanti all'escursione Wikiplantbase #Parco di Migliarino - San Rossore - Massaciuccoli, 2018

Plumbaginaceae

Limonium narbonense Mill.
H ros - Eurimedit.

Literature data: Caruel 1860, under the name Statice limonium L.; Corti 1956 [*M. Savelli*, s.d., FI; *L. Ricci*, s.d., FI]; Corti 1956, under the name *L. vulgare* Mill. var. *macrocladon* (Boiss.) C. E. Salmon; Coaro 1987, Garbari 2001, under the name *L. serotinum* (Rchb.) Pign.; Tomei et al. 2004; Sani & Tomei 2006 Bertacchi et al. 2007; Petraglia 2013; Bertacchi & Lombardi 2014b; Saggese 2016; Lazzeri 2021
Herbarium data: Partecipanti all'escursione Wikiplantbase #Parco di Migliarino - San Rossore - Massaciuccoli, 2018, PI; L. Pinzani, 2020, Herb. Pinzani
Field observations: L. Pinzani, 2021

Plumbago auriculata Lam.
Field observations: L. Pinzani, 2020

Caryophyllaceae

NC **Agrostemma githago** L. subsp. **githago**
T scap - Eurosiber.
Literature data: Caruel 1860, under the name *Lychnis githago* Scop.

Arenaria leptoclados (Rchb.) Guss. subsp. **leptoclados**
T scap - Subcosmop.
Literature data: Garbari 2001, under the name *A. serpyllifolia* L. subsp. *leptoclados* (Reichenb.) Nyman; Sani & Tomei 2006; Saggese 2016
Herbarium data: A. Sani, 2005, PI

Arenaria serpyllifolia L. subsp. **serpyllifolia**
T scap - Subcosmop.
Literature data: Corti 1956, under the name *A. serpyllifolia* L. var. *viscida* (Hall. f. in Lois.) DC., var. *scabra* Fenzl in Lebed.
Field observations: F. Roma-Marzio, 2021; L. Pinzani, 2022

Cerastium arvense L. subsp. **arvense**
H scap - Paleotemp.
Literature data: Garbari 2001; Tomei et al. 2004

Cerastium brachypetalum Desp. ex Pers. subsp. **brachypetalum**
T scap - Eurimedit. (Subpont.)
Literature data: Baroni 1897-1908, Garbari 2001, Tomei et al. 2004, under the name *Cerastium brachypetalum* Desp. ex Pers.; Corti 1956, under the name *C. brachypetalum* Desp. var. *glandulosum* Koch; Arrigoni 2019, under the name *Cerastium tauricum* Spreng.

Cerastium brachypetalum Desp. ex Pers. subsp. **roeseri** (Boiss. & Heldr.) Nyman
T scap - Stenomedit.-Turan.
Literature data: Arrigoni 2019, sub *Cerastium roeseri* Boiss. & Heldr.

Cerastium glomeratum Thuill.
T scap - Eurimedit.
Literature data: Caruel 1860; Corti 1956 [*F. Parlatore*, s.d., FI]; Corti 1956; Arduini & Ercoli 2012; Saggese 2016; Lazzeri in Buono et al. 2022
Herbarium data: L. Pinzani, A. Giacò, 2020, Herb. Pinzani

Field observations: B. Pierini, 2014; I. Arduini, 2024

Cerastium ligusticum Viv.
T scap - W-Medit.
Literature data: Caruel 1860, under the name *C. campanulatum* Viv.; Corti 1956 [*O. Beccari*, s.d., FI; *Parlatore*, s.d., FI; *A. Tassi*, s.d., FI]; Corti 1956; Coaro 1987; Garbari 2001; Sani & Tomei 2006; Arduini & Ercoli 2012
Herbarium data: D. Ciccarelli, 2012, PI; L. Pinzani, 2020, Herb. Pinzani
Field observations: L. Pinzani, 2021, 2022

Cerastium pumilum Curtis
T scap - Eurimedit.
Literature data: Garbari 2001; Bonari et al. 2019

Cerastium semidecandrum L.
T scap - Eurasiat.
Literature data: Caruel 1860; Corti, 1951, 1956; Corti 1956 [*P. Savi*, s.d., FI; *F. Parlatore*, s.d., FI]; Garbari 2001; Tomei et al. 2004; Sani & Tomei 2006; Arduini & Ercoli 2012; Lombardi 2015
Herbarium data: A. Sani, 2005, PI; D. Ciccarelli, 2012, PI; L. Pinzani, 2021, Herb. Pinzani

Cerastium siculum Guss.
T scap - Stenomedit.
Literature data: Coaro 1987; Arrigoni, 2019
Herbarium data: L. Pinzani, 2021, Herb. Pinzani

NC **Corrigiola litoralis** L. subsp. **litoralis**
T scap - Medit.-Atl.
Literature data: Baroni 1897-1908

Dianthus balbisii Ser. subsp. **balbisii**
H scap - Centromedit.-Mont.
Literature data: Caruel 1860, under the name *Dianthus carthusianorum* L.; Baroni 1897-1908, under the name *D. carthusianorum* L. var. *balbisii* Ser.; Coaro 1987, also under the name *D. carthusianorum* L. subsp. *carthusianorum*
Herbarium data: Partecipanti all'escursione Wikiplantbase #Parco di Migliarino - San Rossore - Massaciuccoli, 2018, PI
Field observations: J. Molina, 2007; B. Pierini, 2014; partecipanti all'escursione Wikiplantbase #Parco di Migliarino - San Rossore - Massaciuccoli, 2018; F. Roma-Marzio, 2019; L. Pinzani, 2019, 2020

Dianthus virgineus L.
H scap - W-Medit.
Field observations: J. Franzoni, 2021; L. Pinzani, 2022

NC **Gypsophila vaccaria** (L.) Sm.
T scap - W-Asiat. (Archeofita)
Literature data: Baroni 1897-1908, under the name *Vaccaria parviflora* Moench

Herniaria glabra L. subsp. **glabra**

T scap - Paleotemp.
Literature data: Baroni 1897-1908; Corti, 1954, 1956, under the name *H. glabra* L. forma *subciliata* Babingt.; Garbari 2001
Herbarium data: L. Pinzani, 2021, Herb. Pinzani

Herniaria hirsuta L. subsp. **hirsuta**
T scap/H caesp - Paleotemp.
Literature data: Caruel 1860; Lazzeri in Buono et al. 2022

Lychnis flos-cuculi L. subsp. **flos-cuculi**
H scap - Eurosiber.
Literature data: Caruel 1860; Corti, 1951, 1956; Gellini et al. 1986; Coaro 1987; Garbari 2001; Tomei et al. 2004; Bertacchi & Lombardi 2016
Herbarium data: Partecipanti all'escursione Wikiplantbase #Parco di Migliarino - San Rossore - Massaciuccoli, 2018, PI
Field observations: A. Mo, 2022; L. Pinzani, 2022; I. Arduini, 2023

NC **Moehringia pentandra** J.Gay
T scap - Eurimedit.
Literature data: Caruel 1860, under the name *M. trinervia* Clairv. var. *pentandra* Webb

Moehringia trinervia (L.) Clairv.
T scap - Eurasiat.
Literature data: Corti 1956; Gellini et al. 1986; Garbari 2001; Lombardi 2015; Bonari et al. 2019
Herbarium data: F. Roma-Marzio, L. Peruzzi, 2019, PI
Field observations: partecipanti all'escursione Wikiplantbase #Parco di Migliarino - San Rossore - Massaciuccoli, 2018; I. Arduini, 2023

NC **Moenchia erecta** (L.) G.Gaertn., B.Mey. & Scherb. subsp. **erecta**
T scap - Submedit.-Subatl.
Literature data: Caruel 1860, under the name *Cerastium glaucum* Gren. var. *quaternellum* Gren.

Paronychia echinulata Chater
T scap - Stenomedit.
Literature data: Lombardi 2015; Peruzzi et al. 2017b [*A. Sani*, 2015, PI]

Petrorhagia dubia (Raf.) G.López & Romo
T scap - S-Medit.
Literature data: Caruel 1860, under the name *Dianthus velutinus* Guss.; Coaro 1987, under the name *P. velutina* (Guss.) P.W.Ball & Heyw.; Garbari 2001, under the name *Vaccaria hispanica* (Mill.) Rauschert [*Ciacchi, Lorè*, 1997, PI] and under the name *Dianthus tripunctatus* Sm. [*Ciacchi, Lorè*, 1998, PI; *s. coll.*, 1998, PI]; Lombardi 2015; Peruzzi et al. 2019 [*A. Sani*, 2015, PI]

Petrorhagia prolifera (L.) P.W.Ball & Heywood
T scap - Eurimedit.
Literature data: Coaro 1987
Herbarium data: F. Roma-Marzio, M. D’Antraccoli, 2018, PI

Petrorhagia saxifraga (L.) Link subsp. **saxifraga**

H caesp - Eurimedit.
Literature data: Caruel 1860, Baroni 1897-1908, under the name *Tunica saxifraga* Scop.; Corti 1956 [*Picciuoli*, FI]; Garbari 2001
Field observations: I. Arduini, 2024

Polycarpon tetraphyllum (L.) L. subsp. **diphyllum** (Cav.) O.Bolòs & Font Quer
T scap - Stenomedit.
Literature data: Orlandi & Arduini 2010
Herbarium data: L. Pinzani, 2019, Herb. Pinzani

Polycarpon tetraphyllum (L.) L. subsp. **tetraphyllum**
T scap - Eurimedit.
Literature data: Caruel 1860; Corti, 1951, 1956; Garbari 2001
Herbarium data: Partecipanti all'escursione Wikiplantbase #Parco di Migliarino - San Rossore - Massaciuccoli, 2018, PI
Field observations: L. Peruzzi, 2015; partecipanti all'escursione Wikiplantbase #Parco di Migliarino - San Rossore - Massaciuccoli, 2018; L. Pinzani, 2022

Sabulina mediterranea (Ledeb. ex Link) Rchb. subsp. **mediterranea**
T scap - NW-Medit.
Literature data: Corti 1956, Garbari 2001, Arrigoni, 2019, under the name *Minuartia mediterranea* (Link) K.Maly
Herbarium data: L. Pinzani, 2021, Herb. Pinzani
Field observations: L. Pinzani, 2021

Sabulina tenuifolia (L.) Rchb. subsp. **tenuifolia**
T scap - Paleotemp.
Literature data: Caruel 1860, under the name *Alsine tenuifolia* Crantz
Herbarium data: L. Pinzani, 2021, Herb. Pinzani

Sagina apetala Ard. subsp. **apetala**
T scap - Eurimedit.
Literature data: Corti 1956, under the name *S. apetala* Arduino subsp. *ciliata* (Fries) J. Ball, subsp. *apetala* var. *barbata* Fenzl; Garbari 2001

Sagina maritima Don
T scap - Medit.-Atl.
Literature data: Garbari 2001

Sagina micropetala Rauschert
T scap - Eurimedit.
Field observations: J. Franzoni, 2020

Sagina procumbens L. subsp. **procumbens**
H caesp - Subcosmop.
Literature data: Bertacchi et al. 2010; Orlandi & Arduini 2010; Bertacchi & Lombardi 2014a
Field observations: F. Roma-Marzio, 2019

Saponaria officinalis L.

H scap - Eurosiber.
Literature data: Caruel 1860; Corti 1956; Garbari 2001
Field observations: B. Pierini, 2014

NC **Scleranthus annuus** L.
T scap (H bienn) - Paleotemp.
Literature data: Caruel 1860

Silene baccifera (L.) Durande
H scap - Eurosiber.
Literature data: Pedullà & Garbari, 2004, under the name *Cucubalus baccifer* L.

Silene canescens Ten.
T scap - Stenomedit.
Literature data: Caruel 1860, Baroni 1897-1908, under the name *S. sericea* All.; Sani & Tomei 2006; Bertacchi et al. 2009, Bertacchi et al. 2010, Bertacchi & Lombardi 2014a, Bertacchi & Lombardi 2014b, under the name *S. colorata* Poir.; Arrigoni, 2019
Herbarium data: L. Pinzani, 2020, Herb. Pinzani
Field observations: B. Pierini, 2004; L. Pinzani, 2021, 2022

Silene conica L.
T scap - Paleotemp.
Literature data: Baroni 1897-1908; Coaro 1987, under the name *S. conica* L. subsp. *subconica* (Friv.) Gavioli**;** Saggese 2016; Lazzeri in Buono et al. 2022
Herbarium data: L. Pinzani, 2021, Herb. Pinzani; L. Pinzani, A. Giacò, J. Franzoni, 2021, Herb. Pinzani
Field observations: L. Pinzani, 2021
That from Coaro (1987) is the only record for Tuscany of *Silene subconica* Friv., considered of doubtful occurrence in the region (Bartolucci et al., 2024). There is no herbarium documentation for this record, but other specimens and observations from the same area allow to actually refer this record to *Silene conica* L. Therefore, *S. subconica* is excluded from the flora of the study area and from the regional flora of Tuscany.

NC **Silene cretica** L.
T scap - Stenomedit.-Orient.
Literature data: Caruel 1860

Silene gallica L.
T scap - Eurimedit.
Literature data: Caruel 1860; Corti 1956; Coaro 1987; Garbari 2001; Arduini & Ercoli 2012; Lombardi 2015; Bonari et al. 2019
Herbarium data: Partecipanti all'escursione Wikiplantbase #Parco di Migliarino - San Rossore - Massaciuccoli, 2018, PI; L. Pinzani, 2021, Herb. Pinzani
Field observations: B. Pierini, 2005; partecipanti all'escursione Wikiplantbase #Parco di Migliarino - San Rossore - Massaciuccoli, 2018; L. Pinzani, 2021, 2022

Silene italica (L.) Pers. subsp. **italica**
H ros - Eurimedit.
Literature data: Caruel 1860; Corti 1956 [*P. Savi*, s.d., FI]; Garbari 2001

Silene latifolia Poir.
H bienn - Paleotemp.
Literature data: Caruel 1860, under the name *Lychnis alba* Mill.; Corti 1956, under the name *Melandrium album* (Mill.) Garcke; Coaro 1987, under the names *S. alba* (Miller) Krause and *S. dioica* (L.) Clairv.; Saggese 2016; Lazzeri 2021
Field observations: B. Pierini, 2014; partecipanti all'escursione Wikiplantbase #Parco di Migliarino - San Rossore - Massaciuccoli, 2018; L. Peruzzi, 2019; L. Pinzani, 2020, 2021, 2022; J. Franzoni, A. Giacò, 2023

Silene niceensis All.
T scap - Stenomedit.
Literature data: Caruel 1860; Garbari 2001; Bertacchi et al. 2010; Bertacchi & Lombardi 2014a
Herbarium data: M. Mannocci, 2013, Herb. Museo Livorno; L. Pinzani, 2021, Herb. Pinzani

Silene nocturna L. subsp. **nocturna**
T scap - S-Medit.-Macarones.
Literature data: Caruel 1860
Herbarium data: L. Pinzani, 2021, Herb. Pinzani
Field observations: L. Peruzzi, 2018; B. Pierini, 2018; L. Pinzani, 2022

Silene otites (L.) Wibel subsp. **otites**
H ros - Eurasiat. (steppica)
Literature data: Caruel 1860; Corti 1956 [*M. Savelli*, s.d., FI]; Garbari 2001; Bertacchi et al. 2009
Field observations: R. Righini, 2021

Silene vulgaris (Moench) Garcke subsp. **vulgaris**
H scap - Paleotemp.-Subcosmop.
Literature data: Caruel 1860, under the name *S. inflata* Smith

Spergula arvensis L.
T scap - Subcosmop.
Literature data: Caruel 1860
Herbarium data: Partecipanti all'escursione Wikiplantbase #Parco di Migliarino - San Rossore - Massaciuccoli, 2018, PI
Field observations: L. Pinzani, 2021, 2022

NC **Spergula pentandra** L.
T scap - Subatl.-Submedit.
Literature data: Baroni 1897-1908

Spergularia marina (L.) Besser
T scap - Subcosmop.
Literature data: Caruel 1860, under the name *Lepigonum marinum* Wahlb.; Saggese 2016

Spergularia media (L.) C.Presl
Ch suffr - Subcosmop.
Literature data: Coaro 1987; Garbari 2001; Sani & Tomei 2006; Petraglia 2013; Saggese 2016; Arrigoni, 2019

Herbarium data: A. Sani, 2005, PI
Field observations: L. Pinzani, 2018

Spergularia rubra (L.) J.Presl & C.Presl
Ch suffr - Subcosmop.
Literature data: Caruel 1860, under the name *Lepigonum rubrum* Wahlb; Corti 1956 [*P. Fantozzi*, FI]; Garbari 2001.
Field observations: B. Pierini, 2014, 2017; partecipanti all'escursione Wikiplantbase #Parco di Migliarino - San Rossore - Massaciuccoli, 2018

Stellaria aquatica (L.) Scop.
H scap - Eurosiber.
Literature data: Caruel 1860, Baroni 1897-1908, under the name *Malachium aquaticum* Fries
Field observations: B. Pierini, 2015

Stellaria media (L.) Vill.
T rept - Cosmop.
Literature data: Caruel 1860; Corti 1956; Gellini et al. 1986; Garbari 2001; Saggese 2016; Bonari et al. 2019
Field observations: B. Pierini, 2014; partecipanti all'escursione Wikiplantbase #Parco di Migliarino - San Rossore - Massaciuccoli, 2018; L. Pinzani, 2021, 2022; I. Arduini, 2023

Stellaria neglecta Weihe subsp. **neglecta**
T scap - Paleotemp.
Field observations: B. Pierini, 2014; L. Pinzani, 2022

Amaranthaceae

Alternanthera philoxeroides (Mart.) Griseb.
I rad - Orig. S-America
Literature data: Roma-Marzio & D'Antraccoli in Peruzzi et al. 2016 [*F. Roma-Marzio, M. D'Antraccoli*, 2015, PI]
Herbarium data: L. Pinzani, 2020, Herb. Pinzani
Field observations: F. Roma-Marzio, 2018

Amaranthus albus L.
T scap - Orig. N-America
Literature data: Baroni 1897-1908; Iamonico 2015 [*Santarelli*, 1888, BI; *S. Sommier*, 1896, RO]; Lazzeri in Buono et al. 2022

Amaranthus blitum L. subsp. **blitum**
T scap - Cosmop.
Literature data: Caruel 1860; Pedullà & Garbari, 2004, under the name *A. lividus* L.
Herbarium data: M.L. Pedullà, 1999, PI; L. Pinzani, 2020, Herb. Pinzani

NC *Amaranthus crassipes* Schltdl. subsp. *crassipes*
Literature data: Iamonico 2015 [*G. Savi*, 1839, FI]
Only temporarily cultivated in the Botanic Garden of the University of Pisa.

NC *Amaranthus hybridus* L. subsp. *cruentus* (L.) Thell.
Literature data: Baroni 1897-1908, under the name *A. patulus* Bert.

Amaranthus deflexus L.
T scap/H scap - Orig. S-America
Literature data: Iamonico 2015 [s. *coll.*, 1847, PAL]; Arduini & Ercoli 2012
Field observations: L. Pinzani, 2020, 2021

NC **Amaranthus graecizans** L. subsp. **sylvestris** (Vill.) Brenan
T scap - Paleotemp.
Literature data: Baroni 1897-1908, under the name *A. sylvestris* Vill.

Amaranthus hybridus L. subsp. hybridus
T scap - Orig. America
Literature data: Iamonico 2015 [*T. Caruel*, 1862, RO]
Herbarium data: L. Pinzani, 2020, Herb. Pinzani

NC *Amaranthus polygonoides* L.
Literature data: Iamonico 2015 [s. *coll.*, 1839, FI]
Only temporarily cultivated in the Botanic Garden of the University of Pisa

Amaranthus retroflexus L.
T scap - Orig. N-America
Literature data: Baroni 1897-1908; Iamonico 2015 [*Cicioni*, 1909, PERU]; Lazzeri in Buono et al. 2022

Amaranthus tuberculatus (Moq.) J.D.Sauer
Literature data: Lazzeri et al. in Alessandrini et al. 2013

Amaranthus viridis L.
Herbarium data: L. Pinzani, 2020, Herb. Pinzani

Arthrocaulon macrostachyum (Moric.) Piirainen & G.Kadereit
Ch succ/P succ - Medit.-Macarones.
Literature data: Coaro 1987 [*E. Coaro*, 1984, PI; *G. Pistolesi, E. Coaro*, 1986, PI], under the name *Arthrocnemum glaucum* (Delile) Ung.-Sternb.; Bertacchi et al. 2007; Petraglia 2013
Field observations: partecipanti all'escursione Wikiplantbase #Parco di Migliarino - San Rossore - Massaciuccoli, 2018; L. Pinzani, 2020

NC **Atriplex halimus** L.
P caesp - Stenomedit.
Herbarium data: A. Fiori, 1903, FI

Atriplex littoralis L.
T scap - Eurasiat.
Literature data: Garbari 2001

NC **Atriplex patula** L. subsp. **patula**
T scap - Circumbor.

Literature data: Caruel 1860

Atriplex prostrata Boucher ex DC.
T scap - Circumbor.
Literature data: Baroni 1897-1908, under the name *A. hastata* L.; Coaro 1987, Sani & Tomei 2006, Bertacchi et al. 2009, under the name *A. latifolia* Wahlenb.; Saggese 2016; Lazzeri in Buono et al. 2022
Field observations: partecipanti all'escursione Wikiplantbase #Parco di Migliarino - San Rossore - Massaciuccoli, 2018

Atriplex tatarica L.
T scap - Eurimedit.-Asiat.
Herbarium data: D. Marchetti, 1981, SIENA

Beta vulgaris L. subsp. **maritima** (L.) Arcang.
H scap - Eurimedit.
Literature data: Saggese 2016
Herbarium data: F. Picco, 1995, Herb. Picco

Beta vulgaris L. subsp. *vulgaris*
Literature data: Caruel 1860; Coaro 1987; Pedullà & Garbari, 2004
Herbarium data: L. Pinzani, 2019, Herb. Pinzani
Field observations: B. Pierini, 2014, 2017, 2021; L. Pinzani, 2021, 2022

NC **Chenopodiastrum hybridum** (L.) S.Fuentes, Uotila & Borsch
T scap - Circumbor.
Literature data: Caruel 1860, under the name *Chenopodium hybridum* L.

Chenopodiastrum murale (L.) S.Fuentes, Uotila & Borsch
T scap - Subcosmop.
Herbarium data: L. Pinzani, 2020, Herb. Pinzani

Chenopodium album L. subsp. **album**
T scap - Subcosmop.
Literature data: Caruel 1860; Orlandi & Arduini 2010; ; Arduini & Ercoli 2012; Lazzeri in Buono et al. 2022
Field observations: partecipanti all'escursione Wikiplantbase #Parco di Migliarino - San Rossore - Massaciuccoli, 2018; L. Pinzani, 2021

Chenopodium opulifolium Schrad. ex W.D.J.Koch & Ziz
T scap - Paleotemp.
Literature data: Caruel 1860
Field observations: partecipanti all'escursione Wikiplantbase #Parco di Migliarino - San Rossore - Massaciuccoli, 2018

NC **Chenopodium vulvaria** L.
T scap - Eurimedit.
Literature data: Caruel 1860, under the name *C. olidum* Curt.

Dysphania ambrosioides (L.) Mosyakin & Clemants
T scap - Orig. America
Literature data: Baroni 1897-1908, Pedullà & Garbari, 2004, under the name *Chenopodium ambrosioides* L.
Herbarium data: L. Pinzani, 2020, Herb. Pinzani; I. Arduini, 2023, PIAGR
Field observations: L. Pinzani, 2020

NC *Dysphania atriplicifolia* (Spreng.) G.Kadereit, Sukhor. & Uotila
Literature data: Caruel 1860, Montelucci 1962, under the name *Cycloloma platyphyllum* Moq.

Dysphania botrys (L.) Mosyakin & Clemants
T scap - Eurasiat.-Subcosmop.
Literature data: Caruel 1860, under the name *Chenopodium botrys* L.; Orlandi & Arduini 2010; Arduini & Ercoli 2012

NC *Dysphania multifida* (L.) Mosyakin & Clemants
Literature data: Montelucci 1962, under the name *Chenopodium multifidum* L.

Halimione portulacoides (L.) Aellen
Ch frut/P rept - Circumbor.
Literature data: Coaro 1987 [*E. Coaro*, 1984, PI; *G. Pistolesi*, *E. Coaro*, 1984, PI]; Bertacchi et al. 2007; Petraglia 2013; Saggese 2016
Herbarium data: Partecipanti all'escursione Wikiplantbase #Parco di Migliarino - San Rossore - Massaciuccoli, 2018, PI; L. Pinzani, 2020, Herb. Pinzani

NC **Lipandra polysperma** (L.) S.Fuentes, Uotila & Borsch
T scap - Paleotemp.-Circumbor.
Literature data: Caruel 1860, under the name *Chenopodium polyspermum* L.

NC **Oxybasis chenopodioides** (L.) S.Fuentes, Uotila & Borsch
T scap - Subcosmop.
Literature data: Iamonico et al. 2013 [*Beccari*, 1861, FI; *Beccari*, 1862, FI]

NC **Oxybasis rubra** (L.) S.Fuentes, Uotila & Borsch
T scap - Circumbor.
Literature data: Caruel 1860, Baroni 1897-1908, under the name *Chenopodium rubrum* L.

NC **Oxybasis urbica** (L.) S.Fuentes, Uotila & Borsch
T scap - Eurosiber.-Subcosmop.
Literature data: Caruel 1860, under the name *Chenopodium urbicum* L.

Salicornia fruticosa (L.) L.
Ch succ - Eurimedit.
Literature data: Baroni 1897-1908; Tomei et al. 2004, under the name *Arthrocnemum fruticosum* (L.) Moq; Petraglia 2013, under the name *Sarcocornia fruticosa*

Salicornia perennans Willd. subsp. **perennans**
T scap - W-Europ.

Literature data: Caruel 1860, under the name *S. herbacea* L.; Coaro 1987, Garbari 2001, under the name *S. europaea* L.; Tomei et al. 2004, Sani & Tomei 2006, Bertacchi et al. 2007, Petraglia 2013, Bertacchi & Lombardi 2014b, Saggese 2016, under the name *S. patula* Duval-Jouve
Herbarium data: L. Pinzani, 2020, Herb. Pinzani

Salicornia perennis Mill. subsp. **perennis**
Ch succ - Subcosmop.
Literature data: Bertacchi et al. 2007, Saggese 2016, Arrigoni, 2019, under the name *Sarcocornia perennis* (Miller) A.J.Scott

Salsola tragus L.
T scap - Paleotemp.
Literature data: Caruel 1860, Garbari 2001, Tomei et al. 2004, Sani & Tomei 2006, Bertacchi et al. 2007, Bertacchi et al. 2009, Bertacchi & Lombardi 2014, under the name *S. kali* L.; Corti 1956, under the name *S. kali* L. var. *brevimarginata* Koch
Herbarium data: A. Sani, 2005, PI; D. Ciccarelli & M. Sammartino, 2009, PI

Soda inermis Fourr.
T scap - Paleotemp.
Literature data: Coaro 1987, Garbari 2001, Petraglia 2013, under the name *Salsola soda* L.
Herbarium data: L. Pinzani, 2020, Herb. Pinzani
Field observations: partecipanti all'escursione Wikiplantbase #Parco di Migliarino - San Rossore - Massaciuccoli, 2018

Suaeda spicata (Willd.) Moq.
T scap - Cosmop.
Literature data: Sani & Tomei 2006, Bertacchi et al. 2007, Petraglia 2013, Saggese 2016, under the name *Suaeda maritima* (L.) Dumort.
Herbarium data: L. Pinzani, 2020, Herb. Pinzani
Field observations: partecipanti all'escursione Wikiplantbase #Parco di Migliarino - San Rossore - Massaciuccoli, 2018

Aizoaceae

Carpobrotus acinaciformis (L.) L.Bolus
Ch suffr - Orig. S-Africa
Literature data: Bertacchi et al. 2010; Bertacchi & Lombardi 2014a

NC *Tetragonia tetragonoides* (Pall.) Kuntze
Literature data: Lazzaro et al. 2013 [*M. Savelli*, 1925, FI]

Phytolaccaceae

Phytolacca americana L.
G rhiz - Orig. N-America
Literature data: Corti 1956; Garbari 2001; Arduini & Ercoli 2012; Lombardi 2015
Field observations: partecipanti all'escursione Wikiplantbase #Parco di Migliarino - San Rossore - Massaciuccoli, 2018; I. Arduini, 2023

Nyctaginaceae

Bougainvillea glabra Choisy
Field observations: observed on iNaturalist in 2024
Only cultivated

Mirabilis jalapa L.
Field observations: observed on iNaturalist in 2024
Only cultivated

Montiaceae

NC **Montia arvensis** Wallr.
T scap/I rad - Medit.-Subatl.
Literature data: Caruel 1860, under the name *M. fontana* L.

Basellaceae

Anredera cordifolia (Ten.) Steenis
G rhiz - Orig. S-America
Literature data: Pedullà & Garbari, 2004

Portulacaceae

Portulaca oleracea L.
T scap - Subcosmop.
Literature data: Caruel 1860; Orlandi & Arduini 2010; Danin 2011 [*A. Danin*, 2007, HUJ]; Arduini & Ercoli 2012
Field observations: H. Öhm, 2024

Cactaceae

Opuntia ficus-indica (L.) Mill.
P succ - Neotrop.
Field observations: observed on iNaturalist in 2019

Cornaceae

Cornus mas L.
P caesp/P scap - SE-Europ.-Pont.
Literature data: Coaro 1987 [*G. Pistolesi, E. Coaro*, 1984, PI]; Bertacchi et al. 2010

Cornus sanguinea L. subsp. **hungarica** (Kárpáti) Soó
P caesp - Eurasiat.-Temp.
Literature data: Caruel 1860, Corti 1956, 1970, Gellini et al. 1986, Coaro 1987 [*G. Pistolesi, E. Coaro*, 1984, PI], Garbari 2001 [*B. Ciacchi, G. Lorè*, 1996, 1997, PI], Bertacchi & Lombardi 2016, under the name *C. sanguinea* L.; Lazzeri 2021
Herbarium data: R. Poli, 1989, PI

Field observations: partecipanti all'escursione Wikiplantbase #Parco di Migliarino - San Rossore - Massaciuccoli, 2018; L. Pinzani, 2020, 2022

Ebenaceae

Diospyros lotus L.
Literature data: Peruzzi et al. 2019 [*F. Roma-Marzio, M. D'Antraccoli*, 2018, FI, PI]

Primulaceae

Cyclamen hederifolium Aiton subsp. **hederifolium**
G bulb - N-Medit.
Literature data: Caruel 1860, under the name *C. neapolitanum* Ten.; Corti 1956 [*M. Savelli*, FI]; Corti 1956, under the name *C. neapolitanum* Ten. var. *praecox* Ten.; Gellini et al. 1986; Garbari 2001; Lombardi 2015; Bertacchi & Lombardi 2016
Field observations: I. Arduini, 2024

Cyclamen persicum Mill.
Field observations: observed on iNaturalist in 2023

Cyclamen repandum Sm. subsp. **repandum**
G bulb - N-Medit.
Literature data: Caruel 1860; Corti 1956 [*P. Savi*, s.d., FI; *P. Fantozzi*, s.d., FI]; Corti 1956, under the name *C. repandum* Sibth. et Sm. var. *repandum*; Gellini et al. 1986; Coaro 1987; Tomei et al. 2004; Bertacchi & Lombardi 2016; Bonari et al. 2019
Field observations: A. Giacò, J. Franzoni, L. Pinzani, 2021; L. Pinzani, 2021; I. Arduini, 2024

Hottonia palustris L.
I rad - Eurosiber.
Literature data: Caruel 1860; Corti 1956 [*P. Savi*, s.d., FI]; Gellini et al. 1986; Coaro 1987
Assessed as EN in the Red List of the Italian Vascular Flora (Rossi et al. 2013)

Lysimachia arvensis (L.) U.Manns & Anderb.
T rept - Eurimedit.-Subcosmop.
Literature data: Caruel 1860, Coaro 1987, Garbari 2001, Tomei et al. 2004, Sani & Tomei 2006, Bonari et al. 2019, under the name *Anagallis arvensis* L.; Corti 1956, 1970, under the name *A. arvensis* L. subsp. *phoenicea* (Scop.) Vollmann; Arduini & Ercoli 2012; Lazzeri in Buono et al. 2022
Herbarium data: A. Sani, 2005, PI; Partecipanti all'escursione Wikiplantbase #Parco di Migliarino - San Rossore - Massaciuccoli, 2018, PI; L. Pinzani, 2021, Herb. Pinzani
Field observations: partecipanti all'escursione Wikiplantbase #Parco di Migliarino - San Rossore - Massaciuccoli, 2018; M. D'Antraccoli, 2019; L. Pinzani, 2021, 2022; H. Öhm, 2024

Lysimachia linum-stellatum L.
T scap - Stenomedit.
Literature data: Corti 1956, Orlandi & Arduini 2010, Arduini & Ercoli 2012, Bonari et al. 2019, under the name *Asterolinon linum-stellatum* (L.) Duby in DC.
Field observations: L. Pinzani, 2022

NC **Lysimachia minima** (L.) U.Manns & Anderb.
T scap - Eurasiat.-Temp.
Literature data: Caruel 1860, under the name *Centunculus minimus* L.

Lysimachia nummularia L.
H scap - Circumbor.-Europ.-Caucas.
Literature data: Caruel 1860; Corti 1956; Coaro 1987; Garbari 2001; Pedullà & Garbari, 2004; Tomei et al. 2004
Field observations: L. Pinzani, 2022

NC **Lysimachia punctata** L.
H scap - SE-Europ.-Pont.
Literature data: Caruel 1860

NC **Lysimachia tenella** L.
H caesp - Atlant.
Literature data: Caruel 1860, Baroni 1897-1908, Corti 1956a [*C. Rossetti*, FI; *Amidei*, FI; *Picciuoli*, FI]; Corti 1956a, 1970, Garbari 2001, under the name *Anagallis tenella* (L.) L.

Lysimachia vulgaris L.
H scap - Eurasiat.
Literature data: Caruel 1860; Baroni 1897-1908; Corti 1956 [*Sommier*, s.d., FI]; Corti 1956; Gellini et al. 1986; Tomei et al. 2004
Field observations: B. Pierini, 2014

Samolus valerandi L.
H scap - Subcosmop.
Literature data: Caruel 1860; Corti 1956, 1970; Gellini et al. 1986; Coaro 1987; Garbari 2001; Tomei et al. 2004; Sani & Tomei 2006; Petraglia 2013; Bertacchi & Lombardi 2014b; Saggese 2016; Lazzeri in Buono et al. 2022
Herbarium data: G. Bedini, 2016, PI; Partecipanti all'escursione Wikiplantbase #Parco di Migliarino - San Rossore - Massaciuccoli, 2018, PI
Assessed as LC in the Red List of the Italian Vascular Flora (Rossi et al. 2020)

Ericaceae

Arbutus unedo L.
P caesp/scap - Stenomedit.
Literature data: Corti 1956, under the name *A. unedo* L. var. *serratifolia* Rouy; Garbari 2001; Bertacchi et al. 2010; Bertacchi & Lombardi 2014a
Herbarium data: A. Chiarugi, R. Corti, 1951, FI
Field observations: L. Pinzani, 2020

Erica arborea L.
P caesp - Stenomedit.
Literature data: Corti 1956; Coaro 1987; Garbari 2001 [*B. Ciacchi, G. Lorè*, 1996, PI]; Tomei et al. 2004; Bonari et al. 2019
Herbarium data: Accad. dei Georg., 1854, FI; A. Chiarugi, 1933, FI; R. Poli, 1989, PI
Field observations: L. Peruzzi, 2017

Erica scoparia L. subsp. **scoparia**
P caesp - W-Medit.
Literature data: Caruel 1860; Corti 1956; Garbari 2001 [*B. Ciacchi*, *G. Lorè*, 1997, 1998, PI]; Tomei et al. 2004; Arduini & Ercoli 2012
Herbarium data: A. Chiarugi, R. Corti, 1951, FI; A. Chiarugi, 1953, FI

NC **Monotropa hypopitys** L.
G par - Circumbor.
Literature data: Baroni 1897-1908, under the name *Hypopitys multifiora* Scop.; Corti 1956, under the name *M. hypopitys* L. var. *glabra* Roth

NC **Orthilia secunda** (L.) House
Ch rept - Circumbor.
Herbarium data: H. van Heurck, 1868, P

Rubiaceae

NC **Asperula arvensis** L.
T scap - Eurimedit.
Literature data: Caruel 1860

Cruciata glabra (L.) C.Bauhin ex Opiz
H scap - Eurasiat.
Literature data: Corti 1956, under the name *Galium vernum* L. var. *typicum* Rouy, var. *hirticaule* Beck; Gellini et al. 1986; Garbari 2001

Cruciata laevipes Opiz
H scap - Eurasiat.
Literature data: Caruel 1860, under the name *Galium cruciata* Scop.; Corti 1956 [*P. Savi*, s.d., FI; *F. Parlatore*, s.d., FI]; Coaro 1987

NC **Cynanchica pyrenaica (**L.) P.Caputo & Del Guacchio subsp. **cynanchica** (L.) P.Caputo & Del Guacchio
H scap - Eurimedit.
Literature data: Corti 1956 [*A. Biondi*, s.d., FI]; Corti 1956, under the name *Asperula cyn*anchica L. subsp. *cynanchica* Beck var. *vulgaris* Rchb.

Galium album Mill. subsp. **album**
H scap - Eurimedit.
Field observations: partecipanti all'escursione Wikiplantbase #Parco di Migliarino - San Rossore - Massaciuccoli, 2018

Galium aparine L.
T scap - Eurasiat.
Literature data: Caruel 1860; Corti 1956; Garbari 2001; Tomei et al. 2004; Bonari et al. 2019; Lazzeri 2021, 2022
Herbarium data: M.L. Pedullà, 1999, PI

Field observations: partecipanti all'escursione Wikiplantbase #Parco di Migliarino - San Rossore - Massaciuccoli, 2018; L. Pinzani, 2021, 2022

Galium debile Desv.
H scap - Eurimedit.
Literature data: Baroni 1897-1908, under the name *G. palustre* L. var. *constrictum* Chaub.; Corti 1956 [*Picciuoli*, s.d., FI]; Gellini et al. 1986; Saggese 2016

NC **Galium lucidum** All. subsp. **lucidum**
H scap - Eurimedit.
Literature data: Caruel 1860, under the name *G. erectum* Huds.; Corti 1956 [*P. Savi*, s.d., FI]; Corti 1956

Galium mollugo L.
H scap - Eurimedit.
Literature data: Caruel 1860; Pedullà & Garbari 2002 [*M.L. Pedullà*, 1999, PI]; Pedullà & Garbari 2004; Saggese 2016; Lazzeri 2021, 2022
Herbarium data: Partecipanti all'escursione Wikiplantbase #Parco di Migliarino - San Rossore - Massaciuccoli, 2018, PI
Field observations: L. Pinzani, 2020, 2021

Galium murale (L.) All.
T scap - Stenomedit.
Literature data: Caruel 1860, under the name *Callipeltis muralis* Mor.; Corti 1956, under the name *G. murale* All. var. *typicum* Fiori; Orlandi & Arduini 2010; Arduini & Ercoli 2012
Herbarium data: L. Pinzani, 2020, Herb. Pinzani
Field observations: L. Pinzani, 2022

Galium palustre L. subsp. **elongatum** (C.Presl) Arcang.
H scap - Eurimedit.
Literature data: Corti 1956, under the name *G. palustre* L. var. *lanceolatum* Uectr.; Coaro 1987, Pedullà & Garbari, 2004, under the name *G. elongatum* Presl; Arrigoni 2019; Lazzeri in Buono et al. 2022

Galium palustre L. subsp. **palustre**
H scap - Europ.-W-Asiat.
Literature data: Caruel 1860; Corti 1956 [*A. Biondi*, s.d., FI]; Corti 1956, under the name *G. palustre* L. var. *vulgare* Vectr.; Gellini et al. 1986; Coaro 1987; Garbari 2001; Tomei et al. 2004; Arduini & Ercoli 2012; Petraglia 2013; Lombardi 2015
Herbarium data: Partecipanti all'escursione Wikiplantbase #Parco di Migliarino - San Rossore - Massaciuccoli, 2018, PI
Field observations: I. Arduini, 2024

Galium parisiense L.
T scap - Eurimedit.
Literature data: Lombardi 2015; Peruzzi et al. 2017b [*A. Sani*, 2015, PI]

Galium rotundifolium L. subsp. **rotundifolium**
H scap - Orof. W-Eurasiat.

Literature data: Tomei et al. 2004
Field observations: I. Arduini, 2024

NC **Galium tricornutum** Dandy
T scap - Eurimedit.
Literature data: Caruel 1860, under the name *G. tricorne* With.

Galium verum L. subsp. **verum**
H scap - Eurasiat.
Literature data: Caruel 1860; Coaro 1987; Garbari 2001; Pedullà & Garbari, 2004; Tomei et al. 2004; Lazzeri in Buono et al. 2022
Field observations: partecipanti all'escursione Wikiplantbase #Parco di Migliarino - San Rossore - Massaciuccoli, 2018; L. Pinzani, 2020, 2022

Rubia peregrina L.
P lian - Stenomedit.-Macarones.
Literature data: Caruel 1860; Corti 1956, under the name *R. peregrina* L. var. *lucida* (L.) Rouy; Coaro 1987 [*G. Pistolesi, E. Coaro*, 1976, PI]; Garbari 2001; Tomei et al. 2004; Bertacchi et al. 2009; Bertacchi et al. 2010; Arduini & Ercoli 2012; Bertacchi & Lombardi 2014a; Bertacchi & Lombardi 2016; Lazzeri 2021
Herbarium data:; C. Del Prete, 1979, PI; R. Poli, 1989, PI
Field observations: L. Peruzzi, 2017; partecipanti all'escursione Wikiplantbase #Parco di Migliarino - San Rossore - Massaciuccoli, 2018; L. Pinzani, 2020, 2021, 2022

Sherardia arvensis L.
T scap - Eurimedit.
Literature data: Caruel 1860; Corti 1956 [*F. Parlatore*, s.d., FI]; Corti 1956, under the name *S. arvensis* L. var. *typica* Bèguinot in Fiori e Paoletti; Coaro 1987; Garbari 2001; Pedullà & Garbari, 2004; Tomei et al. 2004; Sani & Tomei 2006; Arduini & Ercoli 2012; Lombardi 2015; Bonari et al. 2019
Field observations: B. Pierini, 2014; partecipanti all'escursione Wikiplantbase #Parco di Migliarino - San Rossore - Massaciuccoli, 2018; A. Mo, 2022; L. Pinzani, 2021, 2022; I. Arduini, 2024

Theligonum cynocrambe L.
T scap - Stenomedit.
Literature data: Caruel 1860
Field observations: L. Pinzani, 2021

Gentianaceae

Blackstonia acuminata (W.D.J.Koch & Ziz) Domin subsp. **acuminata**
T scap - Medit.
Literature data: Saggese 2016; Lazzeri in Buono et al. 2022

Blackstonia perfoliata (L.) Huds. subsp. **perfoliata**
T scap - Eurimedit.
Literature data: Caruel 1860, under the name *Chlora perfoliata* L.; Corti 1956; Coaro 1987; Garbari 2001; Bertacchi et al. 2009; Petraglia 2013; Bertacchi & Lombardi 2016

Herbarium data: Partecipanti all'escursione Wikiplantbase #Parco di Migliarino - San Rossore - Massaciuccoli, 2018, PI
Field observations: L. Pinzani, 2022

Centaurium erythraea Rafn subsp. **erythraea**
H bienn/T scap - Paleotemp.
Literature data: Caruel 1860, under the name *Erythraea centaurium* Pers.; Corti 1956, under the name *C. umbellatum* Gilib. subsp. *typicum* (Wittrock in herb.) Romm.; Coaro 1987; Garbari 2001; Tomei et al. 2004; Sani & Tomei 2006; Arduini & Ercoli 2012; Lazzeri in Buono et al. 2022
Herbarium data: Partecipanti all'escursione Wikiplantbase #Parco di Migliarino - San Rossore - Massaciuccoli, 2018, PI
Field observations: R. Righini, 2021

Centaurium maritimum (L.) Fritsch
T scap - Stenomedit.
Literature data: Caruel 1860, under the name *Erythraea maritima* Pers.; Corti 1956 [*G. Savi*, FI]
Herbarium data: L. Pinzani, 2020, Herb. Pinzani

Centaurium pulchellum (Sw.) Druce subsp. **pulchellum**
T scap - Paleotemp.
Literature data: Caruel 1860, under the name *Erythraea ramosissima* Pers.; Corti 1956, under the name *C. pulchellum* (Sw.) Druce subforma *albiflorum* (Boiss.) Hayek.; Garbari 2001; Tomei et al. 2004; Petraglia 2013; Lazzeri in Buono et al. 2022
Herbarium data: F. Roma-Marzio, M. D'Antraccoli, 2017, PI
Field observations: B. Pierini, 2017

Centaurium tenuiflorum (Hoffmanns. & Link) Fritsch subsp. **tenuiflorum**
T scap - Paleotemp.
Literature data: Garbari 2001; Tomei et al. 2004; Lombardi 2015; Saggese 2016; Lazzeri in Buono et al. 2022

NC **Cicendia filiformis** (L.) Delarbre
T scap - Subatl.-W-Europ.
Literature data: Caruel 1860

NC **Exaculum pusillum** (Lam.) Caruel
T scap - W-Medit.
Literature data: Baroni 1897-1908, under the name *Cicendia pusilla* Gris.

Schenkia spicata (L.) G.Mans.
T scap - Eurimedit.
Literature data: Caruel 1860, under the name *Erythraea spicata* Pers.; Coaro 1987, Garbari 2001, Tomei et al. 2004, Sani & Tomei 2006, Petraglia 2013, under the name *Centaurium spicatum* (L.) Fritsch

Apocynaceae

D *Cynanchum acutum* L. subsp. *acutum*
Literature data: Caruel 1860

Nerium oleander L. subsp. oleander
P caesp/P scap - Orig. S-Medit.
Literature data: Bertacchi et al. 2009

Periploca graeca L.
P lian - NE-Medit.
Literature data: Caruel 1860; Baroni 1897-1908; Corti 1956 [*S. Sommier*, s.d., FI; *Narducci*, s.d., FI; *D. Bergeest*, s.d., FI; *U. Martelli*, s.d., FI]; Corti 1956, 1970; Gellini et al. 1986; Coaro 1987 [*G. Pistolesi*, *E. Coaro*, 1977, PI]; L. Viegi, 1987, PI; Garbari 2001; Pedullà & Garbari, 2004; Tomei et al. 2004; Bertacchi et al. 2009; Dell'Orso & Franchini 2009; Bertacchi et al. 2010; Arduini & Ercoli 2012; Macchia 2013 [*U. Macchia*, 2013, PI]; Petraglia 2013; Bertacchi & Lombardi 2014a; Lombardi 2015; Bertacchi & Lombardi 2016; Saggese 2016; Lazzeri 2021, 2022
Herbarium data: R. Bavazzano, C. Ricceri, 1963, FI; D. Marchetti, 1983, 2006, PI; M.L. Pedullà, 2000, PI; F. Roma-Marzio, 2012, PI; U. Macchia, 2013, PI
Field observations: M. D'Antraccoli, 2018; partecipanti all'escursione Wikiplantbase #Parco di Migliarino - San Rossore - Massaciuccoli, 2018; L. Pinzani, 2020, 2022; I. Arduini, 2022

Trachelospermum jasminoides (Lindl.) Lem.
Field observations: observed on iNaturalist in 2022, 2024
Only cultivated

Vinca major L. subsp. **major**
Ch rept - Eurimedit.
Literature data: Pedullà & Garbari, 2004; Bertacchi & Lombardi 2016; Peruzzi et al. 2017b [*F. Roma-Marzio, M. D'Antraccoli*, 2017, PI]
Herbarium data: T. Fiaschi, 2018, SIENA
Field observations: B. Pierini, 2014; partecipanti all'escursione Wikiplantbase #Parco di Migliarino - San Rossore - Massaciuccoli, 2018; L. Pinzani, 2022

Vincetoxicum hirundinaria Medik. subsp. **hirundinaria**
H scap - Eurasiat.
Literature data: Caruel 1860, under the name *V. officinale* Moench; Corti 1956, under the name *Cynanchum vincetoxicum* (L.) Pers. var. *typycum* Fiori; Garbari 2001
Field observations: L. Pinzani, 2020, I. Arduini, 2024

Boraginaceae

Anchusa azurea Mill.
H scap - Eurimedit.
Literature data: Caruel 1860, Corti 1956, Garbari 2001, under the name *A. italica* Retz.

Anchusa undulata L. subsp. **hybrida** (Ten.) Bég.
H scap - Stenomedit.
Literature data: Peruzzi et al. 2022 [*A. Mo*, 2022, PI]
Herbarium data: A. Mo, 2022, PI

Borago officinalis L.
T scap - Eurimedit.

Literature data: Caruel 1860; Garbari 2001
Field observations: B. Pierini, 2014; L. Pinzani, 2020, 2021, 2022

NC **Buglossoides arvensis** (L.) I.M.Johnst. subsp. **arvensis**
T scap - Eurimedit.
Literature data: Caruel 1860, under the name *Lithospermum arvense* L.

Cerinthe major L. subsp. **major**
H bienn - Stenomedit.
Literature data: Caruel 1860, under the name *C. aspera* Roth; Corti 1956 [*P. Fantozzi*, FI; *A. Fiori*, FI]; Corti 1956, under the name *C. major* L. var. *aspera* (Roth) Fiori in Fiori e Paol.; Garbari 2001; Pedullà & Garbari, 2004
Herbarium data: G. Cataldi, 1996, SIENA
Field observations: B. Pierini, 2014, 2017; L. Pinzani, 2021, 2022; A. Mo, 2022, 2023

Cynoglossum creticum Mill.
H bienn - Eurimedit.
Literature data: Caruel 1860, under the name *C. pictum* Ait.; Corti 1956, under the name *C. creticum* Mill. var. *typicum* Brand; Garbari 2001; Pedullà & Garbari, 2004

Echium italicum L. subsp. **italicum**
H bienn - Eurimedit.
Literature data: Caruel 1860
Field observations: B. Pierini, 2015; L. Pinzani, 2021

Echium plantagineum L.
T scap - Eurimedit.-Stenomedit.
Literature data: Caruel 1860
Herbarium data: Partecipanti all'escursione Wikiplantbase #Parco di Migliarino - San Rossore - Massaciuccoli, 2018, PI

Echium vulgare L. subsp. **vulgare**
H bienn - Europ.
Literature data: Caruel 1860; Coaro 1987; Garbari 2001

Heliotropium amplexicaule Vahl
Ch frut - Orig. S-America
Literature data: Criscuoli et al. 2011 [*B. Pierini*, 2011, FI, PI]; Arrigoni 2019
Field observations: I. Arduini, 2024

Heliotropium europaeum L.
T scap - Eurimedit.
Literature data: Caruel 1860; Baroni 1897-1908, under the name *Heliotropium supinum* L.; Garbari 2001; Arduini & Ercoli 2012
Herbarium data: L. Pinzani, 2021, Herb. Pinzani
Field observations: B. Pierini, 2014

NC **Lappula squarrosa** (Retz.) Dumort.
T scap (H bienn) - Paleotemp.

Literature data: Caruel 1860, under the name *Echinospermum lappula* Lehm.

NC **Lithospermum officinale** L.
H scap - Eurosiber.
Literature data: Caruel 1860

Myosotis arvensis (L.) Hill subsp. **arvensis**
T scap - Eurasiat.
Literature data: Baroni 1897-1908, under the name *M. intermedia* Link; Corti, 1951, 1956, under the name *M. arvensis* (L.) emend. Hill. var. *dumetorum* (Rouy) Stroh; Coaro 1987; Tomei et al. 2004
Field observations: L. Pinzani, 2022; I. Arduini, 2023

NC **Myosotis discolor** Pers. subsp. **discolor**
T scap - Eurimedit.
Literature data: Baroni 1897-1908, under the name *M. versicolor* Reich.; Corti 1956, under the name *M. collina* Hoffm.

Myosotis laxa Lehm. subsp. **cespitosa** (Schultz) Hyl. ex Nordh.
H bienn/T scap - Europ.-Subatl.
Literature data: Coaro 1987, Tomei et al. 2004, under the name *M. caespitosa* C.F.Schultz; Arrigoni, 2019

Myosotis ramosissima Rochel subsp. **ramosissima**
T scap - Europ.-W-Asiat.
Literature data: Caruel 1860, under the name *M. hispida* Schlecht. ; Garbari 2001; Lombardi 2015
Herbarium data: G. Gestri, 2018, PI2021
Field observations: B. Pierini, 2014; L. Pinzani, 2022

Myosotis scorpioides L. subsp. **scorpioides**
T scap - Europ.-W-Asiat.
Literature data: Caruel 1860, under the name *M. palustris* With.; Corti 1956 [*T. Caruel*, s.d., FI]; Corti 1956, under the name *M. scorpioides* L. emend. Hill. var. *pseudocaespitosa* (Fiori) Stroh, var. *strigulosa* (Rchb.) Stroh; Gellini et al. 1986; Garbari 2001
Herbarium data: F. Roma-Marzio, M. D’Antraccoli, 2015, PI; Partecipanti all'escursione Wikiplantbase #Parco di Migliarino - San Rossore - Massaciuccoli, 2018, PI
Field observations: I. Arduini, 2024

NC **Myosotis sicula** Guss.
H bienn/T scap - Eurimedit.-Sett.
Literature data: Baroni 1897-1908

Myosotis sylvatica Hoffm. subsp. **sylvatica**
H scap (H bienn) - Paleotemp.
Literature data: Gellini et al. 1986; Tomei et al. 2004

NC **Symphytum bohemicum** F.W.Schmidt
H scap - Europ.-Caucas.

Literature data: Caruel 1860, under the name *S. officinale* L.; Bottega & Garbari 2003 [*Bruno*, 1836, TO; *s. coll.*, 1862, BOLO; *Caruel*, 1874, RO; *Passerini*, 1923, PI Herb. Passerini]

Symphytum bulbosum K.F.Schimp.
G rhiz - SE-Europ.
Field observations: L. Pinzani, 2022

Symphytum orientale L.
H scap - Orig. W-Asia
Herbarium data: J. Franzoni, 2022, PI; L. Pinzani, 2022, Herb. Pinzani
Field observations: J. Franzoni, 2020

Symphytum tanaicense Steven
H scap - E-Europ.
Literature data: Peruzzi et al. 2001 [*Savi*, 1842, PI; *Caruel*, 1856, PI; *Grilli*, 1856, FI; *Beccari*, 1861, FI; *Beccar*i, 1862, FI; *Della Nave*, 1981, FI; *Fantozzi*, 1891, FI; *Savelli*, 1918, FI; *Passerini*, 1923, FI; *Passerini*, 1923, PI; *s.coll*, s.d., PI]; Bottega & Garbari 2003 [*Bruno*, 1836, TO; *Calandrini*, 1836, RO; *Savi*, 1842, FI; *Caruel*, 1856, FI; *Grilli*, 1856, FI; *Beccari*, 1861, FI; *Beccari*, 1862, FI; *Della Nave*, 1881, FI; *Fantozzi*, 1891, FI; *Savelli*, 1918, FI; *Passerini*, 1923, FI; *Passerini*, 1923, FI; *s. coll.*, s.d., PI]; Peruzzi et al. 2011
Assessed as CR in the Red List of the Italian Vascular Flora (Rossi et al. 2013)

NC **Symphytum tuberosum** L.
G rhiz - SE-Europ.
Literature data: Baroni 1897-1908

Convolvulaceae

Convolvulus arvensis L.
G rhiz - Cosmop.
Literature data: Caruel 1860; Coaro 1987; Garbari 2001; Pedullà & Garbari, 2004; Saggese 2016; Lazzeri 2021, 2022
Field observations: partecipanti all'escursione Wikiplantbase #Parco di Migliarino - San Rossore - Massaciuccoli, 2018; M. D'Antraccoli, 2019; L. Pinzani, 2020, 2021, 2022; H. Öhm, 2024

Convolvulus cantabrica L.
G rhiz - Eurimedit.
Field observations: B. Pierini, 2024

Convolvulus sepium L.
H scand - Paleotemp.
Literature data: Caruel 1860; Corti 1956; Gellini et al. 1986; Coaro 1987, Pedullà & Garbari, 2004, Tomei et al. 2004, Sani & Tomei 2006, Bertacchi et al. 2009, Saggese 2016, under the name *Calystegia sepium* (L.) R.Br.; Garbari 2001; Lazzeri 2021, 2022
Herbarium data: Partecipanti all'escursione Wikiplantbase #Parco di Migliarino - San Rossore - Massaciuccoli, 2018, PI
Field observations: B. Pierini, 2020; I. Arduini, 2024

NC **Convolvulus silvaticus** Kit.

H scand - SE-Europ.
Literature data: Caruel 1860, under the name *C. sylvestris* Waldst. et Kit.

Convolvulus soldanella L.
G rhiz - Cosmop.
Literature data: Baroni 1897-1908; Corti 1956 [*E. Levier*, s.d., FI; *C. Rossetti*, s.d., FI; *A. Biondi*, s.d., FI; *Picciuoli*, s.d., FI]; Corti 1956, 1970; Garbari 2001; Tomei et al. 2004, Bertacchi et al. 2009, Bertacchi et al. 2010, Bertacchi & Lombardi 2014a, Bertacchi & Lombardi 2014b, under the name *Calystegia soldanella* (L.) R.Br.
Herbarium data: L. Pinzani, 2020, Herb. Pinzani
Field observations: partecipanti all'escursione Wikiplantbase #Parco di Migliarino - San Rossore - Massaciuccoli, 2018; L. Pinzani, 2019, 2021, 2022

Cuscuta campestris Yunck.
T par - Orig. N-America
Literature data: Saggese 2016; Lazzeri in Buono et al. 2022
Field observations: B. Pierini, 2014, 2017

Cuscuta cesattiana Bertol.
T par - Orig. N-America
Literature data: Corti 1956; Sani & Tomei 2006; Bertacchi et al. 2009; Arrigoni, 2019

NC **Cuscuta epithymum** (L.) L. subsp. **epithymum**
T par - Eurasiat.
Literature data: Corti 1956, under the name *C. epithymum* (L.) Nathhorst subsp. *eu-epithymum* Beger var. *rubella* Engelm.

Dichondra micrantha Urb.
G rhiz - Orig. C-America
Literature data: Peruzzi et al. 2007 [*B. Pierini*, 2007, FI, PI]
Field observations: partecipanti all'escursione Wikiplantbase #Parco di Migliarino - San Rossore - Massaciuccoli, 2018; L. Pinzani, 2019, 2022; I. Arduini, 2024; H. Öhm, 2024

Ipomoea indica (Burm.) Merr.
Field observations: observed on iNaturalist in 2021, 2024
Only cultivated

Solanaceae

Alkekengi officinarum Moench
H scap - Eurasiat.-temp.
Literature data: Caruel 1860, Corti 1956 [*s. coll.*, s.d., FI], Corti 1956, under the name *Physalis alkekengi* L.
Field observations: I. Arduini, 2022

Atropa bella-donna L.
H scap - Medit.-Mont.
Literature data: Garbari 2001; Tomei et al. 2004; Arduini & Ercoli 2012
Herbarium data: F. Roma-Marzio, M. D’Antraccoli, 2017, PI

Field observations: I. Arduini, 2023

Datura stramonium L.
T scap - Orig. America
Literature data: Caruel 1860; Garbari 2001; Arduini & Ercoli 2012
Herbarium data: L. Pinzani, 2020, Herb. Pinzani
Field observations: F. Roma-Marzio, 2012; B. Pierini, 2014

NC **Hyoscyamus albus** L.
T scap/H bienn - Eurimedit.
Literature data: Caruel 1860, under the name *H. albus* L. var. *major* Mill.

NC **Hyoscyamus niger** L.
T scap/H bienn - Eurasiat.
Literature data: Caruel 1860

Petunia atkinsiana (Sweet) D.Don ex W.H.Baxter
Herbarium data: L. Pinzani, 2020, Herb. Pinzani

Physalis peruviana L.
Literature data: Lazzeri et al. in Alessandrini et al. 2013

Salpichroa origanifolia (Lam.) Baill.
Ch frut - Orig. S-America
Literature data: Arduini & Ercoli 2012; Lombardi 2015; Arduini & Alessandrini 2024
Herbarium data: L. Pinzani, 2020, Herb. Pinzani
Field observations: I. Arduini, 2010, 2021, 2022, 2023, 2024; Orlandi & Arduini 2010; B. Pierini, 2014, 2015; V. Lazzeri, 2017; F. Roma-Marzio, 2015, 2021; L. Peruzzi, 2017, 2018, 2019; L. Viegi, 2018, 2019, 2022, 2023; L. Pinzani, 2020, 2021, 2022; D. Ciccarelli, A. Mo, 2023; A. Mo, 2024; H. Öhm, 2024
This species is locally invasive, although reported as naturalized in Tuscany, so far (Galasso et al. 2024).

Solanum chenopodioides Lam.
T scap - Orig. S-America
Literature data: Soldano in Peruzzi et al. 2014 [*A. Soldano*, 2010, PI]; Saggese 2016; Lazzeri in Buono et al. 2022
Herbarium data: Partecipanti all'escursione Wikiplantbase #Parco di Migliarino - San Rossore - Massaciuccoli, 2018, PI; L. Pinzani, 2020, Herb. Pinzani
Field observations: B. Pierini, 2014; partecipanti all'escursione Wikiplantbase #Parco di Migliarino - San Rossore - Massaciuccoli, 2018; L. Pinzani, 2021, 2022

Solanum dulcamara L.
NP - Paleotemp.
Literature data: Caruel 1860; Corti 1956 [*M. Savelli*, s.d., FI]; Corti 1956; Gellini et al. 1986; Pedullà & Garbari, 2004; Bertacchi & Lombardi 2016; Lazzeri 2021
Field observations: partecipanti all'escursione Wikiplantbase #Parco di Migliarino - San Rossore - Massaciuccoli, 2018; L. Pinzani, 2021; I. Arduini, 2024

Solanum lycopersicum L.
Literature data: Arrigoni, 2019

Solanum nigrum L.
T scap - Cosmop.
Literature data: Caruel 1860; Corti 1956; Coaro 1987; Garbari 2001; Bertacchi et al. 2009; Arduini & Ercoli 2012; Lombardi 2015; Lazzeri in Buono et al. 2022
Herbarium data: Partecipanti all'escursione Wikiplantbase #Parco di Migliarino - San Rossore - Massaciuccoli, 2018, PI
Field observations: partecipanti all'escursione Wikiplantbase #Parco di Migliarino - San Rossore - Massaciuccoli, 2018; L. Pinzani, 2020, 2022

Solanum nitidibaccatum Bitter
Herbarium data: I. Arduini, 2024, PIAGR
Field observations: I. Arduini, 2024
New record for Tuscany as a casual alien.

Solanum pseudocapsicum L.
Literature data: Roma-Marzio & D'Antraccoli in Peruzzi et al. 2016 [*M. D'Antraccoli*, 2015, PI]

Solanum triflorum Nutt.
Literature data: Orlandi & Arduini 2010; Arduini & Ercoli 2012

Solanum villosum Mill.
T scap - Eurimedit.
Literature data: Orlandi & Arduini 2010; Arduini & Ercoli 2012; Särkinen et al. 2018 [*s.coll*, 1862, E]
Herbarium data: F. Roma-Marzio, L. Peruzzi, 2019, PI
Field observations: F. Roma-Marzio, 2013
Arrigoni (2019) reports *Sedum villosum* L. for the study area, referring in turn to Orlandi & Arduini (2010). Actually, the latter authors recorded *Solanum villosum* Mill., and never *Sedum villosum*.

Oleaceae

Fraxinus angustifolia Vahl subsp. **oxycarpa** (M.Bieb. ex Willd.) Franco & Rocha Afonso
P scap – Pont.
Literature data: Caruel 1860, Baroni 1897-1908, under the name *F. excelsior* L.; Corti 1956, under the name *F. oxycarpa* Willd.; Corti, 1970, under the name *F. excelsior* L.; Gellini et al. 1986, Coaro 1987 [*E. Coaro*, 1984, PI; *G. Pistolesi, E. Coaro*, 1984, PI], Pedullà & Garbari, 2004, Tomei et al. 2004, Petraglia 2013, under the name *F. oxycarpa* Bieb.; Arduini & Ercoli 2012; Lombardi 2015; Bertacchi & Lombardi 2016; Lazzeri 2021
Herbarium data: C. Del Prete, 1979, PI; R. Poli, 1989, PI; B. Ciacchi, G. Lorè, 1996, PI; F. Roma-Marzio, M. D’Antraccoli, 2017, PI
Field observations: L. Peruzzi, 2017; partecipanti all'escursione Wikiplantbase #Parco di Migliarino - San Rossore - Massaciuccoli, 2018; L. Pinzani, 2020, 2022; I. Arduini, 2023

Fraxinus excelsior L. subsp. **excelsior**
P scap – Europ.-Caucas.
Field observations: L. Peruzzi, 2021

Fraxinus ornus L. subsp. **ornus**
P scap - Eurimedit.
Literature data: Caruel 1860; Baroni 1897-1908; Corti, 1951, 1956; Coaro 1987 [*E. Coaro*, 1984, PI]; Tomei et al. 2004; Bertacchi et al. 2010; Bertacchi & Lombardi 2016
Herbarium data: O. Beccari, 1861, FI; A. Chiarugi, 1933, FI; B. Ciacchi, G. Lorè, 1996, PI
Field observations: L. Peruzzi, 2017

Ligustrum lucidum Aiton
P caesp/P scap - Orig. E-Asia
Literature data: Pedullà & Garbari, 2002 [*M.L. Pedullà*, 2000, PI]; LycopusPedullà & Garbari, 2004
Herbarium data: M.L. Pedullà, 2000, PI; F. Ruggiero, 2007, PI; Partecipanti all'escursione Wikiplantbase #Parco di Migliarino - San Rossore - Massaciuccoli, 2018, PI
Field observations: L. Pinzani, 2020, 2022, 2023

Ligustrum sinense Lour.
Field observations: observed on iNaturalist in 2024

Ligustrum vulgare L.
NP - Eurasiat.
Literature data: Caruel 1860; Corti 1956; Coaro 1987 [*E. Coaro*, 1984, PI]; Garbari 2001; Tomei et al. 2004; Bertacchi et al. 2010
Herbarium data: M. Grilli, 1856, FI; S. Sommier, 1899, FI; C. Del Prete, 1979, PI; R. Poli, 1989, PI; M.L. Pedullà, 2000, PI
Field observations: L. Peruzzi, 2017; partecipanti all'escursione Wikiplantbase #Parco di Migliarino - San Rossore - Massaciuccoli, 2018; L. Pinzani, 2020, 2022; I. Arduini, 2023

Olea europaea L.
P caesp/P scap - Stenomedit.
Literature data: Corti 1956, under the name *O. europaea* L. var. *oleaster* (Hoffmg. et Lk.) DC.
Field observations: F. Roma-Marzio, 2019; L. Pinzani, 2020, 2021
Often only cultivated in the study area.

Phillyrea angustifolia L.
P caesp - Steno-W-Medit.
Literature data: Coaro 1987 [*E. Coaro*, 1984, PI]; Garbari 2001; Tomei et al. 2004; Bertacchi et al. 2009; Bertacchi et al. 2010; Arduini & Ercoli 2012; Bertacchi & Lombardi 2014a
Herbarium data: M. Savelli, 1916; A. Chiarugi, 1938, FI; A. Chiarugi, R. Corti, 1951, FI; R. Poli, 1989, PI
Field observations: partecipanti all'escursione Wikiplantbase #Parco di Migliarino - San Rossore - Massaciuccoli, 2018; L. Pinzani, 2020, 2021

Phillyrea latifolia L.
P caesp - Stenomedit.
Literature data: Baroni 1897-1908, under the name *P. variabilis* Timb.-Lagr. ex Nyman; Corti 1956 [*P. Fantozzi*, FI]; Corti 1956, under the name *P. latifolia* L. (emend.) var. *media* (L.) C.K.Schneider forma *virgata* (Willd.) Fiori; Coaro 1987 [*E. Coaro*, 1984, PI]; Lazzeri 2021

Herbarium data: P. Fantozzi, 1891; S. Sommier, 1899, FI; M. Savelli, 1914, 1916; A. Chiarugi, 1938, FI
Field observations: partecipanti all'escursione Wikiplantbase #Parco di Migliarino - San Rossore - Massaciuccoli, 2018; L. Pinzani, 2022

Plantaginaceae

Antirrhinum majus L.
Ch frut - Orig. W-Medit.
Literature data: Caruel 1860; Corti 1956, under the name *A. majus* L. var. *typicum* Fiori; Garbari 2001

NC **Callitriche brutia** Petagna subsp. **brutia**
I rad - Paleotemp.
Literature data: Caruel 1860, under the name *C. hamulata* Kütz. ex W.D.J.Koch

NC **Callitriche obtusangula** Le Gall
I rad - Submedit.-Subatl.
Literature data: Corti 1956 [*P. Savi*, s.d., FI]; Corti 1956

NC **Callitriche palustris** L.
I rad - Circumbor.
Literature data: Caruel 1860, under the name *C. verna* Kütz.

NC **Callitriche platycarpa** Kütz.
I rad - C-Europ.
Literature data: Baroni 1897-1908, under the name *C. stagnalis* Scop. var. *platycarpa* Kütz.

Callitriche stagnalis Scop.
I rad - Eurasiat.
Literature data: Caruel 1860; Corti 1956; Garbari 2001
Herbarium data: Partecipanti all'escursione Wikiplantbase #Parco di Migliarino - San Rossore - Massaciuccoli, 2018, PI; J. Franzoni, M. Franzoni et D. Scalzo, 2019, Herb. Franzoni
Field observations: I. Arduini, 2024

NC **Callitriche truncata** Guss. subsp. **truncata**
I rad - Subatl.-Submedit.
Literature data: Baroni 1897-1908, under the name *C. autumnalis* L. var. *truncata* Guss.; Lastrucci et al. 2024 [*O. Beccari*, 1863, FI; *P. Savi*, 1869, FI]

Cymbalaria muralis G.Gaertn., B.Mey. & Scherb. subsp. **muralis**
H scap/Ch rept - Subcosmop.
Literature data: Caruel 1860, under the name *Linaria cymbalaria* Mill.
Field observations: F. Roma-Marzio, 2019; L. Pinzani, 2021, 2022; F. Roma-Marzio, 2021

Digitalis micrantha Roth ex Schweigg.
H scap - Endem. Ital.
Herbarium data: I. Arduini, 2024, PIAGR
Field observations: A. Spinelli, 2016

Gratiola officinalis L.
H scap - Circumbor.
Literature data: Caruel 1860; Corti 1956 [*Narducci*, FI]; Corti 1956, 1970; Gellini et al. 1986; Coaro 1987; Garbari 2001; Petraglia 2013; Lombardi 2015

Kickxia commutata (Bernh. ex Rchb.) Fritsch subsp. **commutata**
H rept - Stenomedit.
Literature data: Caruel 1860, under the name *Linaria graeca* Chav.; Corti 1956 [*T. Caruel*, s.d., FI]; Coaro 1987; Sani & Tomei 2006
Herbarium data: F. Picco, 1995, Herb. Picco

NC **Kickxia elatine** (L.) Dumort. subsp. **elatine**
T scap - Eurimedit.
Literature data: Caruel 1860, under the name *Linaria elatine* Mill.; Corti 1956 [*E. Levier*, s.d., FI; *M. Savelli*, s.d., FI]

Kickxia spuria (L.) Dumort. subsp**. integrifolia (**Brot.) R.Fern.
T scap - Eurasiat.
Field observations: B. Pierini, 2017

Kickxia spuria (L.) Dumort. subsp. **spuria**
T scap - Eurasiat.
Literature data: Caruel 1860, under the name *Linaria spuria* Mill.; Pedullà & Garbari, 2004
Herbarium data: J. Franzoni, 2019, Herb. Franzoni
Field observations: B. Pierini, 2014; J. Franzoni, 2019

Linaria pelisseriana (L.) Mill.
T scap - Medit.-Atl.
Herbarium data: Partecipanti all'escursione Wikiplantbase #Parco di Migliarino - San Rossore - Massaciuccoli, 2018, PI

Linaria vulgaris Mill. subsp. **vulgaris**
H scap - Eurasiat.
Literature data: Caruel 1860; Coaro 1987
Field observations: B. Pierini, 2014; L. Peruzzi, 2017, 2019; partecipanti all'escursione Wikiplantbase #Parco di Migliarino - San Rossore - Massaciuccoli, 2018; L. Pinzani, 2020, 2022

Misopates orontium (L.) Raf.
T scap - Paleotemp.
Literature data: Caruel 1860, under the name *Antirrhinum orontium* (L.) Rafin.
Herbarium data: F. Roma-Marzio, L. Peruzzi, 2019, PI
Field observations: L. Pinzani, 2022

NC **Plantago afra** L. subsp. **afra**
T scap - Stenomedit.
Literature data: Baroni 1897-1908, *P. cynops* Linn.

NC **Plantago arenaria** Waldst. & Kit.

T scap - SE-Europ.-S-Siber.
Literature data: Caruel 1860; Baroni 1897-1908; Corti 1956 [*Levier*, s.d., FI; *S. Sommier,* s.d., FI; *s. coll.*, s.d., FI]; Corti 1956, under the name *P. indica* L.

Plantago bellardii All. subsp. **bellardii**
T scap - S-Medit.
Literature data: Caruel 1860; Corti 1956; Lombardi 2015; Peruzzi et al. 2017b [*A. Sani, M. D'Antraccoli*, 2014, PI]
Field observations: L. Pinzani, 2022

NC **Plantago cornutii** Gouan
H ros - Asiat.
Literature data: Baroni 1897-1908; Corti, 1970

Plantago coronopus L.
T scap - Eurimedit.
Literature data: Caruel 1860; Corti 1956 [*P. Fantozzi*, FI]; Coaro 1987; Garbari 2001; Tomei et al. 2004; Sani & Tomei 2006; Bertacchi et al. 2009; Petraglia 2013; Saggese 2016; Lazzeri in Buono et al. 2022
Herbarium data: A. Sani, 2005, PI; D. Ciccarelli, 2012, PI
Field observations: partecipanti all'escursione Wikiplantbase #Parco di Migliarino - San Rossore - Massaciuccoli, 2018; L. Pinzani, 2018, 2021, 2022

Plantago lagopus L.
T scap - Stenomedit.
Literature data: Caruel 1860; Sani & Tomei 2006
Herbarium data: A. Sani, 2005, PI

Plantago lanceolata L.
H ros - Cosmop.
Literature data: Caruel 1860; Corti 1956 [*P. Savi*, s.d., FI]; Corti 1956, under the name *P. lanceolata* L. var. *communis* Schlectend.; Coaro 1987; Garbari 2001; Pedullà & Garbari, 2004; Saggese 2016; Lazzeri in Buono et al. 2022
Field observations: partecipanti all'escursione Wikiplantbase #Parco di Migliarino - San Rossore - Massaciuccoli, 2018; M. D'Antraccoli, 2019; L. Peruzzi, 2019; L. Pinzani, 2020, 2021, 2022

Plantago macrorhiza Poir.
H ros - Stenomedit.-Occid.
Herbarium data: M. Mannocci, 2001, Herb. Mus. Livorno

Plantago major L.
H ros - Subcosmop.
Literature data: Caruel 1860; Corti 1956 [*A. Fiori*, s.d., FI]; Coaro 1987, under the name *P. major* L. subsp. *intermedia* (Godr.) Lange; Pedullà & Garbari, 2004; Tomei et al. 2004; Sani & Tomei 2006; Petraglia 2013; Saggese 2016; Arrigoni, 2020; Lazzeri in Buono et al. 2022
Field observations: partecipanti all'escursione Wikiplantbase #Parco di Migliarino - San Rossore - Massaciuccoli, 2018; M. D'Antraccoli, 2019; L. Pinzani, 2020, 2021, 2022

Veronica acinifolia L.

T scap - CSE-Europ.
Literature data: Coaro 1987

Veronica anagallis-aquatica L. subsp. **anagallis-aquatica**
H scap - Cosmop.
Literature data: Caruel 1860
Herbarium data: F. Roma-Marzio, M. D'Antraccoli, 2015, PI
Field observations: B. Pierini, 2015; L. Pinzani, 2021, 2022

Veronica anagalloides Guss. subsp. **anagalloides**
T scap - Eurimedit.
Literature data: Lazzeri in Buono et al. 2022

Veronica arvensis L.
T scap - Subcosmop.
Literature data: Caruel 1860; Corti, 1954, 1956, under the name *V. arvensis* L. var. *typica* Fiori; Garbari 2001; Arduini & Ercoli 2012; Bonari et al. 2019
Herbarium data: F. Roma-Marzio, L. Peruzzi, 2019, PI; L. Pinzani, 2020, Herb. Pinzani
Field observations: L. Pinzani, 2021, 2022; I. Arduini, 2024; H. Öhm, 2024

NC **Veronica beccabunga** L. subsp. **beccabunga**
H rept - Eurasiat.
Literature data: Caruel 1860

Veronica chamaedrys L. subsp. **chamaedrys**
H scap - Eurosiber.
Literature data: Coaro 1987

Veronica cymbalaria Bodard subsp. **cymbalaria**
T scap - Eurimedit.
Literature data: Caruel 1860; Pedullà & Garbari, 2004
Herbarium data: F. Roma-Marzio, L. Peruzzi, 2019, PI
Field observations: B. Pierini, 2014; F. Roma-Marzio, 2014; J. Franzoni, 2019; L. Pinzani, 2020, 2021, 2022

Veronica filiformis Sm.
Ch rept/H rept - Orig. Europ.-Caucas.
Literature data: Coaro 1987

Veronica hederifolia L.
T scap - Eurasiat.
Literature data: Caruel 1860
Field observations: B. Pierini, 2014; J. Franzoni, 2019; L. Pinzani, 2021, 2022

Veronica montana L.
H rept - Centroeurop.
Literature data: Caruel 1860; Corti 1956 [*T. Caruel*, s.d., FI; *P. Fantozzi*, s.d., FI]; Corti 1956; Gellini et al. 1986; Garbari 2001; Tomei et al. 2004
Field observations: I. Arduini, 2024

Veronica officinalis L.
H rept - Eurasiat.
Literature data: Caruel 1860; Corti 1956 [*P. Savi*, s.d., FI]; Corti 1956; Gellini et al. 1986; Garbari 2001; Arduini & Ercoli 2012

Veronica peregrina L.
Literature data: Baroni 1897-1908
Herbarium data: L. Pinzani, 2021, Herb. Pinzani

Veronica persica Poir.
T scap - Orig. W-Asia
Literature data: Caruel 1860; Corti 1956; Coaro 1987; Garbari 2001; Lazzeri in Buono et al. 2022
Field observations: B. Pierini, 2014; partecipanti all'escursione Wikiplantbase #Parco di Migliarino - San Rossore - Massaciuccoli, 2018; M. D'Antraccoli, 2019; J. Franzoni, 2019; L. Pinzani, 2020, 2021, 2022; I. Arduini, 2024

Veronica polita Fr.
T scap - Subcosmop.
Literature data: Caruel 1860, under the name *V. didyma* Ten.
Herbarium data: L. Pinzani, 2021, Herb. Pinzani
Field observations: F. Roma-Marzio, 2015

Veronica praecox All.
T scap - C-Europ.-Submedit.
Literature data: Lombardi 2015; Peruzzi et al. 2017b [*A. Sani*, 2015, PI]

NC **Veronica prostrata** L.
H caesp - Eurasiat.
Literature data: Caruel 1860

Veronica scutellata L.
H scap - Circumbor.
Literature data: Caruel 1860; Lombardi 2015

Veronica serpyllifolia L.
H rept - Circumbor.-Subcosmop.
Literature data: Caruel 1860; Corti 1956; Garbari 2001; Tomei et al. 2004
Field observations: B. Pierini, 2020, I. Arduini, 2024

Scrophulariaceae

Scrophularia auriculata L. subsp. **auriculata**
H scap - Subatl.
Literature data: Corti 1956 [*M. Savelli*, s.d., FI; *A. Vaccari*, s.d., FI]; Corti 1956; Garbari 2001; Arrigoni, 2020
Field observations: B. Pierini, 2006, 2020

Scrophularia canina L.

H scap - Eurimedit.
Literature data: Corti 1956, under the name *S. canina* L. var. *bicolor* (Sibth. et Sm.) Fiori; Garbari 2001
Field observations: J. Franzoni, 2021

Scrophularia nodosa L.
H scap - Circumbor.
Literature data: Corti 1956; Gellini et al. 1986; Garbari 2001; Tomei et al. 2004

NC **Scrophularia peregrina** L.
T scap - Stenomedit.
Literature data: Caruel 1860

NC **Scrophularia umbrosa** Dumort. subsp. **umbrosa**
H scap - Subatl.
Literature data: Caruel 1860, under the name *S. aquatica* L.

Verbascum blattaria L.
H bienn - Paleotemp.
Literature data: Caruel 1860; Baroni 1897-1908; Corti 1956 [*S. Sommier*, s.d., FI]; Coaro 1987
Field observations: B. Pierini, 2014, 2016; L. Pinzani, 2020

Verbascum phoeniceum L.
H scap - S-Europ.-Sudsiber.
Literature data: Baroni 1897-1908; Garbari 2001

Verbascum pulverulentum Vill.
H bienn - S-Europ.
Literature data: Caruel 1860, under the name *V. floccosum* Waldst. et Kit.
Field observations: B. Pierini, 2016

Verbascum sinuatum L.
H bienn - Eurimedit.
Literature data: Caruel 1860; Corti, 1951, 1956; Coaro 1987; Garbari 2001; Pedullà & Garbari, 2004; Sani & Tomei 2006; Lazzeri in Buono et al. 2022
Field observations: L. Peruzzi, 2015; partecipanti all'escursione Wikiplantbase #Parco di Migliarino - San Rossore - Massaciuccoli, 2018; R. Righini, 2021; F. Roma-Marzio, 2021; H. Öhm, 2024

Verbascum thapsus L. subsp. **thapsus**
H bienn - Europ.-Caucas.
Literature data: Caruel 1860; Corti, 1951, 1956; Pedullà & Garbari, 2004; Lazzeri in Buono et al. 2022
Field observations: partecipanti all'escursione Wikiplantbase #Parco di Migliarino - San Rossore - Massaciuccoli, 2018

Linderniaceae

Lindernia dubia (L.) Pennell

T scap - Orig. N-America
Literature data: Peruzzi et al. 2007 [*B. Pierini*, 2007, FI, PI; *J.-M. Tison*, 2007, Herb. Tison]

Lamiaceae

NC **Ajuga chamaepitys** (L.) Schreb. subsp. **chamaepitys**
T scap/H bienn - Eurimedit.
Literature data: Caruel 1860

Ajuga reptans L.
H rept - Europ.-Caucas.
Literature data: Caruel 1860; Corti, 1954, 1956, under the name *A. reptans* L. var. *typica* Fiori; Gellini et al. 1986; Coaro 1987; Garbari 2001; Tomei et al. 2004; Bertacchi & Lombardi 2016
Herbarium data: J. Franzoni, 2019, D. Scalzo, Herb. Franzoni
Field observations: A. Giacò, J. Franzoni, L. Pinzani, 2021; I. Arduini, 2023

Ballota nigra L. subsp. **meridionalis** (Bég.) Bég.
H scap - Submedit.-Subatl.
Literature data: Caruel 1860, Corti 1956, under the name *B. nigra* L.; Pedullà & Garbari, 2004, under the name *B. nigra* L. subsp. *foetida* Hayek
Herbarium data: Partecipanti all'escursione Wikiplantbase #Parco di Migliarino - San Rossore - Massaciuccoli, 2018, PI
Field observations: B. Pierini, 2014; L. Pinzani, 2020; I. Arduini, 2024

Betonica officinalis L.
H scap - Europ.-Caucas.
Literature data: Caruel 1860; Corti 1956, under the name *Stachys officinalis* (L.) Trev. var. *serotina* (Host.) Bèguinot; Gellini et al. 1986; Coaro 1987, Garbari 2001, under the name *Stachys officinalis* (L.) Trevisan

Clinopodium menthifolium (Host) Merino subsp. **menthifolium**
H scap - Europ.
Literature data: Coaro 1987, under the name *Calamintha sylvatica* Bromf. subsp. *sylvatica*; Orlandi & Arduini 2010, Arduini & Ercoli 2012, under the name *Clinopodium nepeta* (L.) Kuntze subsp. s*ylvaticum* (Bromf.) Peruzzi & F.Conti

Clinopodium nepeta (L.) Kuntze subsp. **nepeta**
H scap/Ch suffr - Medit.-Mont.
Literature data: Caruel 1860, under the name *Calamintha parviflora* Lam.; Baroni 1897-1908, under the name *Calamintha officinalis* Moench; Coaro 1987, Garbari 2001, Tomei et al. 2004, Sani & Tomei 2006, Arduini & Ercoli 2012, Lombardi 2015, Bonari et al. 2019, under the name *Calamintha nepeta* (L.) Savi subsp. *nepeta*
Field observations: B. Pierini, 2014; L. Peruzzi, 2019; L. Pinzani, 2021, 2022; I. Arduini, 2024

Clinopodium vulgare L. subsp. **vulgare**
H scap - Circumbor.
Literature data: Caruel 1860, under the name *Calamintha clinopodium* Benth.; Gellini et al. 1986; Coaro 1987

Herbarium data: Partecipanti all'escursione Wikiplantbase #Parco di Migliarino - San Rossore - Massaciuccoli, 2018, PI

NC **Galeopsis angustifolia** Ehrh. ex Hoffm. subsp. **angustifolia**
H scap - Europ.
Literature data: Caruel 1860, under the name *G. ladanum* L.

Glechoma hederacea L.
H rept - Circumbor.
Literature data: Caruel 1860; Coaro 1987

NC **Lamium album** L. subsp. **album**
H scap - Eurasiat.-Temp.
Literature data: Caruel 1860

Lamium amplexicaule L.
T scap - Paleotemp.
Literature data: Caruel 1860
Field observations: B. Pierini, 2014; L. Pinzani, 2021, 2022

NC **Lamium bifidum** Cirillo subsp**. bifidum**
T scap - Stenomedit.
Literature data: Caruel 1860; Corti 1956 [*P. Savi*, s.d., FI]; Corti 1956
The lectotype of this taxa was collected from the forests near Pisa (Peruzzi et al. 2019).

Lamium maculatum L.
H scap - Eurasiat.-Temp.
Literature data: Caruel 1860; Bonari et al. 2019
Field observations: L. Pinzani, 2022

Lamium purpureum L.
T scap - Eurasiat.
Literature data: Caruel 1860; Coaro 1987; Garbari 2001; Pedullà & Garbari, 2004
Field observations: B. Pierini, 2014; L. Pinzani, 2021, 2022

Lavandula angustifolia Mill.
Field observations: observed on iNaturalist in 2021
Only cultivated

Lycopus europaeus L.
H scap/I rad - Paleotemp.-Circumbor.
Literature data: Caruel 1860; Baroni 1897-1908; Corti 1956 [*S. Sommier*, s.d., FI]; Corti 1956, under the name *L. europaeus* L. f. *pubescens* Benth. in DC.; Gellini et al. 1986; Coaro 1987; Garbari 2001; Pedullà & Garbari, 2002 [*M.L. Pedullà*, 1999, PI]; Pedullà & Garbari, 2004; Lazzeri 2021
Field observations: L. Pinzani, 2020

Lycopus exaltatus L.f.
H scap - Eurosiber.

Literature data: Arrigoni, 2020

Marrubium incanum Desr.
H scap – NE-Medit.
Literature data: Tomei et al. 2004

Melissa officinalis L. subsp. **altissima** (Sm.) Arcang.
H scap - Stenomedit.
Literature data: Caruel 1860, Gellini et al. 1986, under the name *Melissa officinalis* L.; Corti 1956, under the name *Melissa officinalis* L. var. *officinalis* Briq.; Garbari 2001; Pedullà & Garbari, 2004, under the name *Melissa officinalis* L. subsp. *officinalis*
Field observations: J. Molina, 2007; L. Pinzani, 2021; I. Arduini, 2024

Mentha aquatica L. subsp. **aquatica**
H scap - Paleotemp.
Literature data: Caruel 1860; Baroni 1897-1908; Corti 1956; Gellini et al. 1986; Coaro 1987; Tomei et al. 2004; Lombardi 2015; Lazzeri in Buono et al. 2022
Field observations: L. Pinzani, 2021

Mentha longifolia (L.) L.
H scap - Paleotemp.
Literature data: Baroni 1897-1908, under the name *M. sylvestris* L. var. *glabra* Parl.; Garbari 2001; Arrigoni, 2020 under the name *M. longifolia* (L.) L. var. *glabra* Parl.

Mentha microphylla K.Koch
H scap - E-Medit.
Literature data: Arrigoni, 2020

Mentha pulegium L. subsp. **pulegium**
H scap - Eurimedit.-Subcosmop.
Literature data: Caruel 1860; Coaro 1987; Garbari 2001; Tomei et al. 2004

Mentha spicata L.
H scap - Eurimedit.
Literature data: Pedullà & Garbari, 2004; Saggese 2016
Herbarium data: L. Pinzani, 2020, Herb. Pinzani
Field observations: L. Peruzzi, 2019

Mentha suaveolens Ehrh. subsp. **suaveolens**
H scap - Eurimedit.
Literature data: Caruel 1860, under the name *M. rotundifolia* L. ; Garbari 2001; Lazzeri in Buono et al. 2022

Mentha ×rotundifolia (L.) Huds.
Literature data: Corti 1956; Garbari 2001

Micromeria graeca (L.) Benth. ex Rchb. subsp. **graeca**
Ch suffr - Stenomedit.
Literature data: Caruel 1860; Baroni 1897-1908, under the name *Satureja graeca* L.

Herbarium data: F. Roma-Marzio, M. D’Antraccoli, 2017, PI
Field observations: L. Peruzzi, 2019

NC **Nepeta cataria** L.
H scap - E-Medit.-Turan.
Literature data: Caruel 1860; Corti 1956 [*P. Savi*, s.d., FI]

Prunella laciniata (L.) L.
H scap - Eurimedit.
Literature data: Garbari 2001; Arduini & Ercoli 2012

Prunella vulgaris L. subsp. **vulgaris**
H scap - Circumbor.
Literature data: Caruel 1860, under the name *Brunella vulgaris* L.; Corti 1956; Gellini et al. 1986; Coaro 1987; Tomei et al. 2004; Arduini & Ercoli 2012; Lombardi 2015; Bertacchi & Lombardi 2016
Field observations: B. Pierini, 2017; partecipanti all'escursione Wikiplantbase #Parco di Migliarino - San Rossore - Massaciuccoli, 2018; I. Arduini, 2024

Salvia clandestina L.
H scap - Stenomedit.
Literature data: Caruel 1860, under the name *S. multifida* Sibth. et Sm.; Corti 1956 [*M. Savelli*, s.d., FI]; Corti 1956 [*G. Savi*, s.d., FI; *P. Savi*, s.d., FI]; Garbari 2001
Herbarium data: L. Pinzani, A. Giacò, J. Franzoni, 2021, Herb. Pinzani
Field observations: A. Giacò, J. Franzoni, L. Pinzani, 2021; L. Pinzani, 2022

NC **Salvia pratensis** L.
H scap - Eurimedit.
Literature data: Caruel 1860

Salvia rosmarinus Spenn.
NP/P caesp - Stenomedit.
Literature data: Corti 1956, under the name *Rosmarinus officinalis* L.; Garbari 2001
Herbarium data: M.L. Pedullà, 2000, PI
Field observations: U. Macchia, 2016; A. Giacò, J. Franzoni, L. Pinzani, 2021

Salvia verbenaca L.
H scap - Medit.-Atl.
Literature data: Caruel 1860; Corti 1956 [*P. Savi*, s.d., FI; *A. Fiori*, s.d., FI]; Corti 1956; Coaro 1987; Garbari 2001; Tomei et al. 2004
Herbarium data: F. Roma-Marzio, L. Peruzzi, 2019, PI
Field observations: L. Pinzani, 2020, 2022

Salvia yangii B.T.Drew
Field observations: observed on iNaturalist in 2024
Only cultivated

Scutellaria columnae All. subsp. **columnae**
H scap - NE-Medit.-Mont.

Literature data: Caruel 1860; Corti 1956; Coaro 1987

Scutellaria galericulata L.
G rhiz - Circumbor.
Literature data: Caruel 1860; Gellini et al. 1986; Garbari 2001; Tomei et al. 2004; Petraglia 2013

Scutellaria hastifolia L.
G rhiz - SE-Europ.
Literature data: Caruel 1860; Baroni 1897-1908; Coaro 1987

Stachys germanica L. subsp. **salviifolia** (Ten.) Gams
H scap - NE-Medit.
Literature data: Baroni 1897-1908, under the name *S. italica* Mill.; Falciani 1997 [*P. Pellegrini*, 1890, PI]

Stachys montana (L.) Peruzzi & Bartolucci subsp. **montana**
T scap - Medit.-Turan.
Literature data: Garbari 2001, Tomei et al. 2004, under the name *Sideritis montana* L.

Stachys palustris L.
H scap - Circumbor.
Literature data: Caruel 1860; Baroni 1897-1908; Corti 1956 [*P. Savi*, s.d., FI; *A. Biondi*, s.d., FI]; Corti 1956; Lazzeri 2021

Stachys recta L. subsp. **recta**
H scap - Orof. N-Medit.
Literature data: Baroni 1897-1908; Corti 1956 [*S. Sommier*, s.d., FI]
Field observations: observed on iNaturalist in 2021

Stachys romana (L.) E.H.L.Krause
T scap - Stenomedit.
Literature data: Caruel 1860, Coaro 1987, Garbari 2001, Sani & Tomei 2006 [*A. Sani*, 2005, PI], Arduini & Ercoli 2012, under the name *Sideritis romana* L.

Teucrium capitatum L. subsp. **capitatum**
Ch suffr - Stenomedit.
Literature data: Caruel 1860, Corti, 1951, 1954, 1956, Garbari 2001, Tomei et al. 2004, Sani & Tomei 2006, under the name *T. polium* L.; Lombardi 2015
Herbarium data: D. Ciccarelli, 2011, PI
Field observations: L. Pinzani, 2022

Teucrium chamaedrys L. subsp. **chamaedrys**
Ch suffr - Eurimedit.
Literature data: Caruel 1860; Corti, 1951, 1956; Coaro 1987; Garbari 2001; Tomei et al. 2004; Sani & Tomei 2006; Arduini & Ercoli 2012; Lombardi 2015; Bonari et al. 2019
Field observations: I. Arduini, 2024

Teucrium fruticans L. subsp. *fruticans*
Field observations: observed on iNaturalist in 2020, 2024

Only cultivated

Teucrium scordium L. subsp. **scordioides** (Schreb.) Arcang.
H scap - Europ.
Literature data: Caruel 1860, under the name *T. scordium* L.; Corti 1956 [*Levier*, s.d., FI]; Corti 1956, under the name *T. scordioides* Schreb.; Sani & Tomei 2006 [*A. Sani*, 2005, PI], under the name *T. scordium* L.

Vitex agnus-castus L.
P caesp/P scap - Orig. Medit.-Turan.
Literature data: Caruel 1860; Corti 1956 [*P. Savi*, s.d., FI; *Marcucci*, s.d., FI]; Garbari 2001; Tomei et al. 2004

NC *Ziziphora capitata* L. subsp. *capitata*
Literature data: Caruel 1860

Paulowniaceae

Paulownia tomentosa (Thunb.) Steud.
Field observations: observed on iNaturalist in 2022, 2023
Only cultivated

Orobanchaceae

Bellardia trixago (L.) All.
T scap – Euri-Medit.
Field observations: observed on iNaturalist in 2022, 2024

Bellardia viscosa (L.) Fisch. & C.A.Mey.
T scap - Medit.-Atl.
Literature data: Caruel 1860, under the name *Eufragia viscosa* Benth.; Corti 1956, under the name *Bartsia viscosa* L.; Coaro 1987, Garbari 2001, under the name *Parentucellia viscosa* (L.) Caruel; Saggese 2016
Herbarium data: Partecipanti all'escursione Wikiplantbase #Parco di Migliarino - San Rossore - Massaciuccoli, 2018, PI
Field observations: B. Pierini, 2015, 2017; partecipanti all'escursione Wikiplantbase #Parco di Migliarino - San Rossore - Massaciuccoli, 2018; L. Pinzani, 2021, 2022; A. Mo, 2023

NC **Melampyrum arvense** L. subsp. **arvense**
T scap - Eurasiat.
Literature data: Caruel 1860

NC **Odontites luteus** (L.) Clairv. subsp. **luteus**
T scap - Eurimedit.
Literature data: Caruel 1860; Montelucci 1964

Odontites vernus (Bellardi) Dumort. subsp. **serotinus** Corb.
T scap - Eurasiat.

Literature data: Caruel 1860, under the name *O. vulgaris* Stev. α *verna*; β *serotina*; Baroni 1897-1908, under the name *O. serotina* Reich.; Coaro 1987, under the name *O. serotina* Dumort

Orobanche artemisiae-campestris Gaudin
T par - Eurimedit.
Literature data: Sani & Tomei 2006, under the name *O. loricata* Rchb.; Saggese 2016

Orobanche crenata Forssk.
T par - Eurimedit.-Turan.
Literature data: Caruel 1860, under the name *O. pruinosa* Lap.
Field observations: partecipanti all'escursione Wikiplantbase #Parco di Migliarino - San Rossore - Massaciuccoli, 2018

Orobanche hederae Vaucher ex Duby
T par - Eurimedit.
Literature data: Baroni 1897-1908; Garbari 2001
Field observations: I. Arduini, 2024

Orobanche litorea Guss.
T par - Medit.
Literature data: Peruzzi et al. 2017a [A. *Bertacchi, M. D'Antraccoli*, 2016, PI]

Orobanche minor Sm.
T par - Subcosmop.
Literature data: Caruel 1860, under the name *O. yuccae* P.Sav.; Coaro 1987
Herbarium data: J. Franzoni, 2019, Herb. Franzoni
Field observations: partecipanti all'escursione Wikiplantbase #Parco di Migliarino - San Rossore - Massaciuccoli, 2018

Parentucellia latifolia (L.) Caruel
T scap - Eurimedit.
Literature data: Caruel 1860, under the name *Eufragia latifolia* Gris.; Corti 1956 [*P. Savi*, s.d., FI]; Corti 1956, under the name *Bartsia latifolia* (L.) Sibth. & Sm.
Herbarium data: L. Pinzani, 2021, Herb. Pinzani
Field observations: A. Giacò, J. Franzoni, L. Pinzani, 2021; B. Pierini, 2021

Phelipanche nana (Reut.) Soják
T par - Paleotemp.
Literature data: Coaro 1987 [*E. Coaro*, 1984, PI], under the name *Orobanche ramosa* L. subsp. *mutelii* (F.W.Schultz) Coutinho

Phelipanche purpurea (Jacq.) Soják
T par - Europ.-Sudsiber.
Literature data: Caruel 1860, under the name *Phelipaea coerulea* C.A.Mey; Garbari 2001, Tomei et al. 2004, under the name *Orobanche purpurea* Jacq.
Field observations: L. Pinzani, 2021

NC **Phelipanche ramosa** (L.) Pomel
T par - Paleotemp.

Literature data: Caruel 1860, under the name *Phelipaea ramosa* C.A.Mey.

NC **Rhinanthus alectorolophus** (Scop.) Pollich subsp. **alectorolophus**
T scap - Europ.
Literature data: Arrigoni 2020

Lentibulariaceae

NC **Utricularia australis** R.Br.
I nat - Circumbor.
Literature data: Caruel 1860
Herbarium data: Della Nave, 1881, FI; M. Savelli, 1915, FI

NC **Utricularia minor** L.
I nat - C.Europ.
Herbarium data: s. coll., 1881, FI

NC **Utricularia vulgaris** L.
I nat - Circumbor.
Literature data: Caruel 1860

Acanthaceae

Acanthus mollis L. subsp. mollis
H scap - Orig. W-Medit.
Field observations: L. Pinzani, 2020, 2022
Bignoniaceae

Campsis radicans (L.) Bureau
Field observations: observed on iNaturalist in 2020, 2024
Only cultivated

Catalpa bignonioides Walter
Field observations: observed on iNaturalist in 2024
Only cultivated

Verbenaceae

Duranta erecta L.
Field observations: observed on iNaturalist in 2024
Only cultivated

Lantana camara L. subsp. *camara*
Field observations: observed on iNaturalist in 2021, 2024
Only cultivated

Phyla canescens (Kunth) Greene
Literature data: Coaro 1987, Arrigoni, 2020 under the name *Lippia canescens* Kunth
Field observations: J. Franzoni, 2020; H. Öhm, 2024

Phyla nodiflora (L.) Greene
H rept – Orig. Pantrop.-Subtrop.
Literature data: Caruel 1860, Baroni 1897-1908, Corti 1956 [*Targioni-Tozzetti*, s.d., FI; *S. Sommier*, s.d., FI; *Picciuoli*, s.d., FI; *D. Bergeest*, s.d., FI; *F. Schultz*, s.d., FI*; s. coll.*, s.d., FI]; Garbari 2001, Tomei et al. 2004, Bertacchi et al. 2007, Petraglia 2013, under the name *Lippia nodiflora* (L.) Michx.; Sani & Tomei 2006
Herbarium data: A. Sani, 2005, PI

Verbena bonariensis L.
Literature data: Monti & Tomei 1983 [*G. Monti, P.E. Tomei*, 1978, PI]
Field observations: B. Pierini, 2020

Verbena brasiliensis Vell.
T scap - Orig. America
Herbarium data: Partecipanti all'escursione Wikiplantbase #Parco di Migliarino - San Rossore - Massaciuccoli, 2018, PI; L. Pinzani, 2021, Herb. Pinzani
Field observations: B. Pierini, 2024

Verbena officinalis L.
H scap - Paleotemp.
Literature data: Caruel 1860; Coaro 1987; Garbari 2001; Pedullà & Garbari, 2004; Tomei et al. 2004; Petraglia 2013; Saggese 2016; Lazzeri in Buono et al. 2022
Field observations: partecipanti all'escursione Wikiplantbase #Parco di Migliarino - San Rossore - Massaciuccoli, 2018; L. Peruzzi, 2019; L. Pinzani, 2020, 2021, 2022; H. Öhm, 2024

Campanulaceae

Campanula erinus L.
T scap - Stenomedit.
Literature data: Caruel 1860
Herbarium data: F. Roma-Marzio, L. Peruzzi, 2019, PI
Field observations: B. Pierini 2015

Campanula rapunculus L.
H bienn - Paleotemp.
Literature data: Caruel 1860; Corti, 1954, 1956, under the name *C. rapunculus* L. var. *typic*a Fiori; Coaro 1987; Garbari 2001
Field observations: partecipanti all'escursione Wikiplantbase #Parco di Migliarino - San Rossore - Massaciuccoli, 2018; I. Arduini, 2024

NC **Jasione montana** L.
H bienn/T scap - Europ.-Caucas.
Literature data: Caruel 1860

Legousia speculum-veneris (L.) Chaix subsp. **speculum-veneris**
T scap - Eurimedit.
Literature data: Caruel 1860, under the name *Specularia speculum veneris* A.DC.; Pierini in Peruzzi et al. 2011 [*B. Pierini*, 2011, PI]

NC **Solenopsis laurentia** (L.) C.Presl
T scap - Stenomedit.-Occid.
Literature data: Caruel 1860, under the name *Laurentia michelii* A.DC.

Menyanthaceae

Ex **Menyanthes trifoliata** L.
I rad - Circumbor.
Literature data: Corti 1956 [*Narducci*, FI]

Ex **Nymphoides peltata** (S.G.Gmel.) Kuntze
I rad - Eurasiat.-Temp.
Literature data: Caruel 1860, *Limnanthemum nymphoides* Hoffmanns. & Link

Asteraceae

NC **Achillea ageratum** L.
H scap - Stenomedit.-Occid.
Literature data: Caruel 1860

Achillea maritima (L.) Ehrend. & Y.P.Guo subsp. **maritima**
Ch suffr - Medit.-Atl.
Literature data: Caruel 1860, under the name *Diotis candidissima* Desf.; Corti 1956 [*G. Savi*, s.d., FI; *Biondi*, s.d., FI]; Corti 1956, under the name *Diotis maritima* (L.) Sm.; Garbari 2001, Tomei et al. 2004, Sani & Tomei 2006, Bertacchi et al. 2009, Bertacchi & Lombardi 2014b, under the name *Otanthus maritimus* (L.) Hoffmanns & Link
Herbarium data: D. Ciccarelli & M. Sammartino, 2009, PI

Achillea millefolium L. subsp. **millefolium**
H scap - Eurosiber.
Literature data: Caruel 1860; Garbari 2001

Ambrosia artemisiifolia L.
T scap - Orig. N-America
Literature data: Lazzeri et al. in Alessandrini et al. 2013

Ambrosia psilostachya DC.
G rhiz - Orig. N-America
Literature data: Lazzeri in Buono et al. 2022

Anacyclus radiatus Loisel. subsp. **radiatus**
T scap - Stenomedit.
Literature data: Caruel 1860; Saggese 2016
Herbarium data: L. Pinzani, 2021, Herb. Pinzani
Field observations: partecipanti all'escursione Wikiplantbase #Parco di Migliarino - San Rossore - Massaciuccoli, 2018

Andryala integrifolia L.

T scap - W-Medit.
Literature data: Caruel 1860, under the name *A. sinuata* L.
Field observations: partecipanti all'escursione Wikiplantbase #Parco di Migliarino - San Rossore - Massaciuccoli, 2018; F. Roma-Marzio, 2020; L. Pinzani, 2022

Anthemis arvensis L. subsp. **arvensis**
T scap/H scap - Stenomedit.
Literature data: Caruel 1860; Coaro 1987; Garbari 2001
Field observations: partecipanti all'escursione Wikiplantbase #Parco di Migliarino - San Rossore - Massaciuccoli, 2018; I. Arduini, 2024

Anthemis cotula L.
T scap - Eurimedit.
Literature data: Caruel 1860; Corti 1956; Garbari 2001

Anthemis maritima L. subsp. **maritima**
H scap - W-Medit.
Literature data: Bertacchi et al. 2009; Ciccarelli et al. 2013 [*D. Ciccarelli*, 2011, PI]
Field observations: R. Righini, 2021; L. Pinzani, 2022

Arctium minus (Hill) Bernh.
H bienn - Europ. (Eurimedit.)
Literature data: Caruel 1860, under the name *Lappa officinalis* All.; Corti 1956; Garbari 2001

Artemisia annua L.
T scap - Orig. Eurasiat.
Literature data: Anzalone 1979; Atzori et al. 2009 [*D. Marchetti, A. Soldano*, 1983, GE, SIENA; *L. Peruzzi*, 2008, PI]
Herbarium data: F. Roma-Marzio, M. D’Antraccoli, 2017, PI; L. Pinzani, 2020, Herb. Pinzani; I. Arduini, 2023, PIAGR
Field observations: B. Pierini, 2017

Artemisia caerulescens L. subsp. **caerulescens**
Ch suffr - Eurimedit.
Literature data: Caruel 1860, under the name *A. maritima* L.; Coaro 1987, under the name *A. cretacea* (Fiori) Pign.; Garbari 2001; Tomei et al. 2004; Bertacchi et al. 2007, under the name *A. coerulescens* L. var. *palmata* Lam.; Petraglia 2013; Saggese 2016
Herbarium data: L. Pinzani, 2020, Herb. Pinzani

Artemisia verlotiorum Lamotte
H scap/G rhiz - Orig. E-Asia
Literature data: Caruel 1860; Montelucci 1934; Montelucci 1962; Pedullà & Garbari, 2004
Field observations: partecipanti all'escursione Wikiplantbase #Parco di Migliarino - San Rossore - Massaciuccoli, 2018; L. Peruzzi, 2019; L. Pinzani, 2020, 2021, 2022; I. Arduini, 2023

Artemisia vulgaris L.
H scap - Circumbor.
Field observations: partecipanti all'escursione Wikiplantbase #Parco di Migliarino - San Rossore - Massaciuccoli, 2018; L. Pinzani, 2020

NC **Asteriscus aquaticus** (L.) Less.
T scap - Stenomedit.
Literature data: Baroni 1897-1908

Baccharis halimifolia L.
NP - Orig. N-America
Literature data: Coaro 1987; Dell'Orso & Franchini 2009; Saggese 2016; Lazzeri 2021, 2022
Herbarium data: L. Pinzani, 2020, Herb. Pinzani
Field observations: partecipanti all'escursione Wikiplantbase #Parco di Migliarino - San Rossore - Massaciuccoli, 2018

Bellis annua L. subsp. **annua**
T scap - Stenomedit.-Macarones.
Literature data: Garbari 2001

Bellis perennis L.
H ros - Circumbor.
Literature data: Caruel 1860; Corti 1956 [*S. Sommier*, s.d., FI]; Corti 1956; Coaro 1987; Garbari 2001; Arduini & Ercoli 2012; Bonari et al. 2019
Field observations: B. Pierini, 2014; L. Peruzzi, 2015; partecipanti all'escursione Wikiplantbase #Parco di Migliarino - San Rossore - Massaciuccoli, 2018; M. D'Antraccoli, 2019; F. Roma-Marzio, 2019; L. Pinzani, 2021, 2022; F. Roma-Marzio, 2021; I. Arduini, 2023

Bidens frondosa L.
T scap - Orig. N-America
Literature data: Caruel 1860; Tomei et al. 2004

NC **Bidens tripartita** L. subsp. **tripartita**
T scap - Eurasiat.
Literature data: Caruel 1860; Baroni 1897-1908

Bidens vulgata Greene
T scap - Orig. N-America
Literature data: Galasso et al. 2016 [*B. Pierini*, *L. Peruzzi*, 2015, PI]
Field observations: L. Pinzani

Calendula arvensis (Vaill.) L.
T scap/H bienn - Eurimedit.
Literature data: Caruel 1860
Field observations: L. Pinzani, 2021, 2022

NC **Carduus nutans** L. subsp. **nutans**
H bienn - W-Europ.
Literature data: Caruel 1860

Carduus pycnocephalus L. subsp. **pycnocephalus**
H bienn - Eurimedit.-Turan.

Literature data: Caruel 1860; Corti, 1951, 1956; Coaro 1987; Garbari 2001; Pedullà & Garbari, 2004; Lazzeri 2021
Herbarium data: J. Franzoni, 2019, Herb. Franzoni
Field observations: B. Pierini 2015; partecipanti all'escursione Wikiplantbase #Parco di Migliarino - San Rossore - Massaciuccoli, 2018; L. Pinzani, 2021, 2022; I. Arduini, 2024

Carduus tenuiflorus Curtis
H bienn/H scap - Subatl.-W-Europ.
Literature data: Arrigoni 2020

NC **Carlina corymbosa** L.
H scap - Stenomedit.
Literature data: Caruel 1860; Corti 1956, under the name *C. corymbosa* L. var. *genuina* Boiss.

NC **Carlina lanata** L.
T scap - Stenomedit.
Literature data: Caruel 1860

Carthamus lanatus L.
T scap - Eurimedit.
Literature data: Caruel 1860, under the name *Carduncellus lanatus* Mor.; Lombardi 2015
Field observations: B. Pierini, 2014

Centaurea aplolepa Moretti subsp. **subciliata** (DC.) Arcang.
H scap - Endem. Ital.
Literature data: Caruel 1860, Baroni 1897-1908, under the name *C. aplolepis* Morett.; Corti 1956 [*E. Levier*, s.d., FI; *s. coll.*, s.d., FI]; Arrigoni 2003 [*P. Savi*, 1841, FI: *s. coll.*, 1847, FI, Herb. Beccari; *Amidei*, 1868, FI; *Sommier*, 1871, FI; *Roberti*, 1876. FI; *Biondi*, 1886, FI; *Savelli*, 1913, FI; *Chiarugi*, 1934, FI]; Macchia & Bedini 2014 [*U. Macchia*, 2013, PI]
Herbarium data: L. Pinzani, 2019, Herb. Pinzani
Assessed as EN in the Red List of the Italian Vascular Flora (Rossi et al. 2013)

NC **Centaurea aspera** L. subsp. **aspera**
H scap - Stenomedit.-N-Occid.
Literature data: Caruel 1860

NC **Centaurea calcitrapa** L.
H bienn - Eurimedit.-Subcosmop.
Literature data: Caruel 1860; Baroni 1897-1908

NC **Centaurea cyanus** L.
T scap - Stenomedit.-Subcosmop.
Literature data: Caruel 1860

Centaurea jacea L. subsp. **gaudinii** (Boiss. & Reut.) Gremli
H scap - SE-Europ.
Literature data: Garbari 2001, under the name *C. bracteata* Scop.

Centaurea nigrescens Willd. subsp. **nigrescens**
H scap - Europ.
Literature data: Caruel 1860, Baroni 1897-1908, under the name *C. transalpina* Schleich.; Pedullà & Garbari, 2004; Arrigoni 2020, under the name *C. nigrescens* Willd. subsp. *vochinensis* (W.D.J.Koch) Nyman; Lazzeri 2021
Field observations: L. Pinzani, 2020, 2021

NC **Centaurea solstitialis** L. subsp. **solstitialis**
H bienn - Subcosmop.
Literature data: Caruel 1860

Centaurea sphaerocephala L. subsp. **sphaerocephala**
H scap - Stenomedit.-Occid.
Herbarium data: U. Macchia, G. Bedini, 2015, PI

Chondrilla juncea L.
H scap - Eurimedit.-S-Siber.
Literature data: Caruel 1860
Field observations: L. Peruzzi, 2019; L. Pinzani, 2022

Cichorium intybus L.
H scap - Cosmop.
Literature data: Caruel 1860; Coaro 1987; Tomei et al. 2004; Saggese 2016; Lazzeri 2021
Field observations: B. Pierini, 2014; partecipanti all'escursione Wikiplantbase #Parco di Migliarino - San Rossore - Massaciuccoli, 2018; L. Peruzzi, 2019; L. Pinzani, 2020, 2021, 2022; F. Roma-Marzio, 2021

Cirsium arvense (L.) Scop.
G rad - Eurasiat.
Literature data: Caruel 1860; Corti 1956; Coaro 1987; Garbari 2001; Saggese 2016; Lazzeri in Buono et al. 2022
Field observations: B. Pierini, 2017; partecipanti all'escursione Wikiplantbase #Parco di Migliarino - San Rossore - Massaciuccoli, 2018; I. Arduini, 2024

NC **Cirsium oleraceum** (L.) Scop.
H scap - Eurosiber.
Literature data: Caruel 1860

NC **Cirsium palustre** (L.) Scop.
H bienn - Paleotemp.
Literature data: Caruel 1860

Cirsium vulgare (Savi) Ten.
H bienn - Subcosmop.
Literature data: Caruel 1860, under the name *C. lanceolatum* Scop.; Garbari 2001; Pedullà & Garbari, 2004; Lombardi 2015; Saggese 2016; Lazzeri in Buono et al. 2022
Herbarium data: G. Cela, 1966, PI
Field observations: L. Pinzani, 2021, 2022

Cladanthus mixtus (L.) Chevall.
T scap - Stenomedit.-Occid.
Literature data: Caruel 1860, Corti 1956 [*A. Fiori*, s.d., FI]; Corti 1956, under the name *Anthemis mixta* L.; Lazzeri in Buono et al. 2022
Herbarium data: L. Pinzani, 2020, Herb. Pinzani

Coleostephus myconis (L.) Cass. ex Rchb.f.
T scap - Stenomedit.
Literature data: Caruel 1860, under the name *Chrysanthemum myconis* L.; Coaro 1987; Pedullà & Garbari, 2004, under the name *C. clausonis* Pomel; Bertacchi et al. 2009
Herbarium data: G. Deandrea, 1994, Herb. Picco
Field observations: partecipanti all'escursione Wikiplantbase #Parco di Migliarino - San Rossore - Massaciuccoli, 2018; G. Bedini, 2020; L. Pinzani, 2021, 2022; H. Öhm, 2024

Cota altissima (L.) J.Gay
T scap - S-Europ.-W-Asiat.
Literature data: Caruel 1860, under the name *Anthemis cota* L.; Pedullà & Garbari, 2004, under the name *Anthemis altissima* L.

Cota tinctoria (L.) J.Gay subsp. **tinctoria**
H bienn - C-Europ.
Literature data: Caruel 1860, under the name *Anthemis tinctoria* L.
Herbarium data: Partecipanti all'escursione Wikiplantbase #Parco di Migliarino - San Rossore - Massaciuccoli, 2018, PI

Crepis bellidifolia Loisel.
H scap/T scap - Stenomedit.-Occid.
Literature data: Baroni 1897-1908; Garbari 2001; Tomei et al. 2004; Saggese 2016
Assessed as LC in the Red List of the Italian Vascular Flora (Rossi et al. 2020)

Crepis bursifolia L.
H scap - C-Medit.
Herbarium data: F. Roma-Marzio, L. Peruzzi, 2019, PI
Field observations: L. Peruzzi, 2018; M. D’Antraccoli, 2019; L. Pinzani, 2022; H. Öhm, 2024

Crepis capillaris (L.) Wallr.
T scap - C-Europ.-Subatl.
Literature data: Garbari 2001

Crepis dioscoridis L.
T scap - Orig. S-Europ.
Literature data: Orlandi & Arduini 2010; Arduini & Ercoli 2012

NC **Crepis foetida** L. subsp. **foetida**
T scap/H bienn - Eurimedit.
Literature data: Caruel 1860; Corti 1956 [*P. Savi*, s.d., FI]

Crepis leontodontoides All.
H ros/H scap - W-Medit.

Literature data: Caruel 1860; Corti, 1951, 1956; Garbari 2001; Arduini & Ercoli 2012

NC **Crepis neglecta** L. subsp. **neglecta**
T scap - Eurimedit.-N-Orient.
Literature data: Caruel 1860

Crepis pulchra L. subsp. **pulchra**
T scap - Eurimedit.
Literature data: Caruel 1860
Field observations: B. Pierini, 2015, 2020; L. Pinzani, 2022

Crepis sancta (L.) Bornm. subsp. nemausensis (P.Fourn.) Babc.
T scap - Orig. Eurimedit.
Literature data: Corti 1956, under the name *Lagoseris sancta* (L.) K.Maly subsp. *nemausensis* (Gouan) Thell.; Coaro 1987, Garbari 2001, Lombardi 2015, under the name *C. sancta* (L.) Babc. subsp. *sancta*; Arduini & Ercoli 2012, under the name *C. sancta* (L.) Babc.
Field observations: B. Pierini, 2014; L. Pinzani, 2021, 2022

Crepis setosa Haller f.
T scap - Eurimedit.-Orient.
Literature data: Caruel 1860; Corti 1956, under the name *C. setosa* Hall. f. subsp. *typica* Babc.; Coaro 1987; Garbari 2001; Arduini & Ercoli 2012
Herbarium data: Partecipanti all'escursione Wikiplantbase #Parco di Migliarino - San Rossore - Massaciuccoli, 2018, PI

Crepis suffreniana (DC.) Steud.
T scap - Europ.
Literature data: Baroni 1897-1908; Corti 1956 [*Roberti* (ex Herb. Levier), 1876, FI; *A. Fiori*, 1881, FI; *A. Fiori*, 1910, FI; *P. Savi*, s.d., FI; *A. Chiarugi, R. Corti*, 1951, FI]; Corti 1956, 1970; Garbari 2001; Tomei et al. 2004; Arduini & Ercoli 2012
Herbarium data: Della Nave (ex Herb. Pichi-Sermolli), 1881, FI; O. Beccari, 1862, PI
Assessed as LC in the Red List of the Italian Vascular Flora (Rossi et al. 2020)

Crepis vesicaria L.
T scap/H bienn - Submedit.-Subatl.
Literature data: Caruel 1860; Corti 1956; Coaro 1987; Garbari 2001; Bertacchi et al. 2009; Bertacchi et al. 2010; Bertacchi & Lombardi 2014a
Field observations: partecipanti all'escursione Wikiplantbase #Parco di Migliarino - San Rossore - Massaciuccoli, 2018; L. Pinzani, 2021, 2022; H. Öhm, 2024

NC **Cynara cardunculus** L. subsp. **cardunculus**
H scap - Stenomedit.
Literature data: Caruel 1860

Dimorphotheca ecklonis DC.
Field observations: L. Pinzani, 2021

Dittrichia graveolens (L.) Greuter
T scap - Medit.-Turan.

Literature data: Caruel 1860, under the name *I. graveolens* (L.) Desf.; Orlandi & Arduini 2010; Arduini & Ercoli 2012; Lazzeri in Buono et al. 2022
Field observations: L. Peruzzi, 2019; I. Arduini, 2024

Dittrichia viscosa (L.) Greuter subsp. **viscosa**
H scap - Eurimedit.
Literature data: Caruel 1860, Corti 1956, Coaro 1987, Tomei et al. 2004, Petraglia 2013 Bertacchi & Lombardi 2014b, under the name *I. viscosa* (L.) Ait.; Garbari 2001; Sani & Tomei 2006; Arduini & Ercoli 2012; Saggese 2016; Lazzeri 2021, 2022
Herbarium data: F. Picco, 1995, Herb. Picco
Field observations: partecipanti all'escursione Wikiplantbase #Parco di Migliarino - San Rossore - Massaciuccoli, 2018; L. Peruzzi, 2019; L. Pinzani, 2020, 2021, 2022; I. Arduini, 2023; H. Öhm, 2024

Eclipta prostrata (L.) L.
T scap - Orig. America
Literature data: Peruzzi et al. 2007 [*B. Pierini*, 2007, FI, PI; *J.-M. Tison*, 2007, Erb. Tison]

Epitrachys italica (DC.) Bureš, Del Guacchio, Iamonico & P.Caputo
H bienn - SE-Europ.
Literature data: Caruel 1860, under the name *Cirsium italicum* DC.
Field observations: I. Arduini, 2024

Erigeron acris L. subsp. **acris**
H scap/H bienn - Circumbor.
Literature data: Baroni 1897-1908; Corti 1956 [*S. Sommier*, s.d., FI]; Garbari 2001

NC *Erigeron annuus* (L.) Desf. subsp. *annuus*
Literature data: Baroni 1897-1908, under the name *Stenactis bellidiflora* Al.Braun

Erigeron bonariensis L.
T scap - Orig. America
Literature data: Caruel 1860, under the name *Conyza ambigua* Dec.
Herbarium data: F. Roma-Marzio, L. Peruzzi, 2019, PI

Erigeron canadensis L.
T scap - Orig. America
Literature data: Caruel 1860; Baroni 1897-1908; Corti 1956 [*S. Sommier*, s.d., FI]; Corti 1956; Gellini et al. 1986; Coaro 1987, Garbari 2001, Tomei et al. 2004, Bertacchi et al. 2009, under the name *Conyza canadensis* (L.) Cronq.; Arduini & Ercoli 2012; Lombardi 2015; Saggese 2016; Lazzeri in Buono et al. 2022
Herbarium data: M.L. Pedullà, 1999, PI
Field observations: L. Peruzzi, 2019; I. Arduini, 2023

Erigeron karvinskianus DC.
H scap - Orig. Subtrop.
Literature data: Barsali 1909; Savelli 1915; Corti 1956, under the name *E. karwinskyanus* DC. var. *mucronatus* (DC) Aschers.; Garbari 2001; Pedullà & Garbari, 2004
Herbarium data: F. Roma-Marzio, L. Peruzzi, 2019, PI

Field observations: L. Pinzani, 2021

Erigeron sumatrensis Retz.
T scap - Orig. America
Literature data: Orlandi & Arduini 2010; Arduini & Ercoli 2012; Lazzeri in Buono et al. 2022
Field observations: L. Pinzani, 2020; I. Arduini, 2024

Eupatorium cannabinum L. subsp. **cannabinum**
H scap - Paleotemp.
Literature data: Corti 1956; Coaro 1987; Garbari 2001
Field observations: B. Pierini, 2014

Filago germanica (L.) Huds.
T scap - Paleotemp.
Literature data: Corti 1956; Coaro 1987, under the name *Filago vulgaris* Lam.; Lombardi 2015
Herbarium data: Partecipanti all'escursione Wikiplantbase #Parco di Migliarino - San Rossore - Massaciuccoli, 2018, PI

NC **Filago pyramidata** L.
T scap - Eurimedit.
Literature data: Corti 1956, under the name *F. germanica* L. subsp. *spathulata* (Presl.) Rouy

NC **Galactites tomentosus** Moench
H bienn - Medit.
Literature data: Caruel 1860

NC **Galatella linosyris** (L.) Rchb.f. subsp. **linosyris**
H scap - Eurimedit.-S-Siber.
Literature data: Caruel 1860, under the name *Linosyris vulgaris* Dec. β *palustris*

Galatella tripolium (L.) Galasso, Bartolucci & Ardenghi subsp. **pannonica** (Jacq.) Galasso, Bartolucci & Ardenghi
H bienn - Eurasiat.
Literature data: Caruel 1860, under the name *Aster tripolium* L.; Corti 1956 [*S. Sommier*, s.d., FI]; Corti 1956, under the name *Aster Tripolium* L. var. *glaber* Bolzon; Coaro 1987, under the name *Aster tripolium* L., *Aster tripolium* L. subsp. *pannonicus*; Garbari 2001, Tomei et al. 2004, Bertacchi et al. 2007, Bertacchi et al. 2009, Petraglia 2013, Bertacchi & Lombardi 2014b, under the name *Aster tripolium* L.; Saggese 2016, under the name *Tripolium pannonicum* (Jacq.) Dobrocz. subsp. *pannonicum*; Lazzeri 2021, 2022
Field observations: A. Giacò, J. Franzoni, L. Pinzani, 2021

NC *Galinsoga parviflora* Cav.
Literature data: Baroni 1897-1908; Savelli 1915

Galinsoga quadriradiata Ruiz & Pav.
T scap - Orig. S-America
Herbarium data: L. Pinzani, 2020, Herb. Pinzani

Gamochaeta americana (Mill.) Wedd.

H scap - Orig. S-America
Field observations: B. Pierini, 2018

Gazania linearis (Thunb.) Druce
Field observations: J. Franzoni, 2021

NC **Geropogon hybridus** (L.) Sch.Bip.
T scap - Stenomedit.
Literature data: Caruel 1860, under the name *G. glaber* L.

Glebionis segetum (L.) Fourr.
T scap - Eurimedit.
Literature data: Caruel 1860, Bertacchi et al. 2009, under the name *Chrysanthemum segetum* L.
Herbarium data: L. Pinzani, 2020, Herb. Pinzani
Field observations: L. Pinzani, 2021

Gnaphalium uliginosum L.
T scap - Eurosiber.
Literature data: Caruel 1860; Garbari 2001; Tomei et al. 2004; Lombardi 2015

Hedypnois rhagadioloides (L.) F.W.Schmidt
T scap - Stenomedit.
Literature data: Caruel 1860, under the name *H. polymorpha* DC.; Corti 1956, under the name *H. rhagadioloides* (L.) Willd. subsp. *cretica* (L.) Hayek; Garbari 2001; Lombardi 2015
Herbarium data: L. Pinzani, 2021, Herb. Pinzani
Field observations: L. Pinzani, 2022

Helianthus annuus L. subsp. *annuus*
Field observations: L. Pinzani, 2021

Helianthus tuberosus L.
G bulb - Orig. N-America
Field observations: G. Pacifico, 2022

Helichrysum italicum (Roth) G.Don subsp. **italicum**
Ch suffr - S-Europ.
Literature data: Caruel 1860, under the name *H. angustifolium* Guss.; Corti, 1951; Arduini & Ercoli 2012
Herbarium data: F. Roma-Marzio, M. D’Antraccoli, 2017, PI; L. Pinzani, 2020, Herb. Pinzani
Field observations: L. Pinzani, 2022

NC **Helichrysum luteoalbum** (L.) Rchb.
T scap - Subcosmop.
Literature data: Caruel 1860, under the name *Gnaphalium luteo-album* L.

Helichrysum stoechas (L.) Moench subsp. **stoechas**
Ch suffr - W-Medit.

Literature data: Caruel 1860; Corti 1956 [*Picciuoli*, FI]; Corti 1956, under the name *H. stoechas* (L.) Moench var. *typicum* Fiori; Garbari 2001; Tomei et al. 2004; Sani & Tomei 2006; Bertacchi et al. 2009; Bertacchi et al. 2010; Bertacchi & Lombardi 2014a
Herbarium data: D. Ciccarelli, 2011, PI
Field observations: R. Righini, 2021

Helminthotheca echioides (L.) Holub
T scap - Eurimedit.
Literature data: Caruel 1860, under the name *Helminthia echioides* Gaertn.; Corti 1956, 1970, under the name *Picris echioides* L. var. *tuberculata* Fiori; Coaro 1987, Garbari 2001, Petraglia 2013, under the name *Picris echioides* L.; Saggese 2016; Lazzeri in Buono et al. 2022
Herbarium data: Partecipanti all'escursione Wikiplantbase #Parco di Migliarino - San Rossore - Massaciuccoli, 2018, PI
Field observations: partecipanti all'escursione Wikiplantbase #Parco di Migliarino - San Rossore - Massaciuccoli, 2018; M. D'Antraccoli, 2019; L. Peruzzi, 2019; L. Pinzani, 2020, 2021

NC **Hieracium bifidum** Kit. ex Hornem.
H ros/H scap - Orof. Europ.
Literature data: Corti 1956, under the name *H. bifidum* Kit. gruppo *eu-bifidum* Zahn

NC **Hieracium murorum** L.
H scap - Europ.
Literature data: Caruel 1860

Hyoseris radiata L.
H ros - Stenomedit.
Field observations: L. Pinzani, 2021, 2022

Hypochaeris achyrophorus L.
T scap - Stenomedit.
Literature data: Bonari et al. 2019

Hypochaeris glabra L.
T scap - Eurimedit.
Literature data: Caruel 1860; Corti 1956 [*Roberti*, FI]; Corti, 1954, 1956, under the name *H. glabra* L. var. t*ypica* Fiori; Garbari 2001; Arduini & Ercoli 2012; Lombardi 2015; Bonari et al. 2019
Herbarium data: L. Pinzani, 2022, Herb. Pinzani

Hypochaeris radicata L.
H ros - Europ.-Caucas.
Literature data: Caruel 1860; Garbari 2001; Tomei et al. 2004; Bertacchi et al. 2009; Bertacchi et al. 2010; Arduini & Ercoli 2012; Bertacchi & Lombardi 2014a; Bonari et al. 2019; Lazzeri in Buono et al. 2022
Field observations: partecipanti all'escursione Wikiplantbase #Parco di Migliarino - San Rossore - Massaciuccoli, 2018; L. Pinzani, 2021, 2022

NC *Inula helenium* L. subsp. *helenium*
Literature data: Caruel 1860

Jacobaea erratica (Bertol.) Fourr.
H bienn - Centroeurop.
Literature data: Caruel 1860, Coaro 1987, under the name *Senecio erraticus* Bert.
Herbarium data: F. Picco, 1995, Herb. Picco; L. Pinzani, 2020, Herb. Pinzani
Field observations: partecipanti all'escursione Wikiplantbase #Parco di Migliarino - San Rossore - Massaciuccoli, 2018

NC **Jacobaea erucifolia** (L.) G.Gaertn., B.Mey. & Scherb. subsp. **erucifolia**
H scap - Eurasiat.
Literature data: Caruel 1860, under the name *Senecio erucifolius* L.

Jacobaea maritima (L.) Pelser & Meijden subsp. **maritima**
Ch suffr - Stenomedit.-W-Medit.
Literature data: Sani & Tomei 2005 [*A. Sani, P.E. Tomei*, 2005, FI]
Herbarium data: D. Ciccarelli, 2012, PI

Lactuca perennis L.
H scap - Eurimedit.-Occid.
Literature data: Garbari 2001

Lactuca saligna L.
T scap/H bienn - Eurimedit.-Turan.
Literature data: Caruel 1860; Coaro 1987; Orlandi & Arduini 2010; Arduini & Ercoli 2012
Herbarium data: L. Pinzani, 2020, Herb. Pinzani
Field observations: L. Pinzani, 2021

Lactuca sativa L. subsp. **serriola** (L.) Galasso, Banfi, Bartolucci & Ardenghi
H bienn/T scap - Eurimedit.-S-Siber.
Literature data: Caruel 1860, under the name *L. scariola* L.
Field observations: B. Pierini, 2014; partecipanti all'escursione Wikiplantbase #Parco di Migliarino - San Rossore - Massaciuccoli, 2018; L. Peruzzi, 2019; L. Pinzani, 2022

Lactuca virosa L.
T scap/H bienn - Medit.-Atl.
Field observations: L. Peruzzi, 2018

Lapsana communis L. subsp. **communis**
T scap - Paleotemp.
Literature data: Caruel 1860, under the name *Lampsana communis* L.; Corti 1956, under the name *L. communis* L. var. *typica* Fiori in Fiori e Paoletti; Coaro 1987; Garbari 2001; Pedullà & Garbari, 2004
Field observations: partecipanti all'escursione Wikiplantbase #Parco di Migliarino - San Rossore - Massaciuccoli, 2018

Leontodon hispidus L. subsp. **hispidus**
H ros - Europ.-Caucas.
Literature data: Garbari 2001

Leontodon rosanoi (Ten.) DC.

H ros - Eurimedit.
Literature data: Caruel 1860, Sani & Tomei 2006, under the name *L. villarsii* Lois.; Arduini & Ercoli 2012, under the name *L. leysserii* (Wallr.) Beck
Herbarium data: A. Sani, 2005, PI

Leontodon saxatilis Lam. subsp. **saxatilis**
T scap/H scap - Medit.-Mont.
Literature data: Caruel 1860, under the name *Thrincia hirta* Roth; Sani & Tomei 2006, under the name *L. taraxacoides* (Vill.) Mérat

Leontodon tuberosus L.
H ros - Stenomedit.
Literature data: Caruel 1860, under the name *Thrincia tuberosa* DC.
Field observations: A. Mo, I. Pucci, S. Pascacaldi, L. Pinzani, 2022

Leucanthemum ircutianum DC. subsp. **ircutianum**
H scap - Eurosiber.
Literature data: Garbari 2001, under the name *L. vulgare* Lam.

Leucanthemum ircutianum DC. subsp. **leucolepis** (Briq. & Cavill.) Vogt & Greuter
H scap - Eurimedit.
Literature data: Corti 1956, under the name *Chrysanthemum leuchanthemum* L. subsp. *triviale* Gaud. forma *pallidum* Fiori in Fiori e Paoletti; Garbari 2001, under the name *Leucanthemum pallens* (J.Gay ex Perreym.) DC.
Herbarium data: L. Pinzani, 2020, Herb. Pinzani

Limbarda crithmoides (L.) Dumort. subsp. **longifolia** (Arcang.) Greuter
Ch suffr - Medit.-Atl.
Literature data: Caruel 1860, Corti 1956 [*S. Sommier*, s.d., FI], Coaro 1987, Tomei et al. 2004, Sani & Tomei 2006, Bertacchi et al. 2007, Bertacchi et al. 2009, Petraglia 2013, Bertacchi & Lombardi 2014b, under the name *Inula crithmoides* L.; Saggese 2016
Herbarium data: D. Ciccarelli, 2011, PI
Field observations: partecipanti all'escursione Wikiplantbase #Parco di Migliarino - San Rossore - Massaciuccoli, 2018; A. Mo, I. Pucci, S. Pastacaldi, L. Pinzani, 2022

Logfia gallica (L.) Cosson & Germ.
T scap - Eurimedit.
Field observations: L. Pinzani, 2022

Logfia minima (Sm.) Dumort.
T scap - Centroeurop.-S-Siber.
Literature data: Lombardi 2015, under the name *Filago minima* (Sm.) Pers.; Peruzzi et al. 2017b [*A. Sani, M. D'Antraccoli*, 2014, PI], under the name *Filago minima* (Sm.) Pers.

Matricaria chamomilla L.
T scap - Subcosmop.
Literature data: Caruel 1860; Pedullà & Garbari, 2004
Field observations: partecipanti all'escursione Wikiplantbase #Parco di Migliarino - San Rossore - Massaciuccoli, 2018; L. Pinzani, 2021

NC **Onopordum acanthium** L. subsp. **acanthium**
H bienn - Eurasiat.-Medit.-Turan.
Literature data: Caruel 1860

NC **Pallenis spinosa** (L.) Cass. subsp. **spinosa**
T scap/H bienn - Eurimedit.
Literature data: Caruel 1860, under the name *Asteriscus spinosus* Gren. et Godr.

NC **Pentanema britannicum** (L.) D.Gut.Larr., Santos-Vicente, Anderb., E.Rico & M.M.Mart.Ort.
H scap - Medioeurop.-W-Asiat.
Literature data: Caruel 1860, under the name *Inula britannica* L.

Pentanema salicinum (L.) D.Gut.Larr., Santos-Vicente, Anderb., E.Rico & M.M.Mart.Ort.
H scap - Europ.-Caucas.
Literature data: Coaro 1987, under the name *Inula salicina* L.

Pentanema squarrosum (L.) D.Gut.Larr., Santos-Vicente, Anderb., E.Rico & M.M.Mart.Ort.
H bienn - Eurasiat.
Literature data: Caruel 1860, Corti 1956, Garbari 2001, under the name *Inula conyza* DC.

NC **Petasites hybridus** (L.) G.Gaertn., B.Mey. & Scherb. subsp. **hybridus**
G rhiz - Eurasiat.
Literature data: Caruel 1860, under the name *P. officinalis* Moench

Phagnalon sordidum (L.) Rchb.
Ch suffr - W-Medit.
Literature data: Caruel 1860
Herbarium data: F. Roma-Marzio, L. Peruzzi, 2019, PI
Field observations: B. Pierini, 2015; M. D'Antraccoli, 2019; F. Roma-Marzio, 2021; L. Pinzani, 2022

NC **Picnomon acarna** (L.) Cass.
T scap - Stenomedit.
Literature data: Caruel 1860, under the name *Cirsium acarna* (L.) Moench

Picris hieracioides L. subsp. **hieracioides**
H scap/H bienn - Eurosiber.
Literature data: Caruel 1860; Coaro 1987; Garbari 2001; Pedullà & Garbari, 2004; Lazzeri in Buono et al. 2022
Field observations: partecipanti all'escursione Wikiplantbase #Parco di Migliarino - San Rossore - Massaciuccoli, 2018; L. Peruzzi, 2019; L. Pinzani, 2020, 2021, 2022

Pilosella officinarum F.W.Schultz & Sch.Bip.
H ros - Europ.-Caucas. (Subatl.)
Literature data: Tomei et al. 2004, under the name *Hieracium pilosella* L.

Pilosella piloselloides (Vill.) Soják subsp. **piloselloides**

H scap - Europ.
Literature data: Corti 1956 [*Picciuoli*, FI]; Corti, 1954, 1956, under the name *Hieracium piloselloides* Vill. gruppo *florentinum* (All.) Zahn; Garbari 2001, Arduini & Ercoli 2012, under the name *Hieracium piloselloides* Vill.
Field observations: L. Pinzani, 2022

NC **Pilosella piloselloides** (Vill.) Soják subsp. **praealta** (Vill. ex Gochnat) S.Bräut. & Greuter
H scap - Europ.
Literature data: Caruel 1860, under the name *Hieracium praealtum* Vill. ex Gochnat

Pulicaria dysenterica (L.) Bernh.
H scap - Eurimedit.
Literature data: Caruel 1860; Baroni 1897-1908; Fiori 1943, under the name *P. dysenterica* (L.) Bernh. var. *ramosissima* Lec. et Lam; Coaro 1987; Garbari 2001; Pedullà & Garbari, 2004; Petraglia 2013; Saggese 2016; Lazzeri in Buono et al. 2022
Field observations: partecipanti all'escursione Wikiplantbase #Parco di Migliarino - San Rossore - Massaciuccoli, 2018; L. Pinzani, 2020; H. Öhm, 2024

Pulicaria odora (L.) Rchb.
H scap - Eurimedit.
Field observations: L. Peruzzi, 2019

Pulicaria sicula (L.) Moris
T scap - Stenomedit.
Literature data: Caruel 1860; Baroni 1897-1908; Corti 1956 [*S. Sommier*, s.d., FI; *Roberti*, s.d., FI]; Garbari 2001

NC **Pulicaria vulgaris** Gaertn.
T scap - Paleotemp.
Literature data: Caruel 1860

Reichardia picroides (L.) Roth
H scap - Stenomedit.
Literature data: Corti 1956, under the name *R. picroides* (L.) Roth var. *vulgaris* (Desf.) O. Ktze forma *pinnatifida* Fiori in Fiori e Paoletti; Garbari 2001; Bonari et al. 2019; Lazzeri in Buono et al. 2022
Herbarium data: D. Ciccarelli, 2012, PI; F. Roma-Marzio, L. Peruzzi, 2019, PI
Field observations: partecipanti all'escursione Wikiplantbase #Parco di Migliarino - San Rossore - Massaciuccoli, 2018; L. Peruzzi, 2019; B. Pierini, 2020; L. Pinzani, 2021, 2022

Scolymus hispanicus L. subsp. **hispanicus**
H bienn - Eurimedit.
Literature data: Caruel 1860; Corti 1956 [*P. Fantozzi*, s.d., FI]; Corti 1956; Coaro 1987; Garbari 2001; Pedullà & Garbari, 2004
Field observations: B. Pierini, 2014; L. Pinzani, 2019

Scolymus hispanicus L. subsp. **occidentalis** F.M.Vázquez
H bienn - W-Medit.
Literature data: Lazzeri in Buono et al. 2022

Scolymus maculatus L.
T scap - S-Medit.
Literature data: Caruel 1860
Herbarium data: B. Pierini, 2024, PI

NC **Scorzonera laciniata** L. subsp. l**aciniata**
H bienn/T scap - Paleotemp.
Literature data: Caruel 1860, under the name *Podospermum laciniatum* (L.) DC.

Senecio angulatus L.f.
Field observations: observed on iNaturalist in 2024

Senecio inaequidens DC.
T scap/Ch suffr - Orig. S-Africa
Field observations: L. Pinzani, 2021

Senecio lividus L.
T scap - Stenomedit.
Literature data: Corti 1956; Garbari 2001

Senecio sylvaticus L.
T scap - Europ.
Literature data: Orlandi & Arduini 2010; Arduini & Ercoli 2012

Senecio vulgaris L. subsp. **vulgaris**
T scap - Eurimedit.
Literature data: Caruel 1860; Corti 1956; Garbari 2001; Pedullà & Garbari, 2004; Arduini & Ercoli 2012
Field observations: B. Pierini, 2014; L. Peruzzi, 2017; L. Pinzani, 2021, 2022; I. Arduini, 2024

NC **Serratula tinctoria** L. subsp. **tinctoria**
H scap - Eurosiber.
Literature data: Caruel 1860

Silybum marianum (L.) Gaertn.
H bienn - Medit.-Turan.
Literature data: Caruel 1860
Field observations: B. Pierini, 2014; L. Pinzani, 2022

NC *Solidago gigantea* Aiton
Literature data: Baroni 1897-1908, under the name *S. serotina* Ait.

Solidago virgaurea L. subsp**. litoralis** (Savi) Briq. & Cavill.
H scap - Endem. Ital.
Literature data: Caruel 1860, under the name *S. virgaurea* L. γ *litoralis* Dec.; Corti 1956 [*S. Sommier*, s.d., FI]; Corti, 1970; Garbari & Cecchi 2000 [*G. Savi*, 1806, BOLO; *s. coll.*, 1846, TO; *P. Savi*, 1857, PI; *Beccari*, 1866, FI; *Passerini*, 1905, PI; *Savelli*, 1913, FI]; Bertacchi et al. 2009,

Bertacchi et al. 2010, Bertacchi & Lombardi 2014a, Bertacchi & Lombardi 2014b, under the name *Solidago litoralis* Savi
Field observations: A. Mo, I. Pucci, S. Pascacaldi, L. Pinzani, 2022
Assessed as EN in the Red List of the Italian Vascular Flora (Rossi et al. 2013)

Sonchus arvensis L. subsp. **arvensis**
H scap - Eurosiber.
Literature data: Tomei et al. 2004; Orlandi & Arduini 2010; Arduini & Ercoli 2012
Field observations: F. Roma-Marzio, 2021

Sonchus asper (L.) Hill subsp. **asper**
T scap - Eurasiat.
Literature data: Corti 1956, under the name *S. asper (*L.) Hill. subsp. *decipiens* (DNtrs.) Zenari, subsp. *spinosus* (Lam. p. p.) Zenari, subsp. *runcinatus* (Fiori) Zenari forma *subintegrifolius* Zenari; Coaro 1987; Garbari 2001; Tomei et al. 2004; Sani & Tomei 2006; Bertacchi et al. 2009; Arduini & Ercoli 2012; Saggese 2016
Herbarium data: L. Pinzani, 2021, Herb. Pinzani
Field observations: partecipanti all'escursione Wikiplantbase #Parco di Migliarino - San Rossore - Massaciuccoli, 2018; M. D'Antraccoli, 2019; L. Pinzani, 2021, 2022; J. Franzoni, A. Giacò, 2023

Sonchus bulbosus (L.) N.Kilian & Greuter subsp. **bulbosus**
G rad - Medit.
Literature data: Caruel 1860, under the name *Crepis bulbosa* Froel.; Corti 1956, Garbari 2001, Bonari et al. 2019, under the name *Aetheorhiza bulbosa* (L.) Cass.
Field observations: L. Pinzani, 2022

Sonchus maritimus L.
H scap - Eurimedit.
Literature data: Caruel 1860; Corti 1956 [*S. Sommier*, s.d., FI; *M. Savelli*, s.d., FI]; Corti 1956; Coaro 1987; Garbari 2001; Arduini & Ercoli 2012

Sonchus oleraceus L.
T scap/H bienn - Subcosmop.
Literature data: Caruel 1860, under the name *S. laevis* Bart.; Coaro 1987; Pedullà & Garbari, 2004; Lombardi 2015
Field observations: L. Peruzzi, 2015, 2019; partecipanti all'escursione Wikiplantbase #Parco di Migliarino - San Rossore - Massaciuccoli, 2018; L. Pinzani, 2020, 2022; I. Arduini, 2024

NC **Sonchus tenerrimus** L.
T scap/H scap - Stenomedit.
Literature data: Caruel 1860, under the name *S. laevis* Bart.

Symphyotrichum squamatum (Spreng.) G.L.Nesom
T scap/H scap - Orig. America
Literature data: Coaro 1987, under the name *Aster squamatus* (Sprengel) Lojac.; Saggese 2016; Lazzeri 2021, 2022
Herbarium data: F. Roma-Marzio, L. Peruzzi, 2019, PI
Field observations: partecipanti all'escursione Wikiplantbase #Parco di Migliarino - San Rossore - Massaciuccoli, 2018; L. Peruzzi, 2019; L. Pinzani, 2020, 2021, 2022

NC *Symphyotrichum ×salignum* (Willd.) G.L.Nesom
Literature data: Baroni 1897-1908, under the name *Aster salignus* Willd.

Tanacetum parthenium (L.) Sch.Bip.
H scap - Eurasiat.
Literature data: Caruel 1860, under the name *Pyrethrum parthenium* (L.) Sm.
Field observations: I. Arduini, 2023

NC **Tanacetum vulgare** L. subsp. **vulgare**
H scap - Eurasiat.
Literature data: Caruel 1860

Taraxacum F.H.Wigg. sect. **Taraxacum**
H ros - Circumbor.
Literature data: Corti 1956, under the name *T. obovatum* (Willd.) DC.; Coaro 1987, Garbari 2001, Bonari et al. 2019, under the name *T. officinale* Weber
Field observations: L. Peruzzi, 2013; I. Arduini, 2023

Tolpis umbellata Bertol.
T scap - Stenomedit.
Literature data: Baroni 1897-1908; Peruzzi et al. 2022 [*G. Astuti, A. Cutroneo, F. Roma-Marzio, R. Vangelisti*, 2022, PI]

Tolpis virgata (Desf.) Bertol. subsp. **virgata**
H scap - Stenomedit.
Literature data: Tomei et al. 2004; Lombardi 2015
Field observations: partecipanti all'escursione Wikiplantbase #Parco di Migliarino - San Rossore - Massaciuccoli, 2018

Tragopogon dubius Scop.
H bienn - S-Europ.-Caucas.
Field observations: B. Pierini, 2014

NC **Tragopogon eriospermus** Ten.
H bienn - NE-Medit.
Literature data: Caruel 1860; Corti 1956 [*M. Savelli*, s.d., FI]; Corti 1956

Tragopogon porrifolius L.
H bienn/T scap - Eurimedit.
Literature data: Caruel 1860; Coaro 1987; Garbari 2001; Saggese 2016; Peruzzi et al. 2017b [*F. Roma-Marzio, A. Carta, M. D'Antraccoli*, 2017, PI]
Field observations: M. Mirabile, 2015; B. Pierini, 2017; partecipanti all'escursione Wikiplantbase #Parco di Migliarino - San Rossore - Massaciuccoli, 2018; L. Pinzani, 2021, 2022; J. Franzoni, A. Giacò, 2023; H. Öhm, 2024

Tragopogon pratensis L.
H scap - Eurosiber.
Literature data: Bertacchi et al. 2010; Bertacchi & Lombardi 2014a

Tussilago farfara L.
G rhiz - Paleotemp.
Literature data: Caruel 1860; Lazzeri in Buono et al. 2022

Urospermum dalechampii (L.) Scop. ex F.W.Schmidt
H scap - Eurimedit.-Centro-Occid.
Literature data: Caruel 1860; Coaro 1987; Bertacchi et al. 2009; Bertacchi et al. 2010; Bertacchi & Lombardi 2014a
Field observations: partecipanti all'escursione Wikiplantbase #Parco di Migliarino - San Rossore - Massaciuccoli, 2018; L. Pinzani, 2021, 2022

Urospermum picroides (L.) Scop. ex F.W.Schmidt
T scap - Eurimedit.
Literature data: Corti 1956, under the name *U. picroides* (L.) F. W. Schm. var. *picroides*; Garbari 2001
Herbarium data: L. Pinzani, 2021, Herb. Pinzani

Xanthium orientale L.
T scap - Orig. America
Literature data: Levier & Sommier 1891, Baroni 1897-1908, under the name *X. macrocarpum* DC.; Corti 1956 [*M. Savelli*, s.d., FI]; Garbari 2001, Tomei et al. 2004, Bertacchi et al. 2009, Petraglia 2013, under the name *X. italicum* Moretti
Field observations: partecipanti all'escursione Wikiplantbase #Parco di Migliarino - San Rossore - Massaciuccoli, 2018; L. Pinzani, 2020

Xanthium strumarium L.
T scap - Cosmop.
Literature data: Caruel 1860; Levier & Sommier 1891, under the name *X. macrocarpum* DC.; Corti 1956 [*M. Savelli*, s.d., FI]; Corti 1956; Garbari 2001

Viburnaceae

Sambucus ebulus L.
G rhiz/H scap - Eurimedit.
Literature data: Caruel 1860; Corti 1956; Garbari 2001
Field observations: B. Pierini, 2014; L. Pinzani, 2021, 2022

Sambucus nigra L.
P caesp - Europ.-Caucas.
Literature data: Corti 1956; Gellini et al. 1986; Coaro 1987; Tomei et al. 2004; Bertacchi & Lombardi 2016
Herbarium data: M.L. Pedullà, 2000, PI
Field observations: partecipanti all'escursione Wikiplantbase #Parco di Migliarino - San Rossore - Massaciuccoli, 2018; L. Pinzani, 2020, 2021, 2022; I. Arduini, 2023

NC **Viburnum opulus** L.
P caesp - Eurasiat.
Literature data: Caruel 1860

Viburnum tinus L. subsp. **tinus**
P caesp - Stenomedit.
Literature data: Tomei et al. 2004; Bertacchi et al. 2010; Bertacchi & Lombardi 2014a
Herbarium data:
Field observations: partecipanti all'escursione Wikiplantbase #Parco di Migliarino - San Rossore - Massaciuccoli, 2018; A. Mo, 2023

Caprifoliaceae

Abelia chinensis R.Br.
Field observations: observed on iNaturalist in 2023
Only cultivated

Centranthus ruber (L.) DC. subsp. **ruber**
Ch suffr - Stenomedit.
Literature data: Caruel 1860
Field observations: L. Pinzani, 2022

Cephalaria transsylvanica (L.) Roem. & Schult.
T scap - SE-Europ.-Caucas.
Literature data: Caruel 1860
Herbarium data: L. Pinzani, 2020, Herb. Pinzani
Field observations: B. Pierini, 2004; L. Pinzani, 2021, 2022

Dipsacus fullonum L. subsp. **fullonum**
H bienn - Eurimedit.
Literature data: Caruel 1860, under the name *D. sylvestris* Mill.; Corti 1956, under the name *D. silvester* Huds.; Coaro 1987; Saggese 2016
Field observations: partecipanti all'escursione Wikiplantbase #Parco di Migliarino - San Rossore - Massaciuccoli, 2018; L. Pinzani, 2020, 2021, 2022; I. Arduini, 2024; H. Öhm, 2024

Knautia arvensis (L.) Coult.
H bienn/H scap - Eurasiat.
Literature data: Caruel 1860; Bertacchi & Lombardi 2016

NC **Knautia integrifolia** (L.) Bertol. subsp. **integrifolia**
T scap - Eurimedit.
Literature data: Caruel 1860, under the name *K. hybrida* Coult.

Lomelosia rutifolia (Vahl) Avino & P.Caputo
H scap - Stenomedit.-Occid.
Literature data: Caruel 1860, under the name *Scabiosa rutaefolia* Vahl; Corti 1956 [*P. Savi*, s.d., FI]; Garbari 2001, under the name *Pycnocomon rutifolium* (Vahl) Hoffmanns. & Link

Lonicera caprifolium L.
P lian - SE-Europ.
Literature data: Caruel 1860; Gellini et al. 1986; Coaro 1987
Field observations: A. Giacò, J. Franzoni, L. Pinzani, 2021

Lonicera etrusca Santi
P lian - Eurimedit.
Literature data: Caruel 1860; Corti 1956 [*Corinaldi*, s.d., FI]; Corti 1956, 1970; Bertacchi et al. 2010; Bertacchi & Lombardi 2014a
Herbarium data: C. Del Prete, 1980, PI

Lonicera implexa Aiton subsp. **implexa**
P lian/P caesp - Stenomedit.
Literature data: Corti 1956, 1970; Garbari 2001; Bertacchi & Lombardi 2016
Field observations: I. Arduini, 2024

Lonicera japonica Thunb.
P lian - Orig. E-Asia
Literature data: Corti 1956, 1970; Garbari 2001
Herbarium data: L. Pinzani, 2020, Herb. Pinzani
Field observations: L. Pinzani, 2022

Scabiosa uniseta Savi
H bienn - Endem. Ital.
Literature data: Coaro 1987
Assessed as LC in the Red List of the Italian Vascular Flora (Rossi et al. 2020)

Sixalix atropurpurea (L.) Greuter & Burdet
H bienn (T scap) - Stenomedit.
Literature data: Caruel 1860, under the name *Scabiosa atropurpurea* L.; Saggese 2016; Lazzeri 2021
Herbarium data: L. Pinzani, 2020, Herb. Pinzani
Field observations: B. Pierini, 2017; L. Pinzani, 2021

NC **Succisa pratensis** Moench
H scap - Eurosiber.
Literature data: Caruel 1860, under the name *Scabiosa succisa* L.

NC **Valeriana officinalis** L. subsp. **officinalis**
H scap - Europ.
Literature data: Baroni 1897-1908

Valerianella carinata Loisel.
T scap - Eurimedit.
Herbarium data: L. Pinzani, 2021, 2022, Herb. Pinzani
Field observations: L. Pinzani, 2021

NC **Valerianella coronata** (L.) DC.
T scap - Eurimedit.
Literature data: Baroni 1897-1908

NC **Valerianella dentata** (L.) Pollich
T scap - Submedit.-Subatl.

Literature data: Caruel 1860

NC **Valerianella echinata** (L.) DC.
T scap - Stenomedit.
Literature data: Caruel 1860

Valerianella eriocarpa Desv.
T scap - Stenomedit.
Literature data: Caruel 1860; Corti 1956
Herbarium data: L. Pinzani, 2021, Herb. Pinzani
Field observations: A. Mo, I. Pucci, S. Pascacaldi, L. Pinzani, 2022

Valerianella locusta (L.) Laterr.
T scap - Eurimedit.
Literature data: Coaro 1987; Garbari 2001
Herbarium data: L. Pinzani, 2021, Herb. Pinzani
Field observations: B. Pierini, 2014

Valerianella muricata (Steven ex M.Bieb.) W.H.Baxter & Wooster
T scap - Stenomedit.- Orient.
Herbarium data: L. Pinzani, G. Astuti, 2022, Herb. Pinzani

NC **Valerianella rimosa** Bastard
T scap - Eurimedit.
Literature data: Caruel 1860, under the name *V. auricula* Dec.

Pittosporaceae

Pittosporum tobira (Thunb.) W.T.Aiton
NP/P caesp - Orig. E-Asia
Literature data: Corti 1956; Garbari 2001; Bertacchi et al. 2009; Bertacchi et al. 2010; Bertacchi & Lombardi 2014
Field observations: partecipanti all'escursione Wikiplantbase #Parco di Migliarino - San Rossore - Massaciuccoli, 2018; L. Pinzani, 2022

Araliaceae

Hedera helix L. subsp. **helix**
P lian - Submedit.
Literature data: Caruel 1860; Corti 1956, 1970; Gellini et al. 1986; Coaro 1987 [*G. Pistolesi, E. Coaro*, 1977, PI]; Pedullà & Garbari, 2004; Tomei et al. 2004; Bertacchi et al. 2009; Bertacchi et al. 2010; Arduini & Ercoli 2012; Bertacchi & Lombardi 2014a; Lombardi 2015; Bertacchi & Lombardi 2016; Bonari et al. 2019; Lazzeri 2021
Herbarium data: C. Del Prete, 1979, PI; B. Ciacchi, G. Lorè, 1996, PI
Field observations: R. Dell'Orso, 2015; L. Peruzzi, 2017; partecipanti all'escursione Wikiplantbase #Parco di Migliarino - San Rossore - Massaciuccoli, 2018; L. Pinzani, 2020, 2021, 2022; I. Arduini, 2022

Hydrocotyle vulgaris L.

G rhiz/I rad - Europ.-Caucas.
Literature data: Corti 1956; Gellini et al. 1986; Garbari 2001; Tomei et al. 2004; Sani & Tomei 2006; Petraglia 2013
Assessed as EN in the Red List of the Italian Vascular Flora (Rossi et al. 2020)

Apiaceae

Aegopodium podagraria L.
G rhiz - Eurosiber.
Literature data: Caruel 1860
Field observations: L. Pinzani, 2020

Ammi majus L.
T scap - Eurimedit.
Literature data: Caruel 1860
Herbarium data: L. Pinzani, 2020, Herb. Pinzani
Field observations: L. Pinzani, 2020

Anethum foeniculum L.
H scap - S-Medit.
Literature data: Caruel 1860, under the name *Foeniculum officinale* All.; Saggese 2016, under the name *Foeniculum vulgare* Mill.
Herbarium data: L. Pinzani, 2020, Herb. Pinzani
Field observations: B. Pierini, 2014; partecipanti all'escursione Wikiplantbase #Parco di Migliarino - San Rossore - Massaciuccoli, 2018; L. Pinzani, 2020, 2021, 2022

Anethum piperitum Ucria
H scap - Medit.
Literature data: Coaro 1987, under the name *Foeniculum vulgare* Mill. subsp. *piperitum* (Ucria) Coutinho
Herbarium data: F. Picco, 1995, Herb. Picco
Field observations: B. Pierini, 2024

Anethum ridolfia Spalik & Reduron
T scap - Stenomedit.
Field observations: L. Peruzzi, 2024

Angelica sylvestris L. subsp. **sylvestris**
H scap - Eurosiber.
Literature data: Caruel 1860; Baroni 1897-1908
Field observations: L. Pinzani, 2021

NC **Anthriscus caucalis** M.Bieb.
T scap - Paleotemp.
Literature data: Corti 1956, under the name *A. scandicina* (Web.) Mansf.

Anthriscus sylvestris (L.) Hoffm. subsp. **sylvestris**
H scap - Paleotemp.
Literature data: Caruel 1860

Field observations: partecipanti all'escursione Wikiplantbase #Parco di Migliarino - San Rossore - Massaciuccoli, 2018

NC **Apium graveolens** L.
H scap - Medit.
Literature data: Caruel 1860; Baroni 1897-1908; Corti 1956 [*O. Beccari*, s.d., FI; *M. Savelli*, s.d., FI]

NC **Berula erecta** (Huds.) Coville
G rhiz - Circumbor.
Literature data: Caruel 1860, under the name *Sium angustifolium* L.

NC **Bifora testiculata** (L.) Spreng.
T scap - Stenomedit.
Literature data: Caruel 1860, under the name *B. flosculosa* Bieb.

Bupleurum baldense Turra
T scap - Eurimedit.
Literature data: Caruel 1860, under the name *B. aristatum* Bartl.; Peruzzi et al. 2022 [*G. Astuti, A. Cutroneo, F. Roma-Marzio, R. Vangelisti*, 2022, PI]

NC **Bupleurum subovatum** Link ex Spreng.
T scap - Medit.-Turan.
Literature data: Caruel 1860, under the name *B. protractum* Hoffm. et Link

Bupleurum tenuissimum L.
T scap - Eurimedit.
Literature data: Caruel 1860; Coaro 1987
Herbarium data: L. Pinzani, 2020, Herb. Pinzani

Cervaria rivini Gaertn.
H scap - Eurosiber.
Field observations: B. Pierini, 2014

Chaerophyllum temulum L.
T scap - Eurasiat.
Field observations: B. Pierini, 2015

Conium maculatum L. subsp. **maculatum**
H scap/H bienn - Paleotemp.-Subcosmop.
Literature data: Caruel 1860
Field observations: B. Pierini, 2014

Crithmum maritimum L.
Ch suffr - Eurimedit.-Stenomedit.
Literature data: Sani & Tomei 2006; Bertacchi et al. 2009; Bertacchi et al. 2010; Bertacchi & Lombardi 2014a
Field observations: L. Pinzani, 2018; M. D’Antraccoli, 2019; A. Mo, 2023

NC *Cyclospermum leptophyllum* (Pers.) Sprague ex Britton & P.Wilson
Literature data: Caruel 1860, under the name *C. ammi* Lag.

Daucus broteroi Ten.
T scap - Endem. Ital.
Field observations: L. Peruzzi, 2024
Assessed as LC in the Red List of the Italian Vascular Flora (Rossi et al. 2020)

Daucus carota L. subsp. **carota**
H bienn/T scap - Paleotemp.-Subcosmop.
Literature data: Caruel 1860; Coaro 1987; Garbari 2001; Lombardi 2015; Saggese 2016
Field observations: B. Pierini, 2014; partecipanti all'escursione Wikiplantbase #Parco di Migliarino - San Rossore - Massaciuccoli, 2018; L. Peruzzi, 2019; L. Pinzani, 2020, 2021, 2022

Daucus pumilus (L.) Hoffmanns. & Link
T scap - Stenomedit.
Literature data: Caruel 1860, under the name *Orlaya maritima* Koch; Corti 1956 [*C. Rossetti*, s.d., FI]; Corti 1956; Garbari 2001, Bertacchi et al. 2009, under the name *Pseudorlaya pumila* (L.) Grande

Echinophora spinosa L.
H scap - Eurimedit.
Literature data: Caruel 1860; Baroni 1897-1908; Corti 1956 [*S. Sommier*, s.d., FI]; Garbari 2001; Tomei et al. 2004; Sani & Tomei 2006; Bertacchi et al. 2009; Bertacchi & Lombardi 2014a; Bertacchi & Lombardi 2014b

Eryngium campestre L.
H scap - Eurimedit.
Literature data: Caruel 1860; Garbari 2001
Field observations: L. Pinzani, 2022

Eryngium maritimum L.
G rhiz - Medit.-Atl.
Literature data: Caruel 1860; Corti 1956 [*A. Archbald*, s.d., FI; *E. Levier*, s.d., FI]; Corti 1956; Tomei et al. 2004; Sani & Tomei 2006; Bertacchi et al. 2009; Bertacchi et al. 2010; Bertacchi & Lombardi 2014a; Bertacchi & Lombardi 2014b
Herbarium data: L. Pinzani, 2019, Herb. Pinzani
Field observations: partecipanti all'escursione Wikiplantbase #Parco di Migliarino - San Rossore - Massaciuccoli, 2018; R. Righini, 2021; L. Pinzani, 2022

NC **Helosciadium inundatum** (L.) W.D.J.Koch
H scap/I rad - Subatl.-W-Europ.
Literature data: Baroni 1897-1908

Helosciadium nodiflorum (L.) W.D.J.Koch subsp. **nodiflorum**
H scap/I rad - Eurimedit.
Literature data: Caruel 1860
Herbarium data: L. Pinzani, 2020, Herb. Pinzani
Field observations: L. Pinzani, 2021, 2022

D **Oenanthe crocata** L.
Literature data: Caruel 1860

NC **Oenanthe fistulosa** L.
H scap - Eurasiat.
Literature data: Caruel 1860

NC **Oenanthe globulosa** L.
H scap - Stenomedit.-Occid.
Literature data: Caruel 1860; Baroni 1897-1908

Oenanthe lachenalii C.C.Gmel.
H scap - Medit.-Atl.
Literature data: Caruel 1860; Baroni 1897-1908; Corti 1956 [*O. Beccari*, s.d., FI]; Coaro 1987; Petraglia 2013

NC **Oenanthe peucedanifolia** Pollich
H scap - Medit.-Atl.
Literature data: Caruel 1860; Baroni 1897-1908

Oenanthe pimpinelloides L.
H scap - Medit.-Atl.
Literature data: Corti 1956; Gellini et al. 1986; Coaro 1987; Garbari 2001; Lazzeri 2021
Herbarium data: Partecipanti all'escursione Wikiplantbase #Parco di Migliarino - San Rossore - Massaciuccoli, 2018, PI
Field observations: partecipanti all'escursione Wikiplantbase #Parco di Migliarino - San Rossore - Massaciuccoli, 2018; I. Arduini, 2024

Oenanthe silaifolia M.Bieb.
H scap - Medit.-Atl.
Literature data: Corti 1956, under the name *O. silaifolia* M. Bieb. var. *media* (Griseb.) Beck; Coaro 1987

Oreoselinum nigrum Delarbre
H scap - Europ.-Caucas.
Literature data: Caruel 1860, under the name *Peucedanum oreoselinum* Moench
Field observations: observed on iNaturalist in 2021

NC **Orlaya platycarpos** W.D.J.Koch
T scap - Stenomedit.
Literature data: Caruel 1860, under the name *Caucalis daucoides* L.

Pastinaca sativa L. subsp. **sativa**
H bienn - Eurosiber.-Subcosmop.
Literature data: Caruel 1860
Field observations: L. Pinzani, 2021

Petroselinum crispum (Mill.) Fuss

Field observations: L. Pinzani, 2021

NC **Pimpinella peregrina** L.
H bienn - Eurimedit.
Literature data: Caruel 1860

Scandix pecten-veneris L. subsp. **pecten-veneris**
T scap - Subcosmop.
Literature data: Caruel 1860; Pedullà & Garbari, 2004
Herbarium data: L. Pinzani, 2022, Herb. Pinzani

Seseli tortuosum L. subsp. **tortuosum**
H bienn - Stenomedit.
Literature data: Caruel 1860; Bertacchi et al. 2010; Bertacchi & Lombardi 2014a
Herbarium data: L. Pinzani, 2020, Herb. Pinzani

Sison amomum L.
H bienn - Submedit.-Subatl.
Literature data: Caruel 1860; Coaro 1987

Smyrnium olusatrum L.
H bienn - Medit.-Atl.
Literature data: Caruel 1860; Pedullà & Garbari, 2004
Field observations: B. Pierini, 2014; F. Roma-Marzio, 2014; L. Pinzani, 2022

Thysselinum palustre (L.) Hoffm.
H scap - Eurosiber.
Literature data: Tomei et al. 2004, under the name *Peucedanum palustre* Moench

Tordylium apulum L.
T scap - Stenomedit.
Literature data: Corti 1956; Coaro 1987
Field observations: B. Pierini, 2014; L. Pinzani, 2021

NC **Tordylium maximum** L.
T scap - Eurimedit.
Literature data: Caruel 1860

Torilis arvensis (Huds.) Link subsp. **arvensis**
T scap - Subcosmop.
Literature data: Caruel 1860, under the name *T. helvetica* Gmel.; Corti 1956, under the name *T. arvensis* (Hudson) Link sens. ampl. subsp. *divaricata* (Moench) Thellung; Saggese 2016; Lazzeri 2021, 2022
Herbarium data: Partecipanti all'escursione Wikiplantbase #Parco di Migliarino - San Rossore - Massaciuccoli, 2018, PI
Field observations: B. Pierini, 2014; partecipanti all'escursione Wikiplantbase #Parco di Migliarino - San Rossore - Massaciuccoli, 2018

Torilis japonica (Houtt.) DC.

T scap - Paleotemp.-Subcosmop.
Literature data: Coaro 1987; Pedullà & Garbari, 2004
Field observations: partecipanti all'escursione Wikiplantbase #Parco di Migliarino - San Rossore - Massaciuccoli, 2018

Torilis nodosa (L.) Gaertn. subsp. **nodosa**
T scap - Eurimedit.-Turan.
Literature data: Caruel 1860; Sani & Tomei 2006
Herbarium data: A. Sani, 2005, PI; J. Franzoni, 2019, Herb. Franzoni; L. Pinzani, 2020, Herb. Pinzani
Field observations: L. Pinzani, 2020, 2022; H. Öhm, 2024

NC **Turgenia latifolia** (L.) Hoffm.
T scap - Eurimedit.
Literature data: Caruel 1860

NC **Visnaga daucoides** Gaertn.
T scap - Eurimedit.
Literature data: Caruel 1860, Baroni 1897-1908, under the name *Ammi visnaga* Lam.

References

Anzalone B., 1979. La diffusione di *Artemisia annua* L. in Italia. Informatore Botanico Italiano, 10(3) (1978): 394–396.

Anzalone B., Brilli–Cattarini A.J.B., 1980. Segnalazioni Floristiche Italiane: 26. *Cyperus eragrostis* Lam. (Cyperaceae). Informatore Botanico Italiano, 11(2) (1979): 173.

Arduini I., Alessandrini V., 2024. The novel invader *Salpichroa origanifolia* modifies the soil seed bank of a mediterranean mesophile forest. Plants 2024, 13(2): 226.

Arduini I., Ercoli L., 2012. Recovery of understory vegetation in clear-cut stone pine (*Pinus pinea* L.) plantations. Plant Biosystems 146(supplement 1), 244–258.

Arrigoni P.V., 2018. Flora analitica della Toscana. Volume 3. Edizioni Polistampa, Firenze. 533 pp.

Arrigoni P.V., 2019. Flora analitica della Toscana. Volume 6. Edizioni Polistampa, Firenze. 547 pp.

Arrigoni P.V., 2020. Flora analitica della Toscana. Volume 7. Edizioni Polistampa, Firenze. 475 pp.

Baldini R.M., 1993. The genus *Phalaris* L. (Gramineae) in Italy. Webbia, 47(1): 1–53.

Baroni E., 1897–1908. Supplemento generale al Prodromo della Flora Toscana di T. Caruel. Società Botanica Italiana, Firenze.

Bedini G., Carta A., Garbari F., Peruzzi L., 2011. Schede per una Lista Rossa della Flora vascolare e crittogamica italiana: *Hypericum elodes* L. Informatore Botanico Italiano, 43(2): 405–406.

Bernardello R., Giovannini A., Marchetti D., 2003. Notule Pteridologiche Italiche: 46–47. 46. *Asplenium* × *bouharmontii* Badré et Prelli; 47. *Nephrolepis cordifolia* C. Presl. In: Marchetti D. (ed.), Notule Pteridologiche Italiche. II (32–63). Annali del Museo Civico di Rovereto, Sezione: Architettura, Storia, Scienze Naturali, 17 (2001): 109–110.

Bertacchi A., Lombardi T., 2014a. Diachronic analysis (1954–2010) of transformations of the dune habitat in a stretch of the Northern Tyrrhenian Coast (Italy). Plant Biosystems, 148(2): 227–236.

Bertacchi A., Lombardi T., 2014b. *Spartina versicolor* Fabre in coastal areas of Tuscany (Italy). Contribuţii Botanice, 49: 49–60.

Bertacchi A., Lombardi T., 2016. I boschi di Coltano: aspetti storici, fisionomici e vegetazionali di un paesaggio forestale relitto nella pianura di Pisa (Toscana). Atti della Società Toscana di Scienze Naturali, Memorie, Serie B, 122 (2015): 117–128.

Bertacchi A., Lombardi T., Bocci G., 2009. Il paesaggio vegetale dell'ambiente dunale di Calambrone nel litorale pisano (Toscana settentrionale). Informatore Botanico Italiano, 41(2): 281–292.

Bertacchi A., Lombardi T., Tomei P.E., 2007. Le aree umide salmastre della Tenuta di San Rossore (PI): zonazione e successione delle specie vegetali in relazione alla salinità del suolo. Inter Nos, 1: 63–72.

Bertacchi A., Lombardi T., Vecci L., 2010. Gli ambienti dunali di Tirrenia (PI): aspetti vegetazionali e floristici. Inter Nos, 2: 73–80.

Bonari G., Knollová I., Vlčková P., Xystrakis F., Çoban S., Sağlam C., Didukh Y.P., Hennekens S.M., Acosta A.T.R., Angiolini C., Bergmeier E., Bertacchi A., Costa J.C., Fanfarillo E., Gigante D., Guarino R., Landi M., Neto C.S., Pesaresi S., Rosati L., Selvi F., Sotiriou A., Stinca A., Turcato C., Tzonev R., Viciani D., Chytrý M., 2019. CircumMed Pine Forest Database: an electronic archive for Mediterranean and Submediterranean pine forest vegetation data. Phytocoenologia, 49(3): 311–318.

Bottega S., Garbari F., 2003. Il genere *Symphytum* L. (Boraginaceae) in Italia. Revisione biosistematica. Webbia, 58 (2): 243–280.

Carta A., Pierini B., Peruzzi L., 2008a. Aggiornamenti e novità sulla distribuzione di *Isoëtes gymnocarpa* e *I. histrix* (Lycopodiophytina) in Toscana. Atti della Società Toscana di Scienze Naturali, Memorie, Serie B, 115 (2008): 43–45.

Carta A., Pierini B., Peruzzi L., 2008a. Distribuzione di *Ophioglossum lusitanicum* L.(Psilotopsida) in Toscana. Informatore Botanico Italiano, 40(2): 135–141.

Caruel T., 1860. Prodromo della Flora Toscana. Le Monnier, Firenze.

Caruel T., 1870. Secondo supplemento al Prodromo della Flora Toscana di T. Caruel. Firenze.

Coaro E., 1987. Flora e vegetazione del Bosco dell'Ulivo (Parco di Migliarino, S. Rossore e Massaciuccoli). Quaderni del Museo di Storia Naturale di Livorno, 8(1): 5–14.

Corsi G., Garbari F., Maffei F., 1999. Il genere *Urtica* L. (Urticaceae) in Italia. Revisione biosistematica. Webbia, 53(2): 193–235.

Corti R., 1951. *Stipa trichotoma* Nees nella selva di San Rossore, nuovo inquilino della flora toscana. Giornale Botanico Italiano, 58(3–4): 475–486.

Corti R., 1956. Ricerche sulla vegetazione dell'Etruria. X. Aspetti geobotanici della selva costiera. La Selva pisana a San Rossore e l'importanza di questa formazione relitta per la storia della

vegetazione mediterranea. Nuovo Giornale Botanico Italiano, nuova serie, 62 (1–2) (1955): 75–262.

Corti R., 1970. Visita alla Tenuta di San Rossore. In: VV.AA., Escursione sociale in Versilia e sulle Alpi Apuane, 16–19 giugno 1969. Informatore Botanico Italiano, 1(2) (1969): 130–131.

Coulot P., Rabaute P., 2016. Monographie des Leguminosae de France. Tome 4. Tribù des Fabeae, des Cicereae et des Genisteae Société Botanique du Centre-Ouest, Jarnac. 902 pp.

Falciani L., 1997. Systematic revision of *Stachys* sect. *Eriostomum* (Hoffmans. & Link) Dumort. in Italy. Lagascalia, 19(1–2): 187–238.

Ferrarini E., Ciampolini F., Pichi Sermolli R.E.G., Marchetti D., 1986. Iconographia Palynologica Pteridophytorum Italiae. Webbia, 40(1): 1–202.

Fiori A., 1943. Flora Italica Cryptogama, 5. Pteridophyta. Tipografia Mariano Ricci, Firenze.

Galasso G., Conti F., Peruzzi L., Alessandrini A., Ardenghi N.M.G., Bacchetta G., Banfi E., Barberis G., Bernardo L., Bouvet D., Bovio M., Calvia G., Castello M., Cecchi L., Del Guacchio E., Domina G., Fascetti S., Gallo L., Guarino R., Gubellini L., Guiggi A., Hofmann N., Iberite M., Jiménez-Mejías P., Longo D., Marchetti D., Martini F., Masin R.R., Medagli P., Musarella C.M., Peccenini S., Podda L., Prosser F., Roma-Marzio F., Rosati L., Santangelo A., Scoppola A., Selvaggi A., Selvi F., Soldano A., Stinca A., Wagensommer R.P., Wilhalm T., Bartolucci F., 2024. A second update to the checklist of the vascular flora alien to Italy. Plant Biosystems 158: 297–340.

Galasso G., Domina G., Adorni M., Ardenghi N.M.G., Banfi E., Bedini G., Bertolli A., Brundu G., Calbi M., Cecchi L., Cibei C., D'Antraccoli M., De Bastiani A., Faggi G., Ghillani L., Iberite M., Latini M., Lazzeri V., Liguori P., Marhold K., Masin R., Mauri S., Mereu G., Nicolella G., Olivieri D., Peccenini S., Perrino E., Peruzzi L., Petraglia A., Pierini B., Prosser F., Roma–Marzio F., Romani E., Sammartino F., Selvaggi A., Signorile G., Stinca A., Verloove F., Nepi C., 2016. Notulae to the Italian alien vascular flora: 1. Italian Botanist 1: 17–37.

Galasso G., Domina G., Alessandrini A., Ardenghi N.M.G., Bacchetta G., Ballelli S., Bartolucci F., Brundu G., Buono S., Busnardo G., Calvia G., Capece P., D'Antraccoli M., Di Nuzzo L., Fanfarillo E., Ferretti G., Guarino R., Iamonico D., Iberite M., Latini M., Lazzaro L., Lonati M., Lozano V., Magrini S., Mei G., Mereu G., Moro A., Mugnai M., Nicolella G., Nimis P.L., Olivieri H., Pennesi R., Peruzzi L., Podda L., Probo M., Prosser F., Ravetto Enri S., Roma–Marzio F., Ruggero A., Scafidi F., Stinca A., Nepi C., 2018. Notulae to the Italian alien vascular flora: 6. Italian Botanist 6: 65–90.

Galasso G., Domina G., Ardenghi N.M.G., Arrigoni P., Banfi E., Bartolucci F., Bonari G., Buccomino G., Ciaschetti G., Conti F., Coppi A., Di Cecco V., Di Martino L., Guiggi A., Lastucci L., Leporati M.L., Tirado J.L., Maiorca G., Mossini S., Olivieri N., Pennesi R., Romiti B., Scoppola A., Soldano A., Stinca A., Verloove F., Villa M., Nepi C., 2016. Notulae to the Italian alien vascular flora: 2. Italian Botanist 2: 55–71.

Galasso G., Domina G., Angiolini C., Bacchetta G., Banfi E., Barberis D., Bardi S., Bartolucci F., Bonari G., Bovio M., Briozzo I., Brundu G., Buono S., Calvia G., Celesti-Grapow L., Cozzolino A., Cuena-Lombraña A., Curuzzi M., D'Amico F.S., Dagnino D., De Fine G., Fanfarillo E.,

Federici A., Ferraris P., Fiacchini D., Fiaschi T., Fois M., Gubellini L., Guidotti E., Hofmann N., Kindermann E., Laface V.L.A., Lallai A., Lanfredini P., Lazzaro L., Lazzeri V., Lonati M., Loreti M., Lozano V., Magrini S., Mainetti A., Marchini M., Marignani M., Martignoni M., Mei G., Minutillo F., Mondino G.P., Motti R., Musarella C.M., Nota G., Olivieri N., Pallanza M., Passalacqua N.G., Patera G., Pilon N., Pinzani L., Pittarello M., Podda L., Probo M., Ravetto Enri S., Rosati L., Salerno P., Selvaggi A., Soldano A., Sotgiu Cocco G., Spampinato G., Stinca A., Terzi M., Tondi G., Turcato C., Wellstein C., Lastrucci L., 2021. Notulae to the Italian alien vascular flora: 12. Italian Botanist 12: 105–121.

Garbari F., 2001. La Flora di S. Rossore (Pisa) aggiornata al 1999. Atti della Società Toscana di Scienze Naturali, Memorie, Serie B, 107 (2000): 11–42.

Gellini R., Pedrotti F., Venanzoni R., 1986. Le associazioni forestali ripariali e palustri della Selva di San Rossore (Pisa). Documents Phytosociologiques, 10(2): 27–41.

Gestri G., Alessandrini A., Sirotti M., Carta A., Peruzzi L., 2010. Contributo alla conoscenza della flora vascolare endemica di Toscana ed aree contermini. 2. *Bellevalia webbiana* Parl. (Asparagaceae). Informatore Botanico Italiano, 42(2): 423–429.

Iamonico D., Lastrucci L., Viciani D., 2013. Notulae alla cecklist della Flora vascolare Italiana, 15: 1965. Informatore Botanico Italiano, 45(1): 95–96.

Lastrucci, L., Saiani, D., Mugnai, A., Ferretti, G., Viciani, D., 2024. Updating the distribution of the genus *Callitriche* (Plantaginaceae) in Italy from the study of the *Herbarium Centrale Italicum* collections. Mediterranean Botany, 45(2): e87474.

Lazzaro L., Ferretti G., Galasso G., Lastrucci L., Foggi B., 2013. Contributo alla conoscenza della flora esotica dell'Arcipelago Toscano, Italia. Natural History Science, 154 (1): 3–24.

Lazzeri V., 2014. Note floristiche tosco–sarde III: novità per le regioni Toscana e Sardegna. Quaderni del Museo di Storia Naturale di Livorno, 25: 67–77.

Lazzeri V., 2021. Ecology of *Baccharis halimifolia* L. in Tuscany (Italy) and its impacts on native vegetation: where are we and where are we going to? Quaderni del Museo di Storia Naturale di Livorno, 27: 47–67.

Lazzeri V., 2022. Contributo alla flora vascolare delle zone umide di Toscana I. La flora dell'area umida di Stagno (Pisa). In: Buono V., Canzonieri A., Longo D., Nicolella G. (eds.), Acta Plantarum Notes 8: 11–17. Araba Fenice Edizioni, Boves (Cuneo).

Lazzeri V., Mascia F., Sammartino F., Campus G., Caredda A., Carlesi V., Fois M., Gestri G., Mannocci M., Mazzoncini V., Lombraña A.C., Santinelli M., 2013. Novità floristiche per le regioni Sardegna e Toscana. In: Alessandrini A., Buono V., Lazzeri V., Longo D., Magni C., Manni Q.G., Nicolella G. (eds.), Acta Plantarum Notes 2: 42–59. Araba Fenice Edizioni, Boves (Cuneo).

Lazzeri V., Sammartino F., 2013. Noterelle 0079. *Physalis peruviana* L. In: Alessandrini A., Buono V., Lazzeri V., Magni C., Manni Q.G., Nicolella G. (eds.), Acta Plantarum Notes 1: 132. Araba Fenice Edizioni, Boves (Cuneo).

Levier E., Sommier S., 1891. Addenda ad Floram Etruriae. Nuovo Giornale Botanico Italiano, 23: 241–270.

Linnaeus C., 1753. Species Plantarum. Volume 2. Impensis Laurentii Salvii, Holmiae [Stockholm].

Lombardi L. (ed.), 2015. Monitoraggio vegetazionale e floristico delle aree interessate dalle azioni, coordinamento scientifico interventi di compensazione. Relazione Tecnica per progetto allestimento Route Nazionale R–S 2014, Ente Parco Regionale Migliarino San Rossore Massaciuccoli.

Mainardi R., 1982. Alcune considerazioni sulla nidificazione del Cavaliere d'Italia *Himantopus himantopus* (L.) all'Ulivo (Pisa). Quaderni del Museo di Storia Naturale di Livorno, 3: 79–82.

Montelucci G., 1934. L'*Artemisia verlotorum* Lamotte a Roma e in altre località italiane. Nuovo Giornale Botanico Italiano, 41: 242–248.

Montelucci G., 1962. Avventizie nuove e antiche sul litorale pisano-versiliese. Nuovo Giornale Botanico Italiano, nuova serie, 68(3–4) (1961): 406–415.

Montelucci G., 1964. Ricerche sulla vegetazione dell'Etruria XIII. Materiali per la flora e la vegetazione di Viareggio. Webbia, 19(1): 73–347.

Orlandi C., Arduini I., 2010. Note ad integrazione della flora di San Rossore (Pisa). Informatore Botanico Italiano, 42: 473–477.

Parlatore F., 1857. Flora Italiana. Volume 2. Tipografia Le Monnier, Firenze. 638 pp.

Pedullà M.L., Garbari F., 2004. La flora della rete di canalizzazione della pianura nord–occidentale pisana. Quaderni del Museo di Storia Naturale di Livorno, 17: 11–72.

Peruzzi L., 2021. In: Selmi G.: È un'attrazione l'invasione verde dei fiori di loto alle antiche mura. Tirreno Pisa–Pontedera, 11 agosto 2021. 4 pp.

Peruzzi L., Pierini B., Tison J.-M., 2007. Notulae alla checklist della flora vascolare italiana, 5: 1435–1438. Informatore Botanico Italiano, 40(1): 103–104.

Peruzzi L., Galasso G., Domina G., Bartolucci F., Santangelo A., Alessandrini A., Astuti G., D'Antraccoli M., Roma-Marzio F., Ardenghi N.M.G., Barberis G., Conti F., Bernardo L., Peccenini S., Stinca A., Wagensommer R.P., Bonari G., Iamonico D., Iberite M., Viciani D., Del Guacchio E., Giusso del Galdo G.P., Lastrucci L., Villani M., Brunu A., Magrini S., Pistarino A., Brullo S., Salmeri C., Brundu G., Clementi M., Carli E., Vacca G., Marcucci R., Banfi E., Longo D., Di Pietro R., Passalacqua N.G., 2019. An inventory of the names of native, non-endemic vascular plants described from Italy, their loci classici and types. Phytotaxa, 410: 1–215.

Peruzzi L., Viciani D., Adami M., Angiolini C., Astuti G., Bonari G., Bonaventuri G., Castagnini P., de Simone L., Domina G., Fanfarillo E., Fedeli R., Ferretti G., Festi F., Fiaschi T., Foggi B., Franzoni J., Gabellini A., Gennai M., Gestri G., Giacò A., Gottschlich G., Maccherini S., Mugnai M., Pierini B., Pinzani L., Roma-Marzio F., Sarmati S., Vannini A., Zangari G., Bedini G., 2021. Contributi per una flora vascolare di Toscana. XIII (813–873). Atti della Società Toscana di Scienze Naturali, Memorie, Serie B, 128: 85–94.

Peruzzi L., Viciani D., Agostini N., Angiolini C., Ardenghi N.M.G., Astuti G., Bardaro M.R., Bertacchi A., Bonari G., Boni S., Chytrý M., Ciampolini F., D'Antraccoli M., Domina G., Ferretti G., Guiggi A., Iamonico D., Laghi P., Lastrucci L., Lazzaro L., Lazzeri V., Liguori P., Mannocci M., Marsiaj G., Novák P., Nucci A., Pierini B., Roma-Marzio F., Romiti B., Sani A., Zoccola A.,

Zukal D., Bedini G., 2017a. Contributi per una flora vascolare di Toscana. VIII (440–506). Atti della Società Toscana di Scienze Naturali, Memorie, Serie B, 123 (2016): 71–82.

Peruzzi L., Viciani D., Angiolini C., Apruzzese M., Banfi E., Bonini I., Bonari G., Calvia G., Carta A., Castagnini P., Chierchini F., D'Antraccoli M., Ferretti G., Ferruzzi S., Festi F., Fröhner S., Franzoni J., Galasso G., Gestri G., Gottschlich G., Lazzaro L., Lazzeri V., Mannucci N., Marchetti D., Mugnai M., Pasquinelli P., Pinzani L., Reduron J.-P., Roma-Marzio F., Romanacci G., Romano O., Selvi F., Soldano A., Stinca A., Verloove F., Bedini G., 2020. Contributi per una flora vascolare di Toscana. XII (739–812). Atti della Società Toscana di Scienze Naturali, Memorie, Serie B, 127: 101–112.

Peruzzi L., Viciani D., Angiolini C., Astuti G., Banfi E., Bardaro M.R., Bianchetto E., Bonari G., Cannucci S., Cantini D., Castagnini P., D''Antraccoli M., Esposito A., Ferretti G., Fiaschi T., Foggi B., Franceschi G., Galasso G., Gottschlich G., Lastrucci L., Lazzaro L., Maneli F., Marchetti D., Marsiaj G., Mugnai M., Roma-Marzio F., Ruocco M., Salvai G., Stinca A., Bedini G., 2018. Contributi per una flora vascolare di Toscana. X (606–663). Atti della Società Toscana di Scienze Naturali, Memorie, Serie B, 125: 67–76.

Peruzzi L., Viciani D., Angiolini C., Astuti G., Banfi E., Benocci A., Bonari G., Bruni G., Caramante P., Caré M., Carta A., Castagnini P., Cheli A., Ciampolini F., D'antraccoli M., Ferretti G., Ferruzzi S., Fiaschi T., Foggi B., Fontana D., Galasso G., Gallo L., Galvani D., Gestri G., Grazzini A., Lastrucci L., Lazzaro L., Loppi S., Manganelli G., Mugnai M., Piazzini S., Pierini B., Roma-Marzio F., Sani A., Selvi F., Soldano A., Stinca A., Bedini G., 2017b. Contributi per una flora vascolare di Toscana. IX (507–605). Atti della Società Toscana di Scienze Naturali, Memorie, Serie B, 124: 73–86.

Petraglia A., 2013. Consulenza specialistica per l'acquisizione di dati mediante rilievi fitosociologici e floristici delle principali zone umide del Parco Migliarino – San Rossore – Massaciuccoli. Relazione Tecnica, Parco Regionale di Migliarino – San Rossore – Massaciuccoli. 87 pp.

Pierini B., 2011. Notulae alla flora esotica d'Italia, 5: 99. Informatore Botanico Italiano, 43(2): 374.

Pierini B., 2011. Segnalazione 168-169. In: Peruzzi L., Viciani D., Bedini G. (eds.), Contributi per una flora vascolare di Toscana. III (143–180). Atti della Società Toscana di Scienze Naturali, Memorie, Serie B, 118: 39–46.

Pierini B., 2013. Notulae alla checklist della flora vascolare italiana, 16: 2010. Informatore Botanico Italiano, 45(2): 303–304.

Pierini B., 2013. Segnalazione 263. In: Peruzzi L., Viciani D., Bedini G. (eds.), Contributi per una flora vascolare di Toscana. V (247–319). Atti della Società Toscana di Scienze Naturali, 120: 35–44.

Pignotti L., 2003. *Scirpus* L. and related genera (Cyperaceae) in Italy. Webbia, 58 (2): 281–400.

Roma-Marzio F., D'Antraccoli M., 2016. Segnalazioni 432–434. In: Peruzzi L., Viciani D., Bedini G. (eds.), Contributi per una flora vascolare di Toscana. VII (357–439). Atti della Società Toscana di Scienze Naturali, Memorie, Serie B, 122: 61–72.

Rossi G., Montagnani C., Gargano D., Peruzzi L., Abeli T., Ravera S., Cogoni A., Fenu G., Magrini S., Gennai M., Foggi B., Wagensommer R.P., Venturella G., Blasi C., Raimondo F.M., Orsenigo S., 2013. Lista Rossa della Flora Italiana. 1. Policy Species e altre specie minacciate. Comitato Italiano IUCN e Ministero dell'Ambiente e della tutela del Territorio e del Mare.

Rossi G., Orsenigo S., Gargano D., Montagnani C., Peruzzi L., Fenu G., Abeli T., Alessandrini A., Astuti G., Bacchetta G., Bartolucci F., Bernardo L., Bovio M., Carta A., Castello M., Cogoni D., Conti F., Domina G., Foggi B., Gennai M., Gigante D., Iberite M., Lasen C., Magrini S., Nicolella G., Pinna M.S., Poggio L., Prosser F., Santangelo A., Selvaggi A., Stinca A., Tartaglini N., Troia A., Villani M.C., Wagensommer R.P., Wilhalm T., Blasi C., 2020. Lista Rossa della Flora Italiana. 2 Endemiti e altre specie minacciate. Comitato Italiano IUCN e Ministero dell'Ambiente e della tutela del Territorio e del Mare.

Saggese A., 2016. Studi floristici e vegetazionali delle aree umide salmastre della Toscana settentrionale: il caso di Galanchio. Tesi di laurea magistrale in Conservazione ed Evoluzione, Università di Pisa, 2015–2016.

Sani A., D'Antraccoli M., Peruzzi L., 2015. *Sporobolus cryptandrus*. In: von Raab-Straube E., Raus T. (eds): Euro+Med–Checklist Notulae, 4 [Notulae ad floram euro–mediterraneam pertinentes 33]. Willdenowia, 45: 125.

Sani A., Tomei P.E., 2005. *Aeluropus litoralis* (Gouan) Parl. (Poaceae), *Euphorbia pithyusa* L. subsp. *pithyusa* (Euphorbiaceae), *Juncus gerardi* Loisel. (Juncaceae), *Senecio willdenowii* Peruzzi et N.G. Passal. (Asteraceae). In: Sezione Toscana della Società Botanica Italiana, Notule floristiche per la Toscana: 24–26. Edizioni ETS.

Sani A., Tomei P.E., 2006. La vegetazione psammofila del litorale di San Rossore (Toscana settentrionale) e la sua importanza conservazionistica. Parlatorea, 8: 99–119.

Sani A., Tomei P.E., 2009. Segnalazione 084. In: Peruzzi L., Viciani D., Bedini G. (eds) Contributi per una flora vascolare di Toscana. I. (1–85). Atti della Società Toscana di Scienze Naturali, Memorie, Serie B, 116: 33–44.

Särkinen T., Poczai P., Barboza G.E., Weerden G.M. van den, Baden M., Knapp S., 2018. A revision of the Old World Black Nightshades (Morelloid clade of *Solanum* L., Solanaceae). PhytoKeys 106: 1–223.

Savelli M., 1915. Brevi notizie su alcune piante naturalizzatesi nei dintorni di Pisa. Bullettino della Società Botanica Italiana, 22 (8–9): 81–84.

Savi G., 1798. Flora Pisana. Volume 1. Pietro Giacomelli, Pisa. pp. 489

Soldano A., 1993. Il genere *Oenothera* L., subsect. *Oenothera*, in Italia (Onagraceae). Natura Bresciana, 28 (1992): 85–116.

Soldano A., 2014. Segnalazioni 322–328. In: Peruzzi L., Viciani D., Bedini G. (eds) Contributi per una flora vascolare di Toscana. VI (320–356). Atti della Società Toscana Scienze Naturali, Memorie, Serie B, 121: 29–35

Tomei P.E., Bertacchi A., Sani A., Consiglio M., 2004. La vegetazione della Tenuta di S. Rossore. Note esplicative della Carta della Vegetazione di San Rossore 1:10000. Pacini Ed., Pisa

Tomei P.E., Camangi F., 2014. Tradizioni alimurgiche in Toscana. Pacini Fazzi Editore, Lucca. 15–16; 19; 23–24; 28–30; 33–36 pp.

Welti E.M., Ansaldi M., Carta A., Bedini G., 2011. Distribuzione del genere *Epipactis* (Orchidaceae) in provincia di Pisa. Atti della Società Toscana di Scienze Natutali, Memorie, Serie B, 117 (2010): 65–69.
